# 120th Virtual Annual Meeting and Conference Medical Library Association, Inc. May 19, 2020, and July 27–August 28, 2020

**DOI:** 10.5195/jmla.2021.1169

**Published:** 2021-01-01

**Authors:** JJ Pionke, Ellen M. Aaronson

**Affiliations:** 1 pionke@illinois.edu, Proceedings Coeditor and Applied Health Sciences Librarian, University Library, University of Illinois at Urbana-Champaign, 1408 West Gregory, Urbana IL 61801; 2 aaronson.ellen@mayo.edu, Proceedings Coeditor and Librarian, Mayo Clinic Libraries, 200 First Street SW, Rochester, MN 55905

## INTRODUCTION

The Medical Library Association (MLA) held its 120th annual meeting and conference virtually May 19, 2020, and July 27–August 28, 2020, due to the coronavirus disease 2019 (COVID-19) pandemic. The meeting was titled the “MLA'20 vConference and Exhibits,” and the theme was “2020 Vision: The Future in Focus.” Because of the pivot from an in-person meeting to a virtual conference, the

conference was broken into segments, all available using a variety of online platforms. Total attendance was 1,074. Additional content including the program and various electronic presentations from the business meetings and plenary, poster, and program sessions can be accessed by all meeting registrants via the MLA'20 vConference website.

## MLA 2020 BUSINESS MEETING (PLENARY 1)

### Tuesday, May 19, 2020, 1:00 p.m.–2:25 pm, central

The MLA 2019/20 Presidential Address was held prior to the MLA '20 vConference and virtually due to the COVID-19 pandemic.

Debra Cavanaugh: Welcome, everyone, and thanks for joining the MLA Annual Business Meeting. A couple things to mention before we get started. We have muted your phones and mics today. You will see and hear from MLA's president, president-elect, the executive director, and a few others. We have a lot of members participating today, so we ask that you use the chat panel for MLA Business Meeting discussion only.

If a motion needs to be discussed, at that time, we will ask members to raise their hands and we will call on you. You'll be unmuted and be able to ask your question. If we cannot hear you, you will be asked to submit your question in the chat panel. And we will practice raising hands during the meeting. If you're participating only by phone today, you'll be able to raise your hand by pressing *9 and then *9 to lower it again.

We will be using Polling Promotions during the meeting, and we'll have a practice poll to make sure that you know where to go and can vote. Members will have opportunities to ask questions during the MLA open forums held on Tuesdays in May and June. And, finally, we are recording the business meeting today and will make that available soon.

The session continued with Kevin Baliozian, executive director of MLA.

Kevin Baliozian: Thank you for joining us. Earlier this month, the US Supreme Court live streamed to the public its first phone hearing. Well, today, at MLA, we hold our first virtual annual business meeting, and this is indeed an historic month.

COVID-19 is changing the way we are meeting and interacting, and you have been at the forefront of that change. It has been a stressful three months full of disruptions and uncertainties. The word “chaos” comes to mind. In 2019, MLA initiated its own disruptions with the many initiatives you will hear about in a few minutes. In 2020, COVID-19 is doing the job for us. So, MLA is adapting, transforming, experimenting, venting—just all of the things that you're doing in your work and in your personal lives.

We thank the Board of Directors, the 2020 National Program Committee, the tireless headquarter staff, and all of you, so committed and able to envision and to pivot to a new normal. Support and your dedication to the profession and MLA has been exemplary. We salute your spirit, your energy, and we look forward to seizing the moment to excel and set a course to a bright, albeit uncertain, future.

We have been fortunate this year to have had Julia Esparza, AHIP, as our leader in this tumultuous year. It is with great pleasure that I introduce your 2019/20 president, Julia Esparza, AHIP.

Julia Esparza, AHIP: Good afternoon, I am pleased to officially welcome you to the 120th Annual Business Meeting of the Medical Library Association! It is wonderful to know that so many of you are with us today participating in our first virtual annual meeting. Welcome!

As you now know, our annual meeting will be held virtually later this summer (we will call it the MLA '20 vConference). We are looking forward to the exciting new ways we will experience contributed content and keynote sessions, interact with presenters and colleagues, and connect with vendors via virtual exhibits, education sessions, and one-on-one video chats.

The 2020 National Program Committee, Chair Janna C. Lawrence, AHIP, Cochair Melissa De Santis, AHIP, and MLA headquarters staff are hard at work crafting the details of this innovative program. This is an opportunity for the new MLA '20 vConference to reach a larger and more diverse audience than our usual face-to-face format. We will learn a lot from this common experience.

I have invited Janna and Melissa to provide us with a preview of the vConference in a few minutes. Kevin will also provide details in his executive director report.

The format for this annual business meeting session will be quite different as well, as we are holding it separate from the MLA '20 vConference that will be held later this summer. We are meeting now to comply with the May rotation of our association year. We also want to stay connected and not wait until this summer to interact.

Here is an overview of what we will be doing today:

- Preview of the vConference
- Outgoing Presidential Address by Julia Esparza, AHIP
- Annual Business Meeting
- Incoming Presidential Inaugural Address by Lisa K. Traditi, AHIP

In May and June, please join us for a vConnections Association Series, including the Chapter Council and Communities Council annual meetings and six open forums. That is the place for deep dives into MLA strategic initiatives and question-and-answer sessions.

Later this summer during the MLA '20 vConference, we will recognize the many contributors to the 2020 meeting, the vendors who support us during the year, and the award recipients and other honorees who have demonstrated their excellence to our profession.

I'm sure many of you have lots of questions. Because the annual business meeting format is so formal (it's not a “town hall”), do connect throughout the year with board members, committee chairs, and committee members; attend the open forums that are specifically designed for dialogue; communicate with your community and chapter council representatives; contact the MLA headquarter staff who are there to inform and help; and yes, please read *MLAConnect* as well.

I hope that you will understand that because of the formal business meeting format, I will only address questions from members in the method defined by the MLA Bylaws and Robert's Rules of Order. That will be explained by our parliamentarian in a few minutes.

Kevin Baliozian: Thank you, Julia. I am delighted to have this opportunity to express my gratitude for your strong leadership. It has been a pleasure to work with you in this past year.

The MLA Board, headquarters staff, and I have found comfort and leadership in your cool-cat style, your active listening, your decisiveness, and your focus in all situations including the most challenging ones.

Though you may deny it, you do have a sense of humor, and I can totally relate to your occasional sarcasm. You are a straight shooter and never shy away from a necessary critical conversation.

Your vision for MLA is anchored in your expert knowledge and your experience of the health information profession, your broad and deep connection with MLA members, and your curiosity and your ability to always think outside of the box.

On your Facebook profile, you write that you are “a librarian who loves to teach and promote excellent patient care.” Toward that goal, you have spent the last twelve years in the Health Sciences Library and Department of Medical Library Sciences at Louisiana State University Health Sciences Center (LSU)–Shreveport.

You have been an excellent role model for medical librarians who want to engage in scholarly activities, and you have actively supported the educational needs of health care providers, students, library personnel, and the community through instructional efforts.

Julia, in recognition of your accomplishments, you were named Stafford and Marianne Comegys Endowed Professor in Medical Library Science. Your MLA peers have honored you with the President's Award, the Lucretia W. McClure Excellence in Education Award, and the Estelle Brodman Award for Academic Medical Librarian of the Year.

Change was the major theme of your 2019 inaugural address. You are recognized by your colleagues as being “fearless and tireless” in times of change and going above and beyond to see that everyone is prepared to embrace change. We can certainly vouch for that.

Here to talk to us about MLA's 2019/20 year, please welcome again, Julia Esparza.

## MLA 2020 PRESIDENTIAL ADDRESS

Julia Esparza: Thank you, Kevin. As outgoing president, I have the honor and pleasure to summarize MLA's 2019/20 association year and recognize the many individuals who have spearheaded positive change in our association.

Today, I want to talk to you today about how MLA has hit the lotto. As you may remember, last May using the metaphor of pennies, I emphasized how you as individual members bring diversity and value to MLA. At the end of my speech, I called on members to actively participate in the communities transition, be involved through volunteering, respond to surveys, and be a part of general MLA service. I was not disappointed. A wealth of volunteers worked hard to position MLA for future continued success.

I want to spend my time talking about your work for MLA over the past year. I do want to emphasize that while I may be highlighting a few activities in this speech, there are twenty to thirty more activities taking place each year involving many additional members. That work is just as important in making MLA great. Unfortunately, or fortunately as some staff might say, they only give me fifteen minutes. The committees, juries, ad hoc committees, and task forces accomplish amazing goals. It is always important to remember that these groups rely on caucuses and individual members to accomplish much of that work. Only through your involvement can MLA continue to thrive.

The Ad Hoc Committee to Review Core Clinical Journals was charged with creating a possible strategy to revise the National Library of Medicine's current Abridged Index Medicus or the Core Clinical Journal list, which had 118 titles. The committee spent 5 years culling through data from government agencies, patient discharges, institutional journal usage, and outside sources. From the original journal list, Medical Subject Headings (MeSH) expanded from 47 to 80 to represent growing clinical areas. Then, utilizing the journal information available from those previous resources, the committee identified 253 journals that might have clinical relevance. The preprint report will be available in the coming weeks. We thank the committee for their excellent work to create a possible strategy for identifying clinically useful literature as it changes in the future.

The Annual Meeting Innovation Task Force was charged with examining the education, contributed content, association life, and networking of the annual meeting to align with MLA's initiatives in education, communities, diversity and inclusion, credentialing, scholarships, and integrated content. This task force couldn't have come at a better time with the significant changes facing the 2020 National Program Committee. To gather information, they had 7 focus groups and conducted a survey that obtained 775 responses. They are still compiling data but will share information at the open forum on Annual Meeting Innovation on Tuesday, June 9, from 1:00 p.m. to 2:00 p.m., central. Thank you, task force members for your hard work.

We did it! We have completed the transition to the new communities organization for MLA. Close to sixty members helped establish goals for the seven domain hubs to work on going forward. The caucus delegates and members are ready to work on innovative projects. For me, the most exciting result of the communities transition is that in 1 year, by removing barriers, there has been a 37% increase in caucus participation by membership.

Members will be better served as caucuses have merged together, several have changed names, and 2 new caucuses have been created. The domains and caucuses have great plans for the coming year. Make sure you check out the open forum on Communities on Tuesday, May 26, from 1:00 p.m. to 2:00 p.m., central. Thank you everyone for your diligent work to create a new future for MLA.

The Diversity and Inclusion Task Force conducted a survey to which 850 members responded. A survey preprint has been posted to MLANET. In addition, they spent a significant amount of time analyzing the language that MLA used in our vision, mission, values, and code of ethics statements. After the analysis, the committee submitted a motion to the board, which was approved, to revise the language used by MLA to promote more diversity and inclusion. They also presented to the board a motion that was approved again for the establishment of a new diversity, equity, and inclusion standing committee to ensure MLA continues to move forward. Please review their report and join them at the open forum on Diversity and Inclusion on Tuesday, June 2, from 1:00 p.m. to 2:00 p.m., central. At the forum, learn more about their excellent work for our association and your responses to their questions, and ask any questions you may have. We thank this group for their phenomenal work.

In the past year, the InSight Initiative Task Force held three summits. MLA's InSight Initiative engages health sciences librarians and information providers in high-level, high-value dialogue on issues that matter to both our communities. It aims to reinforce, expand, and communicate the value that we contribute in the chain of moving information from author to reader. Beneficial results from the summits provided the board with the data to create a standing committee to continue this valuable work. I highly recommend you check out the InSight Initiative page on MLANET to read all of the available summit reports and to learn more about this project.

We thank those on the initial task force for their dedication to the InSight Initiative, those who have helped plan or participated in the three summits that occurred in the past year, those who supported the initiative financially, and to Gerald J. Perry, AHIP, FMLA, for chairing this task force and creating an environment for success.

The Research Training Institute (RTI) was developed to provide practicing health sciences librarians with an opportunity to immerse themselves in instruction and focused activities related to scholarly research, inquiry, and publishing—three things I love. RTI developed out of the MLA Research Imperative Task Force action plan, which was endorsed by the Board of Directors in 2016. In 2017, the Institute of Museum and Library Services (IMLS) awarded a three-year grant to implement RTI. The IMLS funding, section and chapter funds, and contributions by other organizations helped keep the cost of RTI low. This was to reduce barriers to participation for those who may lack professional development budgets. Thank you to faculty who have taken time to train these eager new researchers, the consultants, advisors, and to Susan Lessick, AHIP, FMLA, for spearheading this vital initiative.

There are currently 1,228 members of the Academy of Health Information Professionals. Yay! The academy allows us to show the incredible work we do in teaching, scholarship, and learning. I encourage you to think about how the academy can provide you a road map to keep your professional development on track.

Thank you to the members of the Credentialing Committee (sorry, I didn't have room for all of your names on this) for working so hard to revise the points index to reflect new roles for the communities and on reviewing 231 applications in the past year. Your dedication is appreciated by all those who participate.

It is also exciting to report that over 684 individuals have earned the Consumer Health Information Specialization, and 151 have earned our newer Disaster Information Specialization. In the 7 education committees, there are over 60 individuals working hard to bring the most exciting education to you. Join the groups in the open forum on Education on Tuesday, June 16, from 1:00 p.m. to 2:00 p.m., central. During the forum, you can learn about the 2 new specializations in the works, the fact that MLA can now offer Illinois continuing nursing education, the many exciting educational opportunities on the horizon, and how you, as MLA, can share your knowledge through teaching.

The Books Panel under the leadership of Carolann Lee Curry, chair, approved three books to go from proposal to contract. Two books are on event planning in libraries, and one is on the role of medical librarians on institutional review boards and committees.

Christine Willis, AHIP, as editor of *MLAConnect,* made sure you had the information needed to function in these turbulent times. The highest viewed articles for the year were those on COVID-19, obviously, and the continuation of the Electronic Funds Transfer System (EFTS).

The *Journal of the Medical Library Association (JMLA)* will be fortunate to have Katherine Goold Akers serving as editor for another three years, as she was reappointed to another tenure. In the past year, *JMLA* reached a major milestone on October 1, 2019, when the journal implemented a new data-sharing policy. Thank you to the 220 peer reviewers who took the time to make sure the quality of the journal continues. In addition, thank you to the members of the Books Panel, the *MLAConnect* Editorial Board, and the *JMLA* Editorial Board for your hard work to make sure these publications are of the highest quality. Please learn more about how these groups will be aligning with the new domain structure and about some other exciting activities they have planned at the open forum on Publications on Tuesday, June 30, from 1:00 p.m. to 2;00 p.m., central.

There are over 80 individuals serving on award, grants, and scholarship committees and juries. This year, over 220 grant and scholarship applications and 66 award nominations were received. The review of this material takes many hours for the committee and jury members. While I will have the privilege of announcing the President's Award winners later in this meeting, I can't wait for the Awards Ceremony during the MLA '20 vConference. For me, it is always a highlight to see how hard our members plan to work through grants and scholarships or what our many talented members and groups have achieved by their awards. Thank you to all who have served on these committees and juries in the past year.

MLA issued several statements on the health and welfare of medical librarians during COVID-19. In addition, with the Association of Academic Health Sciences Libraries, MLA submitted testimony on the fiscal year 2020 National Library of Medicine funding.

Around fifty people a year help prepare comments on federal topics. I highly recommend you read the comments produced by MLA members on these important issues. Watch in the future for calls for members to prepare comments as part of future work groups. Thank you to those who worked on these documents to make sure health sciences librarians had a voice when it comes to these topics.

COVID-19 has obviously made our year more challenging. I would like to recognize three members—Jess L. Callaway, Angela Spencer, AHIP, and Ellen Aaronson, AHIP—on their work to create the shared knowledge resource, COVID-19 Resources for Medical Librarians and Other Health Information Professionals. This is an excellent example of a group coming together to provide a quick response to an information need that benefits us all. Thank you for your hard work. In addition, thanks to those other individuals who submitted material and continue to submit material for them to review and add to the page.

Back in 1998, I worked at ECRI, and I was in charge of installing an EFTS upgrade for the interlibrary loan assistant, and I handled problems with the software. I continued to rely on EFTS when I began working as a hospital librarian in 2002, and at my current institution, we rely on EFTS to handle our financial transactions. Imagine my horror to learn that EFTS might go away. MLA knew this resource was vital to many members. Those participating in the new EFTS look forward to safer interlibrary loan financial transactions. I am excited that the system implementation will be over the summer. Kevin Baliozian, executive director, will talk a bit more about EFTS in his executive director report.

I thank my colleagues on the 2019/20 Board of Directors. We have had an exciting year, maybe a little too exciting as far as I'm concerned, and each of you have provided patience, intelligence, humor, and heart in all the work you did.

Thank you to the MLA staff who work hard to make sure that MLA continues to be the best organization for health sciences librarians.

To close my presentation, I have a few additional thank yous. Again, thanks to Jo Adams who set me on the path to librarianship at age eight through her innovative teaching and wonderful ability to engage students. I also knew then that I never wanted to be a school librarian and deal with kids like me. Thanks to my husband Michael Hackett, who has put up with so much chaos over the year, and to my son, who is oblivious to the chaos most of the time but from time to time picks up that I am stressed. To the wonderful faculty and staff that I work with at LSU–Shreveport, I thank you for your support and patience with my multiple online meetings, projects, and general stress. Finally, I thank those of you who provided me with this opportunity to serve you to the best of my ability.

Now, on to one of the most exciting things I will get to do during my presidency. Each year, the president has the privilege of honoring health sciences librarians who have made outstanding contributions to the profession. As this will be my last meeting as president, I am extremely happy to present the following awards. I look forward to the future when I can see each honoree in person and shake their hand. In addition to this presentation, this year's recipients were recognized in the March 26 *MLAConnect* and can be seen in the slides that follow.

Susan Lessick, AHIP, FMLA, has driven MLA's research vision since 2015, serving as chair of the Research Imperative Task Force and establishing MLA's Research Training Institute.

Susan, your energy and commitment to providing research training and opportunities is priceless to MLA as the fellows participating in the RTI are the next generation of leaders and researchers. They will be able to provide the evidence and research behind many health information services going forward. Susan's leadership brought a success that went far beyond the initial initiative established by the MLA Board.

The RTI was partially funded by a three-year IMLS grant that ends this June. Susan is leading the effort for a second three-year IMLS grant with a focus on expanding on the current RTI with an innovative virtual instructor-led curriculum. We want to scale up our research training to more librarians. Susan and her faculty are hard at work planning the virtual teaching at this year's RTI session, which will be held online in July.

Thank you, Susan.

Gerald J. Perry, AHIP, FMLA, University of Arizona Libraries, University of Arizona–Tucson, has championed the MLA InSight Initiative since its inception in early 2016. Jerry, under your leadership, the InSight Initiative has grown from an inspirational concept to a structured program that has earned praise from both librarians and participating organizations. Since 2018, there have been four in-person summits and one virtual summit. The March 2020 summit was shifted to a virtual meeting due to the COVID-19 pandemic. Jerry, you have been an ambassador, thought leader, and guide, generously contributing your time to steering the InSight Initiative Task Force and actively participating in the InSight Initiative work groups and summits.

Yesterday, the MLA Board approved a motion that establishes the InSight Initiative Steering Committee as a standing committee in charge of continuing this initiative. The committee will be composed of an equal number of librarians and representatives of the publishing industry, another big step in setting MLA up as the place for dialogue between our two communities.

Thank you, Jerry.

Several committees, task forces, and members are recognized today for leading the change in MLA's fundamental structure in ways that have not happened since the 1970s.

In 2014, the Futures Task Force presented their final report to MLA with a call for change. The report identified issues in MLA that hindered the full involvement of membership. The report called for a reorganization to revitalize MLA to make it more inclusive, diverse, and welcoming.

The Strategic Priorities Task Force in 2015–2016 examined the Futures Task Force report and prioritized the recommendations for potential implementation.

Through 2016 and 2017, the Rising Stars conducted action research, with their Rising Stars mentors' guidance, to identify ways to accomplish the goals that the Futures Task Force developed and to complement the implementation and recommendations of the Strategic Priorities Task Force.

Using the previous work as a starting point, the Communities Strategic Goal Task Force from 2016–2019 developed a plan to ensure the reorganization of MLA in a way that would keep the diverse identities of the many subgroups and would allow the organization to become more inclusive, welcoming, and responsive to members.

The MLA Board of Directors approved the strategy that the Communities Strategic Goal Task Force proposed and appointed the Communities Transition Team to bring the future of MLA to reality. Even in the face of sometimes challenging interactions, everyone from 2013 to the present put MLA first.

Twenty twenty and the years leading up to it have been an exceptional time of transition and change for our profession. I am deeply honored to present all of them and recognize their distinguished accomplishments.

Don't miss the Communities open forum on Tuesday, May 26, 1:00 p.m. to 2:00 p.m., central time.

Thank you all for your service.

## MLA 2020 BUSINESS MEETING (PLENARY 1) (CONTINUED)

Here to give us a preview of this summer's MLA '20 vConference, please welcome 2020 National Program Committee Chair Janna C. Lawrence, AHIP.

Janna Lawrence, AHIP: Hi! I'm Janna Lawrence, and I'm the chair of this year's National Program Committee (NPC). Melissa De Santis, AHIP, is the cochair of the NPC, which is made of seventeen of your fellow MLA members, plus another twenty or so on the Local Assistance Committee (LAC). We have been planning, and replanning, this meeting for two years, and originally thought that our last Zoom meeting would be a week ago last Friday. Instead, we are still Zooming.

One thing I am sure of is that switching an already planned in-person meeting to a virtual one is not the ideal way to plan a virtual meeting. Nonetheless, with a tremendous amount of assistance from the staff at MLA headquarters, we are doing just that. We are figuring out how to reproduce, virtually, the interactivity and social aspects that are so important when you attend MLA. We're figuring out ways to preserve that give-and-take with paper and poster presenters, and how to retain the synergy of immersion sessions. We want to be able to interact with and learn from our exhibitors. We are even planning virtual social events.

I think we've all discovered in the last couple months that there are ways to do things that we hadn't considered before. They might not be ideal—I would really love to see all of you in person, just like I would love to see my coworkers and my library's users in person—but we can make it work.

The last time the physical MLA meeting was cancelled was during World War II, when there was no MLA meeting in 1942, 1943, or 1944. Think about that: four years without seeing each other! And at that time, there was really no other way to meet. I checked the issues of the *Bulletin of the Medical Library Association*, the *Journal of the Medical Library Association's (JMLA's)* predecessor, for those years, wondering if there had been some sort of print-based conference, and what I found were committee reports from each year.

I know that the members from those years would be amazed at what we can do now. We are learning as we go, just like many of us are learning how to run our libraries remotely as we do it, and we hope that you find the results educational and enjoyable. And I really hope to see you all in person next year in Washington, DC. Thank you.

Julia Esparza: Next up, for the association's 2020 Annual Business Meeting, I recognize Chris Shaffer, AHIP, MLA's parliamentarian, who will assist us with the business portion of our meeting.

Chris Shaffer, AHIP: Hello, fellow MLA members. As MLA's parliamentarian, to support this groundbreaking electronic business meeting, I had the pleasure to look into the WebEx features that allow questions and voting. Before I get started in my official role, let's practice together some of the WebEx features to make sure that you're familiar with them before the real deal.

First, let's practice hand raising. During the meeting, there will be times when you will be asked to raise your hand using the “raise hands” feature in WebEx. This will happen when members wish to speak to an issue on the floor. Now, let's practice. Everyone, please find the “hands raised” icon on the right-hand side of your screen. You might need to open the Participants panel by clicking the round “head” icon on your WebEx screen. Please click on the “hands raised” icon. Great. No further action is needed. The moderator will tally the count of hands and share them with the presiding officer the names of members who wish to speak when the time comes.

So, now, let's practice voting. First, a motion is introduced. Then, the presiding officer restates the motion to ask if there is discussion. There are about eight more steps. After each person raises their hand, they will have two minutes to speak, and then, after all members have had a chance to speak, each member will be given one additional minute to speak if they so desire.

Now, let's practice. A poll should have appeared on your screen. If you don't see the poll, look on the right side of your screen or click the circle with three dots at the bottom of your screen and choose “polling.” You have two minutes to vote yes, no, or abstain. Do you like anchovies on your pizza? As a vegetarian, I'm going to choose to abstain from this question. Don't forget to push the “submit” button to enter your vote.

After the vote, for a normal vote, the sergeant-at-arms would announce the vote count. Today, I will take this opportunity while you're casting your practice vote to mention that Robert's Rules of Order allow business to be conducted by unanimous consent, which removes the need for discussion and a full vote. Any member present may object to unanimous consent and require the president to open the floor for discussion and put the question to the members for a vote. Today, we plan to use unanimous consent and will use the WebEx voting tool to allow members to register an objection.

Now that we are all WebEx pros, let's get back to business. And it looks like 301 of us, 70%, do not like anchovies, and 11% abstained, so only 17% of you are the people who like fish on pizza.

So, here we go.

As MLA's parliamentarian and to support this groundbreaking electronic business meeting, I reviewed the MLA Bylaws, Robert's Rules of Order, the motion to hold the 2020 Business Meeting Online, various documents related to the content of previous MLA meetings, MLA's Parliamentary Procedures, and Sample Rules for Electronic Meetings by the authors of Robert's Rules. Our Bylaws Committee also read many of these documents.

My assessment, which is supported by the MLA Bylaws Committee, is that current plans for holding the MLA 2020 Business Meeting electronically on May 19, 2020, in other words, today, complies with the bylaws and parliamentary rules. Maryland, where MLA is incorporated, generally permits corporations to conduct remote and virtual meetings. Maryland's nonprofit statute defines quorum as “present in person or by proxy” but appears silent on remote and virtual meetings in general. However, a close reading of the in-person quorum laws indicates that they only apply to rescheduled meetings when a quorum was not obtained at an initial meeting and are silent on the question of determining quorum for a regular meeting. I'm sure you're all fascinated by these details.

Therefore, I and the MLA Bylaws Committee recommend that MLA follow the procedures outlined on MLANET for counting a quorum. A link to these procedures was distributed on May 12 to members via email, and they are posted on MLANET at the web address displayed on your screen.

It is the opinion of the Bylaws Committee and the parliamentarian—that's me—that following these procedures complies with the MLA Bylaws.

Julia Esparza: Thank you, Chris. Unless there are objections, we will follow these procedures.

I'm pleased to introduce Linné Girouard, AHIP, MLA's sergeant-at-arms, who will assist us with the counting of the quorum.

I now call our meeting to order.

According to the MLA Bylaws, a quorum of 200 of the voting members is required for transaction of business. Sergeant-at-arms, what is the count?

Linné Girouard, AHIP: Madame President, the count is 499.

Julia Esparza: There being more than 200 voting members present, we have a quorum.

Chris will continue walking us through our next order of business: adopting Special Rules of Order that will support how we introduce, discuss, and vote on new business. Chris?

Chris Shaffer: So, Robert's Rules allow for electronic meetings as long as there are conditions of opportunity for simultaneous oral communication among all participating members equivalent to those of meetings held in one room or area. It is my opinion that MLA's plan for the use of WebEx is in compliance, and the Bylaws Committee concurs. Robert's Rules says it “may be advisable” for organizations to establish additional rules relating to electronic meetings. The authors of Robert's Rules have published Sample Rules for Electronic Meetings that are expected to be part of the twelfth edition, due out in September 2020, and all of us parliamentarians will eagerly rush to buy a new copy.

The Bylaws Committee and parliamentarian recommend adopting the proposed Special Rules of Order. A link to these rules was distributed on May 12 to members via email, and the rules have been posted on MLANET at the address on your screen. The rules address issues such as interrupting a member, motions submitted in writing, and so forth, as you can see on your screen.

Given that proper notice of these rules has been sent to MLA members, the Bylaws Committee moves that MLA adopt the Special Rules of Order. These rules will be in effect in perpetuity but can be revised, suspended, or eliminated by a two-thirds vote at any future annual meeting.

Julia Esparza: Thank you, Chris. It has been moved that the Special Rules of Order as they appear on MLANET be adopted. The motion requires a vote in favor by two-thirds of voting members present at the meeting and remains in effect in perpetuity. It can be revised, suspended, or eliminated by a two-thirds vote at any future annual meeting.

I propose to approve the motion by unanimous consent. Any member may object, in which case we will call for discussion and vote in the normal manner. Does anyone object? The poll is going to be open for two minutes each time we have one open.

[Two minutes of silence.]

Sergeant-at-arms, were there any objections?

Linné Girouard: Madame President, no objections.

Julia Esparza: There being no objections, the motion is approved, and the Special Rules of Order are adopted. Thank you, Chris, for all your work.

Gurpreet Kaur Rana, MLA's secretary, will move to adopt the agenda for the 2020 business meeting.

[Two-minute pause.]

Julia Esparza: Sergeant-at-arms, what is the vote?

Linné Girouard: Madame President, no objections.

Julia Esparza: Thank you. There being no objections, the motion is approved, and the agenda is adopted. Thank you, Chris, Linné, and Gurpreet. Please stick around as we may need to call on you.

Kevin Baliozian, executive director, will now introduce MLA's Board of Directors.

Kevin Baliozian: Thank you, Julia. It's my pleasure to introduce the members of the 2019/20 Board of Directors:* President: Julia Esparza, AHIP

- President-Elect: Lisa K. Traditi, AHIP
- Immediate Past President: Beverly Murphy, AHIP, FMLA
- Treasurer: Shannon D. Jones, AHIP
- Secretary: Gurpreet Kaur Rana
- Chapter Council Chair: Donna R. Berryman, AHIP
- Section Council Chair, now Communities Council Chair: Elizabeth R. Lorbeer, AHIP
- Directors at Large:
  - Marie T. Ascher
  - Stephanie Fulton, AHIP
  - Sally Gore
  - Sandra Irene Martin, AHIP
  - Meredith I. Solomon, AHIP

Please congratulate your 2019/20 Board of Directors. I can hear you clapping from here!

Julia Esparza: Thank you, Kevin. Now it is my honor to introduce, Shannon D. Jones, AHIP, MLA's treasurer, who will now present the treasurer's report.

Shannon D. Jones, AHIP: It's my pleasure to present to you today, MLA colleagues, as your treasurer—I have the distinct pleasure as serving as the treasurer for the past year, and it's been an exciting ride, so I'm happy to share some of the highlights.

As your treasurer, I share the financial stewardship of our association with the executive director, Kevin Baliozian, and rely on the insights and review of the Finance Committee to ensure that the Board of Directors can exercise its duty of care.

Our work includes reviewing the budgets and financials prepared by the MLA staff, working with the MLA independent auditors to ensure compliance and best practice, setting MLA's investment strategy with MLA's independent financial advisor, examining key MLA pricing models, and analyzing contract terms with MLA's management company, MCI USA.

As you can imagine, we have been quite busy this year with the financial consequences of COVID-19. I am so grateful to have been supported in my role as treasurer by an experienced group of colleagues. And so, I am especially grateful to Beverly Murphy, Sandra Martin, and Barbara A. Epstein, AHIP, FMLA. These are three librarians I look up to tremendously, and to have their support this year has been excellent. I hate to see them roll off the committee, but they've done the association an excellent job. I also want to thank Kevin Baliozian. He didn't put his name on the slide, but I want to thank Kevin for all the hard work that he has done, and Kelly Weaver from MCI for providing so much expert advice and guidance for us.

So, let's talk about 2019 and a review of that year before we move onto 2020. The year 2019 was one of transition and continued investment, and we did a pretty good job. We expected, and budgeted for, an operational deficit. We transitioned to an association management model and expected transition costs as a result. That decision was motivated by the objective to reduce longer-term core costs, tap into new expertise, and scale up our investments in innovation. MCI USA, our management company, has been an outstanding partner through this crisis, with fast pivots in remote working, innovation in the annual meeting, and protection of MLA's interests in the many contract negotiations that we had to work through this year.

We have continued to invest in new education offerings. This year, we are introducing twelve new self-paced courses.

We did very well in our investment portfolio, until COVID-19 came about.

So, 2019 was also a year of transition for MLA communities. What I had the pleasure of doing was meeting with the former section treasurers on a monthly basis just to dialogue about how we're going to fund activities, scholarships, and awards moving forward in the new format. So, at the end of 2019, $250,000 of accumulated section funds went into MLA's endowment account so that we could sustain the funding of scholarships and awards over time.

Now, we're going to move to talking about what pays for what. And so, this chart here shows you, essentially, how things get paid and how we use the money we collect to pay for things. So, the first category talks about our contributors, our membership—us, our annual meeting revenues, and then vendor advertisement, job postings, and those types of things.

In the middle column, you see our break-even column. These are things we get external funding for, so to speak, and then we use that funding to pay for particular programs.

The cost centers are things that all of us, as MLA members, value. Those are the things that allow us to have a value-added experience at MLA. So, we use the contributions from membership, the annual meeting, and the vendor advertising to pay for work in the caucuses, the chapters, and the committees. So, that's just a little overview of how the money is dispersed.

Let's go on to the next slide. Just to show you where our revenues come from, we want to be as transparent as possible, and I want to be very clear with you all. I am not a paid MLA staff member; I am a volunteer member, just like you all. And so, when we hear that membership costs are too high, the registration rates are too high, education should be free, MLA shouldn't depend on vendors, I have said those very same things myself. But now that I serve in this role, I know that achieving those things for our membership takes some work. But we wanted you to see what the breakdown looks like.

So, when we say we shouldn't rely on vendors, you see that 36% of our revenues come from vendors, the meeting registration, the membership. We're showing this to you to say that MLA is not a major corporation like Walmart, Target, or Google, which are making money. This is what we have. Everything that we take into the association, we invest it back into MLA.

And MLA is us. Each one of us gets some value-added experience or service from MLA. So, everything we take in, we give right back to either making our association better or trying to help you be able to fund professional development, come to the conference, and so on.

So, please note: We hear you.

Also, keep in mind that those 4 revenue streams make up 99% of our MLA revenues, so, we won't exist if we don't have these things.

MLA strives to deliver value. Value costs money. We do realize that you all have tight budgets and that some of you operate under financial hardship. This is why we are steadily working to increase the amount of financial support that MLA provides through scholarships and grants. And the vendors help with those things, too. This is really for those people who think vendors may have some influence. The *JMLA* and *MLAConnect* editors operate with editorial independence. So do our National Program Committees, our curriculum committees, our Books Panel, our Joint MLA/Association of Academic Health Sciences Libraries (AAHSL) Legislative Task Force, and our caucuses. Though all those entities have editorial independence, they aren't influenced, necessarily, by the vendors.

Now, let's look at 2020. So, we were on track to have a banner year at MLA when it comes to our finances. But we all know what happened: COVID-19 happened. And so, before COVID-19 hit, we were on track to meet the budget, with revenues set to increase by 25%. Now, we are looking at significant financial uncertainty, with operational losses ranging between meeting net revenue budget at break-even and losing up to $900,000. That's a huge range of uncertainty, in addition to over $500,000 of investment losses.

The good news is that MLA is financially strong and equipped to weather this storm. We have over $1.8 million in our association reserve fund, which is separate from our endowment. It can withstand even the most pessimistic $900,000 loss. And that's assuming that we had no virtual conference. Even if we had a $250,000 to $500,000 operational loss, it would be a major success because we can withstand that. It wouldn't be good, but we can withstand it, and our association will continue on.

So, we're on track with our transformation and our investment in education. Those are essential to the long-term relevance and sustainability of the association.

Finally, I know that people have questions about the finances, and we want to give you an opportunity to ask those questions. And so, we just added a Finances open forum. Please plan to attend. It's going to be Wednesday, June 24, from 1:00 to 2:00 p.m., central. We want you to come and ask us questions so that we can demystify the MLA finances for you and help answer questions for you that you may have wondered about, or you want to know why MLA can't do a certain thing this way, or why do we have to pay for this. We want to answer those questions for you, because, moving forward, we want to be as transparent as possible when it comes to the finances, because, again, this is our MLA, yours and mine.

So, thank you for allowing me to present to you, and I'm going to turn it back over to Kevin.

Julia Esparza: Thank you, Shannon. The next order of business is the executive director's report. Kevin, please present your report.

Kevin Baliozian: Thank you, Shannon. Thank you, Julia. Shannon, thank you for sharing the general financial context we are navigating. It's an interesting time. Janna, thank you as well for presenting highlights of the MLA '20 vConference and Exhibits. I know that the NPC, the MLA staff, and our technology providers are working really hard and fast to design and execute what we intend to be an outstanding online experience. The details are forthcoming. We've already put quite a bit up on the MLANET page for the annual meeting and have a few more weeks before we can detail all this up.

So, let me give you a highlight. This is not a Zoom call with breakouts or simple live streaming. This is a comprehensive, exciting, interactive experience between presenters, attendees, and vendors that will be supported by the latest technology provided by MLA's long-term partner, Cadmium. In this chart, you'll see basically three phases.

There's a preparation phase, which is actually happening now but will involve a lot of the presenters and exhibitors in June to, say, mid-July. That is going to include voice synced to slides to uploaded videos, and vendors will be creating their online exhibits. We expect this phase to take up maybe up to sixty days, but probably a little less.

Phase 2 is the middle one *[as shown on chart on screen]*. The meeting is open. It will likely start around August 1, perhaps a week earlier. We want to give you two or three weeks minimum before the actual live experience, which is the last block. So, this middle stage is going to be an on-demand experience. Basically, it's asynchronous. You look at things that you want to look at on your time schedule, whether it's papers, posters, lightning talks, or exhibits, and whatever they have to offer.

The fun starts in the live experience, which will be scheduled over a number of days. We're working out everything that needs to be live right now, because that will include the immersion sessions, the keynotes, the live vendor symposia. It's also an opportunity for you to meet one-on-one through video chats with your representatives at the different exhibits. The idea is that you will have figured out what you want your experience to be during the weeks prior and will come in at set times to participate in interactions at the time that whatever you're interested in is happening.

So, we'll let you know when those things will happen as we nail down the details.

As usual, the entire meeting materials, whether it's the prerecorded things or it's the live events, will be available post meeting. In fact, as a member, you can see those for multiple years. As registered attendees, you will be able to see them immediately.

We know that a number of you need to change your registration. We know that some of you also need to register. We've also introduced institutional registrations and volume pricing so that the MLA '20 vConference is available to a broader audience. We're entirely retooling the way, back end, we were doing this, and we're aiming for June 1 to be able to have you do those changes and those new registrations. In the meantime, go to the meeting page. It's listed at the bottom here *[chart on screen]* for the latest developments, which we will also communicate through *MLAConnect* and through regular emails.

Moving on to our education offerings: As Julia mentioned, this is an exciting year. We already had added quite a few offerings a number of years ago and continuously through a number of committees that were formed in the last couple of years, this is part of MLA's strategic initiative. It involves seven committees and dozens of instructors, instructional designers, grant funding for certain pieces of it, and a measurable investment by MLA in creating curriculums.

To make it easy for you to access online continuing education (CE), we are going to be piloting the MLA CE Passport. For a single price, you will be able to access all of the online CE that is available right now and will be made continuously available through the end of this year. So, things coming online are the webinars from May through December of this year. Also, the self-paced courses—there are four online right now, though we expect eight, perhaps more, actually—will be available as well. Those will be available through the end of the year as part of the CE Passport. There will be a couple months to actually complete those courses, so December 2 is not a firm, drop-dead date in terms of when you have to finish the course. But there will be more detail on that.

In total, we are talking about more than 60 courses available for a price that we've actually published on the meeting page, and there will be more information on that. The base price for members is $375. We are also making no-charge pricing available for furloughed and unemployed members. There will be some parameters around that and details forthcoming. There's volume pricing, and there will be other types of lowered pricing that will be detailed in the next few weeks.

Make sure you join the open forum on Education for a closer look at the new self-paced courses. I know that they intend to actually do a little live demonstration, also, for a deep dive in MLA's education strategy. Also, get your questions ready as well. And feel free at any time to contact the chairs, all the members of all the curriculum committees. They're all listed on the website. You should feel free to ask any question at any time by going straight to the source—in this case, your colleagues on the curriculum committees.

As Julia mentioned, we are launching EFTS. In fact, it's quite busy here in getting all this going. We announced last week the rollout of EFTS, and it's real. It's becoming totally real. We are working through all of the admin right now, signing up libraries. Seven hundred and forty have signed up; 82 in process. We had a goal of 750, so we're just about there. There are a number of folks who are just working things out with their legal departments. COVID-19 hasn't helped; it's slowed things down. A number of EFTS are under furlough. There have already been changes. So, it's a pretty scary situation for a number of libraries, and it's being reflected in EFTS.

The EFTS program is a complete redesign of the software. It was a platform that was introduced by University of Connecticut Health Center (UConn) twenty-five years ago. This was before we had heard of Venmo and PayPal. This was peer-to-peer money exchanges before any of those things happened. It's absolutely phenomenal how that was set up twenty-five years ago.

Today, we're building the latest security features for access and financial flows to get up to the security you expect. We are including application program interfaces (APIs) with DOCLINE, and the National Library of Medicine (NLM) has been great in its collaboration in making this happen. So, we will be connecting with DOCLINE only by these, on group information consortia, on interlibrary loan transactions—all in a totally confidential way and for the sole purpose of running the platform and bringing the information back to you.

Most of the operations, other than the ones with high security and control, will be self-service, and there will be internal controls when money is dispersed to avoid any phishing attempts and other types of illegal activities that could happen if someone got hold of your password.

We expect the user experience to be significantly increased and the administration time to run the platform to be significantly reduced. That's critical, because the volume has gone down, and we agreed to take this on if we could actually do those two things: improve the experience and spend less time in administration.

So, on the screen, you see the rollout, the highlights there. June 1, you start logging in and configuring things. The EFTS system at UConn is going to go offline in terms of processing transactions after May 31 transactions, so if you are not signed up on the new system and you are using EFTS, you will no longer be able to process any transaction past May 31, for anyone. This is not just people who transition. That system will go offline. It will stay online for a few months for people to see their balances, for people to settle their balances, and for the purpose of taking money in and out and closing the accounts. But the June transactions will be processed on the MLA EFTS starting July 15. So, there will be forty-five days in which nothing will be processed. That's typically ten days more than would be normal, but that allows us to work with you to do all the checks that everything is going to be okay. So, exciting, and cross our fingers that there won't be too many glitches. There will be some, but not too many.

This is my sixth year serving as your executive director. And as a tradition—now I guess I can talk about tradition—in all of my dialogues with you, I presented a few statistics. So here, I've just condensed them to a few. And they are around how engaged you are at MLA.

The percentage of you belonging to caucuses is at 69%. You have joined 4 caucuses on average. That used to be 2. So, people are participating in twice as many caucuses as they have in the past, which is great. That was the whole idea: to be more inclusive and diverse in caucus participation, so that's great.

Thirty-seven percent of you are members of the Academy of Health Information Professionals (AHIP), so that's also impressive. Now, you know, the sky's the limit. It should be 100%, right? But 37% is impressive.

Thirty-five percent attend the physical meeting. And this is our challenge here. How do we actually, in a virtual conference setting, make that number higher? Can we get it up to 50%? Can we actually have a lot of nonmembers attend the annual meeting now given volume pricing and those types of things and associations? Is this one of the solutions to greater attendance and greater inclusion at the annual meeting? We need your help, and we'll find out.

Twenty percent of you actually volunteer on a committee, and that's not counting chapters. This is phenomenal. The standard in associations is 5%, so 20% is huge. That's 1 out of 5 who currently have an active role on a committee. And some of you have taken a break, and you're ready to sign up for the next year. But this is phenomenal engagement, so that's wonderful.

And lastly, Shannon mentioned our increase in travel grants. We're up to $200,000 in various ways that we're supporting people joining. That has more than doubled in the last 2 years. And again, the sky's the limit there. We're not quite ready yet to announce the total amount of grants and scholarships for the MLA '20 vConference and the CE Passport. We're working with vendors to commit to sponsor that. But that is forthcoming because we obviously are all aiming to be able to have a meaningful envelope to support those who can't afford the $350 fee, for example, and those who have lost support from their institution for attendance.

So, more to come on that.

That's it for me, so back to you, Julia.

Julia Esparza: Thank you, Kevin. The next order of business is the 2019/20 Annual Report. In the interest of time, we will receive the annual reports in a block. The information reports of the appointed officials, the councils, the committees, the task forces, the representatives, the sections, and the chapters are found in the 2019/20 MLA Annual Report. These reports are posted on MLANET and are available to all members. A paper copy may also be requested from the executive director's office. Please take time to read through the executive summaries of these reports for an overview of the impressive work performed by your peers. The summaries are considerably shorter than the full reports.

Are there any corrections, amendments, or questions from members regarding the content or meaning of any of these reports? If so, please raise your hand using the WebEx option so you and your institution may be recognized.

[Pause.]

Debra Cavanaugh: Julia, we do have a hand raised.

Julia Esparza: All right, I don't see the name. Can you please ask for them to provide their name?

Debra Cavanaugh: Oh, looks like they may have lowered their hand now.

Julia Esparza: Okay, possible error. If there is no objection, these reports will be filed as presented or amended.

Those who are nominated each year as potential members of the MLA Board of Directors are selected by virtue of their experience and reputation to serve the association. But few can imagine beforehand the level of commitment that election to the board requires, especially this year. Directors who have completed their term on the MLA Board have served our association with enthusiasm, dedication, and perseverance. The association and the Board of Directors express our appreciation and recognize each of you today for the extraordinary work and leadership you have provided during your term of service. Thank you for a job well done.

- Marie T. Ascher
- Stephanie Fulton, AHIP
- Elizabeth R. Lorbeer, AHIP
- Sandra Irene Martin, AHIP

Recognition of past president: I would like to express my sincere gratitude to Beverly Murphy, AHIP, FMLA, MLA's 2018/19 president. Beverly, where are you? Please join me on the screen.

Beverly, as the first African American president, you already had a special place in MLA history. However, your leadership was fundamental in helping MLA become more versatile and responsive through the communities transition to make sure that diverse voices were heard, that MLA is focused on inclusion, and for us to learn to “Relax, Relate, and Release.” For many of us, that phrase has been especially invaluable over the last months. Your passion for MLA will continue with your “I Am MLA” campaign. We will never be able to thank you enough for your dedication to our organization, but I hope this crystal gavel (check your mailbox) will remind you that your hard work is deeply appreciated by every member of MLA. As MLA president, you have been an inspiration, a great advisor, and confidante. I will miss you next year and regret that I can't share my gratitude in person. Thank you for all you have done for MLA and the profession.

Beverly Murphy, AHIP, FMLA: Thank you, Julia. I hope everyone can hear me. Hello, everyone. Being in this role as the immediate past president is bittersweet. I get to look back over the years and see all that we have accomplished and how far we've all come in our transformation to elevate, but at the same time, I'm saying goodbye to some things while saying hello to new things, and one which I'll talk about in a minute.

One thing is for sure: the historical significance of my role can never be diminished, and the same stands for my love of all of you. You are the sunshine of my life and that's why I'll always be around. So just remember to always keep an open heart and an open mind, and everything will be just fine. Thank you all for the opportunity to serve and do well, I hope. I love you all.

And now, to the next slide. The last time we had a conversation, I told you that I would be looking forward to hearing your stories, and now's the time. Hopefully in June, we will be launching the I Am MLA campaign, which grew out of the need for all of us to gravitate toward the collective understanding that we are MLA, and that it is up to us as members and volunteers, to do what needs to be done for our association. This starts with each unique individual and culminates in a shared vision.

This campaign will serve as a networking portal and membership recruitment and retention tool, and a marketing and promotional venue for our members, our association, and beyond. So, when you hear the call, we, the I Am MLA ambassadors you see on the screen right now, I hope that you will engage with this vision by becoming an I Am MLA ambassador yourself, and join the mosaic of member profiles that we are curating. We are, indeed, stronger together. So, hopefully you'll say this with me, point to yourself, I am MLA, you are MLA, and we are MLA. Stronger together.

Thank you so much.

Julia Esparza: Thank you, Beverly.

The MLA 2020 election was conducted from January 15 to February 19, 2020. Voting statistics can be seen on your screen. Election results were announced on March 12, 2020, in *MLAConnect*. The following are the election results:

Nominating Committee

- Kelsa Bartley
- Antonio P. DeRosa, AHIP
- Patricia Devine
- Xan Y. Goodman, AHIP
- Andy Hickner
- Lisa A. Marks, AHIP
- Tamara M. Nelson, AHIP
- Hannah Rutledge, AHIP
- Angela Spencer, AHIP

Kristine M. Alpi, AHIP was elected to serve as president-elect.

Heather N. Holmes, AHIP, Brenda M. Linares, AHIP, and James Dale Prince, AHIP, were each elected by the membership for a three-year term to the MLA Board of Directors.

Adela V. Justice, AHIP, was elected by the Community Council to serve a three-year term as Community Council chair and will serve on the Board of Directors in that role.

Now, it's time for my year as MLA president to come to a close.

It is my honor and pleasure to introduce your 2020/21 President Lisa K. Traditi, AHIP. Lisa, associate professor and deputy director, was a hospital librarian for nine years in the Denver metro area before joining the faculty of the University of Colorado Health Sciences Library in 1996. Lisa was two times past chair of the Midcontinental Chapter of MLA and an advisory board member for the University of Denver School of Library and Information Science.

Lisa received her master's in library science from the University of Arizona and is a Distinguished Member of MLA's Academy of Health Information Professionals.

In addition, Lisa is the local co-coordinator for “Supporting Clinical Care: An Institute in Evidence-Based Practice for Medical Librarians,” a highly, highly coveted educational workshop. She has contributed chapters to *Evidence-Based Practice: An Implementation Guide for Healthcare Organizations,* published in 2011; the *Medical Library Association Guide to Managing Health Care Libraries,* published in 2011; and *Nursing Research Secrets,* published in 2003.

She is the recipient of the Colorado Council of Medical Librarians' Marla Graber Award and the Midcontinental Chapter of MLA's Bernice Hetzner Award.

As an avid camper, she spends almost every weekend from May through September somewhere in the Rocky Mountains with her handsome dog, Amos, and her even more handsome husband, Frank.

I am thrilled to pass the gavel on to my colleague and friend, Lisa K. Traditi, AHIP.

Lisa K. Traditi, AHIP: Thank you for my gavel, and thank you, Julia. It is a pleasure to be here, both personally and professionally, to thank you for your incredible leadership during your presidential year. I'll say more during my inaugural address, but for now, I'm happy to send you a silver cup (watch your mailbox) as our token of appreciation for all you've accomplished this year. I hope you will display this cup proudly, because it symbolizes a year when MLA broadened its opportunities to build our future under your leadership.

Julia Esparza: Thank you, all, very much.

Lisa Traditi: May I present the members of MLA's 2020/21 Board of Directors.* Lisa K. Traditi, AHIP, President

- Kristine M. Alpi, AHIP, President-Elect
- Julia Esparza, AHIP, Immediate Past President
- Donna R. Berryman, AHIP, Chapter Council Chair
- Sally Gore, Member
- Health N. Holmes, AHIP, Member
- Shannon D. Jones, AHIP, Treasurer
- Adela V. Justice, AHIP, Community Council Chair
- Brenda M. Linares, AHIP, Member
- James Dale Prince, AHIP, Member
- Gurpreet Kaur Rana, Secretary
- Meredith I. Solomon, AHIP, Member
- Kevin Baliozian, Executive Director

I'm looking so forward to working with all of you.

We have no resolutions at this time and will move on to new business.

Do we have any new business to bring before the assembly? Please use the “Raise Hands” feature in WebEx to submit new business.

[Pause.]

We have no new business at this time.

I recognize Gurpreet Rana, secretary of the MLA Board of Directors.

Gurpreet Rana: Thank you, Madame President. I move that we adjourn.

Lisa Traditi: Okay, it has been moved to adjourn, but please stick around after the vote. You'll get more of me.

I propose to approve the motion by unanimous consent. Any member may object, in which case we will call for discussion and vote in the normal manner. Does anyone object?

Linné Girouard: Madame President, there is no objection.

Lisa Traditi: There being no objection, the motion is carried, and the 120th Annual Meeting of the Medical Library Association is adjourned.

[*End of business meeting*.]

## INCOMING PRESIDENTIAL INAUGURAL ADDRESS

### Tuesday, May 19, 2020

The MLA 2019/20 Incoming Presidential Inaugural Address was held prior to the MLA '20 vConference and virtually due to the COVID-19 pandemic.

Lisa Traditi: Thank you all, and thank you to Julia Esparza for your leadership and friendship and the spreadsheets and attention to data and details.

Last year, in your inaugural address, you used a roll of pennies to show how a group of individuals can band together to transform into something more powerful and valuable. Your tenure as president included challenges no one could have foretold. Not only did you face those challenges with determination and grace, you helped the association reach new goals. MLA is stronger because of you.

As I follow in your footsteps and those of the trailblazer Beverly Murphy, I can't help but wonder. How did I get here? The Talking Heads are still one of my favorite bands. Those of you who know me well may be thinking, “My god, what have we done?” But that's for a different talk.

How *did* I get here? I remember my very first MLA annual meeting: Boston, 1989. I earned my master's of library science at the end of 1984 and bounced around different types of libraries until finding my home as a hospital librarian. After three years as a one-person hospital librarian, I finally got the hospital to give me money to attend my first MLA meeting. Eloise C. Foster, AHIP, FMLA, was the president. I'll be honest, I don't remember her speech or that of incoming president Frances Groen, FMLA.

So even though giving this talk is stressful for me, I'm under no illusions that it will go down in history as a special event to any of you.

What I do remember—at that meeting and for many after that—is thinking, I'll never be on that stage as an officer of the association or as president. I imagine some of you are thinking that right now. Well, as my mom has always said, “Make your words sweet, 'cause you never know when you'll have to eat them.” I have spent my career eating my words!

“I'll never be a librarian,” I said, when my mom suggested it as a career to me when I was in high school.

“I'll never work in an academic library.” Now I've worked in one now for over twenty-three years.

“I'll never teach.” Then, I spent a few decades as an education librarian.

“I'll never be an administrator.” Here, I sit in my deputy director office.

I'll never…I'll never…I still haven't learned that it's often a predictor of my next challenge and learning opportunity.

But it's less about how I got here as it is why. Why did I end up eating those words? Why did I get here?

Literally, from week one as a hospital librarian, the health sciences librarians in the Denver community—people like Marla Graber, Sara Katsh, AHIP, Margaret Bandy, AHIP, FMLA, and Rosalind Farnam Dudden, AHIP, FMLA—reached out to me to offer their help, advice, and friendship. When I joined my local, state, and regional library associations, my network grew, and the help kept flowing in.

So many of you have given me advice, opportunities, drinks, and even jobs throughout my career. At first, I started volunteering just because someone asked me to do so. I was surprised and honored to be asked. And, to tell the truth, it made me feel good to give back—to help my professional organizations. I hope I've been of use.

I do know that when I've been in the position to ask—as chair of my state organization and/or my chapter—I really needed people to say yes. It takes an amazing number of people to keep this association, as well as our chapters and local organizations, running. Any of you serving on committees or task forces as officers or in appointed roles know this all too well. Your time and work are needed and appreciated.

But now I see that I have received more than I've given. Each time I did that new thing, I was scared, but also excited by the challenge and chance to build new skills and develop my organizational muscles.

I've learned how to negotiate to get things done with people over whom I really have no authority. I've learned to develop, act on, and deliver strategic plans. I've learned how to plan professional meetings, so many meetings, for anywhere from 25 to 600 people. (I'll stop for a shout-out here to Jon Crossno, AHIP, and the Quint Chapter Meeting planners.) I've learned to balance budgets and time. I've learned that librarians are the most awesome people I've had the pleasure to know, and we're a force to reckon with once we get behind a good idea.

It wasn't magic. It didn't just happen. Someone put my name forward. Someone asked me to serve. It started with sponsorship. A colleague advocated for me, and I said yes to the opportunity. Thank you to all of you who have already said yes.

Volunteer to serve: For the first time in a long time, I didn't have enough people to fill all our jury spots. Never fear, you'll have an opportunity to put your name in for juries coming soon. Watch *MLAConnect* for a volunteer form.

Take a risk. Scare yourself. Say “yes” to leadership opportunities. If you don't get the spot you want the first time, keep saying yes. I'm going to tell you a secret: I ran for the MLA Board of Directors *three* times before I was elected. By the third time, I told the Nominating Committee, “Sure, I'll fill out your roster, but you know I'm not going to win.” And, of course, that's when I was elected.

By the way, if you are a mentor or a boss, sponsor someone coming up in libraries for a role in MLA. Put their name in the hat for opportunities, and then make sure you help them make time for the commitment.

Our association needs you. As Beverly's brilliant campaign says, I Am MLA, You Are MLA, We're All MLA. We can't get where we need to go without you. We need to help each other face the future. Who knows what it will hold?

Many have tried to predict the future of libraries. Even back when I was in library school in the early 1980s, we discussed if libraries would still be relevant or even exist in twenty years. Well, it's been a lot more than twenty years, and we're still here—different, but here.

In 2005, only fifteen years ago, Donald A. B. Lindberg and Betsy L. Humphreys, FMLA, proposed an “evolutionary scenario for the medical libraries of 2015.” They did remarkably well, even five years past their proposed date, and I encourage you to go check out what they wrote. Lots of the things are still true today.

What they didn't predict and what I bet no one has suggested in any of those “What will libraries be like in twenty years” articles: global pandemic. It's almost become cliché to say that we are in unprecedented times. My husband has noted how often we've heard the phrase “existential crisis” in daily news reports. In libraries, we can relate to being in existential crisis. Many of our libraries have lost space or their entire buildings in the past years. Are we still librarians without a physical space?

Jeff D. Williams and Neil Rambo, in the article, “It's the End of the World and We Feel Fine,” described the destruction of the New York University (NYU) Health Sciences Library by Superstorm Sandy in 2012 and encouraged libraries to undergo a thought experiment:

Imagine that you awake tomorrow morning and your library is gone, not in a year, not damaged, but gone. What would you do? What would you replace? Can you reimagine what your library should be and start in that direction instead of recreating what was? What does the plan look like that would take you there?

I challenge all of us to start planning for the future with fresh eyes. What would *we* do if our association was gone? Would we rebuild? Move to a different association and take it over? Happily, we can continue the work started under Past President Teresa L. Knott, AHIP, FMLA, when we started our communities transformation from sections and special interest groups (SIGs) to caucuses. Caucus membership is higher than expected, as you heard earlier. More of us are now engaged than ever.

As Lucretia W. McClure wrote in her introduction to the *Journal of the Medical Library Association (JMLA)* special issue on “New Roles for Health Sciences Librarians,” “Hope was never the issue; keeping pace was the challenge.” We as an association have to continue to keep pace, working together to drive the changes we need to make in order to meet the future, to be there waiting.

How will we get there, where we want to be? Keeping pace is still the challenge.

In her 2012 *JMLA* article called “Evolution, Revolution, or Obsolescence: An Examination of Writings on the Future of Health Sciences Libraries,” Julie J. McGowan, AHIP, FMLA, reviewed the peer-reviewed literature and social media, looking for writings on our future. She concluded:

There is general agreement that health sciences libraries must change to ensure their continued viability. It's up to libraries whether that change will come through evolution or revolution or whether complacency will mean forced obsolescence.

In her 2014 *JMLA* article, “Creating the Future, Jane Blumenthal, AHIP, FMLA, wrote:

The future is not neat and orderly. And it is not waiting for us. We cannot find the future because it does not exist. It will not exist until we create it. The question we need to wrestle with is not “what does the future hold for health sciences libraries,” but rather, “what future are health sciences librarians going to build for themselves?”

So, what *will* we build, for ourselves, for our libraries, and for our association? And what is the most important thing we need to get there? Any time of great uncertainty also brings opportunities for change. I want us not to think about evolution, a process that relies on outside forces to make change. I want us to be more proactive.

Let's train for agility. Agility is the ability to be quick and graceful; the power to move quickly and easily; to be nimble. In sports, agility is the ability to quickly change body position or direction—influenced by balance and coordination. It's the ability to explosively start, decelerate, change direction, and accelerate again quickly while maintaining body control and minimizing a reduction in speed in reaction to what's going on around you. I like that part. To be agile, you're responding to what is going on around you, taking in that information and translating it into a position that will maintain balance and control. But we can't just move fast. We have to stay in control, too.

A couple of years ago, in an email conversation with Gerald J. Perry, AHIP, FMLA, former director of my library and one of my treasured mentors, I shared my thoughts on agility. He agreed that it's important and added:

The choices that need to be made to remain agile and successful must be informed by values that remain constant. Integrity of mission, integrity of values, integrity of commitment to people, integrity of ethics—all these things are essential to success. They make us professional. They sustain the profession and are understood by our clients.

I could not agree more. As an association, we must remain constant to our values and our mission, while treating each other and our partners with respect.

I'm so proud that we have embedded the work of the Diversity and Inclusion Task Force into the language of MLA's mission, vision, and values and into our organization's Code of Ethics. I look forward to appointing the members of the new standing Diversity, Equity, and Inclusion Committee to continue this important and integral work.

As we move forward as an association, planning our first-ever interactive virtual meeting, I want to focus on our ability to quickly respond and even predict changes in our environment before they happen—even better, to direct those changes. How do we develop the capacity in the association for change and the ability to stay current and relevant throughout constant change? How do we become more agile?

I looked at two areas where agility training is used: in sports and dog training. The skills are the same. But since they're fluffier, I chose dogs. Agility training includes:

- Learning basic to advanced skills: It takes practice and guidance by skilled coaches and leaders. Those with more experience can mentor those new to the game.
- Working to increase speed and difficulty and getting better at what we do, while being careful to avoid repetitive motion injuries. And using practice and instructions to correct dysfunctional movements.
- Checking alignment and flexibility to make sure we're not doing things the wrong way and damaging our practice and reputation.
- Building our strength, speed, power, balance, and endurance, so we can stay in it for the long haul.
- We'll use normative data: How do we compare to others? What is the evidence for how we're doing?
- And we'll create individually tailored programs to address specific areas of need where we develop services for specific populations, such as continuing to build our research skills through MLA's popular Research Training Institute, which has been so successful under the leadership of Susan Lessick, AHIP, FMLA.

We're also continuing the work of Jerry Perry and the InSight Initiative Task Force, who are partnering with vendors to identify and examine solutions to vexing scholarly communications and other problems faced by librarians, publishers, and the stakeholders we both serve.

Soon, I'll be appointing MLA members to a new standing MLA InSight Initiative Committee, which will move this terrific effort from a new initiative to being part of MLA's ongoing and strategic work.

And we must remember to keep our heads up while doing all of this in order to analyze the environment and anticipate movements when obstacles appear.

Agility training takes a team ready and willing to work. I'm ready, your Board of Directors is ready, and I hope you are, too. I believe we must build resiliency and agility—for the association's financial health, for our members' economic and employment outlook, and for our ability to change and adapt. I hope you'll work with us to build our resiliency and agility.

[Photo of several dogs.]

By the way, this is not an actual photo of the Board of Directors at work. I mean, we are just as good looking as this group, if maybe a little shaggier.

My promise to you this year is that I'll be working hard to communicate clearly and as often as possible with you. The board will be using *MLAConnect*, as we already do, to share information as soon as we can get it to you.

As Ferris Bueller said, “Life moves pretty fast. I you don't stop and look around once in a while, you could miss it.” So, if we don't get information to you as quickly as we'd like, it's only because we're stopping to look around and make sure we don't miss anything. We want to get things right before sending it out to all of you.

If you want to reach me, my email address is on the slide *[president@mail.mlahq.org]*, or you can find it in my member profile on MLANET. Send your constructive, please, comments and any questions there. If I can't answer your questions, I'll make sure it gets to someone who can.

Now, I have a favor to ask of you. Just like you, we on the board are hanging on as tight as we can. We're trying to keep up with our full-time jobs and home school and take care of our loved ones and work from home and maybe reopen our libraries, and just stay safe—all while doing the work of the association. I ask for your grace as we move into this year—selfishly, for me—but also, and more importantly, for any of us in any kind of volunteer leadership position for MLA, our caucuses, our chapters, in any role. It's already been a challenging year, and it's only May!

We're working hard to connect with all of you and help each other survive and improve as health information professionals and keep the association financially solvent. And we're practicing our agility.

Who knew even six months ago that we'd be making MLA history right now with this very meeting? As we continue to experiment with an all-virtual meeting and virtual open forums and all sorts of other ways to stay connected with you, we will get some things right, and we will likely get some things wrong. We're charting new roads and may have to backtrack, but even if we have to pause to break a new trail, we *will* keep moving forward.

Please know, we're all learning as we work through these times. Let's help each other learn, and along the way, be gentle with each other and with ourselves as we find our way.

We also need to remind each other of our superpowers. I was reminded of this recently when working to set up a space in our library for daily screening of the education-focused employees and students slowly returning to our campus. The librarians made several adjustments to the work flow that seemed intuitive but had not even occurred to our campus partners. We know how to get things organized. I don't think we give ourselves enough credit, and I know our institutions don't see our superpowers, unless we find a way to point them out.

To prepare myself for this day, I watched several *TED Talks* on public speaking. All were great, but one that has stuck with me is by Amy Cuddy, who was part of a team researching how your body language can build your confidence. Her research shows that if you practice a “power pose” for as little as two minutes before going into an important meeting or presentation, it changes your brain waves and helps you radiates confidence.

She recommends going into a private place and striking a superhero pose or victory pose before high stakes meetings. You can see them on the screen—your hands uplifted in a “V” with your face slightly up or hands on hips with a wide stance. The nice thing about online meetings is that no one could see me striking my power pose before this meeting started.

My point is that we all need a little help sometimes, and it's okay to get it from within, from a power pose, or from each other.

When I renewed my MLA membership this year, I took the opportunity to join ALL the caucuses.

So, I've seen you: being superheroes to each other, to the clients and organizations you serve, and to the association.

I see you: whether you've stayed at work or pivoted to working from home.

I see you: creating and sharing search hedges, COVID resource guides, and continuity of operations plans.

I see you: cheering each other on, talking each other off of ledges, and mourning when one of our own is furloughed or laid off.

I see you.

I see the stress and the joy of your lives. I'm living in that stress and joy, too.

I am so proud to work alongside of you, and I cannot wait to see all the ways you continue to show the value of health sciences information professionals to the world. I challenge us all, myself included, to claim our power as health sciences professionals for ourselves, our libraries, and our association.

Practice your superhero pose. Use it to strengthen your voice, and share the brilliant work you do every day. I'll do it, too. And together, we'll pivot to face whatever the future has in store for us.

I have so many people to thank: My current boss, Melissa De Santis, AHIP, and my two previous bosses, Rick B. Forsman, FMLA, and Jerry Perry, for hiring me in the first place, sponsoring me, and giving me time to volunteer for local chapter and MLA opportunities, and for keeping me employed.

My direct reports: Douglas Stehle, Jeff Kuntzman, Nina L. McHale, AHIP, and Yumin Jiang. You are all awesome and brilliant superstars.

All my friends in the association, on the board, and out there in library-land; too many to name, but if you think it's you, it definitely is. All of you have mentored and helped me practice my own agility.

My parents, family, and friends, who love me no matter what.

And, especially, my husband Frank—my partner in adventure, love, and laughter since 1986—who keeps me grounded and pushes me to have more fun and keep facing the future.

Finally, my heartfelt thanks to all of you for the great honor of your trust in selecting me to be president of our Medical Library Association. I'll do my best to be worthy of it.

Thank you.

Thank you, all, for participating in this historic virtual business meeting of the Medical Library Association. Please join us for our upcoming open forums and for the summer MLA '20 vConference.

Thank you.

## LIVE KICK-OFF OPENING SESSION (HELD VIRTUALLY DUE TO THE COVID-19 PANDEMIC)

### Monday, July 27, 2020

Lisa K. Traditi, AHIP: Good morning. I'm Lisa K. Traditi, AHIP, your 2020/21 MLA president and cohost for this MLA '20 vConference and Exhibits opening session.

Julia Esparza, AHIP: And I'm Julia Esparza, AHIP, your 2019/20 MLA president and cohost for today's session. It is really great to be sharing the limelight with you in hosting the kickoff session for MLA's first-ever annual vConference and Exhibits.

Lisa Traditi: Julia, I'm excited as well and thrilled with the energy and enthusiasm of our members, organizers, presenters, MLA staff, and technology partners who've been so committed in preparing this historic meeting. We join in their commitment to making this experience as awesome as we possibly can for you. Thank you, all, for being with us today.

Julia Esparza: Let's welcome all of our meeting participants. Let's also welcome many of you who are joining this opening session and have not yet registered for the meeting. Yes, you can still register, because you do not want to miss out on the keynotes, contributed content, social events, exhibits, and vendor programming. More about the program in a few minutes.

Lisa Traditi: Speaking of vendors, they have been so supportive of this virtual conference. They truly value the partnership with all of you and are an essential part of making this MLA meeting a success. We welcome you as well to this opening session and will be recognizing and thanking you, and celebrating our collaboration later in this program.

Thank you all. You've made MLA a dream come true.

Julia Esparza: Todays agenda: (1) How we got here; (2) MLA '20 experience; (3) recognition of sponsors; (4) MLA InSight Initiative; (5) awards, recognitions, and in memoriam.

Let's get started. Lisa and I will be your emcees for this session. We will invite others to join us as well, so you won't get bored. Here's what's in store.

How we got here: It's been quite a ride from our canceled May meeting in Portland to our first-ever August virtual conference.

MLA '20 experience: Janna C. Lawrence, AHIP, 2020 National Program Committee (NPC) chair, and Melissa De Santis, 2020 NPC cochair, will recognize those who have made this meeting possible. They will provide you with tips on how to best experience the next few weeks.

Recognition of sponsors: Our sponsors deserve medals, so we have some for them. Bronze, silver, gold, and even platinum.

MLA InSight Initiative: Librarians and publishers have collaborated on InSight Initiative projects for three years, and we are ready to share outcomes with you.

Awards, recognitions, and in memoriam: MLA's cherished tradition of presenting this year's awards and recognitions, and honoring and remembering colleagues who have passed away in this last year. We have assembled videos and clips, so we hope that you will find this last segment inspiring.

Lisa Traditi: Some of you may know that MLA has a strategic initiative on annual meeting innovation. For several years, we have been diligently crafting the long-term vision for our annual meeting, looking at attendee experience; diversity, equity, and inclusion; value and cost; audiences; and more. We have surveyed attendees and non-attendees, conducted focus groups, and worked with several program committees on continuous improvement for our meeting. We feel pretty good about our work, and we were ready to wrap it up in May.

And then COVID-19 happened, and the reality of having cancelled the in-person Portland meeting and replacing it with this MLA '20 virtual conference and exhibits. As we all know, in addition to being dreadful and scary, COVID has been an accelerator of change. At MLA, we are embracing this change. We're leading the way in innovation and providing value through virtual gatherings. We are taking this opportunity to achieve our long-term objectives faster and to stay ahead of the curve. We create our own path, and it's no surprise that other library associations look at us as an example to follow.

Julia Esparza: Your personal stories are powerful. Your lives at home and at work have been disrupted. Many of you are keeping the lights on in your physical libraries or are present at points of care. Health care providers depend on the quality of your work and your presence. Researchers rely on your expertise to advance their work. Patients turn to you for reliable information.

Some of you have lost your jobs, been furloughed, or experienced salary reductions. All of us know what it's like to have to do more with less. Our jobs are essential, and we do our work with passion because we believe in our mission to provide better health care by ensuring that the best health information is available for all. Thank you for what you do and for what you believe in.

Lisa Traditi: Julia, I join you in celebrating the spirit of our members and their resiliency through challenges and, at times, adversity. The MLA community shared the horror and outrage being expressed throughout our nation in response to the death of George Floyd and many other victims of police brutality. Twenty-seven MLA caucuses expressed the diversity of the MLA community by publishing their own statements of compassion, anger, and hope, all of which you can view via MLA's home page. We reaffirm our commitment to social justice to working to end racial inequity and systemic racism.

Julia Esparza: Lisa, we had rescheduled the MLA '20 meeting to August in Portland, Oregon. Instead, we are meeting virtually. And the city of Portland is experiencing violence as its citizens affirm their right to express their voice that Black Lives Matter and their demands for police reform. Let's pause to reflect on the extreme tensions that the world is experiencing.

Lisa Traditi: Thank you, Julia. MLA is your organization, and as such, MLA is a mirror of you. What affects you also affects MLA. MLA represents the strength we have when working together. It is during times like today that the power of “we” is so essential, that MLA is so essential. Beverly Murphy, AHIP, FMLA, MLA's 2018/19 president, launched the I Am MLA campaign earlier this year. I Am MLA demonstrates the strength of our community. I Am MLA encourages us to collectively understand that “we” are MLA, and that it is up to us as members and as volunteers to do what needs to be done for our association. This starts with each unique individual and culminates in a shared vision. I encourage you to engage with this vision by becoming an I Am MLA ambassador and joining the mosaic of member profiles on MLANET. We are stronger together.

Julia Esparza: The best-laid plans sometimes fail before they succeed. After all, Walt Disney was fired from the *Kansas City Star* because his editor felt he lacked imagination and had no good ideas. So, we have embraced experimentation and the license to fail, and hopefully succeed.

Lisa Traditi: Julia, are you saying we embrace design thinking? That's totally cool and a modern approach.

Julia Esparza: Well, kind of. More like trial and error. You know, gloat when things go well, and hide when they don't. By hiding, I mean stay away from Twitter.

Lisa Traditi: Seriously, we want your feedback. So much is new here in our design of the content, exhibits, and social interactions. Please be kind and constructive in sharing your feedback with us. We want to make this a fun, educational, productive experience for everyone. This is a good time to mention MLA's new Statement of Appropriate Conduct, which the MLA Board of Directors just approved. It applies to all MLA activities, including conferences, meetings, workshops, online forums, social media, continuing education, and all means of communication relating to MLA activities. It applies to all MLA members, nonmembers, invited guests, speakers, moderators, instructors, exhibitors, staff, and all others who participate in an MLA activity or event.

Our objectives are an open, inclusive, and collaborative environment in the association in all its activities and events and outside the profession; diversity, equity, and inclusion in professional practice; leadership of health sciences libraries and information professionals; advancement and support of accessibility for all stakeholder groups; and irreproachable ethical standards that call for health sciences librarians to conduct all professional relationships with courtesy and respect. Please read the statement. It includes the do's and don't's and how to report inappropriate behavior and the resolution process.

Julia Esparza: Thank you, Lisa. Let's all work together to make this virtual experience an engaging and pleasant one for all of us. Lisa, we were talking about MLA's license to experiment and fail. Let's talk about the MLA innovation we introduced from May to July. MLA launched its vConnections series. Sessions were free and open to MLA members and to the public. Feedback from attendees was excellent, and the attendance was higher than it typically is with in-person events. MLA hosted nine virtual COVID conversations, with attendance reaching up to 220 participants. These weekly conversations were designed to help members address professional and personal pain points related to the current crisis. Members shared resources, tips, and tools for adapting to the new normal, in a format that combined video feed with a facilitator and online chat prior and post to the live event. Seven hundred and eighty-eight individuals so far have joined the Slack channel for chats.

In May, we held our first-ever virtual business meeting. Typically, about 225 members attend an in-person business meeting. This year, more than 500 members participated, many for the very first time. We are not going back to in-person business meetings. Why would we?

The Chapter Council and Community Council also held their annual meetings online. Instead of one open forum session at the annual meeting, typically on two themes, we held seven of them between May and July. Themes included strategic initiatives, education, diversity and inclusion, annual meeting innovation, community transformation, MLA finances, publications, and Rising Stars. More of you attended the live discussion; others watched the recording. We estimate that, overall, several hundred additional members learned about MLA initiatives and engaged with leaders than would normally engage in person.

Lisa Traditi: Thank you, Julia. I, myself, enjoyed the experience and was delighted to see so many new members participate and engage. A few minutes ago, I shared with you that we are taking this opportunity to achieve our long-term objectives faster. Here are a few positive outcomes we can already appreciate.

Community strength: The conversations and general engagement of the events Julia described build on the significant engagement of members and MLA caucuses. The COVID-19 resource pages, accessible from MLANET's home page, is an illustration of the quality and depth of collaboration in MLA communities. I invite you to explore this resource, if you haven't already.

Participation: The evidence is there, and we do believe in evidence. More of you are participating and engaging.

Diversity, equity, and inclusion: You participated in vConnections in the way that worked for you and at no cost. You also participated in more caucuses, also at no additional cost to your MLA membership. That spurs broader engagement by removing barriers and providing a welcome environment.

Year-round programming: We've taken activities packed into three or four days of an annual conference and scheduled them instead over three months, with more of them. That seems to work better for most of you.

Julia Esparza: And while you were enjoying vConnections, Lisa, the NPC for this year's meeting was working with MLA staff and technology partners to redesign this year's annual conference and exhibits. When COVID gave them lemons, they made lemonade. Here to talk to you about the exciting virtual conference you're about to experience, please welcome your chair and cochair of the 2020 NPC, Janna Lawrence and Melissa De Santis.

Janna C. Lawrence, AHIP: Thank you, Julia. I'm Janna Lawrence, your 2020 NPC chair.

Melissa De Santis, AHIP: And I'm Melissa De Santis, your 2020 NPC cochair.

Janna Lawrence: On behalf of the entire National Program Committee, welcome to “2020 Vision: The Future in Focus.” What a theme that is. If any of you have a clear vision of what the future has in store for us, please let me know. It has certainly been interesting and hard work these last few months, with many unexpected twists and turns and even more unknowns. It's wonderful to be here today with this kickoff session. Thank you for your participation and for your trust in us. I have just received the count for this session. There are about 500 watching us so far, with a steady flow coming online as the session progresses. Our 2020 NPC team has been extraordinary. Please join Melissa and me in recognizing them. We also want to recognize our Local Assistance Committee (LAC). They had prepared an incredible journey for you to experience Portland. That part, unfortunately, we cannot really share with you in this virtual setting. We look forward to visiting Portland in the future, although no meeting date has been selected for that visit. Please join Melissa and me in recognizing them.

Melissa, let's share with everyone what's in store for this MLA '20 vConference and Exhibits.

Melissa De Santis: Sure, thank you, Janna. Registered attendees will receive an email from MLA with the links to the online planner before the end of this session. Janna and I will now provide an overview of the planner and how to best navigate it. We have designed this MLA '20 vConference and Exhibits into three phases.

Phase 1 is the live kickoff. It includes the opening session, which is happening right now, followed by awards and recognition. The networking event will be later today. It is for first-time attendees and new members. There will be random group networking presumed that will help us all meet new people.

The exploration phase is the second phase of the MLA '20 vConference. It is a two-week period of asynchronous interactions. Pick the best time for you to view, connect, and interact. Fifty-six vendors have been hard at work uploading information, portable document format files (PDFs), videos, and more for you to view. The poster viewing experience will be outstanding. Some presenters have recorded short audio presentations to augment your experience. Watch lightning talks and paper presentations. Your presenters have recorded their slide presentations with voice synced to each slide. During the exploration phase, you'll review all of this content and interact with presenters through the meeting website and meeting app in group conversations. Please be respectful and adjust your expectations to an asynchronous world where responses may be delayed.

Finally, there will be social events where you can relax and have fun with other attendees.

The third and final phase is the live action week. Clear your calendars between August 10 and 14, stay up late if you are out east, and plan to get up early if you're out west.

Let me highlight a few times. For vendors, twelve Solution Showcases will be carried out live and recorded Monday through Thursday. Vendors will also have video chats, so you can meet with your representatives or attend a discussion or information session. Check your schedule for online video chats with paper and lightning talk presenters, grouped by themes. Those gatherings are limited to twenty people to make sure you can actually have a conversation.

The nineteen immersion sessions have been transformed to be effective virtual learning experiences. If you miss one, you can watch the recording after Live Action Week. Don't miss the three keynote speeches, but if you do, they will be recorded. Two of the keynote speeches will be followed by virtual receptions. You will be able to go from room to room to video chat with your host. Pour yourself a comforting drink and sit on your sofa.

We are particularly excited by the open science session. Make sure to watch the introduction presentation during Exploration Week so that you'll be ready to participate when things get serious. Believe it or not, the 2021 NPC is well into planning for next year's meeting. They will be closing Live Action Week right after the open science session.

That was a lot of information. Janna, can you share some tips and tricks since this will be MLA's first vConference?

Janna Lawrence: Sure. A good place to start is accessing the meeting website. Once you are registered for the meeting, you can use your tablet or laptop to go to MLANET, access the online planner, select login to connect with your MLA username and password, or with your MEDLIB-ED credentials if you don't have an MLANET username. And for MLA members, those two things are the same thing. If you're already logged into MLANET, you won't need to log in again. Then you can fill out your attendee profile with your picture, interests, virtual ribbons, and connection preferences. There is a conference app, and if you want to use it, that will be available on August 5. You can use the favorite feature to mark sessions you want to attend, such as Solution Showcases organized by vendors, plenary speaker sessions, and networking events. Set aside time for the virtual exhibit hall, the poster gallery, and on-demand sessions.

For vendors, you can start viewing their exhibits now and chat with them August 10 to 13—that's Monday to Thursday—from 11:30 a.m. to 3:00 p.m., central time. You can also participate in their live educational sessions August 10 to 14, Monday to Friday, from 8:30 a.m. to 10:00 a.m., central. Those sessions will be recorded as well.

Don't miss the live plenary sessions with our featured speakers. Those include Esther Choo on Monday, August 10, for the John P. McGovern Award Lecture; John Brownstein on Tuesday, August 11, for the Joseph Leiter National Library of Medicine (NLM)/MLA Lecture; Chris Shaffer on Wednesday, August 12, for the Janet Doe Lecture; and Patricia Flatley Brennan, Amanda J. Wilson, and Dianne Babski on Thursday, August 13, for the NLM Update. All sessions will be live with Q&A and open to the public. They will also be recorded for future viewing. You can find out more about our speakers in the online program.

We want to emphasize that starting today, you can access on-demand content. This includes all papers, lightning talks, and the virtual poster gallery. Q&A is enabled now. That means you can ask questions and participate in conversations with the presenter and other attendees by typing in questions, like texting them. For papers and lightning talks, though, you'll be able to video chat live at set times from August 10 to 13, so be sure to check the schedule for exact times for each small group of presenters. And, at any time, you can connect with other attendees and presenters one-on-one directly through the website.

The nineteen immersion sessions will be presented live. You can also watch them later, after they're recorded. Our presenters and facilitators have redesigned their sessions for the virtual experience. We've opted to use Zoom for this for a familiar look and feel, including breakouts.

Melissa De Santis: And if I can add one final tip: Although the whole conference will be fun, here is the schedule for games, music, quests, and prizes. Please note that these fun times begin during the two weeks of exploration, so don't miss it.

Janna Lawrence: Well, Melissa, that's a lot. I can't wait to get started later today. Thanks to the speakers and contributed content for being so flexible and for going the extra mile to make this MLA '20 vConference and Exhibits a success. Your participation is essential to strengthening our community. Here's to recognizing our sponsors and exhibitors. Let's welcome again our emcees, Julia and Lisa.

Julia Esparza: Thank you, Janna and Melissa. I can't tell you how much I appreciate all the work the NPC and the LAC did in preparing for this meeting. Please join Lisa and me in giving a heartfelt welcome and thank you to our vendors, who, once again, demonstrated their outstanding support for MLA and the value they hold in our role as health information professionals.

Vendors contribute to MLA in so many ways. In 2019, vendors provided one million dollars, or 36%, of operating revenues. Just imagine what membership dues, meeting registration, and other subscription costs would be without vendor financial support. Vendors provide content that is valuable to all of us. Vendors and librarians collaborate on initiatives that advance our profession. Let's meet our 2020 annual meeting sponsors. Take time to personally thank them for their support throughout the meeting and efforts.

**Bronze:** Providing $5,000 or more of sponsorship in addition to exhibit costs. In alphabetical order:

- American Pharmacists Association
- American Psychological Association
- CABI
- Clarivate
- ClinicalKey
- Elsevier
- Springer Nature
- Wiley

**Silver:** Providing $15,000 or more of sponsorship in addition to exhibit costs:

- JAMA Network

**Gold:** Providing $30,000 or more of sponsorship in addition to exhibit costs:

- McGraw-Hill
- *New England Journal of Medicine* (NEJM) Group

**Platinum:** Providing $45,000 or more of sponsorship in addition to exhibit costs:

- Wolters Kluwer

Let's hear from Jim Shanahan, vice president and group publisher, McGraw-Hill.

Jim Shanahan: Hello. I'm Jim Shanahan, and I'm the vice president of development from McGraw-Hill Professional. I was very fortunate to present to the esteemed MLA group during the spring 2019 meeting. It's really hard to believe everything that's happened in our world since then. But thank you again for setting this virtual conference up. We really value and appreciate the opportunity to engage with MLA.

We're fortunate at McGraw-Hill Professional to work with many esteemed authors, physicians, library science specialists, and information service specialists. I do want to give a special shoutout today to Anthony Fauci, who is editor of our *Harrison's Principals of Internal Medicine,* as well as *Access Medicine.* Dr. Fauci has worked so tirelessly on behalf of the country in developing policy responses as well as vaccination programs and therapeutic programs to help us get through this very strange COVID-19 time. He is really tireless. And every new weekend, I get a new batch of chapters from him for the next edition of *Harrison's,* beautifully edited, perfectly reviewed, on time, and on spec. He really is an extraordinary man.

We're also very proud of our association with MLA. As we think about the COVID-19 world and the post-COVID-19 world, we're going to be looking very hard at what may stick around in terms of distance learning and changes to the way people learn, are educated, or even practice medicine in the post-COVID-19 world. We are really paying attention to news, trends, and analyses of these issues.

A lot of our products, existing and new, are to some extent going to be supplemented in the future with functionalities and content that really maximize their use for some form of distance learning, whether it's hybrid or hi-flex or any form that comes out of this COVID-19 period. But the essential point is, we're utterly convinced that what will come out of the post-COVID-19 world are changes that are almost permanent in the way students are taught, the way people learn, and the way they do their teaching.

We do have new products for 2020 and early 2021. Our major new product launch this year is Access DermatologyDxRx. Our latest entry into the Access product line, in addition to having a collection of really stellar reference titles on the site. It has an amazing collection of crystal-clear clinical photographs. We're just starting beta testing on new application-based resident engagement tools for the surgery market, which we're very excited about, and beta testing an innovative new cancer therapy decision-making tool. Both of these are going to go through beta for the rest of the year, and we hope to launch them early in 2021.

At any rate, thank you again for this virtual conference and for the opportunity to speak with MLA. We really do value our partnership and everything you do for medical learning and information sciences. Thank you.

Julia Esparza: Let's hear from Susan Haering, director, NEJM Group Licensing, representing the *New England Journal of Medicine.*

Susan Haering: Hello, everyone. I'm Susan Haering, director of NEJM Group Licensing. We're very pleased to be here again this year as a Gold sponsor at MLA '20. We've been part of MLA for a lot of years, and it's our favorite event, not just because MLA leadership really rolls out the red carpet, but because it gives us a chance to see all of you, and many of you have become both colleagues and friends.

Over all the years, you've consistently supported us but also challenged us to do better, and we need both of that right now. From all of us at NEJM Group, I want to thank you especially for fighting the good fight these past months, for keeping sound science and not sensationalism moving toward the front lines. So, please enjoy this fantastic new vConference, and do stop by our virtual booth to say hello. We'll have a chair waiting for you.

Julia Esparza: Let's hear from Vikram Savkar, vice president and general manager for medicine markets, Wolters Kluwer.

Vikram Savkar: I'm pleased to be speaking with you today at the opening of the Medical Library Association virtual annual meeting. On behalf of Wolters Kluwer, we're delighted to continue our partnership with MLA and its dedicated members, who strive for excellence in medical research. We share your mission to train the next generation of clinicians and advanced patient treatment supported by the best available evidence, and are proud to be able to work with you in fulfillment of that mission every day.

This year's theme of bringing the future into focus could not be timelier. The COVID-19 pandemic has accelerated the future in ways that bring both challenges and opportunities. We are all aware of the many challenges and are working together to navigate through these times of crisis with as much stability and care as we can.

But the challenges have also brought opportunities that will lead to important new developments. From digital medical education to telemedicine to data sharing and clinical research, we are collectively having to reimagine how we can work better; what access and collaboration can look like in six months or six years; how we can deepen the contribution of evidence-based research to improve health care outcomes and shorten the cycle to arrive at those outcomes. These will not be simple questions to answer, but with the collective insight of all of you gathered together at this conference, I am certain that these challenges can be solved.

I wish you engaging connections and fruitful discussion over the next three weeks in the reimagined virtual experience of the 2020 MLA annual conference. We at Wolters Kluwer look forward to connecting with you throughout the meeting.

Lisa Traditi: Thank you, Julia, and thank you to all of our sponsors. The MLA InSight Initiative advances collaboration between librarians and participating organizations in the publishing industry. Established as a pilot initiative in 2017, the MLA Board of Directors recently made the InSight Initiative a standing program because of its success. Here to share the excitement of the InSight Initiative, please welcome Gerald J. Perry, AHIP, FMLA, 2017–2020 chair of the MLA InSight Initiative Task Force, and John Gallagher, 2020–2022 chair of the newly established InSight Initiative Steering Committee.

Gerald J. Perry, AHIP, FMLA: Thank you, Lisa. It's been a privilege to serve MLA and the health sciences information community as chair of the MLA InSight Initiative Task Force. For those of you who are not familiar with the InSight Initiative, here is a little bit of what it's about. MLA's InSight Initiative is a thought leadership program engaging health sciences librarians with the information providers in high-level, high-value dialogue on issues that matter to both communities. It aims to reinforce, expand, and communicate the value that both communities contribute in moving validated information from the author to the reader.

The premise of the InSight Initiative is to build good will and collaboration between our two communities. We achieve this through a series of five InSight Initiative summits, four held in Chicago, and the last one held virtually in March. I'm pleased to report that we established a safe, trusting environment and a model for collaboration. The summit participants, split evenly between librarians and publishers, demonstrated their interest and ability to work collaboratively on initiatives to build bridges between our two communities.

The themes and work accomplished at each summit evolved and matured in the time between March 2018 and March 2020. Summit 1 addressed the shared goals of librarians and publishers to engage users of health sciences information in an era in which disrupters such as pirate websites, scientific collaboration networks, and preprint servers pose threats to traditional means of access to scholarly content. Following closely on summit 1's observations, summit 2 showed that both librarians and information providers need to focus more intently on users and how they can collaborate in explaining to users why licensed and sometimes costly content has real value. The findings from summit 3 on bridge building were so surprising that we repurposed that information for the McGovern Lecture for the 2019 annual meeting. We also held an immersion session at the same annual meeting to share with our community other components of our findings. Summits 4 and 5 built upon the issues and key outcomes that summit 3 participants identified, with the same participants to ensure continuity. We focused on producing a set of tangible outcomes through multiple small group work sessions during the summits and then throughout the year.

We are happy to show those today, but before we do that, I want to recognize the participants.

- American Psychiatric Association Publishing
- American Psychological Association
- American Society of Health-System Pharmacists (ASHP)
- Annual Reviews
- BMJ Publishing
- F1000
- JAMA Network
- McGraw-Hill
- NEJM Group
- Oxford University Press
- Springer/Nature
- Wolters Kluwer

Our participating organizations provided the voice of the publishing industry as well as funding for the summits facilitators and travel of library participants. Thank you for your commitment to time, energy, and resources, and for sharing your knowledge and expertise with us. Time is a precious commodity for our organizational participants, and we truly appreciate the hours you have spent outside of our summit meetings to develop products useful for our communities. The trust we have developed through our collaborative work has laid a foundation for better understanding of our user community and how best to support their needs for information access.

I also want to thank the many participating librarians for their commitment to the MLA InSight Initiative. They provided the voice and expertise of the librarian community and committed significant time to research and to develop products and the outcomes that will benefit all of our users as well as librarians and publishers.

Here, then, is an overview of InSight Initiative outcomes. We developed short videos, we created an InSight Initiative end user advisory board, and we created an online forum to raise and discuss issues related to the discovery and access for users.

Here, then, are the titles of videos that you can now access on MLA's YouTube channel and through MLANET's landing page. These are licensed under our Creative Commons attribution, noncommercial 4.0 international license so that publishers and librarians can redistribute them. So, let go ahead and watch one.

[Video.]

Why are predatory journals dangerous? Predatory journals often serve at an outlet for plagiarized or fabricated results. Some authors are motivated to publish quickly or frequently to gain tenure or promotion. They need to publish, no matter the quality or validity of their research. A predatory publisher doesn't care about false claims and results; it's motivated to publish more articles and collect more author fees.

Predatory journals can damage your reputation. Once a journal is recognized by the scholarly community as predatory, its negative reputation is often extended to the authors who have published in them, the author's institution, and maybe even the entire discipline. Even if a research article is based upon good science and reveals important discoveries, the reputation of the journal may tarnish the research and cause others to question its validity.

Bad science can harm patients as well. Patients seeking medical advice and information might discover articles in predatory journals and mistakenly think the findings are legitimate. This could lead patients to disregard the advice of their doctors or fall prey to bogus or possibly harmful treatments.

[End of video.]

Jerry Perry: We've created a PowerPoint presentation that identifies several vexing problems or pain points limiting a user's ability to access information, potential solutions, and level of impact. The presentation is accessible on the InSight Initiative MLANET page and will be published in the January 2021 issue of the *Journal of the Medical Library Association (JMLA)* as part of a scholarly article.

So, thank you for the opportunity to serve as chair during the pilot of this exciting new initiative. This has been a truly rewarding experience, and it has been a privilege. As we look forward to the next stage of the MLA InSight Initiative, I am thrilled that John Gallagher has agreed to chair the new standing committee. John will talk about future directions.

John Gallagher: Thank you, Jerry. Hello, everybody. In its new two-year cycle, the InSight Initiative will include multiple, virtual or face-to-face interactions and specific outcomes agreed to by the participants. We'll continue to focus on identification of the vexing problems and transformational opportunities our communities share. To lead us through this cycle, I am joined by a new Insight Initiative Steering Committee with representatives from both the publishing and library communities. Later this fall, we'll be hosting a forum to help us identify the next vexing problem that we want to jointly tackle. This forum will be open to any interested librarians or publishers, and we really look forward to your participation and input. We'll then be identifying the themes for subsequent summits.

If we've piqued your interest, watch *MLAConnect* for the call to participate in these summits later this fall. Librarians will be asked to accept a two-year participation commitment for the full cycle. We will notify participants in March 2021. Thank you, and back to you, Julia and Lisa.

Julia Esparza: Jerry and John, thank you so much for your inspiring and fun presentation. Collaborations such as the InSight Initiative are challenging, and you have made this initiative a huge success, which benefits both our communities. The MLA Board fully supports your efforts, as do members and participating publishers. Onto the next and final segment of our opening session and what you've all been waiting for: recognizing the achievements of our fantastic members.

Lisa Traditi: Before we get started with awards and fellowships, MLA has a tradition to take a few minutes to recognize the distinguished members who passed away in the last year. Their counsel and friendship will be deeply missed. We're produced this video in honor of their memory.

[A video was shown honoring these members.]

Lisa Traditi: Truly inspiring and beautiful. Thank you. Several of our distinguished awards and fellowships are named after colleagues who have passed away in the last year: Lucretia W. McClure and Donald A. B. Lindberg. They will be greatly missed. Julia, let's get started with our recognitions and awards. This year, you won't be coming on stage to receive your award. Instead, Julia and I will alternate in presenting the awards. We will see the names on the screen, and for those very special moments, we will hear from award recipients in their recorded videos.

Julia Esparza: Over the years, MLA has been extremely fortunate to have many talented members serve in various MLA editorial and coordinator positions. I would like to recognize them today starting from the top and going counterclockwise:* *MLAConnect* editor: Christine Willis, AHIP

- *Journal of the Medical Library Association (JMLA)* editor: Katherine G. Akers
- MEDLIB-L coordinator: Richard James
- Oral History Project Director: Carol Lipscomb, AHIP, FMLA

Thank you for all your efforts on behalf of the association.

Lisa Traditi: MLA advances its mission through its programs and services. Those initiatives are successful thanks to the committed work of MLA volunteers in caucuses, committees, juries, and task forces, and as allied representatives. We recognize our volunteers for your expertise and time in advancing MLA and the profession.

Julia Esparza: This membership year, 328 new members joined MLA. New members bring fresh ideas and energy and are essential to MLA's future. Don't forget to join the new member/first-time attendee networking event later today. It's a great opportunity to meet some new “frolleagues.” What is a “frolleague?” Well, that's a term for friends and colleagues coined by M.J. Tooey, AHIP, FMLA.

Lisa Traditi: The Credentialing Committee evaluates and grants membership to the Academy of Health Information Professionals, which you know as AHIP. There are 1,060 academy members. This is an impressive 40% of MLA members who have earned the academy membership. These slides show the names of the 75 new academy members since the 2019 meeting, including all levels: provisional, member, senior, distinguished, and emeritus.

Julia Esparza: From coast to coast—including Alaska, Hawaii, and several Canadian provinces—MLA's thirteen chapters serve as an important home for members. They offer regional meetings, grants and scholarships, and other programs at the local and state level. Congratulations to the award recipients from our MLA chapters. Let's view them by chapter.

- Hawaii-Pacific Chapter (HPC-MLA)
- Medical Library Group of Southern California and Arizona (MLGSCA)
- Mid-Atlantic Chapter (MAC)
- Midwest Chapter
- New York-New Jersey Chapter (NY-NJ)
- North Atlantic Health Sciences Libraries (NAHSL)
- Northern California and Nevada Medical Library Group (NCNMLG)
- Pacific Northwest Chapter of MLA (PNC)
- Philadelphia Regional Chapter (MLA-Phil)
- South Central Chapter (SCC)
- Southern Chapter (SC)
- Upstate New York and Ontario Chapter (UNYOC)

The MLA Chapter Project of the Year Award recognizes excellence, innovation, and contributions to the profession of health sciences librarianship. This year's recipient is the Pacific Northwest Chapter of MLA for their new logo pin design contest. Let's hear a few words from chapter president, Kathryn Vela, AHIP.

Kathryn Vela, AHIP: Hello. I'm Kathryn Vela, the chair of the Pacific Northwest Chapter of MLA, and on behalf of our chapter, I'm honored to accept the award for MLA Chapter Project of the Year Award. Our logo pin design contest was such a fun and creative way to engage our members with the chapter, and the funds that we raise in the sale of the logo pin design will be used to fund two scholarships to this year's MLA '20 vConference. So, I want to say thank you to everyone in our chapter who made this award possible, and I look forward to seeing what we do next.

Lisa Traditi: Thank you, Kathryn. When MLA converted its in-person meeting to a virtual conference and exhibits, the annual meeting travel grants were also converted to provide 150 free registrations for members needing financial assistance. Thanks to our sponsors, McGraw-Hill and JAMA Network, MLA increased its financial support from $35,000 to $54,000 by using their generous financial support to augment contributions from MLA's endowment and general budget. MLA thanks you for your financial support at a time when it is so essential for our members who are unable to fund their own professional development.

Julia Esparza: The MLA Scholarships are awarded to students enrolled in or entering an MLA-accredited library school. This year's recipients are Samantha Kennefick Wilairat, University of Colorado, Anschutz Medical Campus; and Christiana Julsaint, University of North Texas.

Lisa Traditi: The purpose of the Cunningham Memorial International Fellowship is to assist in the education and training of health sciences librarians from countries outside the United States and Canada. Sadly, though it's not a surprise, this year's fellow was unable to travel. But we do have a video. Let's hear from this year's recipient, Anar Kairatovna Dautova, with her greeting from Kazakhstan.

Anar Dautova: Hello. My name is Anar Dautova. I work at the medical library at Nazarbayev University, a university in Kazakhstan. I really appreciate the MLA Cunningham fellowship for the great opportunity for health sciences librarians to learn and grow professionally. Especially, in Kazakhstan, we don't have a special program for medical librarians so it's not only me that gets this grant, but also all Kazakhstani medical librarians, because I will transfer all my knowledge to them, and we step up to a new world together. Due to the pandemic situation this year, we all learned how to work from a distance and manage our online services. The Nazarbayev University Library provides all necessary virtual services and support for users during distance learning. Thank you and stay safe during this extraordinary circumstance.

Julia Esparza: Let's continue our world travels, Lisa. The MLA Librarians without Borders®/Elsevier Foundation/Research4Life Grants are an expansion of the Elsevier Foundation Librarians without Borders e-library training initiative. This program supports Hinari Research for Life training activities that promote the use of scientific resources in emerging, low-income countries. There were five grant recipients for 2019–2020. Let's hear how they are managing COVID-19 in their libraries.

Francina Ngula Simataa Makondo: Hello. This is Francina Makondo from Lusaka, Zambia. During this COVID virus pandemic, we've been coping by ensuring that we conduct a lot of online meetings, a lot of training, and of course, we've been processing materials and ensuring that we register our users for remote access to the resources that we have so that as people are home, they can still be in touch with the library.

Deodatus Sabas: Hello. About two months ago, universities and colleges in Tanzania were shut down as the government attempted to contain the spread of the coronavirus. The university is open and now things are slowly getting back to normal. At this time, the library is following what the rest of the university campus does. Our physical spaces are open, and the library remains operational as well. Some services such as requests for items have shifted to online to avoid contracting the virus through this means. Also, support such as user education programs through training workshops has been carried out with a limited number of participants sitting at a distance from another as a means of avoiding the spread of the coronavirus. Washing hands and wearing masks is part of us now, as well as sitting and standing at a distance from one another is the new normal at the campus. Thank you.

Fred Kwaku Hayibar: On how we are adapting to the COVID-19 pandemic in Ghana, of course, like most places around the world, we have to adapt all our teaching to online and conducting our research and collecting data, and our researchers will have to switch to some other reviews, and that means the library has a lot of work to do in teaching how to have these conversations. So of course, we have had to follow strict protocols with the wearing of masks, washing our hands, and physical distancing. That's how we have been coping. Thank you.

Lisa Traditi: I want to take one moment and apologize for inadvertent error. We left out the Midcontinental Chapter in the chapter awards, and I want to congratulate awardees from the Midcontinental Chapter.

The T. Mark Hodges International Service Award honors an outstanding individual achievement in promoting, enabling, and/or delivering improvements in the quality of health information internationally. The 2020 award is presented posthumously to Laura Shane Godbolt for her significant service, leadership, and passion in global health sciences librarianship. Shane's good friend and colleague, Donna B. Flake, AHIP, FMLA, will share a few words with you.

Donna B. Flake, AHIP, FMLA: I want to say how much I'm happy that Shane got the Mark Hodges award. She deserves it more than any librarian I know. She reached out with her arms wide around all the medical libraries I can think of in international areas and embraced them. And Shane, cheers for you.

Julia Esparza: The Naomi C. Broering Latinx Heritage Grant provides the librarian with an interest in Latinx community information services the opportunity to pursue a professional activity and cutting-edge medical information services, using the latest technical formats. This year's recipient is Nora Franco. The grant will allow Nora to continue her professional development and gain knowledge of the medical informatics and health care environment to provide health information services.

Lisa Traditi: The Continuing Education Grant supports continuing education to develop applicants' knowledge of the theoretical, administrative, or technical aspects of librarianship. This year's recipient is Marilia Y. Antunez, AHIP. Marilia plans to participate in the online Critical Appraisal Institute for Librarians, or CAIFL, which will benefit her in the reference and instruction services that she provides at her institution, particularly in the areas of evidence-based practice.

Julia Esparza: The Hospital Libraries/MLA Professional Development Grant provides librarians working in hospitals or clinical settings with the support needed for educational or research activities. This year's grant is given to two recipients. Danielle N. Linden, AHIP, will use the grant for professional growth in the area of leadership and management. Louisa Verma, AHIP will use the grant to advance her research techniques.

Lisa Traditi: The Medical Informatics/MLA Career Development Grant provides support for career development activities that will contribute to the advancement of the field of medical informatics. Susanne Fricke, AHIP, will use this grant for the Oregon Health & Sciences University and American Medical Informatics Association “10 by 10 Introduction to Biomedical and Health Informatics” virtual course.

Julia Esparza: The Virginia L. and William K. Beatty Volunteer Service Award recognizes a medical librarian who has demonstrated outstanding and sustained service to MLA and the health sciences library profession. Let's hear from the 2020 recipient, Margaret A. Hoogland, AHIP.

Margaret A. Hoogland, AHIP: Hi, my name is Margaret Hoogland. I'm a clinical medical librarian at the University of Toledo. I'm honored to be the 2020 Virginia L. and William K. Beatty Volunteer Service Award recipient. Over the years, I have benefited tremendously by serving professionally and personally. I've met a lot of new people, and I've had a lot of fun. So, I would encourage you to give service a try. Thank you.

Lisa Traditi: The Frank Bradway Rogers Information Advancement Award is presented in recognition of outstanding contributions in the use of technology to deliver health sciences information, in the science of information, or in facilitation of the delivery of health sciences information. This year's recipient, Gail Kouame, has a few words to share with us today.

Gail Kouame: Hello, I'm Gail Kouame, and I'm honored to receive the Frank Bradway Rogers Information Advancement Award this year on behalf of the entire team who made this project possible. I'm particularly proud of this work because it addresses the needs of one of our nation's invisible populations, the incarcerated. The data from our project show that the health education modules we've provided through secure tablets made a significant impact. The hope is that when incarcerated individuals return to the community, they are better prepared to manage their health and make better informed health care decisions. Thank you very much.

Julia Esparza: The Estelle Brodman Award for the Academic Medical Librarian of the Year is given to a member who has made an outstanding contribution to academic medical librarianship. I am pleased to share this video from Joey Nicholson, the 2020 recipient.

Joey Nicholson: I am so honored to have been selected as the recipient of this year's Estelle Brodman Award for the Academic Medical Librarian of the Year. To explain what this means to me, I have to use a term coined by one of the previous recipients, M.J. Tooey, “frolleague.” My frolleagues in the medical library community are among my best friends and best collaborators. I really couldn't have gotten to where I am without a huge network of frolleagues. Having their support earning this award from them truly means a lot. Thank you.

Lisa Traditi: The Lois Ann Colaianni Award for Excellence and Achievement in Hospital Librarianship is given to a member who has made significant contributions to the profession through overall distinction in hospital librarianship. This year's award is given to Barbara S. Reich, AHIP. Barbara is well known for her commitment to mentoring young librarians. Her amazing work with the Health Sciences Library Association of New Jersey group licensing initiative has made an impact not just on her own patrons, but those patrons at 144 other hospital libraries as well.

Julia Esparza: The Consumer Health Librarian of the Year Award recognizes a consumer health librarian who exemplifies the best in consumer health librarianship. We are pleased to present the 2020 award to Antonio P. DeRose, AHIP. Antonio is known to have the skills of an information professional, the vision of an administrator, and the empathy and listening skills of a social worker. As an oncology consumer health librarian, he has made a significant impact beyond expectations through his passion for transforming practice.

Lisa Traditi: The Louise Darling Medal for Distinguished Achievement in Collection Development in the Health Sciences recognizes accomplishment in collection development for the health sciences. This year's winner is the Association of Vision Science Librarians (AVSL) Vetted List (previously, White List) of Vision Science Journals working group and the AVSL-vetted list reviewers for a robust application that documented significant achievement in regard to collection development. The project itself addresses the ongoing challenge predatory publishing presents in the world of open access. This list and process of evaluation will help save librarians time in the future when responding to questions about the credibility of publications.

Julia Esparza: Before we honor the recipient of the Lucretia W. McClure Excellence in Education Award, we are fortunate to have recordings of Lucretia, which were made when MLA interviewed her for her oral history. Let's listen to a clip where she talks about education of medical librarians.

[A video was played.]

Lucretia W. McClure: I think my greatest concern today is about the education of librarians, because I feel like we should be an active part with our colleagues in the library schools. I've hired a good many young people over the years and they're all good people, worthy people, but there are so many things they don't know that I think they would benefit from in preparation for working in a medical library.

I think, for example, that we should be a part of the review team that goes to look at the medicine classes in library schools. I have no idea what they teach today other than computers. I don't really know what they do. But there were so many basic things that young people did not know when they came to work that I think they would have known had they had a class that developed this area.

So, I think we ought to be partnering with our colleagues in the library schools. We do have faculty who come to MLA, but I don't think we have anything to say about it; at least, I don't know of any relationship directly that we have with them. And I think we should have more contact with library schools. I think the future of librarians is going to be directly related to more science. I think they need more biology, I think they need more genetics, I think they need more of what the medical schools are utilizing. And I don't know that the library schools can do that or should do it, but I think we need to find out about it. I think there are skills that we're going to need down the road, and I don't know that we're prepared for it.

[End of video.]

Julia Esparza: The Lucretia W. McClure Excellence in Education Award honors outstanding practicing librarians who are library educators in the field of health sciences librarianship and informatics. Let's hear from the 2020 recipient, Amy Blevins.

Amy Blevins: Hi, everyone. I don't have the words to adequately express how humbled and honored I am to receive the Lucretia W. McClure Excellence in Education Award this year. I am so sad that we cannot be together in person. So, I will just have to say this from my dining room here in Indianapolis: To all my friends, colleagues, or frolleagues, as M.J. Tooey would say, I want to thank you from the bottom of my heart for your support, inspiration, and friendship throughout the years. I hope we can be together in person again soon.

Lisa Traditi: The MLA Rising Star Program gives members the opportunity to develop skills, knowledge, and personal characteristics needed to become a leader in MLA. The yearly leadership development program matches each Rising Star with a mentor in a comprehensive curriculum. I am pleased to introduce you to the incoming 2020–2021 Rising Stars cohort: Alyssa Kathryn Migdalski, Kathleen Elizabeth Phillips, JJ Pionke, and Erin Smith. We look forward to seeing and hearing more from our new stars in the coming year. I hope you had a chance to attend the MLA Rising Star open forum two weeks ago featuring the 2019–2020 MLA Rising Stars: Kelsa Bartley, Kathryn Houk, AHIP, Jane Morgan-Daniel, AHIP, and Elaina Vitale. Here to tell us more about their Rising Star experiences, let's watch this video they created.

[A video was played.]

Kelsa Bartley: Hello, we are the 2019–2020 MLA Rising Stars: Kelsa Bartley, Kathryn Houk, Jane Morgan-Daniel, and Elaina Vitale. Our project this year was focused on examining the strengths and challenges of the community's transition project through a market analysis of guided interviews with various MLA members and leaders.

Kathryn Houk, AHIP: Through our year-long program, we were introduced to different theories, models, and aspects of leadership through readings and discussions with leaders in the MLA community. We were asked to examine our personal strengths, weaknesses, and preferences as we envision leadership in medical librarianship and the organization.

Jane Morgan-Daniel, AHIP: This program has introduced us to MLA members we had previously never dreamed we'd get the chance to speak with and led to, hopefully, a career-long connection between our cohort. We practice leadership within our cohort as we work on our project and are able to see concepts from our discussions in the interview transcripts.

Elaina Vitale: We encourage any MLA member hoping to explore leadership and their own leadership traits to apply for the Rising Stars program. No matter if you have a title, everyone can step up to leadership in some capacity. This program can help start you on a path to discovery of your opportunities and passions for leadership.

All: Thank you, MLA!

[End of video.]

Julia Esparza: The Ida and George Eliot Prize is awarded for a published work that has been judged most effective in furthering medical librarianship. This year's corecipients are Peace Ossom Williamson, AHIP, and Christian Minter. Their article, “Exploring PubMed as a Reliable Resource for Scholarly Communications Services,” is a reliable resource for scholarly communication services and brings new insights into the workings and internal processes of NLM. It will spark further conversations about the quality of publications in a variety of open-access sessions, not just PubMed, PNC, and MEDLINE.

Lisa Traditi: The Rittenhouse Award recognizes the best unpublished paper on medical librarianship. Sara Clarke, AHIP, is this year's recipient. Her paper, “Blinded Data: Sharing Barriers during Disease Outbreaks,” went beyond reviewing the literature and included interviews with people involved at the federal and organizational level. Sara made it very clear how health sciences librarians can play a prominent role in data sharing during outbreaks. We all look forward to reading Sara's work once it is published.

Julia Esparza: The Eugene Garfield Research Fellowship was established to promote and support research in the history of information science in the medical or health sciences. This year's recipient of the Garfield Fellowship is Toluwase Victor Asubiaro. The aim of his research is to identify what role, if any, has been attributed to librarians and information professionals who are affiliated with institutions in Africa in conducting biomedical systematic reviews. Data will be collected from MEDLINE and analyzed using bibliometrics and content analysis methods.

Lisa Traditi: The David A. Kronick Traveling Fellowship is awarded annually to cover expenses involved in traveling to medical libraries in the United States or Canada for the purpose of studying a specific aspect of health information management. This year's recipient is Mark MacEachern. Mark noted that research reproducibility has become more prominent in the health sciences librarian community. He intends to develop a sustainable and scalable reproducibility service model at his library and create a template for other libraries to follow. We hope that Mark will still travel, even if it's just virtually.

Julia Esparza: Before we announce the Donald A. B. Lindberg Research Fellowship recipient, we would like to share a brief clip with you from Dr. Lindberg's oral history.

[A video was played.]

Donald A. B. Lindberg: And I guess what I learned at Amherst was two things, both of which were surprises. One, I would say, is the thrill of discovery. It's pretty fantastic. How many [times] in your life, to go and do an experiment and discover something that no one in the whole world knew. And then you can write it down and publish it and then everyone can know it? Well, that's a process that I found very striking.

[End of video.]

Julia Esparza: The Donald A. B. Lindberg Research Fellowship funds research linking the information services provided by librarians to improved health and is awarded to a qualified health professional, researcher, educator, administrator, or librarian. The 2020 Lindberg Research Fellowship is awarded to Antonio P. DeRosa, AHIP. His research is titled “Shared Decision-Making among Breast Cancer Patients: A Phenological Study in Exploration into Health Literacy Interventions.” The expected impact is to understand the experience of breast cancer patients in making decisions in partnership with their providers and to explore health literacy information interventions to support the decision making of this patient population.

Lisa Traditi: The Research Advancement in Health Sciences Librarianship Award recognizes organizations whose exemplary actions have served to advance health information research and evidence-based practice in health sciences libraries. We are so pleased to have two recipients this year: the Samuel J. Wood Library at Weill Cornell Medicine in New York, New York, and the Tompkins-McCaw Library for the Health Sciences at Virginia Commonwealth University (VCU) in Richmond. Your colleagues at VCU would like to say hello to you today.

[A video was played.]

Video narrator: Research librarians and staff from Virginia Commonwealth University Libraries, Tompkins-McCaw Library for the Health Sciences, located in Richmond, Virginia, are proud to be recognized with the MLA Research Advancement Award. The efforts of faculty and staff in all departments across VCU libraries have all equally contributed to this significant recognition.

Tompkins-McCaw Library group: Thanks, MLA!

[End of video.]

Julia Esparza: The MLA Research, Development, and Demonstration Project Grant supports projects that will promote excellence in the field of health sciences librarianship and information sciences. The 2020 recipient is Robin Champieux. Her project addresses the lack of internships and training for potential health sciences librarians. It is an important issue affecting the entire profession as a whole and supports MLA's mission for recruitment, membership, and leadership in the profession.

Lisa Traditi: Fellows of MLA are chosen for their outstanding contributions to health sciences librarianship and to the advancement of the purposes of MLA. The 2019/20 Board of Directors named two association members as MLA fellows.

Teresa L. Knott, AHIP, FMLA, has provided exemplary leadership to the members of MLA through many years of participation in the association, including as a member of the Board of Directors and as the 2016/17 MLA president. Teresa is an advocate for professional education librarians and fostering of their role in the academic health sciences center.

Michelle Kraft, AHIP, FMLA, is an active and committed leader, where she has served with great commitment on numerous committees, including the Board of Directors and as the 2015/16 MLA president. Michelle has a stellar reputation as a health sciences information professional and is a diplomat of our profession to others.

Teresa and Michelle now have FMLA as an added credential right next to their AHIP. Congratulations to you both.

Julia Esparza: Each year, the MLA president honors health sciences librarians who have made outstanding contributions to the profession. My year for this 2019/20 privilege was to give out the MLA President's Award. Here is a recap of the 2019/20 President's Award that I gave out in May during the virtual business meeting.

Susan Lessick, AHIP, FMLA, for her work serving as chair of the Research Imperative Task Force in establishing MLA's Research Training Institute, or RTI. Earlier this month, Susan and her team of faculty led the first virtual RTI. Twenty librarians attended this successful session and will be working on the research projects with vendors in the next year. Thursday of last week, MLA received the great news that the Institute of Museum and Library Services (IMLS) has selected RTI to receive a second Laura Bush 21st Century Librarian Program Award, which will start this year. The grants will support RTI's further efforts to scale up research training librarians by developing a comprehensive online, blended learning program. Congratulations, Susan, for raising the bar once again.

Gerald J. (Jerry) Perry, AHIP, FMLA, for championing the MLA InSight Initiative since its inception in 2016. You heard Jerry share the results of the InSight Initiative earlier in this session.

Several committees, task forces, and members were honored for leading the change in MLA's member communities, known as caucuses. Years of analysis, discussion, and at times, disagreement led to bold decisions and structural change that had not happened since the 1970s. The ultimate objectives were to become a more inclusive, diverse, and relevant community and to provide an enhanced personal experience.

I recognize the members of the following as President's Award recipients:

- Futures Task Force
- Strategic Priorities Task Force
- 2016–2017 Rising Stars and mentors
- Communities Strategic Goal Task Force
- Communities Transition Team

They will all receive a new, unique MLA lapel pin. When we are together in person again, I hope they will wear their pins proudly. Thank you all.

Lisa Traditi: It's my turn this year, and I'm looking forward to it. The good news is that MLA continues to reinvent itself and reach new heights, so I should have no problem identifying the next President's Award recipient.

The next distinction is a very special one. The Marcia C. Noyes Award is the highest honor that MLA confers on any individual. I am pleased that M.J. Tooey, AHIP, FMLA, the 2019 Marcia C. Noyes Award recipient, is available to introduce this year's recipient, as is our tradition.

M.J. Tooey, AHIP, FMLA: Hello, MLA friends, colleagues, and frolleagues. I am M.J. Tooey, the 2019 Marcia C. Noyes Award recipient coming to you via the magic of video from my home office, where I've been captive since March 14. Today, I have the distinct pleasure, thrill, and honor of introducing this year's Marcia C. Noyes Award recipient, Gerald (aka “Jerry”) Perry, AHIP, FMLA. The Marcia C. Noyes Award, MLA's highest honor, is named for one of the eight charter members and the first woman president of MLA. MLA tradition has it that the previous year's recipient introduces the current recipient. That would be me and Jerry.

When I reached out to Jerry for his CV, so I could give him a proper Noyes introduction touting all his many achievements and accomplishments, he did send his CV, but he also admonished me to tell the truth. So, what is the truth? Well, I don't know about you, but I need someone who will be significant and/or have an impact on my life. I remember exactly where I was at the time. That is a cosmic signal to me. And though I knew of Jerry, I actually met him at the 1994 MLA Annual Meeting in San Antonio, Texas. I can picture standing in the sun along the River Walk and talking with him, and that memory has stuck with me, and my cosmic signals are never wrong. Jerry is significant in my life and in the lives of many others.

Although Jerry began his career in the Midwest, he has become a true son of the Southwest. Yes, he's been at the University of Arizona since 2015 as the associate dean, University of Arizona Libraries, and for now, he's the director of the University of Arizona's Health Sciences Library as well. And, yes, from 2003 to 2015, he was at the health sciences library at the University of Colorado, Denver, where he was a director for eight years.

He was 2011/12 president of MLA, he served on the Board of Directors, and he is a Fellow of our association. He has served on committees and task forces and has been a prolific author, presenter, and thought leader over the years. Also, who can forget his stunning Janet Doe Lecture last year, “The Activist Health Sciences Librarian,” where he called out, expanded, and set the stage for issues that have become even more relevant this past year.

So, I guess we can add “visionary” to his list of accomplishments. So, yes, Jerry cares about our association, and, yes, he also cares about our profession. But he also cares about humanity. And caring about humanity is more important than an entire list of accomplishments. That is the true measure of a person. Jerry is a Renaissance man, with interests far exceeding his professional portfolio: music, art, social justice, diversity, equity, and inclusion. He melds these into an insightful and thoughtful person. He encourages others. He is a whole person.

So, the downside of this pandemic and virtual conference is that he cannot hear the roar of the applause or see the standing ovation he would surely receive. So, please stand where you are, applaud, whoop, and join me in congratulating Jerry Perry as the 2020 recipient of the Marcia C. Noyes Award. Thank you.

Lisa Traditi: Congratulations to Jerry on receiving this honor. Now let's hear from the man himself: Jerry Perry.

Jerry Perry: Thank you, M.J., for that lovely introduction and for all your amazing grace and style. I humbly accept the Marcia C. Noyes Award with deep appreciation for the team that came together and nominated me. I also am appreciative of the larger organization that has provided me with a professional association home for, gosh, just over thirty years.

My first MLA annual meeting was in 1987, ironically, in Portland, Oregon. And while some of the details escape me, I remember clearly the awe of attending my first national library association conference and the brilliance of the many speakers and leaders. They inspired me, and I remain inspired by MLA and its members, who work so hard and tirelessly on all our behalf to support access to quality health information for those in need. It's for that reason that I'm proud of the membership, I'm proud of the loving kindness that's been extended to me, and I'm very proud to receive this award. Thank you to everyone, and please stay safe in your home.

Lisa Traditi: In addition to a wonderful silver bowl from MLA, Jerry will be presented with flowers from the Foundation of MedChi, the Maryland State Medical Society where Marcia C. Noyes worked and lived for fifty years. Thank you to Jerry's colleagues at University of Arizona Libraries for coordinating the presentation of the silver bowl and flowers to Jerry.

Julia Esparza: This year's award presentations remind us of the outstanding accomplishments our peers make to the profession of health sciences librarianship. It also encourages us to continue to reach for new levels of achievement. Congratulations, everyone!

Lisa Traditi: Thank you, frolleagues, for joining us today to kick off the 2020 vConference. We look forward to spending time with you between now and August 14 for a variety of fun events and engaging sessions. The opening session is now closed.

## OTHER PLENARY SESSIONS

The following plenaries were live cast events due to the COVID-19 pandemic.

### Plenary Session 2. Monday, August 10, 2020, John P. McGovern Award Lecture

How Health Care Inequities Have Been Exacerbated by COVID-19

**Esther Choo, MD, MPH,** professor, Center for Policy and Research in Emergency Medicine, Oregon Health & Science University, Portland, OR

Introduction: Janna C. Lawrence, AHIP, deputy director, Hardin Library for the Health Sciences, University of Iowa, Iowa City, Iowa

### Plenary Session 3. Tuesday, August 11, 2020, Joseph Leiter NLM/MLA Lecture

Digital Epidemiology and the COVID-19 Pandemic

**John S. Brownstein, PhD,** professor, Biomedical Informatics, Harvard Medical School, and chief innovation officer, Boston Children's Hospital

### Plenary Session 4. Wednesday, August 12, 2020, Janet Doe Lecture

The Move to Open: Medical Library Leadership in Scholarly Communication

**Chris Shaffer, AHIP,** university librarian, University of California, San Francisco, CA

Introduction: Gerald (Jerry) Perry, AHIP, FMLA, associate dean and director, University of Arizona Health Sciences Library, University of Arizona Libraries, University of Arizona, Tucson, Arizona

## PROGRAM SESSIONS

Program sessions were available in an on-demand viewing format and a live format. On-demand viewing for more than 150 posters, more than 90 papers, and 34 lightning talks began July 27, 2020; some of these sessions are available on an ongoing basis.

Program abstracts that were scheduled to be presented are available on the MLA '20 meeting website and in a supplemental appendix to these proceedings.

MLA '20 vConference live sessions were presented in the following time slots: Monday, August 10, 2020, 8:30 a.m.–6:30 p.m.; Tuesday, August 11, 2020, 8:30 a.m.–6:00 p.m.; Wednesday, August 12, 2020, 8:30 a.m.–7:00 p.m.; Thursday, 8:30 a.m.–4:30 p.m.; Friday, 9:00 a.m.–3:45 p.m. Featured during these time slots were 19 immersion sessions, 90+ papers, and 34 lightning talks. The live immersions sessions included interactive breakout sessions, question-and-answer sessions, and virtual chat with presenters.

## POSTER SESSIONS

The Poster Gallery featured more than 150 posters in an on-demand viewing format beginning July 27, 2020. These included audio presentations and virtual and/or chat question-and-answer sessions with the authors.

Poster abstracts that were scheduled to be presented are available on the MLA '20 meeting website and in a supplemental appendix to these proceedings.

## OTHER MEETINGS AND EVENTS

Due to the COVID-19 pandemic, the following meetings were held virtually prior to or following MLA '20: 2020 National Program Committee, August 21, 2020; 2021 National Program Committee, September 23, 2020; African American Medical Librarians Alliance Caucus, April 30, 2020; Animal and Veterinary Information Specialist Caucus, July 29, 2020; Awards Committee, May 15, 2020; Bylaws Committee, September 25, 2020; Cancer Librarians Caucus, May 5, 2020; Chapter Council, May 21, 2020; Clinical Librarians and Evidence-Based Healthcare Caucus, June 22, 2020; Collection Development Caucus, May 14, 2020; Community Council, May 20, 2020; Consumer and Patient Health Information Caucus, July 24, 2020; Credentialing Committee, April 2020; Data Caucus, April 6, 2020; Dental Caucus, May 21, 2020; Fellows of MLA, September 17, 2020; Governmental Relations Committee, September 18, 2020; Grants and Scholarships Committee, May 8, 2020; Health Association and Corporate Librarians Caucus, April 20, 2020; History of the Health Sciences Caucus, April 9, 2020; Hospital Library Caucus, November 3, 2020; International Cooperation Caucus, April 21, 2020; Joint MLA/Association of Academic Health Sciences Libraries (AAHSL) Legislative Task Force, September 23, 2020; *Journal of the Medical Library Association (JMLA)* Editorial Board Meeting, September 11, 2020; Latinx Caucus, May 21, 2020; Leadership and Management Caucus, May 29, 2020; LGBTQIA+ Caucus, May 20, 2020; Medical Informatics Caucus, August 26, 2020; Membership Committee, April 15, 2020; *MLAConnect* Editorial Board, April 13, 2020; New Members Caucus, May 6, 2020; Nursing and Allied Health Resources and Services Caucus, May 12, 2020; Oral History Committee, July 24, 2020; Pediatric Librarians Caucus, June 11, 2020; Professional Recruitment and Retention Committee, April 2020; Public Health/Health Administration Caucus, April 13, 2020; Research Caucus, April 20, 2020; Technical Services Caucus, June 18, 2020; Technology in Education Caucus, May 18, 2020; Translational Sciences Collaboration Caucus, April 6, 2020; User Experience (UX) Caucus, July 10, 2020; Vision Science Caucus, May 26, 2020.

## CHAPTER MEETINGS

Chapter meetings took place during 2020: Hawaii-Pacific Chapter Meeting, October 23, 2020; Mid-Atlantic Chapter (MAC) Chapter Meeting, October 19–21, 2020; Midcontinental Chapter (MCMLA)/Midwest Chapter (MWMLA) Chapter Meeting, October 14–16, 2020; New York-New Jersey (NY-NJ) Chapter Meeting, October 15–16, 2020; North Atlantic Health Sciences Libraries (NAHSL) Chapter Meeting, October 26–27, 2020; Philadelphia Regional Chapter Meeting, August 6–7, 2020; South Central Chapter Meeting, October 25–29, 2020; Southern Chapter Meeting, November 16–20, 2020.

## OPEN FORUMS

There were ten open forums held virtually prior to and after the vConference: Communities, May 26, 2020; Diversity and Inclusion, June 2, 2020; Annual Meeting Innovation, June 9, 2020; Education, June 16, 2020; Finances, June 24, 2020; Publications, June 30, 2020; Rising Stars, July 16, 2020; 2019 Research Training Institute (RTI) Fellows, August 5, 2020; MLA '21 Annual Meeting Invitation, September 15; Authors, October 6, 2020.

## NATIONAL LIBRARY OF MEDICINE UPDATE

The National Library of Medicine (NLM) Update took place on Friday, August 14, 2020, from 10:15 a.m.–11:30 a.m. This was followed with “Continue the Conversation,” from 11:30 a.m.–noon with NLM Director Patricia Flatley Brennan.

## LEGISLATIVE UPDATE

“Advocate for Medical Libraries with MLA! Professional Advocacy for Health Sciences Librarians,” was held Monday, August 10, 2020, 10:15 a.m.–11:30 a.m.

## OTHER SPECIAL EVENTS AND RECEPTIONS

### Monday, July 27, 2020

- MLA New Members/First-Time Attendees (NMFT) Program, 3:00 p.m.–4:30 p.m.

### Tuesday, July 28, 2020

- Introduction to Open Science (on-demand), 10:30 a.m.–11:30 a.m.

### Monday, August 10, 2020

- PubMed Update: Monday's Video Chat, noon–1:00 p.m.
- Meet and Greet with Esther Choo, 6:00 p.m.–6:30 p.m.

### Tuesday, August 11, 2020

- PubMed Update: Tuesday's Video Chat, 1:00 p.m. –2:00 p.m.

### Wednesday, August 12, 2020

- PubMed Update: Wednesday's Video Chat, 2:00 p.m.–3:00 p.m.
- Meet and Greet with Chris Shaffer, 6:00 p.m.–7:00 p.m.
- NLM Trivia Night, 6:00 p.m.–7:00 p.m.

### Thursday, August 13, 2020

- PubMed Update: Thursday's Video Chat, noon–1:00 p.m.

### Friday, August 14, 2020

- Roles to Play: Open Science and Health Sciences Librarians, 11:30 a.m.–3:00 p.m.
- MLA '20 vConference Closing Session and performance by MLA '21 (Washington, DC) organizing committees, 3:15 p.m.–3:45 p.m.

## VIRTUAL EXHIBIT HALL AND EXHIBITOR SOLUTION SHOWCASES

The Virtual Exhibit Hall was home to more than sixty-five vendors who presented videos, downloaded product and education materials, and participated in public chat and private video sessions from August 10, 2020–August 13, 2020. Material was available for viewing for thirty days.

Exhibitors held Solution Showcases to provide information and to introduce new products and services. The following sessions were held.

### Monday, August 10, 2020

- Rittenhouse: Precision Collection Development: Patron Drive Acquisition (PDA) for R2
- Third Iron: Simplifying Access with LibKey When Searching Databases, Discovery Services, and the Open Web
- Wiley: Cochrane “Inside/Outside”: An Update from Carol Lefebvre

### Tuesday, August 11, 2020

- American Psychological Association: American Psychological Association Seminar
- Clarivate: Preview the New EndNote Experience
- Evidence Partners: How COVID-19 Revolutionized Literature Review Methodology

### Wednesday, August 12, 2020

- Elsevier: Spotlight on Research
- McGraw-Hill: Informed Care Decisions with McGraw-Hill's New Hematology/Oncology Solution
- Springer Nature: Artificial Intelligence in Health Research: The Basics

### Thursday, August 13, 2020

* Clarivate Web of Science: Tools to Accelerate Drug Discoveries: COVID-19 and Beyond
* Thieme: Thieme's MedOne Platforms
* Wolters Kluwer: So What Do Publishers Actually Do?

### Friday, August 14, 2020

- ECRI: How ECRI Guidelines Trust (EGT) Empowers Medical Librarians to Succeed
- National Library of Medicine: PubMed Central: Expanding Discovery of Preprints

## CONTINUING EDUCATION COURSES

There were no continuing education courses offered during the MLA '20 vConference.

## RESOURCES AND SERVICES

The online itinerary planner, sponsored by Wolters Kluwer, allowed attendees to peruse programs and events online. Live streaming was available on Twitter using the hashtag #MLANET20. The annual meeting blog posts are available on the MLA website. The MLA Professional Recruitment and Retention Committee (PRRC) sponsored the MLA '20 Virtual Resume Clinic after the meeting, during the week of August 23, 2020. The Virtual Hall of Exhibits was available on-demand from July 27–August 28, 2020; live symposia were held August 10–14, 2020; and live video chats were held August 10–13, 2020.

## SUPPLEMENTAL FILES

**Appendix:** MLA '20 Program Session Abstracts

**Appendix:** MLA '20 Poster Session Abstracts

