# Supplementary material for: 120th Virtual Annual Meeting and Conference: Medical Library Association, Inc. May 19, 2020, and July 27–August 28, 2020
Source: J Med Libr Assoc. 2021 Jan 1;109(1):E1–E40. doi: 10.5195/jmla.2021.1169 (PMC7781040; doi:10.5195/jmla.2021.1169)
Supplement: Supplementary file 1 — Appendix: MLA '20 Program Session Abstracts [file jmla-109-1-E1-s01.pdf]

# Medical Library Association

# MLA '20 vConference Immersion Session, Lightning Talk, and Paper Abstracts

MLA
2020
VISION
THE FUTURE
IN FOCUS
vConference & Exhibits
AUGUST 2020
MLANET.ORG #mlanet20

Any unsolicited abstracts for the annual meeting undergo a process of blind peer review. Abstracts of papers intended for presentation are reviewed by members of a panel of reviewers sponsoring the programs. The final decision on program speakers rests with the program planners.

Tuesday, July 28, 10: 30 a.m.–4: 30 p.m.

# Introduction to Open Science (On-demand)

## Introduction to Open Science (On-demand)

**Lisa Federer, AHIP,** Data Science and Open Science Librarian, National Library of Medicine, North Bethesda, Maryland

Researchers are beginning to embrace open, transparent practices, and funders and journals increasingly require research outputs to be publicly available. But what exactly is open science, and why does it matter to librarians? This webinar will provide an introduction to the broad range of activities and practices that fall under the umbrella of open science and how these practices help increase research transparency, enable scientific reproducibility, and expand access to crucial information.

Attendees will learn how they can help bring open science to their institutions and gain a foundation for the open science session to be held on August 14. The on-demand presentation length is approximately 40 minutes.

Click the "Join here" button to view Introduction to Open Science (On-demand) with closed captioning.

Monday, August 10, 10: 15 a.m.–11: 30 a.m.

---

# Information Management Immersion Session 5

## The FAIRest of Them All: Using the FAIR Data Principles to Evaluate Open Data Repositories

**Peace Ossom Williamson, AHIP,** Director for Research Data Services, University of Texas at Arlington, Arlington, Texas

**Virginia (Ginny) Pannabecker, AHIP,** Director, Research Collaboration & Engagement; Life Sci & Health Sci Liaison, University Libraries, Virginia Tech, Blacksburg, Virginia

**Session Format:** An interactive workshop introducing FAIR principles, including hands-on group discussions and activities where participants will share their approaches and work together using guiding questions to find and assess a data repository and reflect on using FAIR principles to support open data sharing.

**Objective:** Come to this interactive workshop to learn how to apply FAIR Data Principles (2016) - which were created around a set of 14 metrics to evaluate and quantify the features of open data, specifically the aspects of: (F) findability, (A) accessibility, (I) interoperability, and (R) reusability - followed by hands-on activities where participants will share their approaches and work together using guiding questions to find and assess a data repository and reflect on using FAIR principles to support open data sharing in your work setting. As funders and publishers are increasingly encouraging and requiring researchers to publish their data, librarians have stepped in to provide expertise around choosing where and how to best prepare, deposit, and publish datasets. This session will provide a pathway for librarians to identify and critically appraise existing data repositories in accordance with the FAIR principles in order to provide guidance on where to deposit data, and how to make data meet quality standards upon publication -- see the detailed session agenda at: https: //tinyurl.com/FAIRestData-MLASessionAgenda.

**Instructional Methods:** The presenters will introduce the FAIR Data Principles and facilitate a discussion where participants share their current practice. Next, participants will receive a scenario: Someone comes to you and wants to deposit a dataset on [….participants will receive different topic or other focused example here…], and they will then work in groups to evaluate a provided short list of 1 or 2 topic-relevant repositories by applying the FAIR Principles to evaluate the repository/ies as a whole (including possibly assessing one of the datasets published within the repositories), ultimately working to determine whether they would recommend the repository/ies to the researchers to use to deposit their data. Afterwards, all of the participants will discuss what they found and one or two takeaways about what they’ll bring back to their institution or daily work from what they discussed and discovered during the session. A resource folder will be provided with materials from the session in openly licensed, editable formats for participants to adapt and repurpose for training or workshops of their own.

**Participant Engagement:** Throughout the session, the presenters will encourage brainstorming and sharing of techniques and sources for finding datasets or repositories, hands-on use of FAIR principles to evaluate datasets and repositories, and a concluding discussion on how participants will use what they explored in the future.

**Sponsors:** PH/HA Caucus and Data Caucus

Monday, August 10, 10: 15 a.m.–11: 30 a.m.

---

# Professionalism & Leadership Immersion Session 4

## Advocate for Medical Libraries with MLA! Professional Advocacy for Health Sciences Librarians

**Margaret Ansell, AHIP,** Associate Chair, Health Science Center Libraries, Gainesville, Florida

**Sandra L. Bandy, AHIP,** Assistant Director for Content Management, Augusta University, Evans, Georgia

**Mary M. Langman,** Director, Information Issues & Policy, Medical Library Association, Chicago

**Teresa Zayas Caban,** Chief Scientist, US Department of Health and Human Services

**Rebecca Goodwin,** Policy Analyst and Open Science Specialist, NLM

**Session Format:** This session will begin with a 15 minute presentation of MLA’s legislative priorities, followed by a panel discussion, Q&A, and two breakout sessions where members will begin addressing strategies for preparing for MLA’s 2021 Capitol Hill Day.

**Description:** Have you been interested in advocating for access to quality health information with your elected officials, but don’t know where to start? Join this immersion session to learn about MLA’s Advocacy Program and the work it does, and participate in small group facilitated discussions about how to engage with your local, state, and national representatives about issues related to medical librarianship and health information.

**Methods:** The session will open with a presentation highlighting how public policy impacts health sciences libraries and issues and policies you will want to know more about to become a strong advocate. Issues that impact health sciences libraries including funding for the National Library of Medicine and National Institutes of Health, and public access to government-funded health information will be discussed. This will be followed by a panel discussion led by members of the Governmental Relations Committee and Joint MLA/AAHSL Legislation Committee. The panel will describe their experiences preparing for and meeting with Congressional representatives. Techniques for having successful meetings will be discussed and links provided to suggested reading materials for keeping informed about both legislative issues and the impact of medical libraries on constituents.

**Participant Engagement:** Participants will be invited to enter breakout rooms to ask questions and engage presenters and each other on topics of interest after the presentation of legislative priorities and throughout the panel and Q&A. Questions and comments will be invited both in person and through the conference mobile app.

**Sponsors:** Governmental Relations Committee and Joint MLA/AAHSL Legislation Committee

Monday, August 10, 11: 30 a.m.–3: 00 p.m.

# Clinical Support Session 1

## Moral Distress Related to Ethical Dilemmas among Consumer Health Information Librarians

**Robin O'Hanlon,** Associate Librarian, User Services, Memorial Sloan Kettering Cancer Center, New York, New York

**Katelyn Angell,** Reference and Instruction Librarian, LIU Brooklyn, Brooklyn , New York

**Samantha Walsh,** Manager of Information & Education Services, Levy Library, Icahn School of Medicine at Mount Sinai, Astoria, New York

**Objectives**: In recent years, moral distress has become a topic of interest among health professionals. Moral distress is most commonly described in the nursing literature, and refers to a situation wherein an individual knows the correct action to take, but is constrained from doing so. While moral distress differs from the classic ethical dilemma, in recent years practitioners and theorists have advocated for a broadening of the definition of moral distress. To date, no study has examined another group of individuals who frequently interact with patients and who may be constrained by the confines of their role - consumer health information professionals. The objective of this study was to determine if consumer health information professionals experience ethical dilemmas and/or moral distress, and to determine what, if any, coping strategies these individuals have developed to manage their distress.

**Methods**: This study employed a mixed methods approach. Quantitative data were gathered via an online survey which was distributed to relevant consumer health information professional listservs. The survey contained demographic questions and a series of questions related to potential distress within the context of work as a consumer health information professional. Qualitative data were also gathered through phone interviews with consumer health information professionals. Interview questions included the interview subject’s definition of moral distress, professional experiences with moral distress, and any coping strategies to manage said distress.

**Results**: The authors received 213 survey responses. To test whether any of our demographic variables help to explain survey response, we used STATA to calculate Pearson correlation coefficients. Individuals who were more likely to experience distress in their occupation as consumer health information professionals included individuals with less experience and individuals who identified as Black and Latinx. Interview data indicated that subjects most commonly experienced ethical dilemmas related to censorship, providing prognosis information, and feeling constrained by institutional policies. Few interview subjects described scenarios that reflected moral distress.

**Conclusions**: Consumer health information professionals do not appear to experience moral distress, at least according to its most narrow definition. It appears that consumer health information professionals may confuse moral distress and ethical dilemmas, or conflate the two. However, consumer health information professionals do consistently experience distinct ethical dilemmas, and the most durable patterns of this distress appears to be related to experience and racial identity. Further study is needed to determine how to best address the impacts of distress caused by ethical dilemmas among these groups.

## Aligning Our Vision with Dental Competencies to Improve Evidence-Based Dentistry Education

**Nena Schvaneveldt, AHIP,** Education Librarian, University of Utah, Salt Lake City, Utah

**Sean Stone,,** Dentistry Librarian, Indiana University School of Dentistry, Indianapolis, Indiana

**Natalie Clairoux,** Dentistry Librarian, Universite de Montreal, Beaconsfield, Quebec, Canada

**Amanda Nevius,** Research & Instruction Librarian - Dental Liaison, Tufts University, Boston, Massachusetts

**Lorraine Porcello,** Branch Librarian, Basil G. Bibby Library, Eastman Institute for Oral Health, Rochester, New York

**Erica R. Brody,** Research and Education Librarian, Virginia Commonwealth University, Richmond, Virginia

**Irene (Rena) Machowa Lubker, AHIP,** Research and Education Informationist, Medical University of South Carolina, Charleston, South Carolina

**Jennifer S. Walker,** Health Sciences Librarian & Liaison to the Adams School of Dentistry, University of North Carolina at Chapel Hill, Chapel Hill, North Carolina

**Background**: Evidence-based practice requires dental student proficiency in (a) asking clear researchable questions, (b) acquiring evidence, and (c) critically appraising research studies. Librarians are well-suited to instruct on these topics due to their expertise and interest in information literacy; however, they are not consistently consulted or invited to teach dental curricula. The development of a dentistry-specific companion document to the Association of College and Research Libraries (ACRL) Framework for Information Literacy would uphold librarian collaboration in dental instruction by demonstrating the precise ways in which librarians’ and dental educators' values and objectives are aligned.

**Description**: Dental librarians from across the United States and Canada first identified 9 American and Canadian dental education competencies with major information literacy and/or critical thinking components. Next, these competencies were individually aligned to frames in the ACRL Framework for Information Literacy. When no overall consensus was found, the mapping was further discussed by small groups until an agreement was reached. These combined competencies and frames were then used to create a rubric with a detailed picture of the levels of critical thinking skills dental students will need at specific milestones in their education. The rubric will be presented to dental faculty and professional associations for feedback and validation. Information literacy learning outcomes were also devised for each dental education competency using Bloom’s Taxonomy of Action Verbs and the ACRL Framework’s knowledge practices and dispositions.

**Conclusion**: Complex concepts, such as critical thinking and evidence-based dentistry, are often best taught in collaboration, and the resulting companion document (including the mapping and a rubric to determine student proficiency) will be a tool that can be used to encourage dental school faculty to collaborate with librarians at the programmatic and course level. Librarians in any subject area may also gain inspiration on how to create and use such materials with their faculty as well.

## Supporting Dietetic Interns with Evidence-Based Practice (EBP)

**Irene (Rena) Machowa Lubker, AHIP,** Research and Education Informationist, Medical University of South Carolina, Charleston, South Carolina

**Amanda Davis,** Sr. Evidence-Based Practice Analyst, Medical University of South Carolina, Charleston, South Carolina

**Background**: To be eligible to sit for the dietetic registration exam, students much complete a comprehensive, clinical and community-based dietetic internship to prepare them with baseline competencies for dietetics. All dietetic interns must complete a research Capstone as part of their training. Evidence-based practice (EBP) is a core competency of both the internship and the research Capstone. An EBP course was developed as part of the orientation to the dietetic internship. The goal of the course was to promote the development of more comprehensive, evidence-based Capstone projects and improve use of EBP during rotations.

**Description**: A librarian and a clinical EBP analyst, both with dietetics and Public Health training and experience, teamed up to create an EBP course for dietetic interns (n=12) at our institution. It included two in-person sessions completed six weeks apart and five online, video-based modules with corresponding quizzes delivered through a Libguide. Knowledge was assessed via a 10-question pre-test at the first in-person session and again six weeks later. Pre- and post-test questions were identical, and focused on PICO, searching, critical appraisal, and the EBP process overall. LibGuide analytics were collected to evaluate interaction with each module. The median (75 pre vs 90 post, mean difference: +15) and mean (75 pre vs 87.5 post, mean difference: +12.5) knowledge scores for dietetic interns in our study increased as a result of the EBP course, but these increases were not statistically significant (p=0.054).

**Conclusion**: This course for dietetic interns was successful at improving mean and median knowledge scores about the EBP process steps, although statistical significance was not reached. These results are limited by the small sample size. We intend to collect similar data with the next cohort of dietetic interns and re-evaluate the results. The analytic feedback provided many opportunities for improvement by the instructors and internship director in preparation for next year’s cohort of dietetic interns.

Monday, August 10, 11: 30 a.m.–3: 00 p.m.

---

# Education Session 1

## Focusing on Support: Using an Existing Shared Customer Service Platform to Provide Technical Instruction to Health Sciences Librarians

**Marina Aiello,** Lead Librarian, Instructional Design and Technology, Kaiser Permanente, Stockton, California

**Marie Beckermann, AHIP,** Assistant Librarian, Kaiser Permanente South Bay Medical Center, Harbor City, California

**Eve Melton, AHIP,** Regional Director Library Services NCAL, Kaiser Permanente, Stockton, California

**Sara Pimental, AHIP,** Senior Consultant, Kaiser Permanente, Oakland, California

**Joy Rodriguez,** Medical Librarian, Kaiser Permanente Fresno Medical Center, Fresno, California

**Melissa A. Spangenberg,** Manager Library Services, Kaiser Permanente, Sacramento, California

**Background**: Kaiser Permanente Librarians oversee several websites, over 20,000 e-resources, and 25 databases, providing clinicians with easy access to information needed for patient care. These websites and e-resources did not have a shared internal ticketing system where library staff could submit questions regarding technical issues. As a result, library technical administrators received questions in multiple ways: instant message, email and phone. Tracking technical issues was extremely difficult, resolutions were sometimes delayed, and occasionally, questions would be lost. Utilizing an existing shared customer service platform enabled library staff to submit tickets for themselves and for their patrons.

**Description**: The internal ticketing system was created to communicate technical issues and resolutions to 32 librarians covering 39 hospitals and 690 clinics over the continental US and Hawaii. An inter-regional committee was formed to establish best practices for design, metrics, and information collection. An email address and web-based form were made available for questions and issues noted by staff. Live and asynchronous training was created for staff as well as frequently asked questions (FAQs) and job aids for librarian self-service. The internal system gave administrators the ability to assign the issue to the correct person, track the progress to resolution, as well as allow librarians to consult with one another for support. The system also helped the team identify when new or updated technical documentation was needed. Evaluation included FAQ growth, surveying librarian satisfaction, and deep analysis of usage metrics.

**Conclusion**: Using an existing customer service platform to deliver technical help to health sciences librarians is an innovative way of addressing the common challenge of providing ongoing support to a library team. Benefits of this system include the provision of just-in-time training, organization and tracking of ongoing issues, easy access to troubleshooting solutions, and robust metrics on the team’s technical support needs. Library technical administrators and health sciences librarians alike will appreciate access to up-to-date technical documentation, asynchronous and multimodal instruction options, as well as the collaborative nature of the platform for both asking and answering questions.

## Creating Bright Futures: Preparing Students for Research with a Longitudinal Evidence-Based Dentistry Project

**Kathryn Houk, AHIP,** Community Engagement & Health Literacy Librarian, University of Nevada Las Vegas, Las Vegas, Nevada

**Background**: First-year dental students in their spring 1-credit seminar were assigned a redesigned, longitudinal PICO project in order to better prepare them to practice with an evidence-based dentistry (EBD) mindset. Previous iterations of the assignment led to disappointing outcomes for students in terms of work distribution among their teams, librarian contact, final presentation quality, and understanding of the research process. The class and project are valued by the faculty as an introduction to EBD, as well as practice for a second year course that requires students to undertake laboratory research leading to a publication or presentation with a literature search.

**Description**: Dental faculty approached the librarian with an opportunity to present within their dental course titled, The American Dental Student, in the spring of 2019. The final project and the limitations seen in previous years were discussed. Incorporating dental faculty suggestions, the librarian developed a new longitudinal and integrated structure for the project. The redesign included five bi-weekly assignments with responsibilities for each group member, grading rubrics for assignments and the final presentation, explicit guidelines outlining expectations for assignments and the final project, and two 50-minute in-class presentations. The reimagined project addressed several Commission on Dental Accreditation (CODA) standards, and all assignments required reflection on the process of developing the search and finding evidence. The bi-weekly assignment structure and reflective nature of the questions encouraged more interaction among group members and frequent meetings with the librarian for clarification and assistance.

**Conclusion**: Short term outcomes include increased contact hours with the librarian, continued work on the project throughout the semester, and dramatically improved final presentations according to faculty graders who attended previous years' presentations. Long term outcomes have yet to be observed, but it is hoped that there will also be improved understanding of the research process and higher quality final products in the second year research course. Student evaluations of the librarian's classes were generally positive, with verbal feedback indicating many students discovering and working through key concepts and roadblocks in information literacy and application of the EBD process.

## Increasing Student Engagement Using an *Amazing Race*–Style Competition

**Emily Gorman, AHIP,** Research, Education & Outreach Librarian, University of Maryland, Baltimore, Baltimore, Maryland

**Background**: Gamification is an increasingly popular method for enhancing student engagement during instruction. Given its success in a variety of educational settings, this concept created the basis of a new learning game for first year pharmacy students. Based on the reality TV show “The Amazing Race,” the activity introduces students to library and drug information resources. The purpose of this program was to increase student engagement in a library orientation session, which had previously been delivered as a traditional lecture-style presentation.

**Description**: “The Amazing Race: Drug Information Edition” was inspired by a popular TV competition involving “legs” where contestants have to complete tasks before receiving clues to the next phase. The librarian developed five legs, each with a series of questions that prompted teams of students to use specific library resources to find the answers. The first implementation in Fall 2018 involved 132 students (split into two groups to make the activity more manageable). Course

instructors and teaching assistants were responsible for checking answers and passing out the question sheets for each leg, while the librarian answered questions and kept track of teams’ progress. The first three teams to finish the race received small prizes. At the end of the class the students completed a brief evaluation form. The activity was modified for Fall 2019 based on student and facilitator feedback.

**Results**: There were 112 responses to the 2018 evaluation, and 82 in 2019. The majority of students in both years indicated that they learned something new during the activity (93.6 % in 2018, 95.1% in 2019). Asked about the overall effectiveness of the class, most students rated it “Good” or “Excellent” (74.8% in 2018, 81.7% in 2019). Responses to open-ended questions contained a larger number of positive comments than negative ones.

**Conclusions**: Overall the reaction to this new activity was encouraging, with students in both years expressing that the activity was fun and engaging. Challenges included questions that had too many possible answers or were unclear in some way. The results suggest that the modifications made for the second implementation were successful in improving the effectiveness of the class. The session has also strengthened the librarian’s relationship with course faculty, which has led to additional teaching opportunities.

## Impact of a Consumer Health Information Specialization (CHIS) Sponsorship Program on the Capacity of Public Library Staff to Provide Health Information to Their Community

**Elizabeth J. Kiscaden, AHIP,** Medical Librarian, University of Iowa, Iowa City, Iowa

**Molly Knapp, AHIP,** Training Development Specialist, NNLM Training Office, Houston, Texas

**Bobbi Newman,** Community Outreach & Engagement Specialist, National Network of Libraries of Medicine - GMR, Iowa City, Iowa

**Erica Lake, AHIP,** Research & Evaluation Specialist, Essentia Health, Duluth, Minnesota

**Michele Spatz,** Community Engagement Coordinator, NNLM Pacific Northwest Region, Seattle, Washington

**Susan M. Wolfe,** Evaluation Specialist, NNLM National Evaluation Office, Cedar Hill, Texas

**Objectives**: Does a CHIS sponsorship program improve the capacity of public library staff to provide health information to their community?

**Methods**: In 2018, the National Network of Libraries of Medicine (NNLM) launched a national sponsorship program to support U.S. library staff in obtaining the Medical Library Association (MLA) Consumer Health Information Specialization (CHIS). To evaluate the impact of the sponsorship program, staff developed a 16-question assessment which was administered to 224 library staff sponsored in the first year of the program. Of the 224 library staff contacted, 126 respondents completed the assessment, a response rate of over 56%. The assessment included questions mapped to competencies for Level I of the CHIS in order to assess behaviors, attitudes, and confidence of library staff who received the specialization. The assessment also examined the value of the specialization and any novel programming or library impact that emerged as a result of receiving the specialization.

**Results**: For each of the competencies assessed, 80% or more of respondents reported an increase in knowledge. Data indicated that knowledge gained was actionable, with 50% of respondents offered new health information programs, over 70% began offering health information services to patrons,

and over 60% weeded or updated their library’s consumer health resources. All respondents indicated that obtaining the specialization met their expectations, but renewing their certificate or obtaining the Level II specialization was not certain. Only 65% of respondents plan to renew, while 33% were unsure and 2% are not planning to renew.

**Conclusions**: This research indicates that sponsorship of the certificate program was successful in increasing the capacity of public library staff to provide health information to their community. The NNLM sponsorship of CHIS resulted in an increase in knowledge surrounding the competencies of the program. Data indicates that knowledge gained was actionable and put into practice by public library staff sponsored through the program. Comments submitted by respondents will be shared with the Medical Library Association and used to streamline the sponsorship process assist in marketing the program.

Monday, August 10, 11: 30 a.m.–3: 00 p.m.

# Education Session 2

## Bringing the Medical Library to the Forefront of Data Analysis and Precision Medicine: Strategies and Statistics

**Nur-Taz Rahman,** Simbonis Fellow in Bioinformatics, Yale University, Cushing/Whitney Medical Library, New Haven, Connecticut

**Sawyer Newman,** Data Librarian for the Health Sciences, Yale University Cushing Whitney Medical Library, New Haven, Connecticut

**Rolando Garcia-Milian, AHIP,** Bioinformatics Support/ Lecturer in Epidemiology, Cushing/Whitney Medical Library/ Yale University, New Haven, Connecticut

**Background**: Currently, biomedical research relies on Bioinformatic data to find causes of diseases, develop precision medicine, track treatment, and predict prognosis. Laboratory and clinical investigators produce large amounts of data that need to be computationally analyzed. However, investigators often lack the skills and/or time required for such analysis, resulting in slow processing that leads to frustration and missed opportunities. The support for investigators with Bioinformatic data is sparse, and the Data Services for the Health Sciences and the Bioinformatics Support Program at the Cushing/Whitney Medical Library (CWML) at Yale University is working together to fill this unmet need.

**Description**: The CWML recruited Data Services and Bioinformatics Support personnel to support investigators in data management during the research life-cycle and data analysis, respectively. By working together, these two separate entities have been able to provide complete computational analysis support to the investigators. Through individual consultations, demonstration-based instructional sessions, and direct collaborations, this program has been able to speed-up bioinformatic research with licensed analysis software; improve investigators’ knowledge of data analysis in programming languages such as R and Python; and ensure best data practices during research. Evaluation on the effectiveness of the program is being carried out via (1) assessments after instructional sessions to understand whether learning goals have been met; and (2) surveys after a period of consultations to understand whether the investigator’s research has progressed.

**Conclusion**: At the time of our presentation we will provide statistics and real-life examples to illustrate that Data Services and Bioinformatics Support can successfully work together to provide high quality instructional sessions on data analysis. These sessions can help patrons learn to execute their own analyses in computing languages, such as R, even if they do not have extensive prior knowledge in this area. We have shown that not only are these sessions extremely popular within the medical community, but they are also of interest to a variety of clinical departments, therefore, enabling the medical library to extend support to the entire medical school research community.

## The Accidental Academic Library: Meeting the Needs of a Health System–Affiliated University

**Heather J. Martin, AHIP,** Director, System Library Services, Providence, Portland, Oregon

**Background**: In 2017 a small liberal arts college affiliated with a large western health system was re-branded, and a School of Health Professions was created to prepare for future staffing shortages and

to train the next generation of healthcare workers. Instead of relying solely upon its own library, University administration reached out to the health system library to meet the information needs of this new group of students, most of whom were active employees of the healthcare enterprise. Thus, a group of hospital librarians found themselves as accidental academic librarians.

**Description**: Library leadership researched academic library bench-marking to propose a budget for collections and FTE. Library staff brushed up on library trends and copyright issues specific to academic libraries and prepared to serve a new type of user. Vendor negotiations became newly complex as they brought in both the corporate and academic sides of the business and raised the issue of how to properly license for the University. Operational challenges included subscription IP overlaps and how to provide remote access to non-employed students and faculty. Librarians had to adjust to the different service level needs for students as compared to employees, many of whom were one and the same.

**Conclusion**: Nearly 3 years later and the health system library has yet to receive any additional funding or FTE to support the University and has built the collection and services on its existing budget. Publisher contracts are revisited on an annual basis as the growth of the school of health professions puts pressure on agreed-upon pricing models, as well as library staffing capacity. Library staff has adapted instructional materials and service expectations to the student population, shifting the balance from a value-add "fish FOR a person" to a "TEACH to fish" approach.

## The Library in Focus: Active Orientations for Future Physicians

**Kathryn Houk, AHIP,** Community Engagement & Health Literacy Librarian, University of Nevada Las Vegas, Las Vegas, Nevada

**Courtney Brombosz,** Research and Education Librarian, Harvey Cushing/John Hay Whitney Medical Library

**Background**: The library is granted little to no formal time in the first-year medical students’ orientation, even though we have a significant footprint in their learning space and contain primarily digital collections. Due to discoverability issues for digital collections and lack of formal marketing outlets for services, the library feels it is crucial to interact with our patrons in person when they first arrive. For the past two years, the library has created an orientation event during breaks in the first-year students’ schedule; incorporating activity stations, vendor gifts, food and prizes to encourage participation.

**Description**: The library kept in mind a few key factors when designing and updating the orientation sessions: the importance of in-person interaction with students, the importance of external motivators for students to voluntarily participate, and how to build internal motivation through creating interesting and useful activities. External motivators included providing food, vendor gifts, library promotional gifts and the chance to win a grand prize upon completion of their orientation passport. Internal motivation factors included a welcoming atmosphere, challenging activities, and a sense of competition. Each year of orientation the had full-time staff and student workers moderate four activity stations focused on People & Services, Online Resources, Library IT, and Circulation. In our second year, we updated all paper and pen activities such as crosswords and matching games to also include our emerging technology, particularly the interactive Google JamBoard.

**Conclusion**: Program outcomes include high rates of participation from students, with 40% of 120 students completing passports the first year and 75% of 60 students completing them in the second year. Working closely with the School of Medicine allowed the orientation to be listed as an official activity on students’ calendars. This likely led to increased participation in the second year. Informal

observation and debriefing with library employees revealed students seemed engaged and learned new information about library policies, procedures, and resources. Headcounts have not been taken, but we hope to develop a process for capturing general attendance during future orientations.

## Building Users’ Search Skills for Systematic Reviews: Development of Self-Directed Learning through Qualitative Synthesis of Guidelines

**Jeffery L. Loo,** Clinical Librarian, UC San Diego Library, La Jolla, California

**Background**: When conducting a systematic review, the search for evidence can be a challenging process for novice searchers. There are complex procedures with multiple sources of guidelines and the prevailing instruction is targeted at intermediate and higher skill levels. To address these challenges, this project created self-instruction materials framed along an explicit search workflow. This instruction was developed through qualitative content analysis of four major systematic review guidelines. The result is a comprehensive yet straightforward self-instruction guide for advanced literature search skills. This paper reports the development methodology and observations from the guide’s use in reference consultations.

**Description**: Instructional development began with the qualitative content analysis of search guidelines by four organizations (Cochrane, NAM, AHRQ, and CRD). Over 300 recommended search objectives and tasks were extracted, with many duplications across the guidelines. This analysis defined the phases of a comprehensive search workflow and synthesized recommendations into search tasks and stepwise procedures. The workflow has five phases addressing search strategy design, search conduct, results management, document retrieval, and search reporting. Additionally, a directory of 150+ recommended databases was compiled. The resulting instructional guide is assigned as preparatory reading before a reference consultation, serves as a discussion framework during the session, and functions as a reference tool afterwards. The guide was evaluated through librarian peer review and user feedback.

**Conclusion**: The self-instruction guide supports the UC San Diego Library’s systematic review service. It is situated in the consultation process as pre-session reading, a discussion framework, and post-reference support. Anecdotal evidence indicates the guide may prompt a user-driven consultation and may facilitate instruction on advanced literature searching. Because the guide synthesizes multiple guidelines, it has potential to standardize library services for systematic reviews. Future evaluation would assess the guide’s pedagogical usability. The guide is publicly available for re-use and customization.

Monday, August 10, 11: 30 a.m.–3: 00 p.m.

# Global Health & Health Equity Session 1

## Accessing Evidence-Based Resources and Conducting Systematic Reviews in Resource-Limited Settings

**Karin Saric,** Information Services Librarian, USC, Los Angeles, California

**Sarah Young,** Liaison Librarian, Carnegie Mellon University, Pittsburgh, Pennsylvania

**Erin RB Eldermire,** Head, Flower-Sprecher Veterinary Library, Cornell University Library, Ithaca, New York

**Masimba Clyde Muziringa,** Deputy Librarian -CHS University of Zimbabwe, University of Zimbabwe, Harare, Zimbabwe

**Israel M. Dabengwa,** Assistant Librarian, National University of Science and Technology, Bulawayo, Zimbabwe

**Background**: We aim to report the outcomes of a 5-day Hinari and systematic review (SR) training workshop in CITY, COUNTRY, and a workshop series continued via web-conferencing. This training served as a pilot to develop a robust training model that provides both training in accessing high quality literature to support evidence-based practice (EBP), and the use and synthesis of evidence to promote publishing of systematic reviews from researchers and librarians in resource limited settings.

**Description**: Methodological and domain expertise are required for established methods for the comprehensive gathering and synthesizing of research. Resource-limited settings require access and the ability to use resources such as those available via Hinari - the World Health Organization program that provides free/low cost access to peer-reviewed resources to developing countries. Due to financial challenges over the last decade, there have been limited opportunities for such specialized training sessions for healthcare professionals in COUNTRY.

To extend previous workshops that focused separately on access to resources and conducting SRs, the authors held a holistic 5-day Hinari/SR training, followed by on-going webinars to support workshop participants as they continued to develop SRs. This paper will discuss instructional content, survey outcomes, and how the two parts of the workshops were aligned to support the development of SRs in resource-limited environments.

**Conclusion**: Forty health professionals were trained including researchers, clinicians, faculty, and librarians. Post-workshop survey results provide evidence that the workshop was well-received, with the majority of participants rating all modules to be relevant and useful. Results of a 6-month follow up survey will be included as well.

## Partnering with Community-Based Organizations to Provide Health Information Outreach in Public Libraries

**Kate Flewelling,** Executive Director, National Network of Libraries of Medicine, Middle Atlantic Region, Pittsburgh, Pennsylvania

**Michael Balkenhol**

**Renae Barger**

**Michelle L. Burda,** Network and Advocacy Coordinator, Health Sciences Library System, University of Pittsburgh, Pittsburgh, Pennsylvania

**Kelsey Cowles,** Academic Coordinator, National Network of Libraries of Medicine, Middle Atlantic Region, Pittsburgh, Pennsylvania

**Erin Seger**

**Tess Wilson**

**Tessa Zindren,** Program Manager, National Network of Libraries of Medicine, Middle Atlantic Region, Pittsburgh, Pennsylvania

**Susan M. Wolfe,** Evaluation Specialist, University of Washington, Cedar Hill, Texas

Background: While public libraries are an important partner in health literacy initiatives, public library staff are often hesitant to initiate health information programming and services. However, they are willing to host and market such programs. Community Based Organizations (CBOs) often have quality existing health programs that would appeal to diverse audiences. The purpose of this pilot project is to fund Community Based Organizations to incorporate MedlinePlus and information about the All of Us Research Program into existing health programming. Funded CBOs would then offer programs in public libraries within the Middle Atlantic Region.

Description: Under a partnership with the All of Us Research Program, the Network of the National Library of Medicine, Middle Atlantic Region (NNLM MAR), provided up to $20,000 to ten Community Based Organizations (CBOs) in Pennsylvania and New York. Funding was available for CBO staff time, travel, program materials and other program costs. Efforts were made to fund programming that had the potential to reach audiences frequently underrepresented in biomedical research. In order to be funded, CBOs needed to have existing high quality health programming that can be adapted for public library audiences and be able to complete in-person programs at public libraries in their area between October 2019 and April 2020. NNLM MAR staff worked with CBOs to integrate relevant health information into programs before they were delivered in public libraries.

Conclusion: This pilot program has two main goals. Goal 1 of this project is to place highly skilled health programming experts in public libraries to conduct no cost programs for public library audiences. Goal 2 is to determine whether funding CBOs to integrate health information resources into existing health programming in public libraries is a replicable model. Objectives and metrics have been established to evaluate the success of the project in meeting the two goals. Preliminary evaluation suggests both goals were met.

## LGBTQ+ Health Research Guides: A Content Analysis

**Gregg A. Stevens, AHIP,** Health Sciences Librarian, Stony Brook University, Stony Brook, New York

**Francisco Javier Fajardo, AHIP,** Information and Instruction Services Librarian, Florida International University, Miami, Florida

**Objectives**: Online research guides are an easy and effective tool to promote LGBTQ+ (lesbian, gay, bisexual, transgender, queer, and other) health information to health care providers and the public. Current literature recommends them for outreach and education. This study was designed to determine how extensive LGBTQ+ health guides are among hospital and academic libraries and which features are most common.

**Methods**: To conduct a content analysis, the authors sought out LGBTQ+ health guides in three ways. They searched the Springshare interface for LibGuides mentioning the word “health” and either “LGBT” or “transgender”. Secondly, the websites of AAHSL member libraries were searched for LGBTQ+ health guides. Finally, the websites of Canadian academic libraries were searched for LGBTQ+ health guides. For inclusion in the study, guides needed to consist of at least one complete page devoted to LGBTQ+ health. Only publicly accessible guides from academic libraries or hospitals were included. A content analysis was subsequently conducted to identify major characteristics of the LGBTQ+ research guides, such as target audience and the type of information provided.

**Results**: LGBTQ research guides were located for 74 institutions. Five of the 74 were hospital libraries and the remainder were academic libraries. Out of 158 AAHSL member libraries, 48 (30.4%) had LGBTQ+ guides. Just over half of the guides (56.8%) featured information targeted to health care providers, and a smaller percentage (37.8%) had consumer health information. Most guides (95.9% provided general LGBTQ+ health information, and the majority (87.8%) also featured information resources for transgender health. Smaller percentages of guides highlighted two other LGBTQ+ health specialties: HIV/AIDS information (48.6%) and women’s health (16.2%).

**Conclusions**: Despite recommendations in the published literature that health sciences libraries can provide outreach easily through online research guides, it would appear that the majority of libraries are missing a valuable opportunity by not maintaining LGBTQ+ health guides. Further research may be necessary to determine how successful existing guides are and what barriers are preventing more libraries from creating guides.

## How One Library’s Location Change Impacted Health Information Requests: Comparing Zip Codes and Health Disparities to Shape Library Services

**David W. Petersen, AHIP,** Research & Learning Services Librarian, University of Tennessee Graduate School of Medicine, Knoxville, Tennessee

**Kelsey Grabeel, AHIP,** Assistant Director, University of Tennessee, Knoxville, Tennessee

**Martha Earl, AHIP,** Director/Associate Professor, University of Tennessee, Knoxville, Tennessee

**Cameron Watson,** Library Associate III, Preston Medical Library, Knoxville, Tennessee

**Objectives**: To analyze the University of Tennessee's Preston Medical Library / Health Information Center library’s consumer health request database to evaluate the impact a library’s move inside of the hospital made in regards to reaching the 21 counties the hospital serves. Additionally, researchers assessed which counties and zip codes requested the most health information as well as the number of requests from zip codes with high poverty levels.

**Methods**: Researchers downloaded data from the Consumer and Patient Health Information System (CAPHIS) database, located on a hospital server. Data has been collected in this database since 1997. Requests are labeled with a form identification number, which allows for personal names to be deleted, thus helping to preserve anonymity. The request forms were sorted and reviewed by county and zip code. Researchers separated data into pre-move and post-move sets. Data will be inputted into Tableau to create maps, visually showing where the largest concentrations of patrons are located as well as associated poverty rates based on zip code within the hospital's 21 county service area.

**Results**: There were 3,141 health information requests from September 21, 2014 to May 31, 2019, representing a substantial increase over the old location. Ninety-nine results were omitted due to being out-of-state. The majority of requests were from Knox county and adjacent counties. Requests

were also received from counties not previously reached and counties with elevated poverty levels. Several local zip codes that had previously not been reached or had very few requests before the library's move now reported substantial use.

**Conclusions**: Collecting data on patron interactions is not only critical for institutional reporting, but also for community outreach. Understanding that data requires taking additional steps to ask precise questions, filter the information, assess local demographics, and provide quality data in a visual format for institutional representatives. Researchers anticipate being able to better tailor services to the community based on the results.

Monday, August 10, 11: 30 a.m.–3: 00 p.m.

# Information Management Session 1

## Development of a Policy on Ingestion of Human Subjects Data Sets: An Institutional Assessment and Large-Scale Repository Scan

**Shanda Hunt,** Public Health Library Liaison & Data Curation Specialist, University of Minnesota, Minneapolis, Minnesota

**Valerie Collins,** Digital Repositories and Records Archivist, University of Minnesota

**Alicia Hofelich Mohr,** Research Support Coordinator, University of Minnesota, Minneapolis

**Background**: The Data Repository for the University of Minnesota (DRUM) is a publicly available collection of digital research data generated by University of Minnesota researchers, students, and staff. Within DRUM, there are 43 datasets with human subjects data. A human subject is a living individual about whom a researcher obtains, uses, studies, analyzes, or generates information. Datasets published in DRUM are openly available, broadly disseminated, and downloadable without restriction. Because of the sensitive nature of human subjects data, and the potential issues with publishing such data in a publicly accessible repository, we assess all human subjects data before acceptance. Ongoing conversations with researchers and other campus units regarding human subjects data in DRUM have brought to light the need for an analysis of our own processes as well as a scan of other repositories’ practices and policies for the purpose of formalizing a human subjects policy for DRUM.

**Description**: We analyzed DRUM’s accepted and rejected human subjects datasets since 2013, in order to evaluate DRUM’s past and current practices regarding human subjects data, the ethical considerations we have weighed, and the actions we have taken in regards to these datasets. This presentation will provide specific examples of rejected datasets and the recommendations that curators made to researchers, and also detail the ethical considerations of publishing de-identified human subjects data when the study participants have not been notified. DRUM’s ongoing efforts to educate and establish campus-wide understanding of the issues led to the second aim of this study: we conducted a scan of 105 repositories that ingest human subjects data and analyzed 1) language related to participant consent on the website and 2) language related to participant consent in the deposit agreement. Finally, the presentation will detail the process of formulating a formal DRUM policy on human subjects dataset submissions based on the results of the analyses.

**Conclusion**: We’ll detail the outcome of the human subjects policy inventory and any progress made as a result of ongoing outreach efforts by DRUM curators at the University of Minnesota. We'll also discuss next steps in getting the new DRUM human subjects policy approved.

## Kickstarting Use of Electronic Lab Notebooks at an Academic Medical School

**Erin Diane Foster,** Research Data Management Program Service Lead, University of California, Berkeley, Berkeley, California

**Elizabeth C. Whipple, AHIP,** Assistant Director for Research and Translational Sciences, Indiana University School of Medicine, Indianapolis, Indiana

**Hannah J. Craven,** Research & Scholarly Communications Librarian, Indiana University School of Medicine, Indianapolis, Indiana

**Background**: As part of Indiana University School of Medicine’s effort to strengthen the research data management practices of its researchers, an electronic lab notebook (ELN) was licensed to improve the organization, security, and share-ability of data/information generated by the institution’s research labs and groups. The initial pilot of the ELN (i.e., LabArchives) at Indiana University School of Medicine involved 15 research labs and the medical library led implementation efforts on behalf of the School’s Office of Research Affairs. Beginning in April 2019, access to LabArchives was expanded beyond the pilot labs and made available to all faculty, students, and staff at IUSM as well as other IU campuses.

**Description**: Beyond the pilot phase, the medical library supports researchers using the electronic lab notebook by 1) delivering trainings that cover strategies for adopting an ELN and a hands-on demo of LabArchives, 2) scheduling one-on-one consults that mirror the structure of the training sessions, and 3) developing best practice guidance to assist in adoption of the ELN. The medical library communicates availability of LabArchives to faculty, students, and staff through presentations delivered at department meetings and write-ups in the institution's newsletter. The Data Services Librarian is the primary contact for this service; however, to make the service more sustainable, three other librarians are trained to deliver the training sessions and conduct individual consults. To assess the use of LabArchives and improve support moving forward, user surveys are distributed twice a year.

**Conclusion**: As of April 2020, there are 560 users of LabArchives at Indiana University. Ongoing challenges include: determining additional support beyond the existing trainings, defining “successful” adoption at the institution level, and further expanding this service in light of current events. By leading the development of this service, the library is more strongly integrated and visible in the research activities of IUSM, particularly as related to information and data management. Additional areas for research policy development were identified as a result of this work, especially related to data/information retention timelines and exit processes for research labs that depart Indiana University School of Medicine.

## Open Educational Resource Produces Licensed Resources to Sustain Project

**Nancy Lombardo,** Librarian, Head of Digital Publishing, University of Utah, Salt Lake City, Utah

**Background**: This program is intended for libraries and library faculty and staff involved in digital publishing, digital collection management, and large collaborations and partnerships. The project can serve as a model for others to publish non-traditional materials and gray literature, and to produce licensed products to support open educational resources.

1) use this model to provide publishing and peer-review services to partner organizations.

2) use academic promotion incentives to build volunteer participation in collaborative projects.

3) use existing digital collections to design unique licensed products that provide sustainable funding for projects.

**Description**: This health sciences library partnered with a national medical society to create an open access, discipline specific repository of peer-reviewed learning objects in many formats. The library is responsible for managing peer-review, technical processing of learning objects, maintaining databases, organizing, and disseminating the collections. The society members are responsible for creating content, serving on the editorial board, designing curriculum outlines,and general guidance. In order to fund the project, the partners have negotiated a contract with an electronic publisher to produce specific curricula as licensed products with royalties supporting the project. The project is

evaluated based on usage statistics, society member engagement, and revenue generated by spin-off products. Benefits of the project include teaching, research, and service credit toward academic advancement for all participants. Educating creators on maintaining copyrights is another important outcome.

**Conclusion**: The project has been successful due to incentives for volunteer participation. Society members receive credit for serving on committees of their national society, acting as section editors for the licensed products, and as authors of peer-reviewed learning objects in an online educational platform. The library serves as the publisher and receives funding to support the digital repository project. The licensed products provide finding to sustain the larger open educational resource. More than half of the users of the repository are international in origin, showing that there is a need for high quality teaching and learning platforms globally.

## Identifying Barriers to Citing Retracted Literature

**Elizabeth Suelzer, AHIP,** User Education and Reference Librarian, Medical College of Wisconsin, Milwaukee, Wisconsin

**Jennifer Deal,** Librarian Lead, Advocate Aurora Library Network, West Allis, Wisconsin

**Karen L. Hanus, AHIP,** Assistant Director, Collection Management and Resource Sharing, Medical College of Wisconsin, Milwaukee, Wisconsin

**Barbara E. Ruggeri, AHIP,** Life & Health Sciences Librarian, Carroll University, Waukesha, Wisconsin

**Elizabeth M. Witkowski,** Clinical Services Librarian, Children's Hospital of Wisconsin, Milwaukee, Wisconsin

**Objectives**: Recognizing that a paper has been retracted can be challenging due to differing indexing practices of journal publishers and citation databases, and a lack of guidance on citing retractions by citation styles. The purpose of this study is to document the retraction policies of various information providers and to highlight barriers to accurately citing retracted literature

**Methods**: We investigated the indexing policies of 50 journals that contain retracted publications. A search for the publication type “Retracted article” was conducted in PubMed on Oct. 20, 2019 and we limited to articles published in English since 2009. The 50 journals that contained the most retracted articles were chosen for analysis. The various journal publishers’ indexing practices for identifying retracted articles were documented in a table. Additionally, we investigated the current retraction policies of major citation databases that cover biomedical literature. If policies were not available online, the database companies were contacted directly. Finally, we identified commonly used citation styles and investigated their guidelines for documenting and citing retracted articles.

**Results**: Journals used a variety of methods for reporting a retracted article’s status. Of the 7 recommendations made by the ICMJE for reporting retracted publications, less than half (70 of 150) of the articles followed all recommendations. Journals were not consistent in labeling articles as retracted when reviewing three sample articles from the same journal. PubMed and Ovid MEDLINE showed the overall best performance in documenting retracted publications. When looking at the same publication in other databases, there were significant differences in how the retracted status appears.

The AMA, APA and NLM/Vancouver writing style guides include instructions for citing retracted literature.

**Conclusions**: Barriers to citing retracted literature exist in part due to the journals and databases that make this information hard to find. There are inconsistencies in the ways that retracted articles are labeled on journal websites and citation databases. While style guides provide guidance on how to cite retracted publications, authors may not be aware of the requirements from each writing style guide, and citation software does not automatically add this information to the citations. Librarians should raise concerns with database vendors on the poor performance of reporting retracted publications, and should educate users on proper citing behavior.

Monday, August 10, 11: 30 a.m.–3: 00 p.m.

# Information Services Session 1

## Seeing Our Open Access (OA) Options: A Comparison of Full-Text Finders

**Elizabeth Moreton,** Clinical Librarian, UNC Chapel Hill, Chapel Hill, North Carolina

**Jamie L. Conklin,** Health Sciences Librarian / Liaison to the School of Nursing, University of North Carolina at Chapel Hill, Chapel Hill, North Carolina

**Adam Dodd,** Data Analyst, Health Technology and Informatics, University of North Carolina, Chapel Hill, Durham, North Carolina

**Objectives**: OA full text finders offer the potential to save researchers time and money in article retrieval with just the click of a button by finding free copies of articles, but to date, studies comparing the user experience and retrieval capabilities of these tools are scarce. This study analyzes the features and effectiveness of OA full text finders in health disciplines.

**Methods**: The investigators tested several types of OA full text finders by attempting to retrieve the full text of a random set of articles in a variety of health disciplines. They performed a structured analysis of the software, looking at quantitative and qualitative dimensions such as data sources, number of steps to retrieve text, and overall ease of use. The investigators also tested the effectiveness of the tools by comparing the number of test articles retrieved by each tool.

**Results**: Overall, Google Scholar Button performed the best regardless of browser. Lazy Scholar and EndNote also performed well. A combination of Google Scholar Button in Chrome, which found the most articles, combined with EndNote, which missed the fewest articles, may be the best approach. It would also be easy to install several tools at once and check multiple sources almost instantly.

**Conclusions**: Though there are many types of OA full text finders, it may save potential users time and money to know which tool is easiest to use and provides access to the most free resources.

## BioData Club: A Partnership Model for Advancing Data Literacy

**Robin Champieux,** Direction of Education, Research and Clinical Outreach, Oregon Health & Science University, Portland, Oregon

**Ted Laderas,** Post-doctoral Research Fellow, OHSU/Department of Medical Informatics and Clinical Epidemiology, Portland, Oregon

**Eric Earl,** Research Associate, Oregon Health & Science University, Portland, Oregon

**Marijane White**

**Beth M. Duckles,** Research Consultant, Insightful

**Background**: BioData Club is an established data science educational initiative at our institution, formed in 2016. A collaboration of the OHSU Library, the Department of Medical Informatics and Clinical Epidemiology, research staff and graduate students, BioData Club hosts monthly workshops that advance knowledge and the use of data management, data science, and open science skills, tools, and best practices.

**Description**: With the support of an NNLM PNR Research and Data Engagement Award, the OHSU Library is working to develop and apply a methodology for associating existing BioData Club resources to relevant Data Information Literacy (DIL) competencies, enabling learners and educators to follow guided pathways for engaging with BioData Club events and materials. Additionally, we are partnering with OHSU students and postdoctoral fellows to develop workshops that leverage their emerging expertise and give them pedagogical training and mentored teaching experience. Our goal is to make BioData Club more educationally robust and accessible, and our evaluation plans include surveying educators, librarians, and learners about the value of our competency mapping methodology for facilitating student success, and their interest in engaging with BioData Club’s educational resources.

**Conclusion**: Our project has been developed to realize the following outcomes:

1). Learners will be able to discover and use educational resources that match their DIL learning goals, and gain knowledge that corresponds to specific scientific competencies and student learning outcomes.

2). Educators and librarians will be able to identify the relationship between BioData Club’s resources and specific DIL knowledge in order to strategically use our educational materials to impact student learning and demonstrate their educational contributions.

3). A larger and more diverse network of learners, educators, and information professionals will be able to discover, access, and reuse BioData Clubs resources.

## EndNote Comes to Campus: Lessons Learned from Supporting an EndNote Site License on an Academic Medical Campus

**Christi Piper, AHIP,** Reference Librarian, University of Colorado Anschutz Medical Campus, Aurora, Colorado

**Cecelia Vetter, AHIP,** NIH Fellow, Education Librarian, University of Colorado Anschutz Medical Campus, Aurora, Colorado

**Background**: An academic medical center acquired a site license for EndNote desktop in fall 2017 with support provided by the health sciences library. EndNote is the first license on campus that allows all faculty, staff, and students access to a paid citation management software. To support EndNote, the library developed a number of initiatives: training sessions and best practices, consultation procedures, a special interest group for library staff, and marketing practices. The support initiatives assist patrons use of EndNote from installation to publication. With two full years of data we have insight about potential requirements to support an EndNote site license.

**Description**: With the implementation of the EndNote site license, the library began tracking Endnote related library services and the amount of librarian time dedicated to supporting EndNote use. The library now provides a weekly one-shot class on using EndNote, EndNote consultations, assistance with troubleshooting software issues and with developing complex workflows for special groups. Additionally, the EndNote Special Interest Group maintains an EndNote LibGuide and discusses any EndNote problems, such as issues that result from updates to software or operating systems. Over the last two years the health science librarians have learned how to manage growing demands for EndNote training and dealt with challenges communicating with the Office of Information Technology, the official owner of the site license. This session will share information and report time data about maintaining a citation management software site license for libraries considering a similar program.

**Conclusion**: This session will present outcomes about the overall time spent by library staff supporting EndNote along with how that time was spent, such as teaching, consulting, troubleshooting, or involvement in the EndNote Special Interest Group. Additionally, information about what needs to be considered before ramping up support for a campus-wide citation management software, how EndNote can assist with project collaboration, best practices for establishing support initiatives, and best practices for communicating within the library and to campus will be presented.

## One Website to Rule Them All: Lessons Learned from a Series of Reorganizations, Integrations, and Creation of One University Libraries’ Website from Three

**Annie M. Thompson,** Director, Science & Engineering and Health Sciences Libraries; Interim Head, Norris Medical Library, University of Southern California, Los Angeles, California

**Megan Rosenbloom, AHIP,** Collection Strategies Librarian, UCLA Library, Valley Village, California

**Holly Thompson,** Information Services Librarian, USC, Los Angeles, California

**Background**: In 2017, the Health Sciences Libraries combined their ILS with the larger university library system’s. This integration led to several other transitions and reorganizations, including the 2018-2019 integration of the two standalone medical and dental library websites into a newly-designed university libraries’ site. The website integration challenged the Health Sciences Libraries to not only reorganize content and rethink services, but to conduct internal education about health sciences libraries’ roles and users to units that primarily serve undergraduate populations. How could one website speak to the needs of such a diverse range of users - from undergrads to clinical researchers?

**Description**: Initially, it was clear that there was little understanding of health sciences needs within the greater university libraries’ system. Health sciences librarians and staff established themselves as key stakeholders and partners with the website design project manager, allowing for them to have a big impact on the future direction of the entire project. This program focuses on external and internal communications strategies, change management during integration, and how to combine the best parts of very different systems into something new and better. Orienting around coherence for a wide range of users, the design process revealed further cultural and service differences among the libraries and areas for improvement in public services and collections management - not only their representation on the website, but the services themselves.

**Conclusion**: While a difficult transition, the overall project allowed for the libraries to work as a team in a time of change, creating a fresh approach for both internal and external users and an opportunity to rethink services. The Health Sciences Libraries staff learned to navigate the new organization and advocate for ourselves and our users. Ultimately, user comments and appreciation for the upgraded site were evidence that change can result in positive outcomes. Future projects include an ILL/Document Delivery service integration and creation of a libraries’ style guide for public facing communications (Libguides, video tutorials, and website content).

## Focusing on Improvement: Developing and Implementing Assessment for Reference Interactions, Research Consultations, Searching Services, and Library Classes

**Cecelia Vetter, AHIP,** NIH Fellow, Education Librarian, University of Colorado Anschutz Medical Campus, Aurora, Colorado

**Samantha Wilairat,** Graduate Assistant, University of Colorado Anschutz Medical Campus, Lakewood, Colorado

**Background**: The academic health sciences library strategic plan includes focusing on patron services assessment. The Education & Reference department provides three instructional services to end users– reference interactions, consultations, and library offered instruction sessions. Based upon a thorough literature review, librarians determined that including user feedback in assessment service analysis would allow the department to make data-driven improvements to the services. The department decided to develop and roll out assessment instruments/surveys for all three services with implementation beginning January 2020.

**Description**: A small Assessment Team was identified to draft the different service assessments. The librarians review current literature, other existing assessment tools, and best practice documents to identify and/or develop potential questions for the assessment instruments. The librarians will work with the different library faculty who oversee these services for feedback and best fit. The librarians will then bring in other faculty and staff for feedback and buy-in. With several different technology options, the department chose a recently purchased software for these initial efforts. The librarians will create the final versions of the instruments in the chosen technology for implementation.

**Conclusion**: The presentation will include information concerning barriers and issues faced by the librarians throughout the development process. It will include any technology glitches or considerations. The presentation will include samples of the instruments and report aggregate findings from the instruments’ use. It will also include best practices to consider for creating or rejuvenating assessment instruments and programs.

Monday, August 10, 11: 30 a.m.–3: 00 p.m.

# Lightning Talks 1

## Health and Wellness: Training for Community Health Workers

**Norice Lee,** Associate Library Director, Burrell College of Osteopathic Medicine, Las Cruces, New Mexico

**Erin W. Palazzolo,** Library Director and Professor of Biomedical Sciences, Burrell College of Osteopathic Medicine, Las Cruces, New Mexico

**Background**: This project has two major goals in training certified Community Health Workers (CHWs), community college students enrolled in a CHW program, and community members residing in the United States-Mexico border region: increasing the knowledge of suicide prevention and HIV/AIDs, and raising awareness and use of National Library of Medicine resources. Funding for the project supports the delivery of eight, six-hour sessions at four training sites in rural or underserved areas. Project partners include librarians, public health educators, and a Spanish language translator. Continuing education credits from the state’s Department of Public Health are available to certified CHWs completing these sessions.

**Description**: This one-year community outreach project targets current and future frontline Community Health Workers (CHWs) caring for those living in rural and underserved border areas. Understanding the significant role that CHWs play within their communities, the project partners came together to find ways to support them in their work, while also raising public awareness of two serious health concerns that exist in our region. With funding obtained for the project from the National Network of Libraries of Medicine South Central Region, the partners developed and are delivering bilingual training curricula on suicide prevention and HIV/AIDS, integrating National Library of Medicine and other online resources for session participants to access hands-on. Assessment of learning is underway measuring pre- and post-test results, in-session activity scores, and overall session evaluations. Achievement of quantitative grant goals and objectives by April 2020 will indicate project success.

**Conclusion**: Intended outcomes:

(1) At least forty Community Health Worker (CHW) and ten CHW student participants will attend a training on Suicide Prevention or HIV/AIDS with National Library of Medicine (NLM) online resources integrated into the training.

(2) Increase training participants' knowledge of suicide prevention.

(3) Increase training participants' knowledge of HIV/AIDS.

(4) Current and future CHWs will use NLM resources to help meet community members' health information needs.

(5) Increase use of high quality, evidence-based resources among CHWs and their community members.

(6) Support CHW education and state certification through the state's Department of Health, Office of Community Health Workers.

## Engaging for Health in Rural and Underserved Communities

**Nandita S. Mani,** Associate University Librarian & Director, Health Sciences Library, University of North Carolina, Chapel Hill, Chapel Hill, North Carolina

**Michelle Cawley,** Head of Clinical, Academic, and Research Engagement, UNC Chapel Hill, Chapel Hill, North Carolina

**Terri Ottosen, AHIP,** Community Engagement and Health Literacy Librarian, University of North Carolina at Chapel Hill, Chapel Hill, North Carolina

**Megan N. Fratta,** Community Outreach and Global Health Librarian, University of North Carolina at Chapel Hill, Chapel Hill, North Carolina

**Background**: A team of four librarians at the University of North Carolina at Chapel Hill's Health Sciences Library received funding from the National Network of Libraries of Medicine (NNLM) to conduct health literacy and consumer health training in five public libraries in rural and underserved areas of North Carolina. The goals of this project were to enable public library staff to provide consumer health information services through an introduction to health reference and health information resources, and to empower these libraries to lead a workshop for their community members.

**Description**: The team partnered with five public libraries in areas of the state with below average health literacy levels. We developed a two-part curriculum with assessments for the “train the trainer” sessions for public library staff. The first part was adapted from an NNLM curriculum and introduces library staff to consumer health reference and trustworthy sources of health information. The second part introduces the “Engage for Health” program curriculum developed by the Hospital & Healthsystem Association of Pennsylvania, Pennsylvania Library Association, and NNLM. This session encourages members of the community to actively engage in their health care and to ask questions of their doctors. We adapted NNLM evaluation tools for each group to assess participants’ learning.

**Conclusion**: In September and October 2019, we led sessions at five libraries reaching a total of 57 public library staff from 12 libraries. Two of the five libraries were able to lead at least one Engage for Health session in their communities prior to the COVID-19 pandemic. Lessons learned during this project will be applied to future outreach throughout the state.

## A Focus on Better Serving Veterinary Responders in the Future

**Laura Rey,** Library Associate II, Texas A&M University Medical Sciences Library/University of North Texas, Bryan, Texas

**Background**: Since Hurricane Katrina, the importance of veterinary disaster response has become recognized. The author’s institution founded its Veterinary Emergency Team (VET) in 2009. It is the largest veterinary disaster response team in the country; it is also a rotation for fourth-year veterinary students.

In October 2018, after receiving a call for new VET members, the library committed an interested staff member to explore information support opportunities for the teams.

**Description**: The library staff member went to trainings and conferences with the team and assisted with several deployments. The staff member identified several library and information opportunities and unasked reference questions.

Animal disaster information and response resources exist from a diverse group of organizations, including FEMA, Red Cross, National Library of Medicine, and ASPCA. Most of these resources focus on mitigation and preparation rather than response.

The pool of veterinary literature is smaller than with human medicine; specialties in veterinary medicine (ophthalmology, dentistry, etc.) use human literature to develop evidence-based practices. Because veterinary disaster response is interdisciplinary, the literature is spread, even hidden, in other areas of literature, like engineering.

**Conclusion**: Librarians have roles to play in support of veterinary disaster response:

Include acceptable levels of animal contamination for chemical and radiological responses, and zoonotic infectious diseases in tools like WISER, where responders already look for information.

Provide scholarly communication support to those writing about veterinary disaster response.

Collect literature relevant to veterinary disaster response needs in a central location and better index them.

Seek opportunities to become involved in information management during disasters in support of first responders, whether it’s helping manage patient records or incoming information about the disaster.

## Fun Labs: The Arts (and Crafts) of Relaxation

**Rebecca A. Morin,** Head of Research and Instruction, Tufts University, Boston, Massachusetts

**Katherine A. Morley Eramo,** Administrative Coordinator, Tufts University, Boston, Massachusetts

**Background**: How do librarians connect to students when most resources are online? How do educators foster community on a campus without a student center? How do students balance the demands of graduate study with the need for time outside of the classroom? One health sciences library has found an answer, building a robust program of “Fun Labs” that bring students together for non-academic activities in a re-envisioned 21st century library space. All it takes is glitter, glue guns, googly eyes, and a willingness to try something unexpected.

**Description**: On a dense urban campus with little space to spare, the library is the gathering place for 3000+ students looking to study and unwind. Fun Labs were born out of a need to better serve the non-academic needs of the community, while acknowledging the hectic pace of graduate student life in the Health Sciences. After one successful craft project involving pumpkins and quick-drying paint, library staff realized that simple, fast art projects in the unconventional setting of the library had potential as an exercise in relaxation and community-building. We soon started planning events monthly and experimented to uncover the best days of the week, time of day, and projects for maximum student participation. Projects were designed to be inexpensive, creative, self-directed, and portable, so students would not need to reserve a large block of time to participate.

**Conclusion**: Fun Labs continue to be popular events, and new projects are added based on student suggestion and continuous evaluation of which crafts draw the most participation. While the goal initially was to provide relaxing activities for stressed students, the program has evolved in unanticipated ways. Fun Labs bring together students from all academic programs on campus, provide a novel and unexpected way to use the library, and help to demystify the library and librarians by creating a relaxing “third space” outside of the rigors of the laboratory or classroom.

## Ask Me about ReDiReCT: Integrating National Library of Medicine (NLM) Resources into Disaster Preparedness and Response Cross-Disciplinary Training

**Sheila W. Green, AHIP,** Bryan Campus Librarian, Texas A&M University, Bryan, Texas

**Martin William Mufich,** Director of Disaster Preparedness, Response and Recovery, Clinical Assistant Professor, Texas A&M College of Nursing, college station, Texas

Background: The goal of this project, funded by the National Network of Libraries of Medicine / South Central Region, was to broadly disseminate information regarding availability and integration of NLM disaster-related resources and applications to Texas A&M University Health Sciences students, first-responders, and the community at large in the central Texas region. Using a train-the-trainer approach and student ambassador model, the project focused on enhancing student and responder knowledge and utility of NLM Disaster related resources.

Description: A Disaster Response Toolkit was developed in concert with Texas A&M University Medical Sciences Library linking to key resources by responder, planner or member of community from the NLM Disaster Information Management Research Center (DIMRC). The training was integrated into the Texas A&M University Health Disaster Week 2020. This inter-disciplinary concentration of more than 750 current and future disaster responders comes together with the community to explore disaster simulation and response. Disaster Week and the activities leading up to the event provided a unique opportunity to reach a broad group of future first responders. By developing a training focused on the availability, utility and application of the NLM resources and building a central resource or portal through University Libraries, the students were able to utilize and share the NLM disaster related resources in and across their respective professions.

Conclusions : Student ambassadors were debriefed regarding the experience, post-event surveys evaluated and pre-post site analytics collected to track activity and utilization of the Disaster Response Toolkit webpage. Lessons learned will be reviewed and applied to upcoming events that prepare our next group of current and future disaster planners and responders.

## Seeing the Whole Picture: Strategies for Expanding and Promoting a Circulating Board Game Collection for Maximum Visibility at Your Institution

**Andrew S. Hamilton,** Assistant Professor, Oregon Health & Science University, Portland, Oregon

**David Forero,** Technology Director, OHSU, Portland, Oregon

**Paul Gardner**

**Background**: After building a circulating collection of Medical/Scientific and Social/Cooperative board games in our Library, we initiated a program of advertising our new collection of games and sponsoring game nights to increase the visibility of the games to our users. This has allowed us to identify interested individuals and groups on campus to increase awareness and create opportunities to promote the further use of the collection at our institution.

**Description**: Our first effort that generated interest was prominently placing our game collection on shelves behind the Library Service Desk. We scheduled our first Library Game Night to coincide with the annual New Student BBQ. We promoted the event with flyers, e-mails, and social media posts. The event allowed us to connect with an attendee who then worked with us to host a 24-hour game marathon at the Library as part of a national charitable event entitled Extra Life Game Day. Plans are in the works to co-sponsor bi-monthly Game Nights in the Library with the Graduate Student Organization as well as conducting surveys of attendees about new games and preferences for

future events. All of our efforts have paid off with every game having circulated at least twice since the launch of the collection last year.

**Conclusion**: We will continue to examine circulation statistics for the games in our collection and gather information from events where our games are featured. We have been gathering feedback from attendees via surveys sent out at the conclusion of our events. Holding events and then establishing developing relationships with partners in the institution has proven to be a very effective way of promoting the use of the collection. We will continue to seek out new partners and locate funding sources on campus that will enable us to acquire new games and expand the scale and scope of future game events.

## Hiring Disabled Librarians: Methods Overview

**Gail Betz,** Research, Education and Outreach Librarian, University of Maryland, Baltimore, Baltimore, Maryland

**Objectives**: Research questions:

Main question: How do academic librarians with disabilities experience the hiring process?

Sub-question: What strategies have disabled academic librarians used in order to successfully navigate the hiring process?

**Methods**: This research project will be conducted using in depth interviews with disabled academic librarians. In-depth interviews allow for more organic and long-form sharing of experiences, which is ideal for discussing topics that involve a lot of potentially sensitive context and personal choice. Only employed librarians will be included for this study; the goal is to gather strategies and tips from librarians who have successfully navigated the academic hiring process with a disability. After interviewing 45 disabled librarians (any disability type), the author will code the qualitative data and pull out recurring themes.

This lightening talk will focus primarily on the literature review that informed the research questions and the chosen methodology. It is not anticipated that full coding of qualitative data will be complete by the date of this presentation, which is why the lightning talk format was chosen.

**Results**: By the time of this presentation, the author anticipates having completed all interviews but not having completed thematic coding. The results discussed will involve how successfully interviews can be conducted for a topic that is sensitive and personal, but necessary for the diversity and equity of our profession. How well did interviews work for this type of research, and how much was learned? Would a different method have been better or more helpful?

**Conclusions**: The conclusion will discuss the next steps of this research project- after collecting the qualitative data, what will be done with it? Was it of the quality and usefulness that I expected and will I be able to form a coherent set of best practices for other disabled academic librarians to use as they look for jobs? For the purposes of this lightning talk, I will be discussing whether the methodology I chose was the right fit.

## Domains of Professional Practice: Analysis of Publications in the *Journal of the Medical Library Association* from 2010 to 2019

**Katherine Goold Akers,** Biomedical Research & Data Specialist, Wayne State University, Detroit, Michigan

**Jill Boruff, AHIP,** Liaison Librarian, McGill University, Montreal, Quebec, Canada

**Roy Eugene Brown, AHIP,** Research and Education Librarian, Virginia Commonwealth University, North Chesterfield, Virginia

**Alexander J. Carroll, AHIP,** Librarian for STEM Research, Vanderbilt University, Nashville, Tennessee

**John W. Cyrus,** Research and Education Librarian, VCU Libraries, Richmond, Virginia

**Melanie J. Norton,** Head of Access and Delivery Services, Yale University, New Haven, Connecticut

**Holly Thompson,** Information Services Librarian, USC, Los Angeles, California

Background: The Medical Library Association (MLA) has defined seven domain hubs aligning to different areas of information professional practice. To assess the extent to which content in the Journal of the Medical Library Association (JMLA) is reflective of these domains, we will analyze the magnitude and temporal dynamics of JMLA articles aligning to each domain hub over the last 10 years.

Description: Bibliographic records for 453 articles published in JMLA from 2010 to 2019 were downloaded from Web of Science and screened using Covidence software. The title and abstract of each article was screened by two reviewers, each of whom assigned the article with up to two tags corresponding to an MLA domain hub (i.e., information services, information management, education, professionalism and leadership, innovation and research practice, clinical support, and global health and health equity). Articles not related to any domain hub were classified as “other”.

Results: We found that JMLA articles aligned well with six of the MLA domain hubs: information services (n= 136), information management (n = 123), education (n = 109), innovation and research practice (n = 109), clinical support (n = 97), and professionalism and leadership (n = 85). Few JMLA articles aligned with the global health and health equity domain hub (n = 18). Twenty articles did not relate to any domain hub.

Conclusions: These results inform the MLA community about our strengths in health informational professional practice as reflected by articles published in JMLA. We plan to fill gaps in JMLA content related to global health and health equity by issuing a call for submissions and parterning with related MLA Caucuses.

Monday, August 10, 12: 00 p.m.–1: 00 p.m.

---

## PubMed Update - Monday's Video Chat

**Marie Collins**

**Rebecca Brown, AHIP,** Training Development Specialist, Spencer S. Eccles Health Sciences Library, Salt Lake City, Utah

**Kathi Canese,** PubMed, Program Manager, NCBI NLM NIH, Bethesda, Maryland

Marie Collins from the National Library of Medicine provides the latest news on PubMed for the Medical Library Association vConference 2020. Marie highlights the improved mobile experience, the “Best Match” sort, interface improvements, and where to go for support and training.

Monday, August 10, 3: 15 p.m.–4: 30 p.m.

---

# Clinical Support Immersion Session 2

## Envision Yourself in the Electronic Health Record: What You Need to Know to Embed Library Services

**Nicole Capdarest-Arest, AHIP,** Head, Blaisdell Medical Library, University of California, Davis, Sacramento, California

**Frances Drone-Silvers,** Biomedical Information Specialist, Carle, Urbana, Illinois

**Alison P. Gehred,** Reference Librarian, Nationwide Children's Hospital, Columbus, Ohio

**Judy Hansen,** Medical Librarian, Washington University, Saint Louis, Missouri

**Erica Lake, AHIP,** Research & Evaluation Specialist, Essentia Health, Duluth, Minnesota

**Shawn Steidinger, AHIP,** Assistant Librarian for Clinical Services, Eccles Health Sciences Library / University of Utah, Salt Lake City, Utah

**Session Format:** Mostly interactive, with synthesis portion at end

**Objective:** For many years, librarians at MLA have discussed their role with clinical workflows and the electronic health record (EHR). However, many still do not have access to the EHR and many hospitals and medical centers still do not offer library services from the EHR or in clinical services. This interactive session will offer participants the opportunity to learn about barriers to library integration into clinical workflows, specifically the EHR, and gain tips for achieving stakeholder buy-in and building clinical informatics integrations through advocacy and learning from others’ achievements in this arena.

**Instructional Methods:** Participants will be guided by the presenters, who each have experience in EHR and clinical integrations. Through interactive small-group discussions using "Liberating Structures"-style themes and processes, groups will explore topics, learning from each other and the presenters who will guide the discussions. Themed topics will relate to the session's learning outcomes and will be highlighted for the entire group throughout the session. In this way, participants will have the opportunity to broaden their individual knowledge and skills around the learning outcomes in a cooperative learning-focused environment.

**Participant Engagement:** Participants will actively participate in guided small group discussions throughout the immersion session; these discussions will vary in process using "Liberating Structures"-style models such as “Impromptu Networking,” “Wicked Questions,” etc. Breakout rooms will create opportunities for novices to learn from those with more experience. Using these structured discussions, participants will work to create an action plan to integrate or grow library knowledge in the EHR. There will also be opportunities to share across groups so that knowledge generated can be assimilated by everyone.

**Sponsors:** Medical Informatics Caucus; Consumer & Patient Health Information Caucus; Clinical Librarians and Evidence-based Healthcare Caucus; Hospital Libraries Caucus

Monday, August 10, 3: 15 p.m.–4: 30 p.m.

---

# Professionalism & Leadership Immersion Session 1

## Envisioning Diverse and Inclusive Library Programming and Outreach

**Ryan Harris, AHIP,** Head of Research and Instructional Services, University of North Carolina, Charlotte, Charlotte, North Carolina

**Shannon D. Jones, AHIP,** Director of Libraries, Medical Univ. of South Carolina, Charleston, South Carolina

**Kelsa Bartley,** Education & Outreach Librarian, University of Miami Miller School of Medicine, Miami, Florida

**JJ Pionke,** Applied Health Sciences Librarian, University of Illinois at Urbana-Champaign, Champaign, Illinois

**Hector R. Perez-Gilbe, AHIP,** Research Librarian for the Health Sciences, Collection Development, Tustin, California

**Session Format:** Interactive panel discussion with Q and A., followed by small group discussions/activities and group share.

**Objective:** The objective of this immersion session will be to provide practical examples of programming, outreach, and instruction/education that librarians are actively doing to educate both librarians and the larger community on issues related to diversity, equity, inclusion, and accessibility. Attendees will also gain a better understanding of these concepts as well.

**Instructional Methods:** Several panelists will present on different programming/outreach they have done. Areas of focus will be book clubs focusing on implicit bias, library accessibility walkthrough, and outreach to the trans community on issues related to health and medication. The audience will be able to ask questions after each speaker. Small table discussions: questions for the group to consider will be, what activities have you done already to facilitate these topics. What are the barriers you see in doing these programs? What are some partnerships you could do to help achieve these?

Group share of discussions from each small group.

**Participant Engagement:** Participants will be able to ask panelists questions

Participants will work on small group activities and share with the rest of the group attending

**Sponsors:** African American Medical Library Alliance Caucus

Tuesday, August 11, 10: 15 a.m.–11: 30 a.m.

# Innovation & Research Practice Immersion Session 2

## Roles for Librarians in Research Impact Services

**Christopher William Belter,** Informationist, National Institutes of Health, Bethesda, Maryland

**Karen Gutzman,** Head, Research Assessment and Communications Department, Northwestern University, Chicago, Illinois

**Tyler Nix,** Informationist, University of Michigan, Ann Arbor, Michigan

**Amy Suiter,** Librarian, Washington University School of Medicine in St. Louis, Saint Louis, Missouri

**Session Format:** The session will be a hybrid between a panel and a fishbowl in which the panelists introduce the roles and guide a series of discussions with the audience about the activities performed in each role and the knowledge and skills necessary to perform them.

**Objective:** Demand for research impact services at medical libraries is growing, but guidelines and best practices on providing these services are scarce. In this session, we reflect on the ways that research impact services have been implemented at our and other organizations and, based on those experiences, offer a preliminary set of roles that medical librarians can fill in providing these services. Through a series of guided discussions with the audience, we will introduce and refine both the roles themselves and the knowledge and skills necessary to perform each role. By the end of the session, participants will have a better understanding of the different forms that research impact services can take in medical libraries and how those services can be implemented at their own institutions.

**Instructional Methods:** The session will begin with a brief presentation introducing what the four roles are and how they relate to each other. There will then be a series of guided discussions on each of the roles facilitated by librarians actively engaged in each role. The discussions will describe the activities performed in each role, highlight the knowledge and skills needed to perform these activities, and provide ample time for audience participation in defining these roles. The session will conclude with a brief recap of the discussions and a series of suggestions on how librarians can implement one or more of these roles in their own libraries.

**Participant Engagement:** During the guided discussions, participants will be encouraged to talk about how they are or are not implementing these roles in their own libraries. Participants will also be encouraged to ask questions of both the panelists and their fellow participants.

**Sponsors:** This session is co-sponsored by the MLA Data Caucus and the MLA Translational Sciences Collaboration Caucus.

Tuesday, August 11, 10: 15 a.m.–11: 30 a.m.

# Innovation & Research Practice Immersion Session 4

## Mobilizing Librarian Engagement with Computable Biomedical Knowledge: Our Roles in Curation, Stewardship, Dissemination, and Advocacy for Equity

**Marisa L. Conte, AHIP,** Associate Director, Research and Informatics, University of Michigan, Ann Arbor, Michigan

**Terrie R. Wheeler,** Director, Weill Cornell Medicine, New York, New York

**Philip D. Walker,** Director, Eskind Biomedical Library / Vanderbilt University, Nashville, Tennessee

**Gerald (Jerry) Perry, AHIP, FMLA,** Associate Dean, and Director, University of Arizona Health Sciences Library, University of Arizona Libraries, Tucson, Arizona

**Chris Shaffer, AHIP,** University Librarian, University of California, San Francisco

**Gabriel Rios,** Library Director, Ruth Lilly Medical Library, Indianapolis, Indiana

**Peter Robert Oxley,** Associate Director of Research Services, Weill Cornell Medicine, New York, New York

**Session Format:** We offer an immersion into the emerging field of computable biomedical knowledge (CBK), and identify opportunities for librarian engagement.

**Objective:** Recognizing the expertise of librarians in information organization, description and dissemination, we introduce a new community: Mobilizing Computable Biomedical Knowledge (MCBK). We present an overview of the community and current projects, and introduce community workgroups.

Digital technology enables knowledge to be represented in computable forms expressed in machine-readable code. Computable biomedical knowledge is decision specific advice to improve human health leveraged at speed and scale by providers and patients through electronic health information systems. Recent CBK research has demonstrated its applicability in the clinical, research, public health, and educational realms. The librarians’ role is essential to curating, storing, and disseminating these computable formats of biomedical knowledge, often called knowledge objects, which are a new form of scholarly publication. Further, librarians advocate for equitably serving the health learning and knowledge needs of all humans, to ensure that each knowledge object reflects the best and most current medical evidence, that the computable formats are interoperable using open standards, and that they are trusted by for use by health systems, societies and individuals to improve human and population health.

**Instructional Methods:**

Part 1 [25 mins]: Session introduction, introductory video on MCBK, video presentations by 3 invited speakers, providing an overview of the MCBK community, commercial partners, CBK as scholarly communication

Part 2: [25 minutes] Participants join breakout rooms aligned with MCBK workgroups [Standards; Technical Infrastructure; Policy & Coordination to Ensure Quality and Trust; Sustainability for Mobilization and Inclusion]. Facilitators rotate between rooms so that all participants have an opportunity to engage with representatives of each working group.

Part 3 [15 minutes] Breakout groups will reconvene for the wrapup - an open discussion guided by focused questions.

**Sponsors:**

Marisa Conte: Taubman Health Sciences Library, University of Michigan; Standards for MCBK Workgroup

Peter Oxley, Samuel J. Wood Library, Weill Cornell Medicine

Jerry Perry: University of Arizona Libraries, University of Arizona; MCBK Steering Committee, Co-chair, Sustainability for Mobilization and Inclusion Workgroup

Gabriel Rios, Ruth Lilly Medical Library, Indiana University School of Medicine; Sustainability for Mobilization and Inclusion Workgroup

Chris Shaffer, University of California, San Francisco; MCBK Steering Committee, Co-chair Technical Infrastructure for MCBK Workgroup

Philip Walker, Annette and Irwin Eskind Biomedical Library, Vanderbilt University; Policy and Coordination to Ensure Quality and Trust Workgroup

Terrie Wheeler, Director, Samuel J. Wood Library, Weill Cornell Medicine; Sustainability for Mobilization and Inclusion Workgroup

Tuesday, August 11, 10: 15 a.m.–11: 30 a.m.

## Professionalism & Leadership Immersion Session 3

### Inclusive Imagining: Encouraging and Capturing Diverse Perspectives to Create an Inspiring, Actionable Strategic Plan

**Catherine B. Soehner,** Associate Dean for Research & Director, Spencer S. Eccles Health Sciences Library, Salt Lake City, Utah

**Elizabeth Frakes, AHIP,** Assistant Librarian for Clinical Services, University of Utah, Salt Lake City, Utah

**Heidi Greenberg,** Associate Director, Administration, University of Utah, SALT LAKE CITY

**Melanie Hawks,** HR Associate Director, University of Utah, Salt Lake City

**Nancy Lombardo,** Librarian, Head of Digital Publishing, University of Utah, Salt Lake City, Utah

**Brandon Patterson,** Technology Engagement Librarian, University of Utah, Salt Lake City, Utah

**Nena Schvaneveldt, AHIP,** Education Librarian, University of Utah, Salt Lake City, Utah

**Shawn Steidinger, AHIP,** Assistant Librarian for Clinical Services, Eccles Health Sciences Library / University of Utah, Salt Lake City, Utah

**Christy Jarvis, AHIP,** Associate Director, University of Utah, Salt Lake City, Utah

**Session Format:** This interactive panel discussion will feature health sciences librarians & professional staff sharing their diverse perspectives and leading attendees through a series of reflective activities.

**Objective:** Join in an interactive discussion where our panel will share how we wrestled with and ultimately answered important questions about strategic planning that you may also face.

• What makes a strategic plan useful, rather than a forgotten artifact of a mind-numbing wordsmithing exercise?

• Do we really need to include everyone in the library in creating the strategic plan?

• What if we can't include all of the ideas of the participants?

• Could we actually enjoy ourselves in the process of creating a strategic plan?

**Instructional Methods:** Each presenter will review their role in the strategic planning process and explain their perspective on the questions listed in the “Objectives” section, using specific examples of choices, conversations, and tools that shaped the experience for the better. Presenters will encourage attendees to identify opportunities for applying concepts learned in small- or large-scale planning processes. A resource list of change management, leadership, and strategic planning readings will be distributed to participants for further context.

**Participant Engagement:** This Immersion Program will begin with attendees participating in a “think, pair, share” exercise to draw on their previous experience with strategic planning. Those ideas will be shared out to the larger group and incorporated into the presentations, during which participants will be encouraged to reflect and engage in open Q&A to the presenters.

**Sponsors:** None

Tuesday, August 11, 11: 30 a.m.–3: 00 p.m.

# Clinical Support Session 2

Moderator: Elizabeth Frakes, AHIP

## Visible, Valuable, and Validated: Reviving a Safety Net Hospital Library

**Timothy Kenny,** Clinical Library Manager / Senior Librarian, JPS Health Network & UNT Health Science Center, Fort Worth, Texas

**Kellie Boyd, AHIP,** Clinical Librarian, University of North Texas Health Science Center/JPS Health Network, Fort Worth, Texas

**Background**: Presents an overview of an innovative relaunch of library services in a county safety net hospital through increased visibility of librarians, retooled value focused library services, and leveraging interprofessional partnerships across hospital departments. It will include a review of qualitative and quantitative metrics as well as a discussion of innovative approaches and barriers encountered during the library relaunch.

**Description**: The library relaunch involved preemptive discussion with hospital stakeholders, particularly the Quality department, in the needs and scope of renewed library services. Everything related to the library was redesigned and rebooted including: the website; the collection; the physical space, & scope of services. This was accomplished through unique partnerships with a health science university, diligent tracking of metrics, and gathering feedback from our key stakeholders (both formal and informal). Grants and awards were procured to launch innovative new programs and expand breadth of services.

**Conclusion**: In the next year, we plan to conduct a thorough retrospective analysis via a mixed methods study to examine the impact of relaunching the library. In addition, we hope to leverage the same scope of retrospective data to draw connections between the new library and library services and impacts on patient care, clinical outcomes, and other quality measures central to the goals of the hospital.

## Reducing Patient Care Delays through a Multimodal Patient Literacy Program

**Sarah L. Carnes, AHIP,** Clinical Librarian, Veterans Health Administration, Bedford, Massachusetts

**Background**: Dental patients at a Veterans Affairs Medical Center experience delays in patient care as a result of health literacy challenges. Due to misinformation, complicated conditions and interventions, anxiety, and a lack of access to helpful information, many patients are unclear and even unprepared for appointments, treatments, and surgeries. This results in delays, cancellations, and rescheduling of care at an estimated rate of 3-15% per month. The Clinical Librarian conducted an extensive process improvement project in collaboration with an interdisciplinary dental clinic staff to mitigate these challenges and reduce delays.

**Description**: The Clinical Librarian used a Lean Yellow Belt methodology to comprehensively define the problem, including using an Input-Process-Output tool. Through meetings and observation, she created a process map and collected data which helped identify specific opportunities for improvement. Analysis of the current state process by staff of multiple professions and facilitated by the Clinical Librarian revealed operational barriers and led to more effective root cause analysis. Courses of action were devised in a collaborative manner, leading to buy-in and relevance. By

defining countermeasures and conducting PDSA (Plan, Do, Study, and Act), the librarian and dental staff refined their solutions and enhanced them with patient-friendly tools and content. The ultimate solutions relied on information in a variety of formats, including analog and digital, delivered by multiple means, including hard copy, email, Patient iPads, and patient care room monitors.

**Conclusion**: Staff have now incorporated the new processes into their patient encounters prior to, during, and after appointments. As the solutions were a departure from known processes and methods, roll out was very gradual. This has allowed for further refining of improvements. The evaluation of the project outcomes demonstrated high satisfaction from both Veterans and staff. In addition, there was a 10-20% reduction in care delays and cancellations. As we add topics related to COVID-19, we anticipate further improvement in patients' preparation for and understanding of their patient care--with the added benefit of enhancing the experiences of patients and staff.

## Empowering Culturally Competent Care: How Hospital Librarians Support Cultural and Linguistic Competency at a Major Health Care Institution

**Eve Melton, AHIP,** Regional Director Library Services NCAL, Kaiser Permanente, Stockton, California

**Scott Boothe,** Lead Librarian, Kaiser Permanente, Berkeley, California

**Quincy D. McCrary,** Health Sciences Librarian, Kaiser Permanente, Richmond, California

Objectives: California has long used legislation to drive social justice in medicine. Laws passed to further continuing medical education (CME) have served to strengthen CME programs in the medical community. CME is also employed to teach professionals how to understand and communicate with patients from diverse backgrounds. Hospital librarians are valuable assets to CME programs. Often tasked with providing resources to augment CME presentations, librarians are empowering physicians to help diverse and socially disadvantaged patients achieve optimal health. This research will explore hospital librarian's support of culturally competent care and California Assembly Bill 1195 at a large healthcare organization.

Methods: This study gathered examples and data of culturally competent education assistance provided by librarians from 2012 to 2019. The authors focused on assistance provided to CME committee members, CME presenters, and CME coordinators. Surveys were administered to both librarians who provide literature searches for CME activities and for those who coordinate CME activities at their affiliated hospitals. Each survey was created to gain insight on the involvement between the librarians at the various KP hospitals and their associated CME departments.

Results: With the implementation of a shared reference platform in 2012, California regions began collecting data on cultural and linguistic competency (CLC) reference activities. As of 2019, 1496 CLC activities were supported. The assistance provided was largely made up of literature searches. A total of 31 surveys were sent to librarians in both hospital regions in Northern and Southern California. Questions focused on how librarians received requests from their respective CME departments, what types of information were presented for CME activities, and how searches were conducted. Survey data showed all respondents reported receiving assistance from the librarians in their medical centers. CME planners were also asked if the help received was beneficial to meeting the intent of California Assembly Bill 1195. Most respondents reported the assistance as extremely beneficial to patient centered care.

Conclusion: Hospitals continue to care for increasingly diverse populations shaped by patients with varied social and cultural backgrounds. Providing health professionals with culturally competent information resources serves to improve the quality of care provided to our patients. This research

unveiled how librarians’ support of culturally competent care, inclusivity, and diversity is beneficial to health care professionals. Such support has the potential to save CME presenters’ research time while satisfying the mandate of AB1195.

## Addressing Disparities in Physician Access to Information in Support of Evidence-Based Practice

**Erinn E. Aspinall, AHIP,** Associate Director, Program Development and Strategy, University of Minnesota Health Sciences Libraries, Saint Paul, Minnesota

**Shanda Hunt,** Public Health Library Liaison & Data Curation Specialist, University of Minnesota, Minneapolis, Minnesota

**Nicole Theis-Mahon, AHIP,** Liaison to the School of Dentistry & Collections Coordinator, University of Minnesota, Minneapolis, Minnesota

**Katherine V. Chew,** Research/Outreach Services Librarian, University of Minnesota Health Sciences Libraries, Minneapolis, Minnesota

**Evan Olawsky,** PhD Candidate, University of Minnesota Department of Biostatistics

**Objectives**: The purpose of this study is to determine if STATE physicians have access to information resources needed to support evidence-based practice (EBP), which supports a culture of safety and patient-centered care.

**Methods**: A survey was distributed to 13,726 physicians licensed to practice in STATE who provided their email address to the STATE Board of Medical Practice. Short-answer responses were independently sorted into categories by two authors, and disagreements were settled by a third. Data analyses were conducted using R. ANOVA models were used to assess differences in continuous variables and across groups in categorical variables. Two-sided t-tests were conducted to compare continuous responses between groups and to compare categorical access to information resources between affiliated and unaffiliated physicians. P-values were adjusted for multiple comparisons by performing a Bonferroni correction. Logistic regression was used to assess the significance of affiliation on categorical responses after controlling for several other variables. Additionally, time spent answering questions was calculated from the number of questions asked and the time spent seeking answers per day.

**Results**: Sample size was 877. Participants spent 24 minutes seeking answers and had an average of 4.41 clinical questions that could not be immediately answered. Thirty-one percent of questions were never answered, and 17% of questions were left unanswered even when physicians sought information. Physicians reported high levels of information needs met (85.8%), though reported limited access to drug resources, citation databases, systematic reviews, and full-text books and articles. Results showed use of flawed search strategies and unreliable sources. A key finding was the extent to which workplace affiliation broadens disparities in information access.

**Conclusions**: National and regional approaches can work to support EBP by reducing the information gap caused by workplace affiliation and other barriers. Further research should be done to identify partnerships, funding, infrastructure, and support that can address information gaps caused by workplace affiliation, and meet physician information-seeking behavior and needs.

Tuesday, August 11, 11: 30 a.m.–3: 00 p.m.

## Education Session 3

Moderator: Chelsea Misquith

### Reimagining Long-Standing Physician Associate Research Curriculum Support

**Caitlin Meyer,** Research & Education Librarian, Yale University, New Haven

**Background**: The library has been involved in the Physician Associate Program research curriculum for more than a decade. Historically, the library taught three 90-minute lectures that started with introductory search skills and scaled to systematic searching. Librarians then held individual meetings with 40+ students about their thesis topics. After a wave of retirements and organizational restructuring, this model was no longer sustainable and overdue for an overhaul.

**Description**: Working closely with faculty, the librarian transitioned the in-class time from lecture to team-based learning. The activities introduced were modeled directly after skills the students needed to complete a successful thesis project: defining research questions, executing scoping and systematic searches across various databases, critical appraisal, and recordkeeping for reproducibility. Instead of individual meetings, the librarian now holds two mandatory, small-group workshops: one for scoping searches and topic refinement for the thesis proposal, and one for creating systematic search strategies for the final project. The new educational initiative is being evaluated immediately through post-class and post-workshop surveys.

**Conclusion**: The new model allows students to take advantage of their peers' subject expertise, learn highly relevant skills in an interactive way, and uses librarian time/resources more efficiently than the previous model. After the first year, the faculty partner anecdotally reported higher citation counts, and more theses were awarded the honors designation than the years before.

### Helping Our Newest Users Focus: Orientation Videos and Materials for Incoming Students

**John D. Jones,** Instruction & Curriculum Librarian, Strauss Health Sciences Library CUAnschutz Medical Campus, Denver, Colorado

**Background**: Libraries are slowly being squeezed out of orientation and introductory time with new incoming students. Many teaching faculty believe and assume that students know what the library has to offer and that the students will seek us out when they have a need. Students believe that anything can be learned from a video – and it better be short and focused. So, considering all of this, the Teaching Team embarked on a mission to identify important areas/services for incoming students to know about and to create appropriate online asynchronous training/education material to convey this information in lieu of face-to-face time.

**Description**: The Teaching Team met to discuss what was needed and strategies for identifying and filling this gap. The team invited campus software experts to present on the available technologies which could be pursued to meet the need. Brainstorming sessions were held to identify specific topics to cover and these were then ranked to identify the most critical to produce first. Team members volunteered for specific topics and began the process of choosing the appropriate medium to convey the required information and to create a product to use with the incoming students. Scripts were written. Handouts were created. Links were identified. Web-based applications were

explored. All of the parts were loaded into a Library 101 guide. Social media and traditional flyers were used to promote the information. Much of the guide has been incorporated into the FAQ database.

**Conclusion**: The presentation will outline the process, as well as, talk about lessons learned, best practices, unifying strategies, and branding. It will discuss how these products have been used to date and what the next steps might be.

## Developing In-Person Teaching Excellence Workshops through Four Pedagogical Lenses: Cultural Humility, Active Learning, Dealing with Challenges, and Technology

**Remzi Kizilboga,** Instructional Designer, NNLM Training Office, Bloomington, Indiana

**Molly Knapp, AHIP,** Training Development Specialist, NNLM Training Office, Houston, Texas

**Rachel Gatewood,** Instructional Designer, University of Iowa, Iowa City

**Jessi Van Der Volgen, AHIP,** Assistant Director NNLM Training Office, University of Utah, Salt Lake City, Utah

**Background**: As a part of a large national organization, our training office aims to support and create high-quality teaching and learning opportunities to serve the diverse needs of audiences nationwide. To achieve this goal, we developed and delivered in-person workshops to our organization’s library staff at eight regional medical libraries. Our in-person training was developed through four pedagogical lenses: 1) Cultural Humility, 2) Active Learning, 3) Dealing with Challenges, and 4) Technology. This paper describes the design, delivery, and outcomes of our in-person training program through these lenses.

**Description**: Each in-person training program is a day and a half. To help our audience’s preparation for the workshops, we delivered supportive materials and small activities through our learning management system, enabling us to provide blended-learning opportunities for our audience. For each of the aforementioned pedagogical lenses, our training team created meta-teaching activities to promote learning, give examples and spark ideas for teaching in-person, with an emphasis on active learning interventions. Between each workshop, we used evaluation data and personal reflections from our team to improve training. At the end of the workshop, learners deliver a presentation as an application and indicator of their learning. Presentations are reviewed under the four pedagogical lenses by other learners.

**Conclusion**: At submission, three of eight workshops are completed, with our last workshop is scheduled to March 2020. By our final submission date, we will have more data to discuss. Preliminary pre and post-survey results of the in-person training, and reflections from our training team indicate positive learning outcomes. For example, learners reported an increase in applying active learning strategies, applying culturally humble strategies and content, presentation skills, using technology in-person teaching and training, and giving and receiving feedback. As a result of our workshops, we created a community of practice where the participants can contribute to the resources continuously.

## Integrated Library Instruction for a Doctoral (PhD) Program in Health-Related Sciences

**Talicia A. Tarver, AHIP,** Research and Education Librarian, Virginia Commonwealth University, Richmond, Virginia

**Nina Exner,** Research Data Librarian, Virginia Commonwealth University, Richmond, Virginia

**Lauretta Cathers,** Program Director, VCU, Richmond

**Background**: This paper summarizes and evaluates a partnership between subject and functional liaison librarians and a health science Ph.D. program to deliver hybrid-online doctoral learning. A health sciences librarian and a data librarian partnered with the director of an interdisciplinary health professions Ph.D. program that includes students from various health professions in a yearly doctoral cohort for a hybrid-online Dissertation Seminar course. Past doctoral students had demonstrated several challenges in information use throughout their prospectus assignment. To improve their use of the literature in all chapters of the dissertation, the librarians were asked to integrate library instruction throughout the dissertation seminar.

**Description**: After discussing course objectives with the program director, the librarians created learning objects aligned with these objectives. The content was posted in an online course guide, linked in the class’ Blackboard page to appear as the class progressed. The class met biweekly and online for assignments in modules based around the chapters of a dissertation proposal. Library materials developed for each module included resources on APA citation style, videos outlining the steps for conducting a literature review, videos on researching theory, and a video on study design literature. The Blackboard discussion board was used to hold “library office hours” in addition to the lead instructor’s office hours. Formative, low-stakes self-assessments were included for library content in each module. A summative assessment was collected at the end. The next version of the course (in progress) has incorporated assessment findings.

**Conclusion**: The students reported a favorable response to having the library materials available in a timely manner, and prospectus assignments showed a more effective incorporation of the literature throughout student research planning. The librarians and program director presented this project to other faculty within the health professions college. This presentation led to connections with other departments and discussion of broadening the library’s role in health professions graduate education. Learning objects from the course are also forming the seeds of a content hub for resources that can be used for all curriculums in the graduate health professions college.

Tuesday, August 11, 11: 30 a.m.–3: 00 p.m.

# Education Session 4

Moderator: Roxanne Bogucka

## Collaborating with a Health Promotion Class to Assess Library Employee Wellness Needs

**Terry Kit Selfe, AHIP,** Translational Research and Impact Librarian, University of Florida, Gainesville, Florida

**Melissa L. Rethlefsen, AHIP,** Associate Dean and Fackler Director, University of Florida, Gainesville, Florida

**Background**: The future of employee wellness initiatives is bright as more corporate and academic employers recognize that investing in a healthy workforce is money well spent. The University of Florida Libraries recently adopted a new strategic direction aimed at improving the wellness of its employees, users, and communities. To support this direction, a Library Wellness Committee (LWC) was created. While the LWC was in its formative stages, a College of Health and Human Performance (CHHP) faculty member approached a library administrator asking if the library would be willing to serve as a real-world example for her Worksite Health Promotion (WHP) course.

**Description**: An Associate Dean of the Libraries and a Co-chair of the LWC met with the WHP course instructor to discuss the possibilities and agreed to the following project. According to the plan, the WHP students would assess the wellness needs of our employees by two different methods, environmental scan and electronic survey. For the former, ten student teams were assigned a different library site to visit and assess the availability of healthy food options, space for offering wellness programs, etc. For the latter, students are in the process of creating an anonymous survey to be distributed to library employees electronically after approval from the human resources department of the Libraries and the LWC. All of the resulting data will be shared with the LWC to serve as a baseline assessment and inform their future program planning.

**Conclusion**: The course collaboration has been a productive, mutually beneficial way to engage with students and faculty. Beyond the obvious advantage to the students of the opportunity to learn through practical application of the subject matter, the libraries also benefited. It enabled us to draw on: the instructor’s depth of knowledge regarding worksite health promotion, the students’ time and effort in performing the assessments, and an unbiased outsiders’ perspective of our employees’ wellness needs, We plan to extend the partnership into next semester, allowing the incoming class to analyze the data, develop wellness programming recommendations and present them to the LWC.

## What Makes a Monster? Innovative Teaching and Outreach to Start a Campus Conversation

**Hannah Friggle Norton, AHIP,** Chair, Health Science Center Library - Gainesville, University of Florida, Gainesville, Florida

**Michele R. Tennant, AHIP, FMLA,** University Librarian, Health Science Center Library, University of Florida, Gainesville, Florida

**Jane Morgan-Daniel, AHIP,** Community Engagement and Health Literacy Liaison Librarian, University of Florida, Gainesville, Florida

**Ariel FitzGerald Pomputius,** Health Science Liaison Librarian, University of Florida, Gainesville, Florida

**Nina Stoyan-Rosenzweig,** Senior Associate in Libraries, University of Florida, Gainesville, Florida

**Background**: Over the course of a year, this library-based project team asked members of our university community, What Makes a Monster? Through connections with organizations across campus and classroom engagement with students, the team created a campus-wide conversation about monster theory and how American society creates monsters- the other- during times of stress.

**Description**: Led by the health science archivist, with four other librarians, the project incorporated curriculum innovation, a speaker series, film screening, and two monster-themed art contests. Undergraduates were deeply engaged in a monster-focused course, using a novel model at our institution of 4 honors students teaching fellow undergraduates (under the guidance of the archivist) and all students curating an exhibit for the local art museum.

Falling during the 200th anniversary of Mary Shelley’s Frankenstein, the project tapped into conversations regarding medical ethics and monstrosity, with one speaker focusing on the Frankenstein movies and screening The Bride of Frankenstein. Another speaker discussed how we make sense of the sometimes beautiful art of “monstrous men.” To conclude the project, the library hosted two art contests, with participants considering the perspectives on monsters discussed throughout the year and responding through drawings, paintings, or comics.

**Conclusion**: The project was highly successful in furthering the conversation on monsters, and received significant media interest as well as engagement in the various activities, as measured by participation and attendance in events, as well as its impact on larger university programs. It also provided a model- that continues to be promoted by the Museum of Art and the Honors Program- and thus has generated institutional change.

## Streamlining Library Classes: Scheduling, Marketing, and Data Gathering in Order to Increase the Value of a Library Service

**Hannah J. Craven,** Research & Scholarly Communications Librarian, Indiana University School of Medicine, Indianapolis, Indiana

**Julia C. Stumpff,** Instructional Design Librarian, Indiana University School of Medicine, Indianapolis, Indiana

**Background**: Are regularly-scheduled library classes a valuable service offered by libraries? Librarians were hesitant to offer recurring library classes because attendance was historically low. The scheduling system was cumbersome, and there was no consistent assessment of attendance, class content, or teaching quality. To improve our recurring library classes, the library increased marketing efforts, standardized evaluation, and adopted a scheduling program that tracked registration and attendance. The library now has baseline data to make future evidence-based decisions about scheduled classes and course content. Further, our library administration can use collected data to demonstrate value of this library service to the medical school.

**Description**: After surveying current practices and discovering the campus had access to a scheduling program, two librarians and a staff member trained themselves on and then employed the program. The program features adopted were: course templates, registration, email reminders, post-class

emails, and attendance tracking. Post-class emails were populated with links to class evaluations. The librarians and staff joined the marketing team to promote the classes across campuses. Marketing prominently noted classes were simultaneously offered in the library and live-streamed. At the end of each semester, class and survey data were exported and cleaned for analysis. With the new system in place, the number of classes offered doubled. This led to three times the number of attendees in Spring 2019 compared to Fall 2018. The average class size increased by one. Survey response rate was 84%. Class attrition rate was 36%.

**Conclusion**: Registration and attendance data is now easily separated by month, day, and hour and will inform future classes scheduling. Further, the qualitative analysis of the evaluation data will be analyzed to examine comments about class content and teaching. This analysis will then be shared with class instructors to improve course delivery. In the future, the registration form will ask about participants’ campus locations to determine if distance learners are reached at other campuses across the state. Lastly, we hope to improve class evaluation questions in order to influence future class content and improve teaching delivery.

## Bad Reputation: Using Three-Dimensional Printed Heart Models to Supplement Cardiac Ultrasound Training for Undergraduate Medical Students

**Kate M. Serralde,** Unit Manager, The Methdology Lab/Preston Smith Library/ TTUHSC, Lubbock

**Objectives**: Undergraduate medical students struggle with cardiac anatomy and physiology when learning ultrasound. 3D printing is an emerging technology yet to be evaluated in teaching first-year undergraduate medical students integrated Point of Care Ultrasound. The study goal was to determine if 3D heart models are effective to aid students in acquiring and interpreting normal cardiac images obtained using B-mode ultrasound.

**Methods**: Methods: 3D cross-sectioned models depicting both halves of a human heart cut along the parasternal long axis (PLAX) plane were created using computed tomography (CT) DICOM data from a normal adult male patient. This data set was processed to develop a solid model, and converted to a stereolithographic file format which was used to generate the 3D print. The resulting cross-sectioned heart models were made available to each group of first-year medical students (total n=196) completing a cardiac POCUS activity in the Structure and Function of Major Organ Systems block at Texas Tech University Health Sciences Center School of Medicine. As part of this cardiac ultrasound imaging activity using standardized patients, each student had to acquire and submit a PLAX image obtained during the session for a grade.

**Results**: Results: The in-house printing for both halves of our 3D cardiac models was only $64. This is considerably less expensive than commercially available models currently on the market. The post-evaluations indicated that the medical student found the 3D sectioned PLAX heart models to be extremely useful for orienting themselves during cardiac ultrasound training.

**Conclusions**: Experiential learning has been linked to improved skill development, with one benefit being better retention of clinical skills education. Incorporating 3D printed heart models in medical anatomy and physiology education, provides evidence supporting 3D printed cardiac models help novice sonographers better understand surface and cardiac anatomy. This translates into helping them achieve proper probe placement and appreciate the orientation of the heart in the body, and thus aids them in rapidly becoming more proficient in obtaining suitable ultrasound images. Students recommended that similar models depicting various regions obtained during short axis imaging be developed and incorporated into the POCUS sessions.

## Promoting Health Literacy and Improved Self-Care Management of Incarcerated Populations Using Secure Tablet Technology

**Gail Kouame,** Assistant Director for Research & Education Services, Augusta University, Augusta, Georgia

**James A. Johnson,** Director, Institute of Public & Preventive Health, Augusta University, Augusta, Georgia

**Objectives**: 1) Deliver health education modules to justice-involved individuals currently incarcerated at project partner sites

2) Measure the impact that health information training has on the level of health literacy, the confidence to improve self-care management skills, and interactions with health care providers

3) Follow a sample of justice-involved individuals for 6 months after they are released to collect post-release outcomes

**Methods**: This presentation describes findings from an Information Resource Grant to Reduce Health Disparities project, funded by the National Library of Medicine. The project aims to engage justice-involved individuals with health education to enhance their knowledge and use of health services and resources.

Project investigators developed a needs assessment tool, educational content, and pre- and post-intervention questionnaires based on those created for an earlier pilot project, and additionally informed by project investigators with input from a nationally recognized health literacy expert. The consent materials, needs assessment, health-related educational modules, as well as pre-and post-intervention questionnaires were made available to justice-involved individuals in ten participating correctional facilities using an existing, secure tablet-based system developed by Edovo. Reminder messages were also sent via the tablets to participating individuals who had accessed the educational content but had not yet completed the questionnaires.

**Results**: Over 3000 individuals accessed the educational content via the tablets and over 100 have completed the pre and post-intervention questionnaires. Significant findings include improvements in: searching for health information; understanding health status; engaging in preventive care; knowing what questions to ask health care providers about diagnoses, treatment, medications, and self-care; and knowing when to seek medical care.

**Conclusions**: Providing health education modules paired with self-directed learning via secure tablets computers is an effective method for improving health literacy and self-care management for incarcerated persons.

Tuesday, August 11, 11: 30 a.m.–3: 00 p.m.

# Innovation & Research Practice Session 1

Moderator: Erin Keenan

## Assessing the Impact of Consultations with Librarians on Faculty Research

**John W. Cyrus,** Research and Education Librarian, VCU Libraries, Richmond, Virginia

**Rachel Amelia Santose Koenig,** Research and Education Librarian, Tompkins-McCaw Library for the Health Sciences, VCU Libraries, Richmond, Virginia

**Objectives**: Research consultations are a standard service in academic libraries consuming significant library resources. However, there is a lack of scholarship evaluating this essential service. This project will increase understanding of the impact of research consultations on faculty productivity and will describe the role of consultations in the research process. This project will also document scholarly products resulting from these consultations.

**Methods**: To better understand consultation impact, the researchers surveyed faculty from five health sciences schools at a large urban, public university who are known users of the consultation service within the past nine months and complete follow-up focus groups with a subset of participants. Survey questions were piloted by other librarians in the same department as well as among users within the university’s health sciences schools. Input from the pilot was incorporated into the survey. The final survey consists of six items, including level of satisfaction with the service, whether the consultation achieved its desired purpose, and any subsequent scholarly products. Finally, the survey asks faculty to comment on the likelihood of their return for future projects and on their referral of others to library consultation services.

**Results**: The survey was distributed to 129 faculty members, of which 67 (52%) completed. Each health sciences school was represented in the survey with the majority of responses from Medicine and Nursing. Approximately half of respondents had been at the institution for 0-5 years and reported 10% or less of their time dedicated to research. Satisfaction with research consultations was high with 100% reporting that the consultation achieved its desired purpose and 75% reporting high satisfaction with their last librarian consultation. Consultations contributed to the following products: journal articles, scholarly presentations, posters, book chapters, a grant proposal background, and various student research projects. Sixty-three percent of participants planned to either acknowledge the librarian’s assistance or include the librarian as co-author on the product.

**Conclusions**: These findings enable us to document the ways in which librarians participate in complex research interactions with faculty. Librarians contribute throughout the faculty research process but especially during question generation, background research, the selection of appropriate methods, and manuscript preparation and dissemination. The results of this survey will be combined with a larger program evaluation effort. The combined results will be used to inform program planning and administrative decision-making within the library.

## Flawed Research in Focus: Retracted Publications in Pharmacy Systematic Reviews

**Caitlin Bakker, AHIP,** Research Services Librarian and Medical School Liaison, University of Minnesota, Minneapolis, Minnesota

**Sarah Jane Brown,** Liaison Librarian to the College of Pharmacy and Medical School, Duluth Campus, University of Minnesota, Minneapolis, Minnesota

**Nicole Theis-Mahon, AHIP,** Liaison to the School of Dentistry & Collections Coordinator, University of Minnesota, Minneapolis, Minnesota

**Objectives**: Publications are retracted for a multitude of reasons; however, identification of retraction notices remains inconsistent and post-retraction use of materials continues. We investigated how retracted publications influence systematic reviews and other evidence-based literature in Pharmacy. We analyzed retracted publications cited in systematic and other comprehensive reviews and examined the application of quality assessment or risk of bias tools.

**Methods**: Retracted research articles and clinical studies in the fields of Pharmacology, Drug Design, and Toxicology were identified through the RetractionWatch Database. Searches were performed in Scopus and Web of Science to identify all articles and reviews citing each retracted item. These results were collected and deduplicated in EndNote, and uploaded to Rayyan for screening. The included systematic and other comprehensive reviews were then assessed to determine whether the retracted publication was cited positively, negatively, or neutrally in support of the findings. We also examined which, if any, quality assessment or risk of bias tool was used in the systematic review, and what the results of that evaluation were. We conducted an analysis of citation trends to show the impact of retracted publications in systematic reviews and the methodological quality of those reviews.

## Grey Literature Inclusion in Nursing Systematic Reviews: A Bibliometric Analysis

**Kerry Dhakal,** Assistant Professor/Research and Education Librarian, The Ohio State University, Columbus, Ohio

**Objectives**: The Cochrane Collaboration recommends the inclusion of grey literature sources when conducting comprehensive systematic reviews. The objective of this bibliometric study is to learn if authors publishing systematic reviews in core nursing journals are including grey literature in their reviews.

**Methods**: We are conducting a search for systematic reviews published between 2017-2019 in journals indexed in PubMed's core nursing journal subset that state they included grey literature sources in their literature review. Articles found in this search that are indexed as systematic reviews or include the phrase systematic review in its title will be included. These articles will then be screened and reviewed using Covidence software. Both authors of this research study will screen and review the articles of this study to ensure inter-rater reliability. We will have an independent reviewer to monitor disagreements in study selection for the data analysis phase of the study. We will conduct a subset study to determine if there are any patterns between those studies that do report including grey literature and those that do not.

**Results**: We are still in the data collection phase of this study. Results will provide insight into the number and type of systematic review study that include grey literature sources published in core nursing journals indexed in PubMed. Variables that will be considered in the data analysis phase include: number of studies that include grey literature and those that do not. Other potential patterns related to journal publication selection, author instructions, number of authors, author affiliations, or author specialties will also be reported.

**Conclusions**: Conclusion are not available at this time. We will report conclusions at the conference, if selected to present.

## Testing a Random Item Screening Approach to Constructing Search Validation Sets

**Edwin Vincent Sperr Jr., AHIP,** Clincial Information Librarian, AU/UGA Medical Partnership, Athens, Georgia

**Objectives**: Complex searches are sometimes derived from tests against a predefined “validation set” of items, a robust but resource-intensive approach. One approach for simplifying the construction of such a validation set relies on selecting from randomly derived results from an initial “seed” search. Larger validation sets are likely better to reflect results from the database as a whole, but searcher effort increases with the number of results screened. A balance between search fidelity and usability is required.

**Methods**: To determine the optimal balance between usability and search fidelity, two approaches were used. In the first, several simple searches were programmatically performed in PubMed using the NCBI’s eutils API, and randomly selected results were extracted from each. These sets of random results were then subsampled into sets of different sizes, and each set was searched against a variety of test conditions. The proportion of each set selected by those conditions were then compared between differently sized sets.

In the second approach, human participants were asked to visit a web application designed to show them randomly selected items from a PubMed search (matched with a selection rubric) and allow them to manually sort those results into “good” and “bad” sets. The resulting sets of items for each rubric were compared between participants.

**Results**: In the first arm of the study, the proportions of items found for each sample search/test condition did vary between randomly selected sets of results. However, it was observed that the amount of this variance decreased markedly as the size of the random set increased to 50.

In the second arm, participants completed a total of 23 set-construction tasks. On average, participants took 34 minutes to complete each task (min: 13 minutes, max: 119 minutes) and screened 2.56 items for each “good” one (min: 1.15, max: 3.85). There was substantial variance in the number of “bad” items for a given question between respondents, but testing showed statistical similarities between their results in at least one case.

**Conclusions**: These results do not yet yield a single answer to the required size for a validation set, but they do suggest that this approach has promise. More research is needed.

Tuesday, August 11, 11: 30 a.m.–3: 00 p.m.

---

# Lightning Talks 2

Moderator: Bridget Jivanelli

## Nursing Clinical Ladder Program: The Embedded Librarian

**Danielle N. Linden, AHIP,** Medical Library Manager, St. Joseph Hospital / Providence St. Joseph Health, Orange, California

**Background**: Changes to our hospital’s performance review process required Clinical Nurse IIIs and IVs to complete an annual Evidence Based Practice (EBP) or Quality Improvement (QI) project in order to maintain or advance their status on the Clinical Ladder. Nurse Researcher met with Clinical Nurse IIs, IIIs and IVs to review the requirements and held Proposal Writing sessions to prepare nurses. All attendees were referred to Librarian-led literature search classes. Researcher met with nurses individually to discuss projects and sent nurses to consult with Librarian. As a result, Librarian conducted 50 literature searches in support of the EBP and QI projects.

**Description**: All projects were to be presented at the hospital’s annual EBP Conference as poster presentations. Librarian sits on Planning Committee and reviewed posters for consistency and clarity based on guidelines provided by the Health System’s Nursing Research Council. Posters were sent to the hospital’s print vendor and returned to the library for storage until the day of conference. Librarian worked with the Health System’s Digital Assets Librarian to have conference website created within the organization’s Digital Commons. Conference site was loaded with the agenda, speakers’ presentations and a pdf copy of each poster. Attendees were encouraged to print materials ahead of time and review their colleagues’ work. Conference site on Digital Commons will remain in perpetuity and serves as internal and external dissemination of new nursing knowledge as a required component of the American Nurses Credentialing Center’s Magnet Recognition Program.

**Conclusion**: Building relationships with Nursing Leadership, Nurse Researcher and staff nurses allows for greater collaboration between clinical and library staff. Offering services that may have been traditionally out of the Librarian’s scope can serve to position oneself as an integral part of the team. Networking across the organization and highlighting the varied skill set a Librarian can provide proves to be a value added service.

## Your Valuable Contribution to Getting and Keeping Magnet Status for Your Hospital

**Helen-Ann Brown Epstein, AHIP, FMLA,** Informationist, Virtua, Mt Laurel, New Jersey

**Janina Kaldan, AHIP,** Library Manager, Morristown Medical Center Health Science Library, Morristown, New Jersey

**Background**: Magnet hospitals are an elite group. Currently there are around 509 hospitals worldwide with Magnet status. Obtaining and keeping Magnet Status is a prestigious accomplishment that recognizes a hospital's superior nursing process and quality patient care leading to the highest levels of safety, quality and patient satisfaction. The hospital librarian, being a member of the Magnet Team can play a major supporting role in obtaining and keeping Magnet status to keep their hospital's competitive advantage.

**Description**: Focusing on the 5 Model Components and the 14 Magnet forces, this abstract suggests tasks and relationships for the librarian with nursing leadership and nursing staff. The librarian must build a strong collection of information resources and offer a variety of access points and learning opportunities to form best practices and practice evidence based care. Also, the librarian should support and participate in initiatives, like a residency or scholarly publishing. The librarian should create alerts of relevant topics, follow them closely and push literature to significant people. As for a journal club, the librarian may facilitate or certainly supply quality papers for discussion. The librarian should maintain a repository of the nurse's publications, posters and podium presentations. The librarian may help to gather, index and edit the required Magnet documents. The librarian is eager to speak to the Magnet appraiser.

**Conclusion**: For participating in the tasks around the Core components and Magnet Forces and serving on the Magnet Team as a Magnet Champion, a hospital librarian may be recognized by the ANCC as an exemplar for supplying quality information resources and service to educate nurses so they may be smarter and better care providers.

## Educating Future Health Sciences Librarians: A Training Program for Graduate Students

**Lauren Elizabeth Robinson,** Medicine Liaison Librarian, University of Kentucky, Lexington, Kentucky

**Stephanie M. Henderson,** Nursing Liaison Librarian, University of Kentucky, Lexington, Kentucky

**Background**: This presentation will discuss a novel training program for our graduate students that builds their awareness of health sciences librarianship. The program will teach them skills that will increase their marketability after graduation. In order to train the next generation of health sciences librarians, it’s imperative that we bridge the gap between what students learn in library school and skills needed to excel on the job. The goal of our six-month training program was to help fuel interest in health sciences librarianship and teach entry level skills to future health sciences librarians.

**Description**: Our medical library was awarded a graduate assistant (GA) for the 2019-2020 academic year. After a GA was hired, we sought to develop a series of trainings that would both enrich the GA’s academic studies in librarianship and enhance their interest in becoming a medical librarian. The courses that were offered ranged from 1.5 to 2 hours in length, and covered topics such as basic and advanced searching, evidence-based practice, scholarly communications, research data management, and systematic reviews.

**Conclusion**: Feedback will be requested that will gauge whether or not the courses offered were beneficial, what we could improve, if the courses supplemented their academic studies, and whether or not the trainings increased their interest in becoming a health sciences librarian.

## New Liaison Librarians: Envisioning and Establishing Meaningful Faculty Partnerships

**Amanda Nevius,** Research & Instruction Librarian - Dental Liaison, Tufts University, Boston, Massachusetts

**Nena Schvaneveldt, AHIP,** Education Librarian, University of Utah, Salt Lake City, Utah

**Background**: Share strategies that worked at two separate institutions to establish relationships with faculty as librarians new to the respective institutions that can lead to integration into student curriculum of evidence-based practice and research, as well as developing the relationship into a true partnership.

**Description**: As mid-career librarians starting at new institutions, we were faced with the common challenge of establishing ourselves as liaisons while advocating for new integration of evidence-based practice and research into the school's curriculum. We desired to establish these interprofessional relationships from the beginning as a partnerships, leading to true course integration rather than less meaningful one-shots.

We vocalized at committee meetings and social events regarding the needs we perceived and ways we could help fill these, rather than targeting specific courses or faculty. Faculty who self-identified as sharing our needs and values then reached out to us, including one program's faculty and administration requesting in-person office hours within the school itself. This laid the groundwork to build collaborative partnerships leading to new yet meaningful and flexible long-term integration. We gathered feedback from our faculty partners to determine their perspectives on what led to these meaningful partnerships.

**Conclusion**: We evaluate faculty-librarian relationships as partnerships if faculty contacted and invited the librarian to integrate into a course, as opposed to the librarian requesting course time or faculty requesting a one-shot. We achieved a total of six such partnerships. We gathered feedback from some of these faculty about our collaboration and will present from both the librarian and faculty perspectives what made these collaborative partnerships work.

## Missing Fields=Misinformed: Why EndNote Customization Is Crucial

**Melissa Ratajeski, AHIP,** Coordinator of Data Management Services, University of Pittsburgh, Health Sciences Library System, Pittsburgh, Pennsylvania

**Rebekah Miller,** Research & Instruction Librarian, University of Pittsburgh, Pittsburgh, Pennsylvania

**Background**: After completing a PubMed search, a librarian will often import the retrieved citations into bibliographic management software such as EndNote for delivery to a patron. However, based on the citation information provided, can the patron easily tell whether the article was retracted, whether there are any comments on the article, or if the authors have any potential conflicts of interest? If the answer is no, librarians need to consider the potential risk this introduces, especially if the article is being considered for inclusion into a systematic review, driving a clinical decision, or as a reference in a grant application.

**Description**: The standard PubMed import filter in EndNote does not automatically include many important fields, including retraction, comment notices, or expressions of concern. Also of note, conflict of interest statements are placed into illogical fields within EndNote. Recognizing these problems, we created a customized EndNote import filter to ensure that all crucial MEDLINE fields would be brought into the software and available for review. We are now talking with librarians within our department to establish best practices for delivering citations depending on project workflow. For example, screening citations within the DistillerSR software requires different considerations compared to an outputted bibliography. .

**Conclusion**: Our goal is to prevent patrons from making misinformed decisions due to incomplete citation records. This lightning talk will demonstrate the steps needed to customize the EndNote PubMed import filter and how to edit preference settings and output styles to display the new fields.

## Library to Library: Knowledge Exchange between Two Health Sciences Library Neighbors

**Megan De Armond,** Research and Instruction Librarian, Touro University Nevada, Henderson

**Anna Ferri,** Research and Learning Librarian, Roseman University of Health Sciences, Henderson, Nevada

**Background**: Two geographically close Academic Health Science Libraries that serve similar constituents, offer similar services and face related challenges and needs, recognized that effective collaboration would be beneficial to our respective institutions and colleagues. The goals of our collaborative project are to 1) improve communication and information sharing 2) identify and build opportunities for our staff members to collectively build new skills and knowledge 3) identify and increase our involvement in existing partnerships at our respective institutions.

**Description**: The development of an active knowledge exchange program came as the result of both libraries hiring librarians new to medical and health sciences librarianship. Building off their own need and desire to develop a local network, they initiated a series of in-person meetings with defined agenda topics to address common challenges and discover new opportunities. Meetings were held at both locations enabling attendees to experience spaces and see workflows in action. The current and ongoing benefits of our knowledge exchange have been: observing how our respective institutions function including ways that procedures and roles align or differ; finding innovative ways to solve common challenges, and sharing knowledge of overlapping tools as well as benefits of differing resources. Immediate benefits include improved workflows around e-resource management and the development of collaborative approaches to research support and training.

**Conclusion**: Our knowledge exchange program is in progress.

The outcomes we expect to measure are:

- Improved organization workflows across library departments/areas

- Improved search strategy and research skills including management of systematic reviews with students and faculty

- Preventing duplication of efforts

- Increased visibility and credibility of respective libraries

- Cost savings with improved skillsets

## Is the Way Clear? Assessing the Usefulness of Resource Guide Sharing in Academic-Public Library Collaboration

**Christina Heinrich,** Research and Instruction Librarian, Tufts University Hirsh Health Sciences Library, Boston, Massachusetts

**Amanda Nevius,** Research & Instruction Librarian - Dental Liaison, Tufts University, Boston, Massachusetts

**Catherine Tess Grynoch,** Research Data & Scholarly Communications Librarian, University of Massachusetts Medical School, Worcester, Massachusetts

**A'Llyn Ettien, AHIP,** Collections Management Librarian, Boston University Medical Library, Boston, Massachusetts

**Katherine A. Morley Eramo,** Administrative Coordinator, Tufts University, Boston, Massachusetts

**Objectives**: This study assesses resource guides as a means of low-barrier, geography-agnostic interprofessional outreach. Resource guides are a mainstay of library services, but how effective is

the format as a method of sharing expertise? This study aims to assess the impact of distributing a resource guide on public library workers’ knowledge of and use of consumer health resources.

**Methods**: Using a two-group pretest/posttest design, consenting public library workers within a single metro area were given a pretest where they were surveyed on their current perceived and actual knowledge of consumer health resources. Subjects were then randomly allocated into control and intervention groups. The intervention group received a digital copy of a consumer health information resource guide designed by health sciences librarians. After a period of three months, both groups completed a posttest identical to the initial pretest survey. At the end of the study, the control group also received a copy of the guide.

**Results**: The intervention group that received the resource packet showed improvement in neither perceived nor actual knowledge of consumer health resources when compared to the control group that did not receive the resource packet. However, the sample size was very small: this is not a statistically significant result.

**Conclusions**: The primary utility of this pilot study is to inform future research on resource guides as a form of interprofessional outreach. This study alone does not prove the [in]efficacy of the resource guide used or guides in general. Distributing resource guides is a very common method of outreach, and further assessment is needed to determine if they are effective.

Tuesday, August 11, 11: 30 a.m.–3: 00 p.m.

# Professionalism & Leadership Session 1

Moderator: Brenda M. Linares, AHIP

## Breaking the Silence: Hosting Awareness Events on Campus during Crisis

**Shawn Steidinger, AHIP,** Assistant Librarian for Clinical Services, Eccles Health Sciences Library / University of Utah, Salt Lake City, Utah

**Brandon Patterson,** Technology Engagement Librarian, University of Utah, Salt Lake City, Utah

**Joan Marcotte Gregory, AHIP,** Associate Director for Access and Inclusion, University of Utah, Salt Lake City, Utah

**Heidi Greenberg,** Associate Director, Administration, University of Utah, SALT LAKE CITY

**Donna Baluchi,** Library Supervisor, University of Utah, Salt Lake City, Utah

**Background**: This academic health sciences library hosted a series of events in 2019 focusing on intimate partner violence (IPV). The collective goal was to bring awareness to the prevalence of incidents involving sexual assault and domestic violence in our community, some resulting in death. We sought to change perspective by focusing on the community around the victim, rehabilitation of the perpetrator and showcasing new technologies and research. Programming required sensitive but impactful publicity and interactions.

**Description**: The series included a lecture, panel discussion, banner display, interactive exhibit, and an event featuring virtual reality. We collaborated with the nursing college to develop events that would be relevant to a campus-wide audience. A campus wellness center compiled local and national IPV resources. Funding for the events came from multiple campus departments. The planning committee was composed of five from the library and two from nursing.

The events were well attended and highlighted in articles in local and university news outlets. While all events were important, the interactive exhibit featuring victims’ stories was the most halting experience. The effect was evidenced by comments like, “Of everything we have seen the library do, this was the most impactful”. The presence of the exhibit was inescapable, drawing people in, creating a visceral understanding of the toll of IPV.

**Conclusion**: We achieved bringing awareness of IPV to an affected campus in a sensitive manner. We were commended for our decision to not ignore issues facing our community and for contributing to a dialogue around healing.

## Perfecting Best Practices to Address Future Challenges in a Geographically Dispersed Hospital Library System

**Joy Rodriguez,** Medical Librarian, Kaiser Permanente Fresno Medical Center, Fresno, California

**Marina Aiello,** Lead Librarian, Instructional Design and Technology, Kaiser Permanente, Stockton, California

**Ana M. Macias, AHIP,** Manager Library Services Sacramento, Kaiser Permanente NCAL, Sacramento, California

**Tenisha Jones,** Medical Librarian, Kaiser Permanente, South San Francisco

**Chandrika Kanungo,** Manager, HSL, Kaiser Permanente, Santa Clara, California

**David G. Keddle,** Director, Medical Library Services, Kaiser Permanente Medical Center, Woodland Hills, California

**Background**: An inter-regional library committee was formed to review and compare varying administrative processes and best practices across geographically dispersed hospital libraries. Strategic decisions were made as to what would comprise a core set of standardized policies that govern all libraries, and a set of procedures or best practices addressing the steps involved to carry out the tasks. Best practices will be used to streamline our services, consistently align with our policies and support the mission of the organization. Standardizing procedures will eliminate redundancy and outdated services as well as improve customer service.

**Description**: The Standards and Policies committee identified the need for procedures that aligned with the approved library policies - namely, Education and Information Services, Library Space Management, Library Technology, Resource Management, Scope of Services and Unauthorized Users. The project involved gathering team-based feedback about identified best practice areas, creating templates and examples of procedures, and drafting the procedures. In addition, the project included using cloud-based collaboration software to review and reconcile the team’s comments before aligning them to their respective policy and submitting the final procedures to the National Library Committee for approval. We called upon the collective experience and expertise of the regional librarians to establish best practices that will streamline our work and reduce administrative complications. The procedures developed include an on-boarding checklist for new employees, inventory and weeding, and streamlining work in our integrated library system.

**Conclusion**: A triennial review of the existing library policies by all librarians coincided with the procedures project. After receiving extensive comments and feedback, the committee revised and updated the policies which facilitated the creation of the procedures. The validated and approved policies and procedures provide a consistent and positive impact on the day-to-day operations of the libraries, regardless of their geographic location. The success of our efforts continues to strengthen the unity of our inter-regional department by the harmonization of various tasks, which ensures greater productivity and consistency in the services offered to our patrons.

## Increasing Library and Librarian Visibility through a Semi-Custom E-Newsletter

**Erinn E. Aspinall, AHIP,** Associate Director, Program Development and Strategy, University of Minnesota Health Sciences Libraries, Saint Paul, Minnesota

**Background**: To develop a communications mechanism that would 1) provide cohesive and consistent communication, 2) offer high-touch content that increases librarian face and name recognition, and 3) provide custom content to meet librarian needs and reflect specific user groups.

**Description**: A semi-custom e-newsletter was developed to provide cohesive and consistent messaging from liaison librarians to their department while offering high-touch custom content. The body is identical for each of the 10 e-newsletters distributed each quarter, though librarians can request customization to reflect their audience. Custom elements include subject lines and reply to fields, welcome messages, headshots, and signatures. Librarians participate in content creation through collaborative content selection, where ranking determines content inclusion and placement within the e-newsletter. Open rates average 70% across 9 distributions (10 e-newsletters/distribution). This is 50 percentage points higher than industry standards, and 14

percentage points higher than our non-custom e-newsletters. Anecdotally, the semi-custom e-newsletters strengthen relationships with user groups as illustrated by librarian feedback. The success of the semi-custom format has led to its implementation across the university library system with similar results.

**Conclusion**: Librarian-led communication can build important relationships with user communities. However, taking an individual approach to communication can lead to inconsistencies with how information is communicated and what information is ultimately shared. Using a strategic marketing approach through the use of semi-custom e-newsletters can result in a high return on investment as it allows for the creation of consistent and cohesive messaging over time while promoting individual librarians to their community.

## A Vision toward Healing: One Library’s Response to a Mass Shooting

**Ryan Harris, AHIP,** Head of Research and Instructional Services, University of North Carolina, Charlotte, Charlotte, North Carolina

**Natalie Ornat,** Humanities Librarian, University of North Carolina at Charlotte, Charlotte, North Carolina

**Background**: During the spring 2019 semester, an active shooting took place in a building located next to an academic library on campus. Five students were injured and two were killed. Many library staff and faculty were on campus at the time and sheltered in place; including several that had students with them during the incident. This paper will discuss the library’s response after the shooting and activities and programming to support library staff and students as the new school year began. The paper will also give perspective and advice on how to potentially respond to an event like this

**Description**: The library was open the day after the shooting to allow patrons to pick up items they abandoned during their evacuation and to allow students to study. Broken doors, abandoned electronics, and half-eaten snacks created an unsettling tableau of the past day’s panic and uncertainty, which made it difficult for library employees to work that day. Library leadership identified the need for employees to undergo optional active shooter training and receive more targeted support from campus counseling. Campus counseling services focused on helping employees identify students in crisis and how to guide them to proper resources for assistance, along with supporting co-workers showing signs of post-traumatic stress and trauma. Several librarians identified a need to have organized activities to welcome students back on campus and reintroduce the library as a safe space at the start of the new academic year.

**Conclusion**: To help reestablish the library as a safe space for learning, community, and growth, a student engagement committee was formed of library faculty and staff. This committee takes a holistic approach to support student wellness and will work alongside campus partners to plan events that encourage self-care, community, and fun.

Staff and faculty have continued to be encouraged to participate in on-campus counseling that has been provided by the university. One library faculty member is serving on the campus Remembrance Committee, which is planning activities and making decisions about the campus’ response to the incident.

Tuesday, August 11, 1: 00 p.m.–2: 00 p.m.

---

# PubMed Update - Tuesday's Video Chat

Marie Collins

Mike Davidson, Librarian, Office of Engagement and Training (OET), National Library of Medicine (NLM)

Molly Knapp, AHIP, Training Development Specialist, NNLM Training Office, Houston, Texas

Marie Collins from the National Library of Medicine provides the latest news on PubMed for the Medical Library Association vConference 2020. Marie highlights the improved mobile experience, the “Best Match” sort, interface improvements, and where to go for support and training.

Tuesday, August 11, 3: 15 p.m.–4: 30 p.m.

# Education Immersion Session 1

## The Future Is Now: Physician Assistant Programs, Practice, and the Library

**Brandi Tuttle, AHIP,** Research & Education Librarian, Duke University Medical Center Library & Archives, Durham, North Carolina

**Caitlin Meyer,** Research & Education Librarian, Yale University, New Haven

**Jolene M. Miller, AHIP,** Director, Mulford Health Science Library, University of Toledo, Toledo, Ohio

**Justine Strand de Oliveira,** Professor Emeritus, Duke University Department of Family Medicine and Community Health, Sao Bras de Alportel, Portugal

**Session Format:** This session will feature a moderated panel discussion and facilitated round tables for small group discussion.

**Objective:** There are now 238 physician assistant programs in the United States and over 130,000 practicing Physician Assistants (PAs). How are medical libraries supporting this rapidly expanding segment of the healthcare workforce? This immersion session will bring together PAs and librarians to evaluate the current landscape of PA education and clinical support, share best practices, and network. The panelists will cover different aspects of supporting PA Programs and practicing physician assistants. Some of the topics will include: an overview of the birth of the Physician Assistant profession and where the profession is headed; supporting the students and faculty of both in-person and online PA programs; curricular and accreditation considerations; an partnering with practicing PAs.

**Instructional Methods:** This session will feature a moderated panel discussion with three Physician Assistant (PA) Library Liaisons who serve an in-person PA educational program, a distance education PA program, and/or PAs in the clinic. Those panelists will overview their services and explore similarities, differences and challenges faced across institutions. We also will have a video presentation from a well-known Physician Assistant clinician/educator on the history and future of the PA profession to further acquaint attendees with the field. After the panel presentations, attendees will discuss topics proposed by the panelists in smaller, facilitated groups. Round table discussion topics may include: mapping library services to curricular and accreditation requirements, strategies for reaching distance students, supporting practicing PAs, building support for new programs, and more.

**Participant Engagement:** Participants will be able to participate in PollEverywhere questions at the beginning of the session to get a sense of who is in the room and how they are currently serving PAs. They will be able to submit questions during the panel component of the session through the conference app and during the Q & A panel portion. During the round table component, participants will share their thoughts and experiences on a pre-defined topic with their smaller group. As a wrap up, each table will be given the opportunity to share a major take away with the larger group.

**Sponsors:** Technology in Education Caucus; Libraries in Health Science Curriculum Caucus; and Clinical Librarians and Evidence-Based Healthcare Caucus

Tuesday, August 11, 3: 15 p.m.–4: 30 p.m.

# Information Services Immersion Session 4

## Collaborating with Nonlibrarians: Enriching Your Teaching, Research, and Engagement

**Jane Kinkus Yatcilla,** Associate Professor of Information Studies, Purdue University Libraries & School of Information Studies, West Lafayette, Indiana

**Katherine Goold Akers,** Biomedical Research & Data Specialist, Wayne State University, Detroit, Michigan

**Lindsay E. Blake, AHIP,** Clinical Services Librarian, University of Arkansas for Medical Sciences, Little Rock, Arkansas

**Jonathan Eldredge, AHIP,** Associate Professor, HSLIC, University of New Mexico, Albuquerque, New Mexico

**Hannah Schilperoort,** Information Services Librarian, University of Southern California, Los Angeles, California

**Natalie Tagge,** Head, Podiatry Library, Temple University Health Sciences Libraries, Philadelphia, Pennsylvania

**Dede Rios, AHIP,** Director of Optometric & Clinical Library Services, Rosenberg School of Optometry, San Antonio, Texas

**Ayaba Logan,** Research and Education Informationist, Medical University of South Carolina, Charleston, South Carolina

**Session Format:** This session will begin with a panel discussion and short presentations highlighting different kinds of collaborations and different collaboration skills, followed by a small-group activity.

**Objective:** It is widely held that solving complex problems is done best by diverse teams that include experts from different social and disciplinary backgrounds. Bringing together an interdisciplinary team can expose the collaborators to new ideas, perspectives, or approaches they may not have considered before. Librarians can bring to the table a range of skills and expertise (e. g., access to information, sophisticated search strategies, organization of information, information literacy, the user experience, bibliometrics, research data management, instructional design). Therefore, the interdisciplinary playing field is a space where librarians can collaborate with non-librarians in many ways, including but not limited to: co-teaching a course, sharing a research project, grant writing; presenting at a conference, or providing community outreach. Some of the benefits of extra-library collaborations include the librarian's deeper sense of connection to the institution, and the non-librarian's greater appreciation of the librarian as a colleague and collaborator.

**Instructional Methods:** Participants will learn through a series of short presentations made by the panelists, followed by Q&A. Then participants will join in small-group activities to brainstorm ideas for collaboration, including the output of the collaboration and potential extra-library collaborators.

**Participant Engagement:** Participants will be encouraged to engage in the Q&A, and they will work in small groups that will be facilitated by one of the panelists, to brainstorm one or more collaboration plans to take home.

**Sponsors:** Veterinary Medical & Health Sciences Librarians Caucus

Research Caucus

Pediatric Librarians Caucus

Nursing and Allied Health Resources and Services Caucus

Tuesday, August 11, 3: 15 p.m.–4: 30 p.m.

## Innovation & Research Practice Immersion Session 3

### You Can Do It: Developing Your Research Identity within Health Sciences Librarianship

**Alexander J. Carroll, AHIP,** Librarian for STEM Research, Vanderbilt University, Nashville, Tennessee

**Ayaba Logan,** Research and Education Informationist, Medical University of South Carolina, Charleston, South Carolina

**Laura Menard,** Assistant Director for Medical Education and Access Services, Indiana University, Indianapolis, Indiana

**Lisa A. Marks, AHIP,** Director, Library Services, Mayo Clinic Libraries-Arizona, Scottsdale, Arizona

**Elizabeth Laera, AHIP,** Medical Librarian, Brookwood Baptist Health, Birmingham, Alabama

**Session Format:** This session will feature a moderated panel discussion of 3 health sciences librarians; after the panel portion, session attendees will have the opportunity to respond to this panel discussion by participating in small group discussions at their table.

**Objective:** Our goal is to convene an Immersion Session that will facilitate a conversation among members of MLA who are interested in increasing their involvement in research activities. To accomplish this, we have recruited a small panel of 3 health sciences librarians who are actively engaged in research. These librarians represent different stages in terms of career and research accomplishments (early-career, mid-career, senior), reflect the diversity of MLA’s membership, and have experience working in a variety of health sciences library settings. Topics discussed by the panel will include “Developing a Research Agenda,” “Research Methods and Study Designs,” and “Creating Space for Research as a Practicing Librarian.”

**Instructional Methods:** Our panelists will share short presentations and participate in a moderated Q&A, consisting of questions from the session moderator as well as questions submitted in real-time by attendees using the conference app. Following this large group portion of the session, we will then adjourn to small group discussions, in which audience members are given the opportunity to react to guided questions and to interact with the panelists who will float between the different tables. Following these small group discussions, small groups will report out their responses to the larger group, spurring additional conversation among the panelists and audience.

**Participant Engagement:** During the small group discussions, attendees will be given a guided question to respond to, and attendees at each table will have the opportunity to volunteer for one of four roles (facilitator, note taker, time keeper, and reporter) designed to promote productive conversations. The facilitator will guide the table’s discussion, the note taker will write down points of discussion, the time keeper will monitor the clock, and the reporter will share out the group’s responses to the larger group after the small group discussion.

**Sponsors:** Research Caucus (formerly the Research Section); Technology in Education Caucus (formerly the Educational Media and Technologies Section); Hospital Library Caucus (formerly the Hospital Library Section)

Wednesday, August 12, 10: 15 a.m.–11: 30 a.m.

# Information Management Immersion Session 3

## Collection Management in the Age of Hospital Mergers

**Elizabeth Laera, AHIP,** Medical Librarian, Brookwood Baptist Health, Birmingham, Alabama

**Aidy Weeks, AHIP,** GME Liaison Librarian & Collections Manager, UNLV, Las Vegas, Florida

**Heather J. Martin, AHIP,** Director, System Library Services, Providence, Portland, Oregon

**Jean Gudenas, AHIP,** Director of Information Resources and Collection Services, Medical University of South Carolina, Charleston

**Angela Spencer, AHIP,** Health Sciences Librarian, Saint Louis University, St. Louis, Missouri

**Session Format:** The session will feature a panel of 3 speakers who will present on a specific issue and share their own experiences as a case study, with at least 30 minutes of Q&A including audience participation.

**Objective:** This session looks to build on a previous successful session on hospital mergers by focusing on the pressing issue of collection management in hospitals and academic settings involved in institutional mergers. Presenters will address collection management policies, finances, licensing, and working with vendors, navigating tricking access issues and site-specific collections, as well as experiences from the academic side. Participants are encouraged to bring their own experiences and share advice, pitfalls, and pearls with the room.

**Instructional Methods:** Using a method commonly seen in clinical grand rounds or morning reports, panelists will present a case study featuring the collection management details in their own mergers. Each panelist will then provide details about the various concepts addressed in the case and illustrate them with examples from their own experiences and the literature. After the presentations, the panelists will answer questions from the participants; however the participants are encouraged to answer each other’s questions and share advice and their own experiences.

**Participant Engagement:** Participants will be encouraged to ask panelists questions, but also to respond to their fellow audience members’ questions with their own experiences. Panelists can elaborate on their own experiences, but the goal is for the participants to share lessons learned and pearls of wisdom so that everyone leaves with multiple ideas to implement.

**Sponsors:** Hospital Libraries Caucus

Collection Development Caucus

Wednesday, August 12, 10: 15 a.m.–11: 30 a.m.

# Information Management Immersion Session 4

## Concentric Conversations: A Data Curation and Reuse Unconference

**Peace Ossom Williamson, AHIP,** Director for Research Data Services, University of Texas at Arlington, Arlington, Texas

**Virginia (Ginny) Pannabecker, AHIP,** Director, Research Collaboration & Engagement; Life Sci & Health Sci Liaison, University Libraries, Virginia Tech, Blacksburg, Virginia

**Session Format:** This will be an unconference in a “concentric conversations” format - an enlarged fishbowl that incorporates strategies for inclusiveness. This session has been updated for the vConference. Changes for the online version are reflected in the Agenda and Topic Suggestion Form.

**Agenda:** https: //docs.google.com/document/d/1jExhw8CZAxs3h_H461F-V0Oag6sQsEUf-qVed85xmto/edit?usp=sharing

**Topic Suggestion Form:** https: //tinyurl.com/DataReuseTopics2020

**Objective:** Share your thoughts and practices in this unconference where open discussion will take place around the various processes that exist and what necessary techniques should be used for assessing the quality of data for curation and for reuse. As more libraries provide services covering more of the research data lifecycle, an increasing number of checklists, toolkits, and resources abound for the process of assessing data quality for curation and for reuse. Therefore, the aim of this immersion session is to bring people of various experiences together to find new or common ground around best practices for assessing data quality and in engaging in data curation. This unconference will allow ideas and practices to be shared where librarians providing these services, working as data researchers, or engaged in related areas, can provide input on how they are incorporating these tools, while the informal nature of the unconference will allow varied levels of participation, from listening and asking questions to sharing practices and presenting briefly.

**Methods:** The participants will sit in two or more rows of concentric circles, seats facing inward, as an expanded fishbowl; there will be two notetakers strategically placed in the room to record the conversation that takes place in a shared open Google Doc. The facilitators will provide an overview of the session (5 mins) and the theme of assessing and ensuring data quality for both curation and reuse along with three examples of conversation topics that fit within the theme (10 mins). Participants will then supply other conversation topics (5-10 mins), and these will be saved to be discussed in focused conversations (60 mins). The immersion session will end with a recap and information about how to access and contribute to the shared notes (5 mins).

**Participant Engagement:** Those sitting in the center will discuss, and those in outer rings can observe and ask questions or switch out with those in the center so they can share about a particular topic. Participants in the center will be encouraged to contribute to the discussion or allow others to switch to keep the conversation going. Anti-oppressive facilitation (https://publiclab.org/system/images/photos/000/020/664/original/Aeorta_facilitation_resource_sheet.pdf) will be implemented to make it an engaging experience for everyone.

**Sponsors:** PH/HA Caucus and Data Caucus

Wednesday, August 12, 11: 30 a.m.–3: 00 p.m.

# Education Session 5

## Moderator: Jessica Sender, AHIP, Focusing on Faculty Facilitator Needs for Small Group Case-Based Learning: Where Might Librarians Fit in?

**Gail Kouame,** Assistant Director for Research & Education Services, Augusta University, Augusta, Georgia

**Julie K. Gaines, AHIP,** Campus Director, AU/UGA Partnership Library, Augusta Univ/Univ of GA Medical Partnership, Athens, Georgia

**Objectives**: The hypothesis for this study is that increased librarian involvement in case-based small group learning will improve faculty facilitators’ assessment of students’ use and citation of information resources in learning objective presentations. Our aim is to determine a role for librarians as collaborators to improve student use of high quality evidence-based information resources for small group learning.

**Methods**: This presentation describes findings from a survey administered to faculty facilitators for small group case-based learning for undergraduate medical students. The aim of the survey was to identify gaps in knowledge and/or skills on the part of faculty facilitators surrounding information resources, and to ascertain whether they perceive of librarian participation as valuable for small group case-based learning.

A link to an online Qualtrics survey was sent via email to faculty facilitators on two campuses (N = 70). The survey was composed of 20 questions, including Likert scales and narrative responses. Selected topics included: comfort in use of library resources, confidence determining appropriate resources to answer clinical and basic science questions; top information resources both facilitators and students consult; whether facilitators review and give feedback about students’ citations of resources; and whether or not they see value in librarian participation.

**Results**: There was a 68% response rate (n=48) to the survey. The majority of respondents had 2 or more years of experience as facilitators. A high percentage felt somewhat or extremely comfortable using library resources and expressed high levels of confidence in determining appropriate information resources to answer clinical or basic science questions. Results indicated that there was a wide variety of information resources used by both faculty and students. While over half of the respondents stated they have not consulted or collaborated with a librarian, 83% see the value of librarian participation in small group case-based learning.

**Conclusions**: Faculty facilitators see value in librarian participation in small group case-based learning. While faculty facilitators say they provide feedback to students on their use of information resources, librarians can provide additional expert advice about appropriate resources to use, and how to cite them properly. Facilitators indicate that students’ use of information resources should be included as part of small group case-based learning evaluations. Future librarian participation could include assistance with on-the-fly fact finding during small group sessions, offering orientation sessions for new faculty facilitators, and promotion of librarians as resources for small group case-based learning.

## Demonstrating Progress in Question Formulation Skills Training among First-Year Medical Students

**Jonathan Eldredge, AHIP,** Associate Professor, HSLIC, University of New Mexico, Albuquerque, New Mexico

**Melissa Schiff**

**Jens Langsjoen**

**Objectives**: Booth indicates that question formulation drives the entire Evidence-Based Practice process. A 2010 systematic review by Horsley et al. determined a lack of conclusive empirical evidence for any method of training in question formulation. This study sought to measure the degree of improvement in student performance in question formulation skills through training and the use of a new rubric.

**Methods**: Quasi-Experiment. Students beginning the course were presented with a clinical vignette and asked to formulate a question based on that vignette. Two weeks later they were trained in question formulation skills in a one-hour session coupled with a one-hour searching skills lab. Students then were post-tested on the same clinical vignette and asked to formulate a question. Students were assessed by three faculty instructors using the rubric that had been incorporated into the training. This rubric measured student performance on question formulation for both the pre- and post-test.

**Results**: We will report our results during February 2020. We anticipate that students will improve their question formulation skills as measured using the same rubric by at least 20%. We expect to use a two-tailed t-test statistical analysis to compare the pre- and post-test scores on the same rubric.

**Conclusions**: Health sciences librarians frequently train students and practitioners on formulating answerable Evidence Based Practice questions. This study seeks to advance progress in training others on question formulation. Five years ago, we sought to improve on existing training protocols for question formulation through a multiple year trial and error development process primarily based on anonymous student evaluations. Our training of first-year medical students included the use of a rubric for student self-assessment. Previous anonymous student evaluations had rated the question formulation training highly. This study sought to measure the degree of improvement of student performance in question formulation skills.

## From Kitchen Sink to Rigor and Reproducibility: Refocusing a Library Skills Class

**Fred Willie Zametkin LaPolla,** Research and Data Librarian, Lead Data Education, NYU Health Sciences Library, New York, New York

**Alisa Surkis,** Assistant Director, Research Data and Metrics/Vice Chair for Research, NYU Health Sciences Library, New York, New York

**Background**: In 2016, our library was invited to teach a required 1-credit research skills class for first year PhD students in biomedical sciences, which included lessons on rigor and reproducibility, literature searching, data visualization, research data management, and other topics. In response to changing NIH requirements on rigor and reproducibility education for training grant recipients, we updated our curriculum for the 2019-20 academic year to be a focused course on Rigor and Reproducibility rather than a general skills class. Briefly, rigor is the scientific soundness of research, and reproducibility is if an experiment can be repeated with similar outcomes.

**Description**: Our team of librarians took relevant elements from our previous class and reframed them around Rigor and Reproducibility, as defined by the NIH and National Academies of Science. We also created new educational content focused on replicability (the ability to repeat a study with similar results) and computational reproducibilty (the ability to re-run analyses), with an invited speaker discussing authentication of biological resources. The class is a mixture of lecture, hands on activities and technology-facilitated discussion. Our goal was to provide a unified educational class to better meet learner needs, and student performance is assessed by summative and formative assignments, as well as in an attitudinal survey at the class's end. We compared the updated class with past years to understand if a focused class on Rigor and Reproducibility is better received than a multi-topic series.

**Results**: A majority of over 2/3 of students found the new rigor and replicability focused material moderately or very useful and indicated by their own self-assessment an increase in knowledge on class topics after the class. All students passed our final summative evaluation, and instructors reported feeling that the new class had fewer issues and students demonstrated a greater capacity to grapple with topics as compared to the old class offering.

**Conclusion**: While valid year-over-year comparisons between different classes are difficult to demonstrate by scores or evaluations, the new iteration of the course was well reviewed in terms of students' self-evaluation of learning and utility. Moreover, while the final exam changed, instructors felt learning was more apparent in the new Rigor and Reproducibility class. Instructors also felt they had a superior experience teaching the new Rigor and Reproducibility curriculum as compared to the old skills-based-class.

## The Methodological Maze: Creating a Workshop on the Process of Conducting a Scoping or Systematic Review

**Andrea Quaiattini,** Liaison Librarian, McGill University Library; Schulich Library of Life Sciences, Physical Sciences, and Engineering, Quebec, Canada

**Lucy Kiester,** Liaison Librarian, McGill University Library; Schuclich Library of Life Sciences, Physical Sciences, and Engineering, Quebec, Canada

**Background**: As the desire to conduct comprehensive knowledge synthesis projects such as systematic and scoping reviews continues to grow, librarians are called upon to provide support to researchers who are often unaware of the complexities of these reviews. Librarians are therefore positioned to be methodological experts, providing more than just search strategy development support.

Librarians must continue to evolve both in content and instructional design of workshops to reflect the changing landscape of review methodologies. We have implemented intentional pedagogical choices to maximize our response to the current needs of medical researchers.

**Description**: Designed for both students and faculty, our workshop guides participants through the entire review process, from question development to publication. Competencies and resources are identified (but not explicitly taught) throughout the workshop so participants gain an understanding of the review process and skills required to be successful.

Crucially, the workshop highlights the methodological differences between scoping and systematic reviews; key differences that are not always clear to researchers, which can have significant effects on the process and success of the project.

This presentation will provide an overview of the workshop structure, discuss our instructional design process using a structure which pointedly highlights the methodological differences between scoping and systematic reviews, and presents the in-workshop tools that were developed specifically for participants. This presentation will enable participants to adapt our format to address their unique scoping and/or systematic review instruction challenges.

**Conclusion**: Our assessment of the workshop has been iterative and ongoing. From colleague feedback, we expanded the scoping review content of our workshop. In the workshop itself, we received comments from participants expressing appreciation of the clarity of the methodologies we present in class. Moreover, we continue to actively collect feedback; part of being a good educator is consistently adapting and responding to learners developing and ongoing skillsets. As we look to the future and these methodologies continue to change, we believe this kind of multimodal workshop will come to take a permanent place in the research activities of scholars.

## Promoting the Work of Librarians through the Academic Pediatric Association Educational Scholars Program

**Elizabeth C. Whipple, AHIP,** Assistant Director for Research and Translational Sciences, Indiana University School of Medicine, Indianapolis, Indiana

**Caroline R. Paul,** Associate Professor, UW West Pediatrics Clinic, Madison

**Wendy L. Hobson-Rohrer,** Associate Vice President for Health Sciences Education, None

**Background**: The Academic Pediatric Association Educational Scholars Program is a 3-year national faculty development program for pediatricians. This program is designed to aid junior faculty early in their academic careers, with each scholar creating and completing a scholarly project to build their skills and a niche in educational scholarship and leadership. In 2014, a medical librarian and two pediatric educators collaborated to conceive, create and design a new module centering on the topic of literature searching. The module provides the junior faculty scholars with fundamental skills to conduct their projects.

**Description**: As an entrée into understanding the place of a project in the literature, the module authors created a new rubric to evaluate the quality of a manuscript’s introduction, background, and reference sections. The authors created and narrated nine narrated videos, including: 1) Turning Your Research Question/Project into Concepts, 2) Controlled Vocabulary, 3) Keyword Searching (MESH), 4) Inclusion and Exclusion Criteria, 5) Medical Education Databases and 6) Getting to the Full Text. After viewing the videos and completing a consultation worksheet, participants scheduled a meeting with their local medical librarian for further help with the development of their project. Next, participants viewed three videos focused on understanding the impact of their scholarly work (Impact Factors, H-Index and Citation Counts, Altmetrics, and Educational scholarship and promotion). Finally, scholars performed a guided reflective critique of their scholarship moving forward.

**Conclusion**: Currently, three cohorts of junior faculty scholars have completed the course. The rubric aided participants to be objective when evaluating papers and provides a tool for use in future manuscript evaluations. While the videos provided context and content, the scholars repeatedly mentioned the most valuable part of this module being the actual meeting with their local medical librarian. The librarian taught the junior faculty about specific databases and nuances of searching, making writing an introduction more manageable. Several participants now consider their librarians as part of “their team” and are working for future collaborations in publishing together.

Wednesday, August 12, 11: 30 a.m.–3: 00 p.m.

# Information Services Session 2

Moderator: Irma Singarella

## Empowering Community Health Workers to Provide Health Information to Hispanic Community Members

**Dana L. Ladd, AHIP,** Health and Wellness Librarian, Health and Wellness Library, Lanexa, Virginia

**Emily J. Hurst,** Interim Director and Associate Dean, Tompkins-McCaw Library for the Health Sciences, VCU Libraries, Richmond, Virginia

**Background**: Librarians from the Tompkins-McCaw Library for the Health Sciences and Health and Wellness Library collaborated with staff from La Casa de la Salud, a non-profit organization to create and conduct a consumer health information training program that will contribute to the reduction in health disparities of Hispanic people in the community.

**Description**: Two health sciences librarians and staff from La Casa de la Salud developed and implemented a program to provide health information resources in the Spanish language to Hispanic community members. The librarians designed a one hour training session for community health workers. The training detailed how to find reliable consumer health information resources applicable to the Hispanic community in the Spanish language and the librarians demonstrated reliable websites and videos. Identified Spanish language consumer health resources were added to the non-profit organization's website and community health workers were then able to train community members on how they can find information to meet their health information needs. Training workshops were designed and provided in English with support from a Spanish speaking translator.

**Results** Two workshops were conducted for La Casa de la Salud community health workers. The first workshop was conducted at the Health and Wellness Library and twelve people attended. The second workshop was held at Vida Nueva church and sixteen people attended. Feedback from written evaluations was very positive about the programs and most reported they learned about new resources and planned to start using at least one resource they learned about.

**Conclusion**: This collaboration allowed the Health and Wellness Library the opportunity to provide awareness of its services and resources to the local Hispanic community.

## National Health Observances: Content to Promote Health Information Resources and the All of Us Research Program

**Elizabeth J. Kiscaden, AHIP,** Medical Librarian, University of Iowa, Iowa City, Iowa

**Brittney Thomas,** Manager, NNLM All of Us Community Engagement Center, The University of Iowa, Iowa City, Iowa

**Lydia N. Collins,** Participant Engagement Lead, NLM Training and Education Center, Pittsburgh, Pennsylvania

**Background**: In 2017, the National Library of Medicine was selected as a community partner for the NIH All of Us Research Program. As a part of this collaboration, the National Network of Libraries of Medicine (NNLM) established the NNLM All of Us National Program which has a goal of increasing consumer access to quality health information. One objective to achieve the goal of the NNLM All of

Us National Program was the creation of health information content for public libraries tied to health observances selected by the All of Us Research Program.

**Description**: The observances for the project were selected to align with priority health topics of the Program. The project was challenging in that the timeline required the release of content immediately, with little opportunity to first evaluate what type of material would be valuable and easiest to implement by public libraries. Given this challenge, the project team developed content in a variety of formats to pilot, such as program kits, social media tools, webinars, and handouts, which were released during the first six months of the project. After this content was released, an assessment was administered to all NNLM public library members. The assessment resulted in over 150 responses which provided data used to revise existing content and guide the development of remaining content for the project.

**Conclusion**: During the first six months of the project, content created for the project was accessed over 40,000 times. Public library staff who responded to the assessment found most of the content easy to use and agreed that it helped to raise public awareness of National Library of Medicine resources. Public library staff indicated that some health observances resonated better than others, and offered suggestions for topics for future consideration. While the content is promoted monthly through national public library partners, awareness among public library staff is low and the project will require consistent marketing to be successful.

## Understanding the Health Information Practices of LGBTQ+ Communities to Improve Medical Librarian Services

**Travis L. Wagner,** Doctoral Candidate, University of South Carolina

**Nick Vera, ,**PhD. Student, University of South Carolina, Columbia, South Carolina

**Vanessa Kitzie**

**Objectives**: This multi-method, three-year qualitative study addresses the following research questions: (1) How does socio-cultural context shape the information creation, seeking, sharing, and use of health information among LGBTQ+ communities? (2) How can these findings inform medical librarian services to LGBTQ+ communities for health promotion?

**Methods**: Data collection consists of 30 individual semi-structured interviews with LGBTQ+ community leaders from STATE (completed), 6-8 focus groups with leaders' communities (in progress), and a community forum informed by the World Café methodology between 30-40 leaders and librarians (in progress). Individual and focus group interview participants also engaged in information worlds mapping, a visual arts-based elicitation method. Data for analysis are verbatim transcripts, analytical memos of information worlds maps, community forum notes, and researcher field notes and reflexivity journals. Data analysis follows qualitative open coding and constant comparison methods. Line-by-line first-cycle process coding identifies initial codes, which the researchers compare, combine, and refine via subsequent data collection and analysis. Second-cycle axial and theoretical coding informs development of a conceptual model that describes key coding categories and the relationships between them. Peer debriefing and participant member-checking serve as validity checks.

**Results**: Preliminary interview findings invert deficit models of LGBTQ+ health and information practices. These models position communities as lacking resources and knowledge to improve their social conditions and envision experts as able to “correct” this deficit. Participants challenged these presumptions by identifying social and structural factors, including experts, as hindrances to

achieving positive health outcomes, and tactically responding to these constraints. For example, several communities stated that being misgendered at the doctor’s office took a significant toll on their mental health. They responded to this lack of expert competency by developing lists of community-approved medical professionals based on collective information assessment.

**Conclusions**: Inverting the deficit model to view experts rather than LGBTQ+ communities, as lacking has implications for social and structural change. From the position of medical librarianship, this change can occur via a shift from outreach, which focuses on information and resource provision, to engagement, which centers community expertise as the driver for information and resource development. Three specific implications informed by this shift and emergent research findings are establishing partnerships with community health workers, facilitating cultural competency training for medical professionals, and offering harm reduction workshops.

## Virtual Focus Groups: Bringing Public Library Workers Together for Consumer Health

**Catherine A. Smith,** Professor, The Information School, University of Wisconsin-Madison, Stoughton, Wisconsin

**Objectives**: To understand the challenges and opportunities for public library workers that arise around health information in the 21st century. POPULATION: 57 public library workers from across the United States. 56% were professionals; 11% held jobs focusing on children and young adults. 42% were in suburban settings, 35% in cities, 14% rural and 9% in towns.

**Methods**: Focus groups allow researchers to explore ideas and insights from a small group of people, using scaffolded questions to keep the participants focused on the topic at hand. For this study, the investigator conducted a series of virtual focus groups using Web-based GoToMeeting (LogMeIn.com). A total of 57 participants met in groups of 2-5 between March and May 2018. Sessions were recorded, transcribed and coded using NVIVO (QSR).

**Results**: Multiple themes were discussed; this presentation focuses on the answers to one key question: "“What is the biggest challenge you see for public library workers who get health information questions?” Participants identified a range of challenges; the five most frequently discussed were the public’s need for medical Advice, their Expectations and role Boundaries for library workers, their Expertise (or lack of it), and the need to Evaluate information. Computer literacy, Health literacy for both patron and librarian, and general literacy were also areas of need. Finally, the need for training in health information resources was a recurring theme.

**Conclusions**: The public's need for health information is one of the oldest recorded in public libraries. To best support the public in its pursuit of health, library administrators and library science educators alike need to join hands across the aisle and begin educating library workers at all levels in the best ways to meet these needs. The virtual focus group methodology proved to be a valuable way to explore the values and the characteristics that library workers in all settings, from major metropolitan areas through villages, have in common.

## Focus on Outreach: A Pop-Up Library Commemorating Florence Nightingale’s 200th Anniversary in 2020

**Dana Gerberi, AHIP,** Librarian, Mayo Clinic, Rochester, Minnesota

**Julie M. Taylor,** Outreach Librarian, Mayo Clinic, Rochester, Minnesota

**Background**: Pop-up libraries have been a trending form of outreach for public and academic libraries the past several years but are still a novel concept in clinical and hospital settings. Engaging with healthcare staff in common spaces with an inviting temporary display provides an opportunity to proactively raise awareness of library resources and services to nontraditional users while also piquing interest in a timely topic or special theme. We describe how a pop-up library was devised and implemented as a unique form of outreach at a main campus of an academic medical center.

**Description**: While spontaneity is an outwardly appealing feature of a pop-up library, a key step involves thorough planning. To reach diverse staff, various high-traffic locations were identified and enticing exhibit items were gathered. Florence Nightingale was selected as the theme for our pop-up library trial to celebrate the 200th anniversary of her birth in 2020 and educate staff about her enduring legacy on modern healthcare. The display includes relevant books and DVDs from the collection, a life-size cut out of Florence Nightingale, a poster highlighting her significant achievements, customized giveaway bookmarks, brochures, interactive trivia combined with an evaluation survey to measure overall impact, a prize-drawing and a laptop for live demonstrations. Two librarians managed the mobile display during busy lunch hours on scheduled dates in early 2020.

**Conclusion**: Our goal was to learn if a pop-up library is successful in promoting the library to hospital and clinical employees who may not use traditional library spaces or even the library website. Using simple evaluation methods, we tracked the number of meaningful interactions at the pop-up display and assessed whether participants gained new information about resources and services offered by the library. Though the Covid-19 pandemic disrupted a number of events, we interacted with over 200 employees during 10 pop-up displays and perceived this method of outreach to be effective for increasing library exposure and visibility.

Wednesday, August 12, 11: 30 a.m.–3: 00 p.m.

## Innovation & Research Practice Session 2

Moderator: Roland Welmaker, Sr.

### Understanding Participant Motivation and Rates of Attrition in Biomedical Datathons and Hackathons

**Bethany S. McGowan, AHIP,** Assistant Professor of Library Science and Health Sciences Information Specialist, Purdue University Libraries and School of Information Studies, West Lafayette, Indiana

**Objectives:** Biomedical data competitions are interdisciplinary live or virtual competitive events that require participants to analyze big biomedical data and develop prototype solutions to real-world problems that accelerate innovation of medical applications, improve healthcare technology design, and help streamline healthcare business models. These competitions, when guided by librarian instruction, support participant's understanding of research data management and health data literacy-related competencies. Participation in biomedical data competitions helps participants identify, consider, and develop solutions to challenges associated with how big biomedical data influences patient well-being. Data competitions are particularly useful for highlighting issues associated with the underuse of uniform data standards and issues associated with navigating siloed data. Long-term data competitions allow participants to submit their work in iterative phases and to receive feedback throughout the hacking process. This approach welcomes beginners and attracts participants who might not participate in traditional data competitions. However, these competitions have high attrition rates. As librarians continue hosting long-term datathons and hackathons, we seek to understand participant motivation and attrition factors.

**Methods:** We hosted 3 three-week-long data competitions in the academic year 2019-2020. One event was team-based, with each teach assigned a team leader; the other two required participants to work individually. Registration for these events included a pre-assessment and a demographic analysis. At the end of each week, participants checked in and received project feedback from judges. In-person interviews were conducted at the end of each competition.

**Results:** Reported factors that influenced attrition included lack of time, lack of ability, and lack of understanding. These factors were most commonly reported amongst participants competing in individual competitions. Retention rates, reported learning gains, and participant motivation greatly improved for individuals competing in the team-based challenge, where the team leader was often cited as a mentor and important to the team's success

**Conclusions:** Findings suggest that retention in long-term data challenges is improved by a team-based model, as opposed to an individual model. We plan to further test these findings in AY 2020-2021 by hosting and comparing individual and team-based challenges. Findings will inform the creation of an OER toolkit to support librarians in planning inclusive data competitions. We will also explore another concept revealed by this study, the influence of the team leader as a mentor. This presentation is supported by the Network of the National Library of Medicine Greater Midwest Region under cooperative agreement number 1UG4LM012346. The content is solely the responsibility of the author.

## Is the Open Access Citation Advantage Real? A Systematic Review

**Caitlin Bakker, AHIP,** Research Services Librarian and Medical School Liaison, University of Minnesota, Minneapolis, Minnesota

**Amy L. Riegelman,** Social Sciences Librarian, University of Minnesota, Minneapolis, Minnesota

**Allison Langham-Putrow,** Scholarly Communications Librarian, University of Minnesota Libraries, Minneapolis

**Objectives**: The potential for open access (OA) publication to increase citation rates of articles was first articulated in 2001. Since then, support for and refutation of the OA citation advantage has been abundant. OA’s influence on citation remains unclear, particularly across disciplines, data sources, and methodological approaches. This systematic review aims to determine if the OA citation advantage is real.

**Methods**: We conducted a systematic search of the literature in accordance with MECIR standards to identify all publications that compared citation rates of OA and non-OA publications. We executed this search across seventeen databases representing a broad range of disciplines. Title and abstract screening, full-text screening, data extraction and risk of bias assessment were completed by two independent reviewers and discrepancies were resolved through consensus or by a third party where necessary. We extracted data to describe both the exposure (OA) and control (non-OA) groups, including number of included studies, as well as cumulative citations and data source of citations. We also recorded how open access was defined, how samples were identified, and the citation window considered. Risk of bias assessment was completed to assess underlying methodological quality of the component studies.

**Results**: With duplicates removed, we screened 2,108 titles and abstracts. 1866 items were removed at this phase, leading to 242 full-text articles being assessed and ultimately 115 items being included in qualitative synthesis. These articles represented a broad range of disciplines, data sources, and outcome measures. Data extraction also uncovered notable issues with incomplete reporting. 54 of the included studies reported an open access citation advantage while 28 reported an advantage in subsets and 32 reported no citation advantage. 1 study reported inconclusive results. Risk of bias assessment and quantitative synthesis are currently underway.

## Examining Open Access Article Performance: Taking a Nearsighted Approach to a Farsighted Problem

**Roxann W. Mouratidis, AHIP,** Head of Scholarly Communications, FL. State University, Tallahassee, Florida

**Martin Wood, AHIP,** Director, Florida State University, Tallahassee, Florida

**Objectives**: To determine if open-access journal articles receive more citations, downloads, and social media attention than toll-access articles in the health sciences.

**Methods**: Using our university’s CV database, we generated a list of faculty names and articles published between 2013-2016. The following criteria were used to refine this list: 1) the faculty member must have an active appointment in the College of Medicine, and 2) the publication must be a scholarly journal article.

After our master list was generated and refined, we identified the open access articles by looking up each journal in the Directory of Open Access Journals, and in the case of hybrid journals, viewing the article’s access options on the journal’s website.

We then manually retrieved article level metrics for each article on our list. These metrics included altmetric scores and the number of citations, downloads, and views each article received. Our primary source of data was the journal’s website. Secondary sources of data included Dimensions and the Altmetric bookmarklet.

**Results:** On average, articles published open access or available in the institutional repository received at least a 20% increase in number of citations, as compared to articles published exclusively behind a paywall.

**Conclusions**: Formal conclusions will be shared at the meeting; however, we anticipate that open-access articles will receive more citations, downloads and/or views, and higher altmetric scores on average than their toll-access counterparts. As such, authors and their institutions or funding agencies would benefit from utilizing open access publishing venues to increase the visibility of their research. Libraries have a role in facilitating open access to research by establishing institutional repositories as an alternative venue for freely sharing faculty-authored publications.

Wednesday, August 12, 11: 30 a.m.–3: 00 p.m.

# Lightning Talks 3

Moderator: Michael S. Fitts

## Making the Rounds: A Clinical Librarian in the Intensive Care Unit

**Lindsay M. Boyce,** Research Informationist II, Memorial Sloan Kettering Cancer Center, New York, New York

**Background**: The role of the clinical librarian (CL) is to provide patient-centered information to clinicians at point of care. The CL attends bedside rounds, weekly reports, and conferences, and provides evidence-based information and literature in real-time. There is very little literature looking at the role and experience of a clinical librarian within the intensive care unit (ICU). The purpose of this lightning talk is to describe the role, experience, and value of a CL embedded in an intensive care unit.

**Description**: For almost two decades our library has been collaborating with clinical units and involving embedded CLs throughout the hospital. Since 2009, a CL has been the primary library point person for all clinicians in the ICU. Currently, the author attends bedside rounds once a week to complement the services already provided to the critical care team. The CL addresses questions that arise during case discussions, and proactively provides information to the clinicians on topics being discussed. The librarian is equipped with a smart phone and provides professionally vetted high-quality information within minutes using PubMed and a variety of point of care mobile applications.

**Conclusion**: The embedded CL provides invaluable support and resources to the critical care team. In multiple cases the information provided by the CL to the critical care team led to a change in a patient’s treatment plan or widened the circle of possible diagnoses to be assessed. With the support of library and hospital administration, creating a CL for the ICU is a vital asset to both the library and its parent institution.

## Focusing on Success: Librarian Support of a Collaborative Multi-Institutional Nursing Evidence-Based Practice Fellowship

**Sola Whitehead,** Medical Librarian, VA Portland Health Care System, Portland, Oregon

**Basia Delawska-Elliott, AHIP,** Health Sciences Education and Research Librarian, Oregon Health & Science University, Portland, Oregon

**Margo Halm,** Associate Chief Nurse Executive, VA Portland Health Care System, Portland, Oregon

**Deborah Eldredge,** Quality, Research & Magnet Director

**Background**: In 2011, an academic medical center initiated a nursing evidence-based practice fellowship. Clinical nurses identified projects on their own unit and learned how to conduct a full evidence-based practice change following the Johns Hopkins model. In 2015, the program expanded to include instructors and participants from an affiliated hospital. In its 8th year, this ongoing program conducted by nurse leaders and librarians from two affiliated institutions supports Magnet accreditation and evidence-based practice change.

**Description**: In support of clinical nurses and Magnet accreditation at two institutions, the Nursing Evidence-Based Practice Fellowship is a collaborative multi-institutional program guiding nurses

through self-identified evidence-based practice projects from November through the following June each year. Health sciences librarians support the program by providing instruction on searching the literature, identifying qualitative and quantitative studies, managing citations, appraising and synthesizing the literature, and disseminating the work at the end of the project through poster and podium presentations. The librarians additionally meet with fellows for individual coaching on literature searching.

Following successful fellowship experiences, fellows have disseminated learning and results at regional and national conferences and taken on additional evidence-based projects. The librarians have observed an increase in nurse confidence regarding literature searching and EBP project management. Program results have led to improved patient experience and clinical care, and cost efficiencies.

**Conclusion**: Evidence-based nursing programs can be powerful tools to support development of nursing research, mentoring, and leadership skills. As members of interdisciplinary instruction teams, librarians play a pivotal role in that success. Combining programs of two institutions, and having librarians that represent each institution, further drives the success of the program by offering expanded perspectives and collaborative instructions for all participants.

## Building a Partnership with Bariatric Surgery to Educate and Support Patients

**Kathy Koch, AHIP,** Librarian, Advocate Aurora Health, Grafton, Wisconsin

**Background**: Obesity affects 93 million adults in the US, and an estimated 252,000 had bariatric surgery in 2018. Recent articles in obesity surgery journals found a lack of high-quality online information for patients considering surgery. To help fill this knowledge gap, a hospital library collaborated with the bariatric surgery program to build a consumer health book collection to educate and support bariatric surgery patients. These books focus on both pre- and post-surgical information needs and supplement information provided during clinic appointments and at support group meetings.

**Description**: In May 2018 the bariatric surgery physician assistant (PA) met with the librarian to discuss library services and resources for her patients. A small collection of bariatric surgery books from the library network was supplemented by purchasing six newer titles. In the following months the collection was promoted at a support group meeting, and a recommended reading list was developed and published in the patient newsletter. Use of the collection was sporadic in the first year. In June 2019, a patient handout with library information and a list of available books was created for the PA to give to patients during their appointments. To increase awareness of the collection, the reading list and library information was included in the patient preoperative booklet. Additional books were purchased over the last 12 months, including second copies of popular books.

**Conclusion**: At the end of the first year, circulation was 16. Through ten months of the year two, circulation increased 150% to 40. The popularity of the collection presented the opportunity to explore other information needs the patients have throughout their surgical journey. An expansion of the collection to include topics such as emotional eating and mindfulness is under consideration. In order to keep circulation steady, routine contact with both patients and the patient care team is needed, such as promoting the collection at support group meetings and updating the team on successes and challenges.

## Coordinating Liaison Services: A New Role to Enhance Information Services

**Rose L. Turner,** Coordinator of Liaison Services, University of Pittsburgh, Pittsburgh, Pennsylvania

**Background**: Liaison librarians serve as the organizational backbone of the information services department at many academic health sciences libraries. Liaisons facilitate communication and outreach between a school or service and the library, but what happens when communication is lacking between librarians? Traditionally liaison services at our library have been siloed, with one librarian responsible for one school/department or service (molecular biology, data services, and scholarly communications). Diverse levels of experience, communication barriers, and incoming new librarians added additional challenges. An innovative solution was required to support collaboration to integrate library services.

**Description**: In late 2018 library administration created the new position of Coordinator of Liaison Services to provide leadership to the library’s liaison program. The main responsibilities of the role are to facilitate communication and knowledge sharing between liaisons and develop best practices for integrating library services and outreach. The Coordinator is also responsible for organizing assessment of liaison services. The coordinator began by facilitating bimonthly informal liaison “teas”. The informal gatherings provided a space to share challenges and opportunities faced by liaisons. As the group evolved, new methods of meeting design were employed to increase communication. Ultimately, an update of the library’s strategic plan led to the self-identification of priorities to work on as a group. These priorities have set the agenda for the year and have resulted in multiple successful collaborative projects among the librarians.

**Conclusion**: Identifying one person as the Coordinator of Liaison Services has resulted in improved information sharing and dissemination. The coordinator also serves as a proxy for the needs of the liaison department, acting as a single point of contact for those in other library departments who have general questions about services. This lightening talk will include examples of the successful projects completed by the liaison group over the past 18 months.

## Adherence to PRISMA Reproducible Search Strategy Requirements for Systematic Reviews

**Angela C. Hardi, AHIP,** Clinical Librarian, Washington University School of Medicine, St. Louis, Missouri

**Laura Elizabeth Simon,** Clinical Librarian, Washington University School of Medicine, St. Louis, Missouri

**Lauren H. Yaeger,** Medical Librarian, Washington University in St. Louis School of Medicine, Saint Louis, Missouri

**Michelle M. Doering,** Clinical Librarian, Washington University School of Medicine, St. Louis, Missouri

**Objectives**: PRISMA has been widely adopted as a standard for reporting systematic review findings. Item eight on the PRISMA checklist requires authors to “present [a] full electronic search strategy for at least one database…such that it could be repeated.” Our study aims to evaluate the reproducibility of search strategies reported in systematic reviews where PRISMA guidelines were reportedly followed.

**Methods**: A search was executed in Ovid-Medline to find systematic reviews that included the term “PRISMA” in the title or abstract. Search results were limited to 2010-2019 and English; 4815 results were retrieved. Two hundred citations were selected for testing the inter-rater reliability of our search reproducibility assessment tool. The assessment tool consists of seven questions that help to

determine if a reproducible search strategy is present in the systematic review. Inter-rater reliability testing was conducted using four reviewers.

A sub-analysis of a selection of the 200 citations used for inter-rater reliability testing was conducted to see how many of the articles included reproducible search strategies. This provided some initial results to review and aided in determining if continuing with the larger study was necessary.

**Results**: Using Fleiss Kappa, it was determined that each of the seven items of the search reproducibility assessment tool had good-excellent agreement (ranging from .772-.900). The assessment tool can reliably be used to rate the reproducibility of searches for the remaining 4615 citations.

Out of the 200 citations used for IRR testing, 125 had coding agreement from 3 out of 4 reviewers on all seven questions and were used for a sub-analysis. Out of these 125 only 52 (41%) included an electronic search strategy and only 32 (25%) included reproducible search strategies. These findings indicate that the larger study is warranted.

**Conclusions**: Though the larger research project is not complete yet, our initial findings demonstrated that it is very likely that systematic reviews that report using PRISMA standards are not actually including reproducible search strategies. By developing an assessment tool and methodically coding the articles, we can begin to show evidence that authors are not adhering to this PRISMA reporting checklist item. Systematic review authors may be contributing to the larger problem of the lack of reproducibility in science. It is also more difficult to assess the quality of a systematic review if adequate information about search methods is not reported.

## Health Sciences Librarians Mentoring Master of Public Health (MPH) Students: A Pilot Project for Community Health

**Jessica A. Koos, AHIP,** Health Sciences Librarian/Senior Assistant Librarian, Stony Brook University Health Sciences Library, Stony Brook, New York

**Jamie Saragossi,** Head of the Health Sciences Library, Stony Brook University, Stony Brook, New York

**Background**: It has been demonstrated that social work interns have successfully provided social services in a public library setting. To build on this premise, academic health sciences librarians partnered with the Master’s Program in Public Health (MPH) to develop a pilot program in which MPH students were trained to deliver consumer health information in public libraries within high needs communities. Nursing students and social welfare students were also part of this program, and they provided services to the community that were directly related to their respective areas of expertise.

**Description**: The health sciences librarians provided training and mentorship to MPH students, so they could proficiently provide health information to patrons in public libraries in areas identified as having health disparities. The MPH student was placed in these libraries to answer health related questions from patrons, using the resources available through the health sciences library, as well as freely accessible consumer health materials. The health sciences librarians also provided health literacy instruction to the nursing and social welfare students to ensure that they could provide accurate and reliable health information to patrons. The students also encouraged to create and deliver health-related programming. A data capture form was completed after each patron contact to identify specific health concerns within the community and to inform future programming and outreach efforts. This experience fulfilled practicum requirements for the MPH student, clinical hours for the nursing students, and contact hours for the social welfare students..

**Conclusion**: The success of this pilot program was evaluated using several measures, including the number of patrons served and feedback from hosting library directors. There have been approximately 300 patron contacts, and overall the library directors are pleased with the success of this program. Students are currently delivering virtual presentations to library patrons on a variety of health topics in place of on-site services, which were suspended due to the COVID-19 pandemic. There are currently plans to continue this program into the fall, including offering virtual one-on-one consultations to patrons as well.

## Duplicate Detection of Search Results for Systematic Reviews: An Evaluation of Covidence and EndNote

**Lily Y. Ren,** Research Communications Librarian, Lane Medical Library, Stanford School of Medicine, Stanford University, Mountain View, California

**Objectives**: One essential, yet time-consuming, phase in the systematic review process is identifying duplicate citations as searches are conducted comprehensively across multiple bibliographic databases. Although reference management programs contain algorithms designed to identify and remove duplicate records, literature suggests inconsistency in its success. This study seeks to evaluate the effectiveness of the duplication function in the commercial programs, EndNote and Covidence.

**Methods**: A sample systematic review search string is designed and translated in the databases Embase (Elsevier) and MEDLINE (Ovid) with a total of 4070 records extracted. Three duplication methods are applied to test the effectiveness of the duplication functions. First, the auto-duplication function in EndNote is used. Second, the systematic EndNote duplication method developed by Bramer et al. (2016) is tested. Lastly, the auto-duplication in Covidence is assessed. Total number of duplicates found, false positives, and false negatives are recorded for each method and calculated for its accuracy.

**Results**: Preliminary assessments are favorable to Bramer et al. (2016)’s systematic method of duplicate record identification in EndNote. The auto-duplication features in both EndNote and Covidence are limited in its success to correctly identify all duplicate records.

**Conclusions**: While Covidence has the potential to greatly facilitate the screening and review of citations for systematic reviews, it lacks rigor and advanced de-duplication features. A citation management program, such as EndNote, may better facilitate in identifying duplicates following Bramer et al. (2016)’s method, paired with manual screening. Both commercial programs have strengths and limitations in successfully identifying all duplicates. They can be used in combination to support different stages of the systematic review process.

Wednesday, August 12, 11: 30 a.m.–3: 00 p.m.

# Mixed Domains Session 1

Moderator: James Dale Prince, AHIP

## Best Practices in Teaching Evidence-Based Medicine: An Observational Study of Entrustable Professional Activities (EPA) 7 in Clinical Clerkships

**Catherine Pepper,** Associate Professor/Coordinator of Library Field Services, Texas A&M University, Austin, Texas

**Andrew S. Hamilton,** Assistant Professor, Oregon Health & Science University, Portland, Oregon

**Kelly Thormodson,** Associate Dean and Director, Harrell Health Sciences Library Penn State University, Hershey, Pennsylvania

**Kristine M. Alpi, AHIP,** University Librarian, Oregon Health & Science University, Portland, Oregon

**Esther E. Carrigan, AHIP,** Professor, Texas A & M University Medical Sciences Library, College Station, Texas

**Objectives**: This qualitative study investigated how—and whether—EBM instruction, as described by AAMC’s EPA7(1), is incorporated into clinical clerkships. Objectives include: (1) Describe methods of teaching and assessing EBM competencies in clinical rotations; (2) Describe extent of librarians’ involvement with teaching EBM in clerkships; (3) Analyze gaps between existing and expected states in key elements of teaching EBM in clinical clerkships.

**Methods**: The investigator gained permission to follow clerkship faculty, residents, and students on clinical patient rounds and in didactic trainings on several services at a large public academic medical institution in Fall 2019 to observe instances of EBM teaching and practice. The investigator recorded demonstrations of EBM instruction, practice, and competency assessment as they occurred on a field guide, which was comprised of functions, competencies, and behaviors as described in EPA 7(1). Semi-structured interviews were conducted with medical librarians and clinical faculty. Questions covered frequency and depth of student exposure to EBM competencies as applied to patient cases, assessment methods, use of UpToDateTM as an equivalent to EBM, and extent of librarian involvement. Aggregated observations of functions, competencies, and behaviors were presented by status, and exemplars identified to highlight how observations corresponded to or conflicted with the interview data.

**Results**: Observations of clinical teaching were recorded in various activities in several clerkships. Data collection will conclude in November 2019. Final data analysis will be completed by February 2020 and results will be reported at the meeting.

**Conclusions**: Conclusions will be presented at the meeting.

## Building on Foundations: A Collaborative Approach to Teaching Evidence-Based Medicine to Veterinary Students

**Kim Mears,** Health Sciences & Scholarly Communications Librarian, University of Prince Edward Island, Charlottetown, Prince Edward Island, Canada

**Jason Stull,** Assistant Professor, Atlantic Veterinary College, Stratford, Prince Edward Island, Canada

**Background**: In a survey of twenty-two AVMA-accredited colleges of veterinary medicine in the US and Canada, authors Shurtz, Fajt, Heyns, Norton & Weingart (2017) found that librarians are an underutilized resource in teaching evidence-based veterinary medicine (EBVM). This program details the collaboration between a veterinary medicine instructor and a librarian to teach a core, one-credit EBVM course to second-year veterinary students.

**Description**: Students attend face-to-face instruction sessions that include lectures and tutorials. Lectures highlight key components of the EBVM process, while tutorials allow for in-depth activities and discussions.The course structure purposely mirrors the Royal College of Veterinary Surgeons' EBVM Toolkit, which is leveraged throughout the course. The main assignment gives students practical experience in EBVM by outlining the process of answering a clinical query and it is formatted according to a journal's (Veterinary Evidence) submission format for a knowledge summary. The librarian establishes a presence by delivering a lecture and tutorial on information organization, databases for veterinary medicine, and strategies for developing searches. The librarian also walks students through an in-class activity and attends all lectures to answer questions and develop a collaborative atmosphere with veterinary instructors and students. Grading is performed jointly by the librarian and veterinary instructor.

**Conclusion**: To determine if the collaboration between a veterinary medicine instructor and a librarian to teach EBVM was successful, evaluation will be conducted using standardized student course evaluations. The survey will also be used to guide future course modifications.

## Arts, Humanities, and Social Sciences in Undergraduate Medical Education: A Scoping Review

**Misa Mi, AHIP,** Professor, Librarian, Oakland University William Beaumont School of Medicine, Rochester, Michigan

**Lin Wu, AHIP,** Assistant Director for Research & Learning Services, Health Sciences Library/University of Tennessee Health Science Center, Memphis, Tennessee

**Yingting Zhang, AHIP,** Information & Education / Research Services Librarian, Rutgers, The State University of New Jersey, New Brunswick, New Jersey

**Wendy Wu,** Information Services Librarian, Wayne State University, Detroit, Michigan

OBJECTIVES: There is a growing interest in integrating arts, humanities, and social sciences (AHSS) into medical education to develop well-rounded physicians. Medical educators are incorporating content of these disciplines into the medical curriculum to help students build empathy and teach communication and teamwork skills. This review aimed to investigate how these disciplines were integrated into medical education and what impact AHSS interventions had on medical students.

METHODS: We used the PRISMA extension for scoping reviews (PRISMA-ScR) to guide the review process. We conducted comprehensive literature searches in August 2019, using online databases including PubMed, EMBASE, CINAHL, Cochrane Library, and several other resources. Search terms included free text words combined with subject headings unique to each database if applicable. Articles that were primary studies investigating AHSS interventions in undergraduate medical education and published in English over the past 10 years were included for the review. Two authors conducted screening in duplicate and independently. We created and piloted a data charting form and paired together to chart data on participants, settings, AHSS interventions, interventions by year, outcome measures, and outcomes. We used Kirkpatrick’s four level model to code learning outcomes resulted from the implementation of AHSS interventions.

RESULTS: Our literature searches yielded a total citation of 6394. After duplicates were removed in Covidence, 3059 citations remained for title/abstract screening. The title and abstract screening led to 52 candidate articles for full-text screening; and 29 articles were selected for data charting. AHSS interventions were integrated into both preclinical and clinical education and delivered in various settings such as classroom, hospital, art museum, assisted-living facility, or retirement home. Duration of intervention ranged from one or few course contact hours to a longitudinal educational experience. Artworks was mostly used in interventions. Questionnaire or survey was the most popular assessment method used in 16 studies followed by Jefferson's Scale for Empathy (student version) used in 6 studies. Compassion or empathy was the primary outcomes assessed in 17 studies.

CONCLUSIONS: The review has provided us with insight into what and how aspects or content of AHSS were incorporated into medical education and how AHSS interventions affected students in medical education. The line of research in the area and a growing interest in AHSS in medical education point to new, abundant possibilities and opportunities for health sciences librarians to make impactful contributions to educating health profession students who will be entrusted to care for us in the future.

## Defining Graduate Medical Education (GME) Librarianship: Creating and Developing a New GME Library Program

**Laura A. Murray,** HCA Graduate Medical Education Librarian, University of South Florida Morsani College of Medicine, Tampa, Florida

**Candice Kunkle,** Graduate Medical Education Project Manager, University of South Florida Morsani College of Medicine, Florida

**Background**: An academic research institution and a corporate hospital system formed a new graduate medical education (GME) consortium. The consortium objectives were to increase the scholarly activity of the residents and fellows in a national hospital system's GME residency program to match the requirements set forth by the ACGME. A GME librarian position was created specifically to serve the GME research programs at Florida area hospitals to help with this objective. The GME Librarian experience is unique and other GME Librarians will benefit from hearing about the activities and lessons learned from the GME Librarian in this position.

**Description**: This paper describes the experience, activities, and lessons learned from the creation of a new GME Library program. It focuses on entering the nine hospitals within the hospital network area, developing training and supports to establish partnerships with the hospitals' program directors and coordinators. It addresses the importance of Meet & Greets, conducting a needs assessment, creating curricula utilizing both academic and corporate content, drawing upon the expertise within the academic home, and addressing pertinent ACGME requirements. It concludes with lessons learned and next steps.

**Findings**: Meet & Greets allowed a low-key way to introduce the GME librarian and university, explain the role in the GME program consortium; introduced initial tools to help RFFs with scholarly work, and established trust and familiarity with the RFFs and hospital GME staff (e.g. program coordinators). A Needs Assessment survey helped us develop a tailored curriculum for each specialty and/ or hospital environment, discover what is known and not known among the RFFs, and set a baseline for future use to determine the success of the library programming. Creating curricula utilizing both academic and corporate content meant maintaining academic integrity and unifying the culture and vision of both institutions to improve patient outcomes and achieve worthwhile academic and scholarly activity required by ACGME. Developing this dynamic and evolving

curriculum requires a continuous improvement process based on feedback from the RFFs, the hospitals, and both institutions.

**Conclusion**: The GME Librarian experience is unique and other GME Librarians will benefit from hearing about the activities. Lessons learned from the GME Librarian in a new academic-corporate consortium: Establishing relationships with all stakeholders early and regularly is critical. It is critical to make friends with the program coordinators. It is important to make yourself invaluable. Invite yourself don’t wait to be invited. An elevator speech goes a long way.

Wednesday, August 12, 11: 30 a.m.–3: 00 p.m.

## Professionalism & Leadership Session 2

Moderator: Joe Swanson, Jr., AHIP

### Developing a Culture of Inclusivity through the Formation of a Library Diversity and Inclusion Team

**Jane Morgan-Daniel, AHIP,** Community Engagement and Health Literacy Liaison Librarian, University of Florida, Gainesville, Florida

**Lauren E. Adkins,** Assistant University Librarian, University of Florida, Gainesville, Florida

**Mary Edwards, AHIP,** Liaison Librarian, University of Florida, Gainesville, Florida

**Chloe Hough,** Evening Circulation Supervisor, University of Florida Health Science Center Library, Gainesville, Florida

**Melissa L. Rethlefsen, AHIP,** Associate Dean and Fackler Director, University of Florida, Gainesville, Florida

**Michele R. Tennant, AHIP, FMLA,** University Librarian, Health Science Center Library, University of Florida, Gainesville, Florida

**Background**: In October 2018, a diversity, equity and inclusion team formed at an academic health science library. The team’s charge is to develop and promote diversity, equity, and inclusion initiatives within the library and beyond to enhance the library’s locus as a safe and welcoming space for all patrons. This presentation focuses on the team’s launch and activities within its first two years, to provide guidance to other libraries thinking of establishing similar committees. Specifically, it discusses the rationale behind the team’s formation and structure, its goal-setting process, the development of campus-based partnerships, project-related successes and challenges, and future plans.

**Description**: The team is comprised of a diverse group of 13 library staff and faculty members. Goal-setting began with the conversation prompt “what does DEI mean to you” and the resulting goals align the team’s scope with broader campus DEI efforts:

1. Assessing and identifying library-based DEI opportunities

2. Applying assessment findings to develop programming (internal and external), spaces, and activities

3. Actively promoting DEI in the library and across campus through collaborations and trainings

With these goals in mind, over the past two years the team has conducted a survey exploring DEI issues faced by patrons; organized trainings about implicit bias, recognizing privilege, nonviolent communication, LGBTQIA+ allyship, and bystander interventions; created a LibGuide on DEI health-related resources; installed a Lactation Pod; advocated for all-gender restrooms; and hosted a well-attended panel discussion on the experiences of healthcare professionals identifying as LGBTQIA+.

**Conclusion**: Although the team has experienced a number of successes, including having recently received the University Libraries’ Team-Award for Diversity, Equity, and Inclusion, ongoing challenges include fostering partnerships with non-library departments to effectively promote DEI

outside of library spaces, resistance to change due to existing organizational culture, and locating funding for events and trainings.

Future plans involve a local environmental scan that will initially examine the inclusiveness of the language on the library’s website and a digital Race Card Wall to facilitate dialogue on experiences of race and identity.

## Vision for the Future: A Hospital Library Partnership to Develop Diversity and Inclusion Programming

**Liz Kellermeyer,** Biomedical Research Librarian, National Jewish Health, Denver, Colorado

**Background**: The purpose of this project was to build a partnership between a hospital library and a larger health institution to enhance diversity-related educational opportunities and resources for their communities. A hospital librarian received a grant that supported two related projects: 1) funding for two speakers to come to campus to provide educational sessions on diversity, inclusion, and equity topics, and 2) launching a dedicated Diversity & Inclusion (D&I) print collection managed by the library.

**Description**: As a member of the hospital’s D&I Council, the librarian worked together with council members and library staff to meet the goals of the grant. Funds were used to co-sponsor a talk on health disparities in cystic fibrosis, and to bring an immersive workshop to campus focusing on cultural humility. Additionally, a D&I print collection was launched, with 20 seed titles. This project fostered collaboration between the library and key stakeholders, demonstrating the library as a valuable partner in the institutional effort towards promoting diversity and inclusion education.

**Conclusion**: Evaluative surveys were built in REDCap and distributed electronically to all attendees. The responses were very positive and showed a high instance of first-time attendees to a D&I sponsored program. Questions related to the speakers’ learning objectives were formulated and attendees were asked to rate their awareness or abilities in these areas before and after the programs; marked increases were seen across the board. A majority of participants stated they were likely to apply something they learned from the programs to their daily lives, and that they would recommend the program. The print collection circulation has been low but steady.

## Using a Critical Librarianship Framework with Medical Library Institutional Repositories: Tactics and Outreach

**Daina Dickman, AHIP,** Scholarly Communication Librarian, Sacramento State University, Sacramento, California

**Background**: Medical libraries are engaging more with institutional repositories IRs and with critical librarianship. Libraries interested in using a critical librarianship framework for their IRs have the opportunity to apply this to their strategic plans. Critical librarianship is the belief that, in our work as librarians, we should examine and fight attempts at social oppression. This concept also applies to IRs. If the IR as a neutral space is unachievable and an actively harmful idea, it is important to strategize how to address that in our repository. Targeted outreach efforts can help to ensure a wider representation of items collected.

**Description**: Outreach tactics applying critical librarianship in an IR can be conceptualized from the beginning or incorporated later in a project. Although more complicated than relying solely on PubMed searches and self-submitted items, these efforts are necessary to ensure the IR is gathering and promoting diversity, equity, and inclusion of authors and subject matter. Targeted outreach

efforts undertaken to promote equity and representation will be discussed. Outreach at an individual author level, organizational departments, and employee affinity groups will be discussed. While PubMed searches capture many articles for inclusion, outreach and

awareness is the main tactic to collect other literature. Strategies for increasing IR representation and diversity of authors, employment categories, locations, and subject matter in a large, complex institution will be explored. It is the goal that a critical librarianship informed IR can be a tool in addressing medical disparities.

**Conclusion**: Critical librarianship informed targeted outreach efforts as part of the launch and/or strategic planning for an IR creates the space to devote staff time and purpose for this intrinsic goal. This is an ongoing approach to medical library IR management and designed to inspire conversation. With both institutional repositories and critical librarianship as emerging and growing concepts in medical librarianship these discussions will be useful and instructive to institutional repository managers in medical and health science libraries.

## Diversity and Inclusion and Professional Development: Implementing an Annual Performance Requirement

**Melissa De Santis, AHIP,** Director, Strauss Health Sciences Library, Aurora, Colorado

**Nina L. McHale, AHIP,** Head, Education & Reference, University of Colorado Strauss Health Sciences Library, Aurora, Colorado

**Lisa K. Traditi, AHIP,** Deputy Director, Health Sciences Library/University of Colorado Anschutz Medical Campus, Aurora, Colorado

**Background**: The leadership team at the Strauss Library (University of Colorado Anschutz Medical Campus) wanted to be intentional in their support of diversity, equity and inclusion. The library has a commitment to diversity in its strategic plan that is in line with the parent institution’s strategic plan. Staff consistently appreciate D&I opportunities that have been brought to the library but they also say it is challenging to make time for D&I with the demands of daily work. After hearing a presentation from the library at the Medical University of South Carolina about a staff requirement for professional development related to diversity, the Strauss Library decided to implement a similar requirement.

**Description**: In January 2019, the library implemented a requirement that all library staff complete a minimum of four hours of professional development on a topic related to diversity and inclusion (D&I) during the performance plan year. The goal of the requirement is to provide a relatively easy method for library staff to explore D&I topics of interest to them as well as provide permission to participate in professional development during the work day. Professional development could be in any format (in person, online, synchronous, asynchronous, etc.) and needed to cover at least one of the 15 protected characteristics identified by the parent institution to count towards the requirement.

**Conclusion**: The program was evaluated midyear (July 2019) with an anonymous survey to all staff. Additionally, library leadership shared experiences and feedback during regular meetings throughout the year. Minor adjustments were made to the requirement based on feedback in July 2019. A second anonymous survey took place at the end of the year (Dec 2019). Based on survey results and feedback from department heads, a decision was made to continue the requirement.

## An Analysis of the Training Needs of the Profession Regarding Accessibility and Disability

**JJ Pionke,** Applied Health Sciences Librarian, University of Illinois at Urbana-Champaign, Champaign, Illinois

**Objectives**: This research aimed to discover what library graduate students are learning about disability and accessibility with one survey. Another survey asked very similar questions to current library employees to determine what the actual educational needs are of the library workforce. While both surveys have been examined individually, the goal is to analyze the continuum of data to ascertain trends.

**Methods**: Two national surveys were distributed via email invitation and social media in the fall, 2018. The Qualtrics platform was used to create the surveys. The surveys were staggered and open for two weeks at a time, with reminders sent every few days. Snowball sampling was used over other methods in order to get as much response as possible across a wide range of people. The library graduate student survey had a total of 187 validated responses. The library employee survey had a total of 219 validated responses.

**Results**: Both groups have similar levels of discomfort or comfort around the same tasks. That said, library employees have more discomfort around technical skills while library graduate students are less comfortable with interacting with patrons. Both groups need more training in these areas. Both groups see accessibility and assistive technology as being relevant in five years. Finally, both groups are strongly interested in more training.

**Conclusions**: Individually, there are deficits for each population, current library employees and current library graduate students. Taken together there is a clear continuum of a need for education and comfort with working with people with disabilities and assistive technologies. There is plenty of room for further research in so far as developing educational interventions and delving deeper into attitudes towards people with disabilities and towards assistive technology.

Wednesday, August 12, 2: 00 p.m.–3: 00 p.m.

---

## PubMed Update - Wednesday's Video Chat

Marie Collins

Rebecca Brown, AHIP, Training Development Specialist, Spencer S. Eccles Health Sciences Library, Salt Lake City, Utah

Kathi Canese, PubMed, Program Manager, NCBI NLM NIH, Bethesda, Maryland

Marie Collins from the National Library of Medicine provides the latest news on PubMed for the Medical Library Association vConference 2020. Marie highlights the improved mobile experience, the “Best Match” sort, interface improvements, and where to go for support and training.

Wednesday, August 12, 2: 30 p.m.–3: 00 p.m.

---

# Lightning Talks 3

Moderator: Michael S. Fitts

## Librarians at Nurse Rounding

**Catisha Benjamin,** Manager of Library Services, Children's Hospital Colorado, Aurora, Colorado

**Marie St. Pierre, AHIP,** Medical Librarian, Childrens Hospital Colorado, Aurora, Colorado

**Background**: When the librarians approached the nurse leaders of the 6th floor of the hospital (two units) about how they could be of more direct assistance, they were invited to attend daily rounds. They attend once per week per unit, and answer any informational needs as arise. The 6th floor tends to post-surgical, general medical, and neurology monitoring patients. A charge nurse, nutritionist, child life specialist, social worker, case manager, and chaplain are present. Each nurse in turn briefly presents their patient’s status, and any concerns. The whole round takes about 40 minutes.

**Description**: The librarians sit in on the rounds and offer both on the spot answers to questions using iPad, or take some time immediately after the round to find the appropriate information (be it article, chapter, or similar). The questions can be implied when a nurse simply mentions something that she does not know much about or can be direct when they specifically ask. The answers can be a diagram about an operation, or more complex information up to a search on certain medicines and their use in certain diagnoses. Care is taken to use authoritative sources. On occasion, a question that is not health care related may come up, and that would also be answered as best as possible.

**Conclusion**: The nurses on these two units do not experience many unusual diagnoses, and are well experienced, so not as many questions were asked as might have been expected, but the questions that arose were answered in a timely manner, within an hour. The librarians have a much better understanding of the work the nursing staff does, and what their informational needs are. The surprising result was the questions asked by the other staff, such as case workers, nutritionists, and social work. Being at rounding helped all staff become more familiar with the library.

Wednesday, August 12, 3: 15 p.m.–4: 30 p.m.

# Clinical Support Immersion Session 1

## Context Is Everything: Answering the Clinical Question for Nursing Care, Culture, and Research

**Michelle R. Lieggi, AHIP,** Clinical Research Librarian, UCSF Medical Center, San Francisco, California

**Marilyn G. Teolis, AHIP,** Information Services Librarian, James A. Haley Veterans Hospital, Tampa, Florida

**Kristin M. Chapman, AHIP,** Clinical Librarian, Howard University, WASHINGTON, District of Columbia

**Helen-Ann Brown Epstein, AHIP, FMLA,** Informationist, Virtua, Mt Laurel, New Jersey

**Session Format:** The session will be a case study format, with brief (10 minute) presentations by each presenter, followed by Q&A and group discussion.

**Objective:** The objective of this session is to present different types of scenarios from nursing practice and demonstrate how librarians can provide support for these scenarios based on the steps of evidence-based practice (EBP). The session will provide an overview of the steps (based on Melnyk et al, Evidence-based practice in nursing & healthcare: a guide to best practice, 2019), illustrating their use in a general hospital setting. Each presenter will present a different scenario and facilitate the development of a clinical question, using the PICOT model (Patient population/disease, Intervention or Issue of interest, Comparison intervention or Issue of interest, Outcome, Time). Each presenter will then outline an approach to addressing the question following the steps of EBP, varying by the specific context of the question: point of care, institutional EBP culture, primary research, systematic reviews. Q&A and group discussion will follow.

**Instructional Methods:** The case study format will immerse participants in a simulated clinical environment where they will participate in developing a clinical question for different nursing practice needs. Participants will hear from expert librarians and have the opportunity to share their own experiences supporting nursing practice with their peers. This will serve to educate participants about practices they can potentially apply within their own institutions.

**Participant Engagement:** Participants will work together to develop clinical questions using PICOT for 4 different clinical scenarios. During Q&A and the group discussion, they will be encouraged to ask questions as well as discuss their own experiences supporting point of care, EBP, primary research, and/or systematic reviews.

**Sponsors:** NAHRS

Wednesday, August 12, 3: 15 p.m.–4: 30 p.m.

## Professionalism & Leadership Immersion Session 2

### Safe Zone Training for LGBTQIA+ Awareness & Inclusivity

**Hannah Schilperoort,** Information Services Librarian, University of Southern California, Los Angeles, California

**Meredith I. Solomon, AHIP,** Outreach Officer, Harvard Medical School, Boston, Massachusetts

**Mary Catherine Lockmiller, AHIP,** Health Science Librarian, Northern Arizona University / Cline Library, Tempe, Arizona

**Jane Morgan-Daniel, AHIP,** Community Engagement and Health Literacy Liaison Librarian, University of Florida, Gainesville, Florida

**Jacqueline Leskovec,** Network Librarian, NNLM GMR, Chicago, Illinois

**Brenda M. Linares, AHIP,** Health Sciences Librarian, School of Nursing, University of Kansas Medical Center, A.R. Dykes Health Sciences Library, Olathe, Kansas

**Brandi Tuttle, AHIP,** Research & Education Librarian, Duke University Medical Center Library & Archives, Durham, North Carolina

**April Wright,** All of Us Community Engagement Coordinator, NNLM Southeastern/Atlantic Region, Baltimore, Maryland

**Emily Vardell,** Assistant Professor, Emporia State University School of Library and Information Management, Olathe, Kansas

**Description for program:** Join your colleagues to discuss LGBTQIA+ inclusive language and practices in a safe learning space online. Together, we will explore the basics of vocabulary, pronoun usage, gender and sexual identities, intersectionality, and privilege. Participants will engage in reflection, discussion, and scenario-based learning in an intensive, fast-paced immersion session, facilitated by librarians knowledgeable in Safe Zone training. Due to scaffolded content and collaborative activities, attendance will be limited to the first 100 participants; attendees are requested to be on time. Please review the session slides and handout as well as the Statement of Appropriate Conduct at MLA Meetings before the session.

**Session Format:** Immersion session with active learning exercises and discussion.

**Objective:**

Participants will leave the session with a better understanding of LGBTQIA+ inclusive language and practices, including vocabulary, pronoun usage, gender and sexual identities, intersectionality, and privilege. This immersion session is meant to be a springboard for continued learning. Materials for continued learning will be provided.

**Instructional Methods:** Facilitators will guide participants through a series of learning activities designed to achieve session objectives.

**Participant Engagement:** Participation will include individual reflection, sharing, scenarios, and worksheets to be done in small and large groups.

**Sponsors:** LGBTQIA+ Caucus

African American Medical Library Alliance Caucus

Social Justice Caucus

Leadership and Management Caucus

Thursday, August 13, 10: 15 a.m.–11: 30 a.m.

# Global Health & Health Equity Immersion Session 1

## Improving Ourselves and Improving Care: A Hands-On Workshop to Address Unconscious Bias in Health Sciences Literature and Health Sciences Library Systems

**Rachel Keiko Stark, AHIP,** Health Sciences Librarian, California State University, Sacramento, Sacramento, California

**Mary-Kate Finnegan, AHIP,** Physical Sciences Librarian, Sacramento State University, Sacramento, California

**Molly Higgins,** Reference and Digital Services Librarian, Library of Congress, Washington DC, District of Columbia

**Session Format:** A workshop that combines lecture with active learning activities.

**Objective:** This session will define implicit bias and identify bias with a focus on racial disparities in scholarly health sciences research, help attendees identify implicit bias in health sciences libraries systems and services. The active learning activities will help attendees learn how to identify resources to craft research questions and search queries that address implicit bias in Health Sciences literature and research. The workshop will provide a foundation for information professionals to better serve and address the needs of the diverse communities that their institutions serve.

**Instructional Methods:** We will have a short list of suggested readings for participants to complete before the sessions, as well as an online, free to access, implicit bias test to complete before the session. During the workshop, participants will break for a number of active learning activities with pairs, small group, and large groups. Along with personal growth focused activities, participants will be invited to engage with the presenters and each other in information skill building experiences.

**Participant Engagement:** There will be an introduction of the terms and agreement on definitions in the workshop, a small group discussion followed by a large group discussion on the Harvard Implicit Association Test will provide attendees with background and opportunities to engage with their table and the larger group. To target different learning styles and different skill levels, our first activity involves writing experiences on sticky notes and later activities are group based learning exercises for skill improvement using various tools and databases.

**Sponsors:** Social Justice Section

African American Medical Librarians Alliance

Thursday, August 13, 10: 15 a.m.–11: 30 a.m.

# Information Services Immersion Session 3

## Focusing on Health Sciences and Public Library Collaborations for *All of Us*

**Darlene Kaskie,** NNLM All of Us Community Engagement Coordinator, NNLM Greater Midwest Region, Iowa City, Iowa

**Debra Werner,** Director of Library Research in Medical Education, John Crerar Library, Chicago, Illinois

**Lynda J. Hartel, AHIP,** Director, The Ohio State University, Columbus, Ohio

**Rachel Tims,** All of Us Community Engagement Coordinator, University of North Texas Health Science Center, Fort Worth, Texas

**Dana Wilkosz,** Health Literacy Educator, New Orleans Public Library, New Orleans, Louisiana

**Mary Houser**

**Session Format:** Panel discussion

**Objective:** Recognizing the important role that public libraries play in the health and wellness of their communities, NNLM funding has enabled academic health sciences and public librarians across the country to make precision medicine and health literacy primary components of their institutional programming. Four librarians will share stories of collaboration with multiple community stakeholders to lead their communities to quality health information while raising awareness of All of Us. From their successes and challenges, discover tips, techniques, and resources to develop similar community engagement strategies for your health outreach endeavors.

**Instructional Methods:** With a focus on improving health literacy around the country for populations Underrepresented in Biomedical Research (UBR), as well as raising awareness of the NIH All of Us Research Program, four librarians will share examples of their collaboration efforts. Handout materials and a video will introduce participants to the All of Us Research Program, which was the funding source for the initiative.

**Participant Engagement:** Minimum of 15 minutes for Q&A with panelists

**Sponsors:** National Network of Libraries of Medicine (NNLM); University of Iowa, Hardin Library for the Health Sciences; University of North Texas Health Science Center; University of Chicago, John Crerar Health Science Library; The Ohio State University Health Science Library

Thursday, August 13, 10: 15 a.m.–11: 30 a.m.

# Information Services Immersion Session 1

## Seeing Things Differently: Evolution, Assessment, and Future Roles of Liaison Librarians

**Emily J. Hurst,** Interim Director and Associate Dean, Tompkins-McCaw Library for the Health Sciences, VCU Libraries, Richmond, Virginia

**Natalie Clairoux,** Dentistry Librarian, Universite de Montreal, Beaconsfield, Quebec, Canada

**Hannah Friggle Norton, AHIP,** Chair, Health Science Center Library - Gainesville, University of Florida, Gainesville, Florida

**Michelle Cawley,** Head of Clinical, Academic, and Research Engagement, UNC Chapel Hill, Chapel Hill, North Carolina

**John W. Cyrus,** Research and Education Librarian, VCU Libraries, Richmond, Virginia

**Session Format:** Lighting talks by three librarians interspersed with facilitated small group breakout activities and discussion.

**Objective:** This session aims to further the conversation about the evolving nature of liaison roles and duties. Through activities, attendees will explore differences in liaison roles and examine the evolution of the liaison librarian in health sciences libraries. Facilitated activities provide an opportunity to brainstorm and discuss what’s next for the role of the liaison librarian. Attendees will share information about ways to assess liaison duties and gain insights that further develop their familiarity with functional support models and other methods to support library users.

**Instructional Methods:** Lightning talks will present background information on three topics relevant to liaison models. The attendees will further explore these topics through inquiry-based learning activities. Using a participant-centered teaching approach, attendees will work collaboratively with information related to the lightning talk topic, summarize and categorize this information, arrive at questions based on their own experience that further elucidate the content, and then synthesize group findings by sharing back finding with all participants. Facilitators will float among the tables to provide guidance on all activities.

**Participant Engagement:** Participants will be asked to respond to lightning talks through a combination of facilitated small group activities and discussion based on Liberating Structures activity plans. During the activities and discussion, participants will record notes and takeaways into a shared online document that will combine across all groups and will be shared with participants and broader MLA membership after the conference as a follow-up.

**Sponsors:** Veterinary Medical and Animal Health Librarians

Libraries in Health Sciences Curriculum

Thursday, August 13, 11: 30 a.m.–3: 00 p.m.

# Education Session 6

Moderator: Kimberrly Loper

### Conducting a Long-Term Evaluation of Data Workshops: Evaluating the Impact of Three Years of Classes

**Fred Willie Zametkin LaPolla,** Research and Data Librarian, Lead Data Education, NYU Health Sciences Library, New York, New York

**Nicole Contaxis,** Lead, Data Discovery, NYU Health Sciences Library, New York, New York

**Alisa Surkis,** Assistant Director, Research Data and Metrics/Vice Chair for Research, NYU Health Sciences Library, New York, New York

**Objectives**: Educational programming is a common way for libraries to provide service in data management, visualization and reproducibility. The impact of this programming is difficult to assess and library literature provides limited information on evaluation efforts. While in-class evaluations were overwhelmingly positive, we sought explore if the data services workshops had an impact on participants’ work after they left the workshop.

**Methods**: A mixed methods framework was employed to assess participant attitudes about the workshops. We administered a survey combining multiple-choice and free text fields to ask participants if attending a library workshop has had an impact on their work life, broadly defined, and if so to write in responses. This survey was sent to all past participants in our library's data workshops. We then invited willing participants to a semi-structured in-person interview, continuing interviews until thematic saturation was achieved. We coded interviewee responses for themes from a phenomenological approach, with the goal of understanding the participants' experience as they see it with library-provided education.

**Results**: While data collection is still on-going, preliminary results indicate that majorities of class participants continue to use what they learned, with 50% using materials either monthly or weekly, and over a third using what they learned a few times a year. Sixty-four percent of participants indicated that the materials they learned in a library workshop, while 24% stated the classes helped them approach challenges in a new way and 47% indicated that they think about issues differently. The process of beginning a long-term evaluation has also elucidated challenges that can help for better research in the future to provide more actionable information on our offerings.

**Conclusions**: While qualitative analysis of interviews is still on-going, our preliminary research indicates that data education workshops appear to have an impact on many of those who attend them's work lives. Additionally, conducting evaluative research has highlighted that evaluation is an ongoing process, with room for iterative improvement.

### Integrating Evidence-Based Medicine Skills into a Medical School Curriculum: A Quantitative Outcomes Assessment

**Laura Menard,** Assistant Director for Medical Education and Access Services, Indiana University, Indianapolis, Indiana

**Amy Blevins,** Associate Director for Public Services, Indiana University School of Medicine, Indianapolis, Indiana

**Objectives**: Library faculty recently participated in the redesign of evidence-based medicine (EBM) content in the medical school curriculum. This project aims to determine whether a librarian-taught approach to EBM instruction featuring a scaffolded, progressive curricular structure results in better grades on an EBM assignment administered during the 3rd year Internal Medicine clerkship compared to an isolated EBM course taught by clinicians.

**Methods**: In order to assess and compare student learning under the legacy, mixed, and updated curricula (see below for details), the PI and a team of faculty members are using a modified Fresno rubric to grade three years’ worth of clerkship-level EBM assignments given to all students after receiving the three different curricular approaches to EBM instruction (n = roughly 300). Once grading is complete, an independent t-test will be used to analyze results.

Year 1 (Legacy) - 2 month isolated course in year 2 & pre-clerkship session taught mostly by clinicians

Year 2 (Mixed) - 2 month isolated course in year 2 plus 3 scaffolded sessions in years 2 & 3 taught by a mix of clinicians & librarians

Year 3 (Updated) - 7 scaffolded sessions in years 1, 2, & 3, taught mostly by librarians

**Results**: This study is ongoing; however, we anticipate being able to present results by May of 2020.

**Conclusions**: This study is not yet complete. However, If this research shows that students perform better on an EBM assignment administered during the first year of clerkship when they have received scaffolded, progressive instruction delivered by library faculty, the results could give health sciences librarians the evidence they need to make a stronger case for more involvement in the curriculum.

## Wicked Workshops: Pulling Back the Curtain on Systematic Review Search Strategy Creation

**Kaitlin Fuller,** Liaison & Education Librarian, University of Toronto, Scarborough, Ontario, Canada

**Erica Hazel Nekolaichuk,** Faculty Liaison & Instruction Librarian, University of Toronto, Toronto, Ontario, Canada

**Background**: The number of knowledge syntheses (KS) published has steadily increased; this trend is reflected at our academic library where we have experienced a dramatic rise in KS consultations.

To meet this need, we developed a workshop series grounded in our belief that graduate students can not only learn KS search skills but that it’s crucial for them to understand how the search affects overall review quality. When designing our instruction, we strove to demystify the KS search process for our students, reflecting deeply on our own habits and being radically transparent about why do things the way we do them.

**Description**: Since March 2017, our 3-part series has been offered 9 times to over 400 participants. It is designed for graduate students in the health sciences and consists of 3 2.5 hour sessions. During these interactive workshops, students practice an objective, structured method for developing exhaustive search strategies; identify potential sources for bias in their search and develop strategies to mitigate them; and prepare search strategies to ensure proper reporting.

Our teaching is rooted in principles of instructional design and we use an iterative process of content creation. Each session utilizes a combination of lecture slides, individual activities, online polls, and

group activities. We believe in authentic and intentional engagement, a focus on processes not tools, and incorporating active learning.

**Conclusion**: Student reflections and follow-up consultations indicate that participants are motivated to improve their searching. Importantly, they show concern for research waste generated by poor reviews, thinking critically about not only improvements they can make in their own reviews, but the difference they can make to the KS landscape overall. Following the workshop, many students are excited to share their new skills with their team, while others expand their team to recruit librarian expertise, or pivot the project entirely for more feasible deliverables. We are expanding our programming to include workshops for supervisors and non-medical librarians working with students conducting KS.

Thursday, August 13, 11: 30 a.m.–3: 00 p.m.

# Information Services Session 3

Moderator: Stefanie Lapka

## Technology-Assisted Systematic Reviewing: Collaboration and Experiences of Health Sciences Librarians from Multi-Institutions

**Misa Mi, AHIP,** Professor, Librarian, Oakland University William Beaumont School of Medicine, Rochester, Michigan

**Yingting Zhang, AHIP,** Information & Education / Research Services Librarian, Rutgers, The State University of New Jersey, New Brunswick, New Jersey

**Lin Wu, AHIP,** Assistant Director for Research & Learning Services, Health Sciences Library/University of Tennessee Health Science Center, Memphis, Tennessee

**Wendy Wu,** Information Services Librarian, Wayne State University, Detroit, Michigan

BACKGROUND: For the past decade, a number of web-based or computer software programs or products have been used to aid the process of collaboration in conducting systematic reviews. The anatomy of these products would be useful for health sciences librarians when making informed decisions on product selection in conducting systematic reviews or in budget planning by administration. Health sciences librarians play a key role in systematic reviews and other types of research syntheses. We, a team of health sciences librarians from four different libraries, have been actively collaborating and leading efforts in conducting systematic reviews at our own home institution.

DESCRIPTION: Health sciences librarians have been integral to the process of systematic reviews and other types of research syntheses. The rigorous process could be labor intensive, involving efforts by members with different expertise and skills. In this presentation, we are going to describe and demonstrate how we collaborated as a team across different settings and what technological tools we utilized to aid the systematic review process. We will examine unique features, functions, and limitations of each product, and share lessons learned and some recommendations regarding approaches to collaboration in conducting systematic reviews in the technology-assisted environment. For our collaboration, we used a team- and project-based approach to identifying a common topic of interest and conducting a systematic review enabled by technologies while sharing our expertise, skills, and learning with each other.

CONCLUSIONS: The multi-institutional collaborative project was not without any challenges. Yet, the collective learning and execution of the project have benefited us in many ways. We had a better understanding of each product’s strengths and limitations; the project enhanced our abilities in conducting systematic review and supporting and collaborating with our library clients. Our collaboration with the technology-assisted systematic review is a great way for us to engage in lifelong learning, continuous professional development, and stay connected even though we are separated by distance and isolated due to the shelter-in-place or social distance order during the unprecedented times.

## An Analysis of Local Systematic Reviews: A Mixed Methods Study

**Emilie Ludeman,** Research, Education and Outreach Librarian, University of Maryland, Baltimore, Baltimore, Maryland

**Katherine Downton, AHIP,** Head of Research, Education and Outreach Services, University of Maryland, Baltimore, Baltimore, Maryland

**Yunting Fu,** Research, Education and Outreach Librarian, Research, Education and Outreach Services

**Andrea Goldstein Shipper,** Research, Education, and Outreach Librarian, University of Maryland, Baltimore, Baltimore, Maryland

**Objectives**: This study updates a 2015 project that collected baseline data measuring a university’s systematic review output and librarian contributions to published reviews. By comparing 2019 data to the initial results, librarians will assess changes in the quality of the university’s systematic reviews by measuring adherence to reporting guidelines and authors’ knowledge of best practices.

**Methods**: A team of librarians at an academic health sciences library serving schools of dentistry, medicine, nursing, pharmacy and social work analyzed systematic reviews (SRs) authored by faculty and researchers at the university. In February 2019, librarians searched the Scopus database to identify systematic reviews published since 2015. The team identified 580 potentially relevant papers, of which 112 were identified as true systematic reviews and met all predetermined eligibility criteria (adapted from Rethlefsen et al. 2015). Reviews were then screened to determine the extent of librarian involvement, as well as adherence to PRISMA guidelines for reporting search methods. Data were compared to the 119 reviews identified during the 2015 project. In October 2019, librarians surveyed corresponding authors of the identified systematic reviews to determine their knowledge of SR guidelines, methods for identifying studies for inclusion, and the extent of librarian involvement.

**Results**: Preliminary analysis of Scopus data shows evidence of increased adherence to PRISMA guidelines and a significant increase in librarian authorship on systematic reviews. Additional analysis is in progress and results of the survey are forthcoming.

**Conclusions**: More authors are adhering to PRISMA standards. We note an increase in librarian involvement, including authorship, in systematic reviews, though librarian authorship remains low.

## ORCID for Researchers: Librarians’ Role in Implementing and Supporting ORCID

**Yingting Zhang, AHIP,** Information & Education / Research Services Librarian, Rutgers, The State University of New Jersey, New Brunswick, New Jersey

**Background**: ORCID provides unique identifies for researchers worldwide. It distinguishes researchers from others with the same or similar names. ORCID iDs are often required by funders, publishers, and research organizations. In October 2017, Rutgers University officially launched ORCID @ Rutgers. This presentation is to share the experience and role of librarians in implementing and supporting ORCID for researchers.

**Description**: Rutgers implemented ORCID in October 2017 under the charge of the university-wide ORCID Implementation Working Group chaired by the VP/UL. To support researchers to use ORCID, the Outreach Subgroup including Open Access Specialist and Research Services Librarian created an ORCID website, developed brochures and handouts, wrote tutorials, etc. In April 2019, a faculty survey was conducted to evaluate the success of this initiative. Among 730 respondents, 57.45% researchers indicated they had created ORCID iDs, 25.21% did not, 17.40% were not sure what

ORCID was. On the question if they have connected their ORCID iD to their NetID, 46% of 397 respondents have done so, but 54% not yet. Additional comments showed that some researchers were not convinced why they should want an ORCID iD and have it connected to NetID. Some others indicated that they would need help.

**Conclusion**: Most researchers have created their ORCID iDs, but many of them have not connected their ORCID iDs to NetIDs due to various reasons. Even though the outreach subgroup has done much work to advocate for ORCID, more work needs to be done to reach out more researchers for their awareness and adoption of ORCID.

Thursday, August 13, 11: 30 a.m.–3: 00 p.m.

## Innovation & Research Practice Session 3

Moderator: Tara Douglas-Williams

### Setting Your Sights on an Interprofessional Library Team? Make Way for a Health Library Informaticist!

**Nicole Capdarest-Arest, AHIP,** Head, Blaisdell Medical Library, University of California, Davis, Sacramento, California

**Christy E. Navarro,** Health Library Informaticist, Blaisdell Medical Library, UC Davis, Sacramento

**Background**: Health sciences libraries are hubs offering centralized resources and services to expand the knowledge and efficiencies of their communities. Increasingly with the growth of big data, open data, and the electronic health record (EHR), clinical and translational researchers must be more data literate. At our biomedical campus, we noticed an increased need for data literacy training, including assistance with navigating health system components related to data across the research lifecycle. Engaging with key stakeholders, we advocated and garnered support for our desire to facilitate fulfilling this need, framing conversations around meeting significant institutional objectives, maximizing efficiencies, and achieving growth potential.

**Description**: Just as the communities in which we exist are interprofessional in nature in order to achieve all needs of research, patient care, and education, our libraries also need to have personnel with skillsets reflective of the 21st century information environment where “information” includes not only literature, but data (including EHR data). We worked with leadership in the school of medicine and the clinical and translational science center to co-fund a new librarian position (the “informaticist”) focused on meeting the needs for data literacy training and health sciences data landscape navigation. We recruited across a variety of professions – information science, public health, informatics, and more – to achieve our goal of filling this informaticist role with a person knowledgeable in the complexities of privacy, compliance, data systems, and the research data lifecycle.

**Conclusion**: Advocating for co-sponsorship of this interprofessional role increased the library’s visibility as an important campus partner in meeting strategic goals of increasing institution-wide data literacy and preparation for conducting and navigating data-driven research, which in turn increase institutional competitive advantage and provide efficiencies in research and education. Capitalizing on interprofessional expertise of an informaticist, such as in informatics and privacy, brings more breadth and depth of partnership opportunities to the library. We already see that successes in this role solidify the library’s position as a vibrant hub for growing knowledge around compliant and evidence-based data creation, use, and dissemination.

### Best. Library. Ever.?: Identifying Library-Climate Enhancement Opportunities through a Multiphase Diversity, Equity, and Inclusion Needs Assessment Project

**Jane Morgan-Daniel, AHIP,** Community Engagement and Health Literacy Liaison Librarian, University of Florida, Gainesville, Florida

**Lauren E. Adkins,** Assistant University Librarian, University of Florida, Gainesville, Florida

**Matthew Daley,** Web Designer, University of Florida

**Mary Edwards, AHIP,** Liaison Librarian, University of Florida, Gainesville, Florida

**Hannah Friggle Norton, AHIP,** Chair, Health Science Center Library - Gainesville, University of Florida, Gainesville, Florida

**Michele R. Tennant, AHIP, FMLA,** University Librarian, Health Science Center Library, University of Florida, Gainesville, Florida

**Elianne D. Rodriguez,** Undergraduate Fellow, University of Florida, Hollywood, Florida

**Objectives**: Charged with developing the local diversity, equity, and inclusion (DEI) climate, a newly established DEI team in an academic health sciences library launched a multi-phase needs assessment project to identify environmental limitations and enhancement opportunities. The project’s objectives were to 1) quantitatively assess patrons’ attitudes on the existing library climate and 2) qualitatively identify contextual factors influencing patrons’ perceptions.

**Methods**: During April-July 2019, HappyOrNot customer satisfaction terminals were situated at the library’s two main entrances on the first and second floors to collect responses to a series of DEI questions. The machines displayed one question per week, inviting patrons to indicate their satisfaction through pressing one of four buttons with a range of happy to sad faces. A comment box collected optional open-ended responses to each question. While giving a broad overview, this method did not allow for analysis of whether diverse population groups experience the library differently and excluded individuals who do not visit the physical library space. Thus, a follow-up online survey was distributed in February-April 2020 to gather more detailed feedback. The survey posed 17 questions (Likert-Scale), as well as additional open-ended and demographic questions. It was distributed via the library’s website, social media, and emails from liaison librarians.

**Results**: The HappyorNot project yielded 3445 responses and 7 comments. Over 90% of participants felt the library is welcoming, safe, and that they are treated equitably and with respect by employees, with one patron commenting “Best. library. ever… I feel at home here.” Negative responses referred to other patrons’ behavior, services for people with disabilities, and services for those whose native language is not English. There were 101 respondents to the online survey; participants’ responses were collated and cross-referenced with the demographic data provided, with the purpose of delineating the experiences of different population groups.

**Conclusions**: Overall, the assessment facilitated better understanding of the library-related DEI needs and expectations of different patron populations. In addition to opening a dialogue on inclusivity topics, the project enabled the DEI Team to identify priority issues for improvement, gave suggestions for specific changes to the library’s physical space, prompted training ideas for all library employees, and provided a baseline against which the impact of the DEI Team’s future work can be measured. Future plans include a library-based DEI environmental scan to identify current resources and resource gaps, which will focus on the priority issues discerned through the assessment project.

## Making Space: A Quantitative Vision for the Future of Library Space Planning

**Kristy E. Steigerwalt,** Head of Clinical Support, University of Missouri-Kansas City, Kansas City, Missouri

**Objectives**: Where do library patrons physically go when they visit the library? Do patrons in a Health Sciences library study in groups or by themselves? Do these patrons prefer tables, carrels, or partitions? How does this compare to the behavior of non-professional students?

**Methods**: A quantitative, observational, study was conducted to determine the number of students physically located in predetermined zones of the library including group, quiet, silent, computer, and study room zones. The study was conducted at an academic health sciences and main campus library. Data gathered included the number of patrons in each of the pre-determined areas, whether each of the students studied alone or in groups, and which of the multiple types of furniture each of the students occupied in the silent study space. Tally counts were taken over the course of one week, on the hour, while the library was open.

**Results**: Our results demonstrated that the majority of our Health Sciences Library patrons were located in the silent study area over the course of the week evaluated. The majority of these patrons and the Main Campus patrons were found to be studying alone. Compared to our Main campus library patrons, our Health Sciences patrons studied alone 16% more and preferred the Silent study area to the moderate volume (Quiet) Area. In the Silent study area at the Health Sciences Library the furniture preference of our Health Sciences patrons was partition seating. For both campuses extended hours have resulted in a 9% increase in the number of patrons using the library.

**Conclusions**: The purpose of this study was to address which areas of the library students gravitate towards as a result of a recent increase in hours both libraries are open. A 9% increase in patron volume was observed for both libraries. The silent area was preferred by Health Sciences Library patrons. The moderate volume area was preferred by Main campus patrons. Secondary outcomes examined what furniture and study habits (i.e. studying alone at a single table vs studying in groups) patrons engaged in. Overall patrons preferred studying alone. At the Health Sciences Library patrons preferred partition seating. This data can be used to inform staffing during hours of operation, furniture selection, and space distribution.

## Evaluation of the Impact of National Library of Medicine (NLM) Associate Fellows’ Projects: 1992–2012

**Kathel Dunn,** Associate Fellowship Coordinator, National Library of Medicine, Bethesda, Maryland

**Background**: The purpose of the evaluation of the impact of National Library of Medicine (NLM) Associate Fellows’ projects is to assess what outcomes resulted from fellowship projects conducted between 1992 – 2012. An additional goal was the opportunity to explore what constitutes a “high impact” project, to inform staff, guide current Associate Fellows in their project selection process, and to highlight high impact projects in recruiting for the Associate Fellowship Program. Previous evaluations of the NLM Associate Fellowship Program to date have not included an assessment of the completed projects.

**Description**: This study is the first assessment evaluating the outcomes of the projects beyond the completion of the project itself. Outcome criteria categories were: saved staff time, saved money, created a new position, new product or service, confirmed proposed approach worked or didn’t work, established objective measures or standards, improved methodology in decision-making or ineffective/not effective/null. Projects were determined to have “high impact” if two or more reviewers agreed on the significance of the project. High impact projects were projects identified by two or more reviewers as resulting in a project that saved staff time, money, or aided in decision-making.

**Conclusion**: Eighty-four percent (142) of the projects were identified by at least one reviewer as having an outcome; twenty-eight (17%) of the projects were identified by two or more reviewers as having an outcome. These twenty-eight projects were determined to be “high impact” projects. Six (21%) of the high impact projects had a publication associated with it, and six (21%) of the high

impact projects had a presentation associated with it. Combined twelve (43%) of the high impact projects had either a publication or national conference presentation associated with it.

Thursday, August 13, 11: 30 a.m.–3: 00 p.m.

# Innovation & Research Practice Session 4

## Moderator: Lindsay E. Blake, AHIP, Research Training Institute (RTI) Assessment Results Two Years After: Building a Research Support System for Health Sciences Librarian-Researchers

**Jodi L. Philbrick, AHIP,** Senior Lecturer, Department of Information Science, University of North Texas, Denton, Texas

**Lorie A. Kloda, AHIP,** Associate University Librarian, Planning & Community Relations, Concordia University, Montreal, Quebec, Canada

**Susan Lessick, AHIP, FMLA, AHIP, FMLA,** Librarian Emerita/Project Director, RTI, University of California, Irvine, Anaheim, California

**Background**: The MLA Research Training Institute (RTI) is a three-year program funded by the Institute of Museum and Library Services (IMLS) to equip practicing health sciences librarians with key research competencies related to scholarly research, inquiry, and publishing. It includes online and face-to-face content and activities, with a five-day immersive research workshop. After completing the workshop, the librarians conduct a rigorous research study during the ensuing year. Two cohorts of 20 health sciences librarians have participated in the RTI, and this paper focuses on program outcomes related to participating librarians’ research knowledge and engagement.

**Description**: The RTI participants’ confidence with respect to research and evidence-based practice skills is assessed before and after the workshop, comparing assessment results for two subsequent years. The instrument used for the pre- and post-assessment survey is based on methods of Brancolini and Kennedy (2017) and adapted for use with the RTI. The results of the pre- and post-assessment survey of research skills and confidence for both cohorts will be shared. The research progress and outputs of both cohorts will be discussed. Additionally, informal feedback and other evidence of participants’ research activities will be shared.

**Conclusion**: The MLA RTI has already had a positive impact on 40 practicing health sciences librarians’ ability to conduct research. Unexpected benefits and drawbacks of the RTI experience will be discussed. Pre- and post-test results demonstrate improvement in the participants’ research skills and confidence. Research progress and outputs of the participants show that they are contributing to the evidence base in health sciences librarianship and building the research capacity of the profession.

## Assessing the Impact of Programming Workshops on Biomedical Research Reproducibility

**Ariel Deardorff ,**Data Services Librarian, UCSF Library, San Francisco, California

**Objectives:** Given the growing need for computational reproducibility in the biomedical sciences many libraries have started teaching programming workshops. However, little is known about the extent to which researchers are able to translate their new coding skills into more reproducible workflows. The goal of this study was to assess the impact of programming workshops on the computational reproducibility of biomedical workflows.

**Methods:** This mixed-methods study consisted of semi-structured in-depth interviews with 14 University of California, San Francisco (UCSF) researchers at two points in time: before they participated in a UCSF Library-led introductory R and Python programming workshop, and three months after they completed the workshop. During the interviews the author collected qualitative data on the tools, methods, and processes researchers used in their work, and quantitative data from a questionnaire that measured evidence of computationally reproducible behaviors. The author analyzed the quantitative data to see if there was a statistically significant difference in reproducible behaviors before and after the workshop, and used a thematic analysis approach on the qualitative data to extract the common characteristics of the research workflows before and after, and explore what enabled or prevented researchers from making changes in their workflows.

**Results:** Pre and post scores on a checklist of reproducible behaviors did not change in a statistically significant manner. The qualitative interviews revealed that several participants had made small changes to their workflows including switching to open source programming languages for their data cleaning, analysis, and visualization. Overall many of the participants indicated higher levels of programming literacy, and an interest in further training. Factors that enabled change included supportive environments and an immediate research need, while barriers included collaborators that were resistant to new tools, and a lack of time.

**Conclusions:** While none of the workshop participants completely changed their workflows, many of them did incorporate new practices, tools, or methods that helped make their work more reproducible and transparent to other researchers. This indicates that programming workshops now offered by libraries and other organizations contribute to computational reproducibility training for researchers.

## Artificial Intelligence in Systematic Reviews: How Does DistillerSR Compare to Traditional Screening Methods?

**Kearin Reid, AHIP,** Medical Librarian, College of American Pathologists, Buffalo Grove, Illinois

**Carol F. Colasacco, AHIP,** Medical Librarian Specialist, College of American Pathologists, Pleasant Prairie, Wisconsin

Objectives: Systematic reviews (SRs) are time-intensive, and the use of artificial intelligence (AI) has the potential to reduce the time required for the systematic review process. Our objective is to determine if the AI function of DistillerSR is comparable to the conventional dual review by human subject matter experts during the title/abstract phase of the SR process.

Methods: This analytical comparative pilot study analyzes the AI function of DistillerSR for the title/abstract review of references during SRs. We performed a retrospective review of two SRs using our conventional method of dual review by human subject matter experts. To determine the equivalency of DistillerSR’s AI function, we created new projects using the same pool of references as the original projects and created an AI training set using the historical data from the original review. Then, we applied the DistillerSR’s AI tool to the remaining references and reviewed and compared the outcomes of the review methods to investigate equivalency. We calculated the sensitivity and specificity of the AI function and assessed the similarities and differences in the results obtained.

Results: To determine sensitivity and specificity, we compared the articles included at the title/abstract screening stage with the final pool of evidence included in the published guidelines, as that is of primary importance to our subject matter experts. The sensitivity of DistillerSR’s AI tool was 93% for Project 1, with the AI tool missing 8 samples during title/abstract review that were included

by human reviewers in the final evidence tables. AI sensitivity for Project 2 was 66%, with the AI tool missing 34 of 116 samples ultimately included in the final evidence tables. The specificity was 58% for Project 1 and 89% for Project 2.

Conclusions: Our pilot project sensitivity calculations for DistillerSR’s AI tool do not currently meet our acceptability threshold to allow the inclusion of this AI tool into our guideline development process. Further research is planned to continue our evaluation by performing a retrospective analysis in at least 4 additional guideline-informing SR projects. These projects vary in size and scope thus will provide valuable data about how the AI tool performs in different scenarios. There is potential that AI can be combined with human review to maximize the AI tool’s capabilities while incorporating the expertise of human subject matter experts. This may help us maintain the high-quality standards required for medical practice guideline development projects.

## Using Free Text Mining Software to Analyze Chat Reference Transcripts: A Pilot Study

**Amy J. Chatfield,** Information Services Librarian, University of Southern California, Los Angeles, California

**Karin Saric,** Information Services Librarian, USC, Los Angeles, California

**Hannah Schilperoort,** Information Services Librarian, University of Southern California, Los Angeles, California

**Objectives**: Several recent publications have demonstrated the value of mining chat reference transcripts using complex and costly text mining software and human-labor-intensive methods to conduct quantitative and qualitative analysis. Voyant is a free online suite of text mining tools. Can Voyant be used in place of other text mining software and methods to identify common themes discussed by users and librarians in chat services?

**Methods**: Transcripts from the chat reference service from an academic medical library covering July 2015- February 2019 were gathered, cleaned, and uploaded into Voyant. Based on prior qualitative research examples, authors developed themes expected to be found and lists of common phrases, keywords, and concepts correlating within themes. Various tools within Voyant were used to analyze data for quantitative and thematic properties. The outputs from each tool were compared and evaluated on time and effort spent to conduct the analysis; value/relevance of data for quality assurance, evaluation, and training development for reference services; and comparative value to other Voyant tools. We anticipate that different tools within Voyant will be more valuable when librarians have different kinds of questions and expected uses for chat reference transcripts, covering timeliness, audience, depth/sophistication (research question versus ready reference), focus of question, and training needs.

**Results**: Using the tools within Voyant is relatively simple, but requires data preparation and planning to be successful. The trends, terms, and collocates tools within Voyant are useful for quantitative data such as timeliness, length, and volume. The collocates, contexts, and phrases tools, along with additional analysis, are most useful for qualitative or thematic data such as differentiation between ready reference and research questions, further analysis of research needs, focus of questions, and training needs. No tools were able to successfully identify types of patrons.

**Conclusions**: Preparing data for Voyant tools and thinking about how to analyze the data is tedious, but using the tools is not. Output from Voyant tools can be used along with other strategies to evaluate the success of chat reference services, drive changes in services, and provide content for

training for reference staff. The information could also be used to support decisions relating to public services in outreach, communications, and instruction.

## Text Mining for Diverse Review Topics: A Prospective Study Comparing Search Strategies Developed with and without Text Mining Tools

**Robin Paynter,** SRC Librarian, Agency for Healthcare Research and Quality, Scientific Resource Center, Portland

**Gaelen P. Adam,** Librarian/Editor/Research Associate, Center for Evidence Synthesis in Health, Brown School of Public Health, South Dartmouth, Massachusetts

**Objectives**: To compare the costs and benefits of searches with and without text-mining tools (TMT):

1) Do TMT decrease the time spent developing strategies?

2) Do TMT identify groups of records that can be safely excluded?

3) Do TMT improve search performance (specificity, sensitivity)?

4) How does the performance of TMT for developing strategies compare for simple or complex review topics?

**Methods**: In this prospective study, we recruited nine systematic review projects, classifying their topics as simple or complex. Each project's information specialist used conventional methods to create a MEDLINE search strategy and another paired information specialist independently created a MEDLINE search strategy using text-mining tools. Text-mining searches were created using freely-available TMT to ensure replication of our methods and relevancy of our findings to all review producers. We collected search results for both MEDLINE strategies, coded and removed duplicates, and sent the citations to the review team for screening. We used the final list of included studies to calculate the sensitivity, specificity, precision, and Number Needed to Read (NNR) for both MEDLINE strategies. We tracked time spent by information specialists to conduct each task in their search development process. Simple and complex topics were analyzed separately to allow comparison.

**Results**: UP searches were more sensitive (92% (95% CI, 85%-99%) than TMT searches (84.9% (95% CI, 74.4%-95.4%). The mean number-needed-to-read was 83 (SD 34) for UP and 90 (SD 68) for TMT. Model search strategy development took UP librarians 12 hours (SD 8) and TMT librarians 5 hours (SD 2). TMTs did not improve identification of excludable records.

**Conclusions**: Across all reviews and for simple SR topics, TMT searches were generally less sensitive and reduced time spent in strategy development than UP searches. For complex SR topics, TMT searches were also less sensitive, but did identify includable studies not found by the UP search.

Thursday, August 13, 11: 30 a.m.–3: 00 p.m.

# Lightning Talks 4

## Turning the Evidence Pyramid Upside Down

**Jennifer DeBerg,** User Services Librarian, Hardin Library for the Health Sciences, Iowa City, Iowa

**Background**: Grading evidence using hierarchical systems have been around since the 1990’s. The evidence pyramid was first developed as a useful aid to identify high quality evidence. The purpose of this talk is to describe an alternative to the evidence pyramid, which is a simple to use but incomplete method for determining the best evidence for practice. Another aim of this talk is to stimulate conversation with colleagues to obtain input and ideas for further consideration.

**Description**: Following discussion with two nurse specialists in evidence-based practice from an affiliated teaching hospital, a literature search was conducted to further examine the problem. One concern is that the pyramid has been used incorrectly at times, especially for questions that are not intervention/therapy focused, which may lead a clinician to not notice or to disregard evidence that may in fact be significant. Another issue is that the pyramid does not incorporate patient (or health system) values, which is an essential element of evidence-based practice. Several other concerns with the pyramid will be detailed. An evidence funnel model will be presented as a supplement or alternative to the evidence pyramid.

**Conclusion**: Turning the evidence pyramid upside down creates the evidence funnel as an innovative solution for sifting through evidence. However, as use of the pyramid has been widely adopted in many health sciences specialties, introducing a different approach is challenging. The education and dissemination of this approach in process at this institution will be described.

## The Central Dogma of Biology and Its Place in Bioinformatic Library Support

**Stacey Elizabeth Wahl,** Research and Education Librarian for the Basic Sciences, Virginia Commonwealth University, Richmond

**Amy L. Olex,** Senior Bioinformatics Specialist, Virginia Commonwealth University, Richmond, Virginia

**Background**: As Health Sciences Libraries evolve, the support they offer scientists continues to expand, incorporating many aspects of the research life cycle. An academic health sciences library partnered with a center for translational research at a research university to offer trainings for scientists interested in bioinformatics. One such effort is a week-long intensive series utilizing free databases from the NCBI. A consistent challenge over four years of workshops has been the differences in biological content background of series participants. To address this challenge we added a basic genetics session to the start of the week-long intensive in the summer of 2019.

**Description**: In this lesson, participants were introduced to the central dogma of biology and utilized that knowledge in active learning sessions, with the goal of a shared understanding of the biological processes of transcription and translation. This understanding is essential to effectively using the NCBI gene and protein databases to interpret data and plan experiments. In addition to laying a solid content foundation, these activities set the stage for an interactive series and allowed participants to feel comfortable with the content and with interacting with each other.

**Conclusion**: Feedback for the sessions was largely positive with 86% of survey respondents indicating enjoying the genetics portion specifically. The activities utilized open access learning materials and

could be adapted for bioinformatic workshops at other health sciences libraries. Future directions for the series include creating additional active learning objects that use problem-based activities to engage the bioinformatic databases.

## A Library and Interprofessional Education (IPE) Collaboration: A Discussion on How the Library Can Play an Instrumental Role in Helping to Plan and Support IPE at Health Sciences Institutions

**Samuel Dyal,** Assistant Director of Library Services, Roseman University of Health Sciences, South Jordan, Utah

**Background**: Interprofessional education/collaboration is an area of growing emphasis at both small and large health providers who are seeking to improve the cost and healthcare effectiveness of patient care. At the presenter’s institution, key members of each of the colleges (Nursing, Pharmacy, Dental Medicine) formed a committee to develop a four-day Interprofessional Edcuation summit to explore ways in which each of the health disciplines could work together to improve patient care and collaborate as teams. The presenter volunteered with the planning committee in order to foster librarian/faculty collaborations and offer ways in which the library can help support interprofessional education.

**Description**: After defining and discussing the learning objectives of the IPE summit with the committee, the presenter identified areas in which the library could effectively support the objectives and fill information gaps. Library staff then worked in collaboration with faculty planning members, to develop new online content related to background research and literature on interprofessional collaboration as well as developed and led one of the primary in-person learning activities of the summit. Through this collaboration the presenter hoped to promote a welcoming environment for future faculty/library partnerships as well as offer library research expertise to the 200+ attendees at the summit. The experience also served as valuable feedback on the information needs of library patrons related to interprofessional collaboration. Towards the conclusion of the summit an evaluation was administered to attendees to gather feedback on the effectiveness of the event.

**Conclusion**: This presentation will briefly describe the results of the four-day IPE summit held in November 2019, as well as a discussion on lessons learned, areas of the collaboration that worked well, and points to ponder on improving future library/IPE collaborations.

## PubMed and Poetry: Where Science Meets Creativity

**Bridget Jivanelli,** Medical Librarian, Hospital for Special Surgery, New York, New York

**Joy Jacobson,** managing editor, HSS, New York, New York

**Background**: The objective of this talk is to demonstrate how collaboration between librarians and non-librarians can create unique educational programming that brings new life to PubMed instruction. The Medical Librarian and Managing Editor of HSS Journal were invited by leadership of the HSS Education Institute (EI) to collaborate on the first “Lunch & Learn” event held by the department. The audience included EI staff with minimal experience with either PubMed or poetry. Presenters were given freedom to decide the topic and format.

**Description**: The respective fields of the presenters have much in common – writing and searching require individuals to play with and manipulate words. A session titled “PubMed and Poetry – Where

Science Meets Creativity” was marketed to EI staff as “an hour of searching our thoughts and the literature.”

A session was planned that would encourage participation through reading and discussing a health care-related poem. The poem was then used as a prompt for the first search of PubMed, introducing MeSH and Automatic Term Mapping – demonstrating how words might be interpreted in an unintended way. After a brief introduction to PubMed the poem was reread. Attendees and presenters then spent 5 minutes writing based on prompts the presenters gave from the poem.

**Conclusion**: The interactive session was well-received, attended by 13 people. A group sonnet was created as attendees and presenters volunteered one line each from their writing. The poem was later emailed to all who attended. Group members said they gained a better understanding of how to perform a basic search in PubMed through the medium of poetry.

This collaboration between a librarian and a poet shed a unique light on tools that encouraged a playful, curious, and cohesive group approach to literature searching. After a successful event, the presenters have decided to bring this session to other audiences at HSS.

## Our Future Researchers: Early Intervention with Evidence-Based Information Literacy to High School Health Sciences Magnet Programs in Underserved Areas

**Mary-Kate Finnegan, AHIP,** Physical Sciences Librarian, Sacramento State University, Sacramento, California

**Background**: The dearth of black and Hispanic medical professionals is due to multifaceted issues which include a frequent lack of academic preparation for doctoral degree programs, lack of concordant mentors, and limited exposure to health careers. For decades now, the percentage of people of color graduating medical school has remained relatively stagnant and not risen to match the demographic makeup of the united states. One recommended pipeline into the health sciences is targeted support services in our communities helping young people build skill sets that advance them as students and professionals.

**Description**: Health Science Librarians can help by teaching search strategies and how to find sources of high-quality Open Access (OA) resources to students at local health sciences high school magnet programs. Magnet programs are focused on a special area of study and structured to be balanced in ethnic and racial student population makeup. Librarians can offer to visit and leverage our skill sets to help give these students a leg up. Using the first hour of a two-hour session cover the topics of using PICOT and synonyms to build a search and then how to apply it in OA databases using relevant filters. Use the second hour to break the students into groups to build a search using worksheets and activity cards. Finally have the student groups take a few minutes to demonstrate their searches to the class in assigned databases.

**Conclusion**: Armed with new search strategies and aware of what to look for when searching for evidence; some students report increased measures of self-confidence in finding high quality sources of research. Assigning different databases to each student group for the final activity creates users that report being able to serve as peer mentors in helping their classmates navigate the different resources.

## We Built It, They All Came, Now What?

**Karen Stanley Grigg,** Health Sciences Librarian for Collections and Instruction, UNC Chapel Hill Health Sciences Library, Chapel Hill, North Carolina

**Jamie L. Conklin,** Health Sciences Librarian / Liaison to the School of Nursing, University of North Carolina at Chapel Hill, Chapel Hill, North Carolina

**Rebecca Carlson, AHIP,** Liaison to the School of Pharmacy, University of North Carolina-Chapel Hill, Chapel Hill, North Carolina

**Elizabeth Moreton,** Clinical Librarian, UNC Chapel Hill, Chapel Hill, North Carolina

**Barbara Rochen Renner,** Library Services Evaluation Specialist and Liaison, Allied Health Sciences, Health Sciences Library, Chapel Hill, North Carolina

**Background**: Liaison librarians are often victims of their own successes. They strive to create embeddedness, and find there are not enough hours to accommodate all those who seek them out. Most liaisons are cognizant of the need to approach their roles strategically, but can find it difficult to carve aside the time needed to plan an approach that allows allow them to reach the largest number of their programs in the most effective way possible. This session will address how librarians at the University of XXX have strategized to create effective and streamlined liaison programs.

**Description**: XXX HSL liaison librarians have employed several approaches for greater efficiency: engagement with faculty to create more streamlined research assignments, curriculum mapping with skill scaffolding, team delivered drop-in “search clinics” for large classes, online assistance via Zoom, expanded subject guides, and team collaboration on instruction and research assistance. Librarians have also looked at tiered service models to reach the maximum number of users. When possible, class assignments are handled by targeted instruction, while masters papers, dissertations, and systematic reviews are handled with a combination of targeted instruction and consultations. Citation management and baseline reference questions are often answered by research assistants, and RAs have also been trained to offer classes in using citation management and Covidence classes. By requesting more embeddedness in classes with research assignments, librarian consultation time is available to assist those with more complex individualized research needs.

**Conclusion**: Outcomes:

Liaisons will analyze their instruction and consultation statistics after implementing these best practices to compare the approximate number of students reached to earlier years. Liaisons will also continue to monitor new opportunities to embed with groups by increasing instruction and search clinics.

## Data Availability Statements: Your “In” with Researchers

**Carrie L. Iwema, AHIP,** Coordinator of Basic Science Services, University of Pittsburgh Health Sciences Library System, Pittsburgh, Pennsylvania

**Melissa Ratajeski, AHIP,** Coordinator of Data Management Services, University of Pittsburgh, Health Sciences Library System, Pittsburgh, Pennsylvania

**Background**: Do you need a compelling reason to initiate a conversation about data with a busy researcher, but don’t know how to start? As data services librarians at a large academic health sciences library, we continually seek novel ways to make connections with biomedical researchers. An easy and straightforward approach we successfully use begins with setting up journal alerts in order to review data availability statements in articles authored by our institution’s researchers.

**Description**: Review of institutional author’s data availability statements provides sufficient discussion points for outreach efforts. However, we also found that these statements, many with a canned response of “will share upon request,” offer insight into how our community views data

sharing, thereby introducing potential areas for patron education. In addition, we unexpectedly discovered that some data availability statements contain inaccuracies, such as broken links and misinformation for data access, that require timely rectification. Whether these inaccuracies are due to publisher error or as a result of author misunderstanding, they emphasize the role librarians can have in ensuring accessibility of data from our own institutions.

**Conclusion**: This lightning talk presents the alerts we created, highlights a sampling of reviewed data availability statements and any subsequent corrections, and shares how we tactfully approach researchers when there is concern of misinformation. This simple approach not only improves upon the usability of purportedly available data but also paves the way for continued mutually beneficial interactions between biomedical researchers and librarians.

## Medical Librarians as Curricular Designers in Quality Improvement and Patient Safety: Advancement of Research and Scholarship through the ResQIPS Framework

**Debra Schneider,** Network Manager, Library Services and Continuing Medical Education, HonorHealth, Scottsdale, Arizona

**Background**: Program Objectives: Create a linkage between resident education & support on Quality Improvement(QI)/Patient Safety(PS) activities to scholarship, with the intent of increasing scholarly projects and improving patient care and patient outcomes in a hospital setting.

Increase participation and interdisciplinary collaboration on research and QI projects within HonorHealth’s GME programs, and more broadly, across multiple disciplines in the network.

**Description**: A librarian-led, interprofessional committee comprised of representatives from GME, Research, QI/PS, Nursing, Pharmacy and Library developed and implemented an innovative framework and curriculum called ResQIPS (Research, Quality Improvement and Patient Safety) in 2016, linking education and support on QI/PS activities to scholarship. Our librarians were selected by our CAO to oversee the project due to their competence in research principles, resource knowledge, and experience working across multiple disciplines. Our librarians teach part of ResQIPS and are actively involved in furthering the spirit of inquiry amongst Honorhealth clinicians. To date, 4 half-day workshops have been held with individual mentoring support offered between workshops. Participants (n=63 in 2017;n=141 in 2018; 2019 data in progress) report high levels of satisfaction with instructional approach, curriculum and delivery model, increased understanding of key research principles and better knowledge of how to access resources and support.

**Conclusion**: Key Findings: The ResQIPS program has made a significant impact on resident scholarship. Residency programs reported a 28% increase in projects due to organizational efforts including the ResQIPS series. Focus is now on faculty and IPE team.

In addition to changing the culture towards a spirit of “inquiry”, project contributions, especially those of organizational importance, have been recognized by the HonorHealth C-suite.

Next Steps:

Received 2 year grant funding to hire fulltime director and scale the educational program for the network.

Integrate feedback from participants for more hands-on, case-based learning.

Develop better measurement tools to capture and quantify program success.

## Developing a Clearer Vision: Understanding Researcher Motivations and Behaviors in Open Access Publishing

**Megan von Isenburg, AHIP,** Associate Dean, Library Services and Archives, Duke University, Durham, North Carolina

**Karen D. Barton, AHIP,** Biomedical Research Liaison Librarian, Duke University Medical Center Library & Archives, Durham, North Carolina

**Virginia M. Carden, AHIP,** Administrative Research Librarian, Duke University, Durham, North Carolina

**Anu Moorthy,** Associate Director of Content and Discovery, Duke University Medical Center Library and Archives, Durham, North Carolina

**Objectives**: Open access (OA) publishing represents an appealing alternative to high collection costs and tight budgets. To facilitate a better understanding of researcher motivations, this analysis seeks to answer the following questions: Does an institutional fund (known as the COPE fund) supporting article processing charges make a difference in researchers' decisions to publish via OA and what motivates researchers to pay for gold OA?

**Methods**: The analysis consists of two parts: descriptive analysis of the institutional OA fund requests and a bibliometric analysis of institutional gold OA publications. Follow-up surveys identified author motivations.

Part one: The institutional OA fund provides limited reimbursement for article processing charges accrued when publishing in fully OA publications. Many requests are denied due to limited funding or type of journal. Requests from academic biomedical researchers were analyzed by role, amount requested, reason for denial, and nature of final publication. Follow-up surveys were conducted to determine researchers' funding options and publication behaviors.

Part two: Through a bibliometric analysis in Web of Science, institutional OA publication patterns were determined. In addition, cross-referencing these results with institutional subscriptions identified those articles for which researchers chose to pay optional OA charges. Follow-up surveys were conducted with these authors to determine their motivations.

**Results**: There were 184 applications (144 unique applicants) to the COPE fund. Most applicants were Assistant or Associate Professors and departments represented were proportional to institutional demographics. Survey results of applicants indicated that most had previous experience with open access publishing. Primary motivations included journal fit, greater potential impact, and colleague recommendations. Analysis of hybrid OA publications showed over 1,200 articles from 2016-2019. Research areas represented among resulting articles mirrored institutional research priorities and were proportional to overall institutional publishing trends. First and last authors were surveyed and primary motivations were personal commitment to open access and greater potential visibility and access.

**Conclusions**: A majority of both groups reported previous experience with open access publishing. Compliance with institutional and NIH Public Access Policy were not reported to be primary motivations for publishing as open access.

Thursday, August 13, 11: 30 a.m.–3: 00 p.m.

---

# Mixed Domains Session 2

Moderator: Zoe Pettway Unno

## Biomedical Reproducibility Workshop Series

**Ariel Deardorff,** Data Services Librarian, UCSF Library, San Francisco, California

**Background:** Given the growing attention to reproducibility in the biomedical sciences there is an increasing need for hands-on reproducibility training for biomedical researchers. To address this need, the Library, Graduate Division, and Open Science Group of University of California, San Francisco (UCSF) collaborated in the Fall of 2019 to create a for-credit workshop series on biomedical reproducibility. The series was targeted at graduate students and postdocs, and aimed to translate recommendations for best practice into actionable steps and training.

**Description:** The eight-part workshop series was designed to meet the Rigor and Reproducibility requirements of the National Institutes of Health, and included sessions on open data, open code, open protocols, open access, designing rigorous experiments, engaging with new forms of peer review, and building a reproducible lab. Sessions were taught by experts from UCSF as well as outside experts in reproducibility and open science. In order to measure the effectiveness of the course, we conducted pre and post workshop assessments to compare attendees' knowledge of reproducibility before and after the series, and to measure uptake of reproducible practices. The larger goal of this project was for subject experts to provide hands-on training that will improve research workflows, stimulate conversations about open science and research reproducibility, and build an open curriculum that can be replicated by other institutions.

**Conclusion:** After completing the workshop series participants indicated they had a more extensive understanding of the workshop topics and were much more likely to talk with their lab about reproducibility, examine their research for potential bias, and share their research code. In addition, 50% of participants mentioned that they intended to keep better records and spend more time on documentation because of the series. Given the success of the workshops, the Library plans on updating the curriculum so it can be delivered as a virtual series in Spring 2021.

Designing and Delivering a Program for Staff Professional Development in an Academic Biomedical Library

**Amanda Scull,** Head of Education and Information Services, Dartmouth College Biomedical Libraries

**Background**: The Biomedical Libraries have recently undergone a shift in how research and information services are provided, with staff providing more ready reference services and utilizing the LibAnswers and RefAnalytics systems to triage and track user interactions. These are new expectations for non-librarian staff, who comprise a mix of long-serving and newer staff members. It was necessary to assess competencies and design training opportunities for staff with the primary objective of increasing confidence and skill with providing reference services. As a secondary objective we sought to foster a new culture of learning and ongoing professional development in the libraries.

**Description**: A month-long training series for LibAnswers and RefAnalytics was delivered to prepare staff for the system transition, and conversation during that training as well as review of preliminary data from RefAnalytics revealed additional areas in need of development. The expansion of the

professional development program for staff included the design and implementation of a Consumer Health training, a Ready Reference Round Up, and the establishment of a monthly Biomedical Libraries Lunch and Learn in which any staff member can present on a topic of interest and expertise to their colleagues. The most substantial undertaking has been a competency-based training program for Information Access Assistants (IAAs), a self-paced course delivered through Canvas that each IAA completes to achieve baseline competency expectations. This course is being used for assessment and development of current staff as well as for training new IAAs.

**Conclusion**: The staff professional development program has been successful in encouraging collaborative learning and increasing staff confidence and skill with patron interactions. Staff members have welcomed the opportunities to learn and develop skills, and initial assessment of the data in RefAnalytics indicates quality improvement in reference interactions and less transferring of questions to librarians. Additional trainings are planned for the coming year and assessment is ongoing, particularly for the competency-based training program.

## Building a Workforce for Data-Driven Research and Health: The National Library of Medicine Data Science Training Program

**Lisa Federer, AHIP,** Data Science and Open Science Librarian, National Library of Medicine, North Bethesda, Maryland

**Dianne Babski**

**Peter Cooper,** Staff Scientist, U.S. National Library of Medicine / National Center for Biotechnology Information, Bethesda, Maryland

**Anna Ripple,** Information Research Specialist, U.S. National Library of Medicine / Lister Hill National Center for Biomedical Communications

**Background**: The National Library of Medicine’s (NLM) 2017 – 2027 Strategic Plan outlines three broad goals, one of which is to “build a workforce for data-driven research and health.” Realizing this vision requires NLM staff to also develop their own data science skills and expertise. The Data Science @NLM (DS @NLM) Training Program was a year-long initiative to provide staff opportunities to enhance their skills and knowledge related to data science. The program’s main goal was not to turn all NLM staff into data scientists, but to provide a common vocabulary and understanding of data science principles regardless of job title.

**Description**: The DS @NLM Training Program included NLM-wide activities, including a Data Science Basics training session, individual training plans (ITP) for all staff, and a pilot intensive training course for selected staff. Staff members selected one of eight personas developed to cover the skills needed in various roles. The ITPs were developed by identifying gaps between the staff member’s knowledge as self-reported in a Data Science Readiness Survey and the skill level of the selected persona. Staff members were then provided a list of courses tailored to their unique needs from a catalog of nearly 250 courses. The year-long initiative culminated with a Data Science Open House, where staff shared how they had used new data science skills and considered how they could continue applying data science to NLM’s work.

**Conclusion**: The DS@NLM Training Program helped to increase staff’s understanding of data science and why it is important to NLM’s mission. NLM leadership and supervisor engagement played a role in staff success; staff who reported receiving support from their supervisor in selecting their persona and courses were almost seven times more likely to have made progress on or completed their ITP. Feedback suggested ongoing interest, with nearly half of respondents to the post-program survey

indicating interest in further intensive, hands-on data science training. Future potential activities include mentoring to help connect NLM staff with their colleagues to learn about data science.

## Envisioning Success: Breaking Down Silos to Engage throughout the Library and Grow Institutional Impact

**Megan G. Van Noord,** Health Sciences Librarian, University of California, Davis, Davis, California

**Nicole Capdarest-Arest, AHIP,** Head, Blaisdell Medical Library, University of California, Davis, Sacramento, California

**Background**: In the academic library landscape, it can be challenging to work across multiple units to create programmatic innovations. At a university with a large library system, including two health sciences libraries operating within the larger organizational construct of the main campus library, we had come to a place wherein, due to organizational structures and “habits of comfort,” groups were not connecting as departments functioned largely as disconnected silos. A reorganization and contiguous personnel changes provided opportunities to bridge the gaps across silos, resulting in better teamwork across units, and also significant impacts to establishing new services to the library’s communities.

**Description**: Breaking down silos in the library and intentionally seeking out cross-functional partnerships to meet service goals catalyzed our services to our external constituents in a way that would have not been possible for each unit/silo individually. We worked across campus libraries - using videoconference as an integral tool - and research units to create a multi-unit service framework model to launch: a systematic review service with enhanced meta-analysis/text analysis features, a bibliometrics service, and health science data services - all in under a year’s time. This ability to co-create with groups within and external to the library to create multiple new projects that positively impact the university mission are notable and appreciated, as exemplified by this feedback: “...has made a difference at our university, exemplified the mission of institutional cross-partnerships, and has positively impacted our research mission.”

**Conclusion**: It is easy to become ingrained in institutional structures and to function in comfortable silos. By re-envisioning organizational challenges as opportunities for innovation, we created new user-centered service frameworks that facilitated service growth and demonstrated impact of the library to the institution. Keys to success included: administrative and team member buy-in that user needs are best met through interdepartmental collaboration; creating shared goals and gaining team consensus around project vision and scope; willingness to craft teams based on experience and skillset, rather than by department, and/or including team members external to the library; openness to different perspectives and problem-solving approaches.

Thursday, August 13, 11: 30 a.m.–3: 00 p.m.

---

# Professionalism & Leadership Session 3

Moderator: Fatima M. Mncube-Barnes

## Understanding Library Student Perspectives on Gaining Impactful, Career-Building Professional Experiences

**Tova Johnson,** Health Sciences Librarian, Oregon Health & Science University, Portland, Oregon

**Kristine M. Alpi, AHIP,** University Librarian, Oregon Health & Science University, Portland, Oregon

**Kourtney Foley,** Volunteer, Oregon Health & Science University, Vancouver, Washington

**Robin Champieux,** Direction of Education, Research and Clinical Outreach, Oregon Health & Science University, Portland, Oregon

**Objectives**: In February 2019 at OpenCon Cascadia, we discussed how unpaid internships can impact equity and diversity in our professions. We sought to understand the perspectives of students working towards a library science Master’s degree on the challenges and benefits associated with gaining practical experience, with the goal of eventually comparing student and provider perspectives to improve internship structure and recruiting.

**Methods**: This ongoing pilot study is recruiting distance library students based in the urban Portland, Oregon area through email lists for three distance MLS programs and word of mouth. Initial in-person and virtual interviews began with questions based on the OpenCon Cascadia discussion, followed by optional, but critical, demographic items related to available financial resources, work obligations, and time constraints, sometimes expanding to additional issues surfaced by participants. After saturation from individual interviews, we will refine our interview guide and hold focus groups with additional local students. Two investigators will independently code and analyze the individual and group transcripts for themes, and the consensus findings will inform a future anonymous survey of a broader range of students, as well as comparison with findings from a separate survey of providers of practical library experiences.

**Results**: The study is paused, and results are not yet available.

**Conclusions**: As only the pilot has been completed, no conclusions can be drawn at this stage of the research.

## Leaders’ Ways of Knowing about Leadership

**Rick L. Fought, AHIP,** Director, University of Tennessee Health Science Center, Memphis, Tennessee

**Mitsunori Misawa,** Associate Professor & Associate Department Head, The University of Tennessee, Knoxville, Knoxville, Tennessee

**Objectives**: The purpose of this study was to better understand how academic health sciences library directors experience leadership and how their experience related to their understanding of effective leadership. The research question that guided this inquiry was: How do academic health sciences library directors understand their leadership and experiences as library leaders?

**Methods**: Qualitative phenomenological research was selected for the research design due to its focus on exploring and understanding the meaning individuals ascribe to a particular phenomenon

or experience. A part of the interpretivist theoretical perspective, phenomenology searches for the essence of a phenomenon from people’s shared experience of it and works particularly well with phenomena that does not lend itself to easy quantification, like leadership.

The study used purposeful sampling and criterion-based sampling strategies to select its participants. Eleven library directors from academic health sciences libraries at public universities with a RU/VH Carnegie Classification agreed to participate in the study. They also met all other selection criteria for the study. Data were collected through two semi-structured interviews with each participant. The data were later transcribed and coded. Thematic analysis was used to analyze the data from which categories and themes emerged.

**Results**: A major theme that emerged from the data was the participants’ understanding of effective leadership, which we described in five categories: (1) What it takes, (2) Building a team, (3) Advocacy and credibility, (4) Awareness of your environment, and (5) Creating a vision. These five categories bring together a variety of aspects of the leadership experience expressed by the research participants during their interviews. These categories represent the most important components of effective library leadership based on the shared experiences of the research participants.

**Conclusions**: Leadership in higher education is complex and unique, and a good evaluation tool for leadership effectiveness in higher education remains elusive. Therefore, the intent of our research question was to capture the essence of the participants’ leadership experiences as library directors at academic health sciences libraries and determine what could be learned about effective leadership from these shared experiences. The categories that emerged from the data provide very useful guidance for being a more effective library leader and how to consider your own leadership situation.

## A Comparison of Health Sciences Librarianship Job Qualifications and MLA’s Competencies for Lifelong Learning and Professional Success

**Jodi L. Philbrick, AHIP,** Senior Lecturer, Department of Information Science, University of North Texas, Denton, Texas

**Ana D. Cleveland, AHIP, FMLA,** Regents Professor, Sarah Law Kennerly Endowed Professor, & Director, Health Informatics Program, University of North Texas, Denton, Texas

**LeAnn Boyce,** Student, University of North Texas, Pilot Point, Texas

**Objectives**: 1. What are the required and preferred qualifications listed in job announcements posted on MEDLIBS-L from July 2, 2018 to July 2, 2019? and 2. How do the required and preferred qualifications compare to the MLA's Competencies for Lifelong Learning and Professional Success?

**Methods**

201 job announcements were collected from July 2, 2018 to July 2, 2019 from MEDLIBS-L and entered in to a Microsoft Excel spreadsheet. Twelve job announcements were excluded from analysis due to incomplete information, leaving 189 complete job announcements. The posting date, position title, type of institution, required qualifications, preferred qualifications, geographical location, and salary (if available) were entered into the spreadsheet from each job announcement. Content analysis of the required and preferred job qualifications was performed using a codebook developed by the authors. Descriptive statistics, including frequencies and bivariate analysis, were completed. The most frequent required and preferred job qualifications were compared with the competency areas in MLA's Competencies for Lifelong Learning and Professional Success to

determine to see if what employers want are in alignment with the association's skills and abilities recommendations.

**Results**: Over two-thirds of the job announcements were for positions in academic libraries, and the rest were for positions in hospital, government, or corporate/organization libraries. The top ten required qualifications were (1) education; (2) having a degree from an accredited program, (3) communication skills; (4) collaboration and teamwork skills; (5) having prior experience; (6) interpersonal skills; (7) technology competency; (8) instructional skills; (9) knowledge of information resources; and (10) management skills. The top ten preferred qualifications were (1) education; (2) technology competency; (3) having prior experience; (4) instructional skills; (5) knowledge of the health environment; (6) professional development; (7) collaboration and teamwork skills; (8) data management; (9) communication skills; and (10 – tied) computer applications and scholarly communication.

The top ten required and preferred qualifications aligned with all but one MLA’s Competencies for Lifelong Learning and Professional Success – Competency 5: Evidence-based Practice & Research.

**Conclusions**: The analysis of 189 job announcements identified that employers want individuals with education (ranging from bachelors to doctoral degrees); prior experience; communication skills, collaboration and teamwork skills; technology competency; and instructional skills. Education was the number one required and preferred qualification. Competency 5: Evidence-based Practice & Research of MLA’s Competencies for Lifelong Learning and Professional Success did not align with any of the top ten required and preferred job qualifications.

## Attitudinal Attributes of Professionalism in Health Sciences Librarians

**Rachel Amelia Santose Koenig,** Research and Education Librarian, Tompkins-McCaw Library for the Health Sciences, VCU Libraries, Richmond, Virginia

**Objectives**: The establishment of professional credentials within health sciences librarianship has become an important initiative within the occupation; however, the examination of attitudinal attributes associated with professionalism in individual librarians has been left primarily unacknowledged. This study will determine current attitudes toward professionalism in health sciences librarians and examine relationships among degrees of professionalism.

**Methods**: A cross-sectional online survey was carried out among participants registered on several library professional listservs. The survey collected socio-demographic information as well as information about work settings and work responsibilities. The Hall Professionalism Inventory, a 25-item five-point Likert scale, was used to measure participants’ overall professionalism and five attitudinal dimensions of professionalism. The study utilized a descriptive comparative-correlational design to describe overall professionalism, the attitudinal attributes of professionalism, and the degree to which professionalism and its attributes are present in health sciences librarians. Descriptive statistical analyses were used to describe sample characteristics through the use of frequencies and percentages for categorical/nominal data and Professionalism scores and subscores (ANOVA, Pearson Product correlations). These analyses additionally identified differences in professionalism scores among different librarian characteristics.

**Results**: 430 participants completed the survey. The mean age of the participants was 46.5 years and the average years of experience in a health sciences library was 13.7 years. Most of the participants were women (87.3%), Caucasian (89.8%), held a Master’s degree in Library Science (90.7%), and worked full-time (95.3%) in an academic setting (73.4%). Overall, the average professionalism score was 83.1 out of 125. Today’s health sciences librarians scored highest in the dimensions of sense of

calling and autonomy, whereas service to the public was the dimension that prompted the weakest response. Those librarians working in more traditional librarian roles, such as in collection development and access services, scored the highest on overall professionalism, while research librarians or subject specialists, those roles where librarians immerse themselves in outside disciplines, scored the lowest.

**Conclusions**: A number of factors influence the attitudes health sciences librarians feel toward professionalism, including their educational background, workplace setting, participation in professional organizations, and years experience. A deeper understanding of these factors allow us to grasp the personal and environmental factors that influence the occupation and gain a better insight into the ways in which current health sciences librarians feel about their place in society.

Thursday, August 13, 12: 00 p.m.–1: 00 p.m.

---

## PubMed Update - Thursday's Video Chat

Marie Collins

Mike Davidson, Librarian, Office of Engagement and Training (OET), National Library of Medicine (NLM)

Molly Knapp, AHIP, Training Development Specialist, NNLM Training Office, Houston, Texas

Marie Collins from the National Library of Medicine provides the latest news on PubMed for the Medical Library Association vConference 2020. Marie highlights the improved mobile experience, the “Best Match” sort, interface improvements, and where to go for support and training.

Thursday, August 13, 3: 15 p.m.–4: 30 p.m.

# Information Management Immersion Session 2

## Part II: Revisioning Data Visualization Services and Training: Data Storytelling and Data Visualization Lessons from the Field

**Nancy Shin,** Research and Data Coordinator, NNLM PNR, Seattle, Washington

**Catherine Tess Grynoch,** Research Data & Scholarly Communications Librarian, University of Massachusetts Medical School, Worcester, Massachusetts

**Fred Willie Zametkin LaPolla,** Research and Data Librarian, Lead Data Education, NYU Health Sciences Library, New York, New York

**Sally Gore,** Manager, Research & Scholarly Communication Services, University of Massachusetts Medical School, Worcester, Massachusetts

**Session Format:** Our immersion session will be a presentation followed by roundtable discussions that will be lead by the four presenters. Participants will have the choice as to which activities to participate in based on their interest level.

**Objective:** This immersion session is a continuation of MLA 2019’s immersion session entitled “Part I: Establishing a Successful Data Visualization Service: Data Visualization Lessons from the Field”. The librarians leading Part II of this immersion session plan on covering the best practices for data visualization and data storytelling with the health librarian in mind. Each librarian will also reflect on their experiences developing data visualization services in the library, training different user groups, and revisioning the role of librarians in data visualization. The lessons will be followed by roundtable discussions and activities centered on real-life and memorable librarian data scenarios. Data visualization is an emerging service in health sciences libraries and this session will appeal to librarians who are interested in gaining data visualization skills and the tools to set up a basic data visualization service or data visualization training at their respective institutions.

**Instructional Methods:** The session will feature a mixture of theory and hands-on learning supported by instructional scaffolding of the material.

**Participant Engagement:** Participation will be encouraged throughout the presentation part of the session with questions posed to the group and responses/answers collected as a group to appeal to all people regardless of their public speaking comfort level. The small breakout sessions will also reduce anxiety of speaking to the larger group and will encourage more shy people to speak up in the small group setting. The session will end with small groups reporting back to the larger group.

**Sponsors:** Data Caucus and Research Caucus.

Thursday, August 13, 3: 15 p.m.–4: 30 p.m.

# Information Services Immersion Session 2

## Transforming Open Access (OA): How We Did It and Are Doing It

**Rikke Sarah Ogawa, AHIP,** Director, Louise M. Darling Biomedical Library and Science and Engineering Library, Los Angeles, California

**Rice Majors,** Associate University Librarian, UC Davis, Davis, California

**Chris Shaffer, AHIP,** University Librarian, University of California, San Francisco

**Sarah McClung,** Head of Collection Development, UCSF Library, San Francisco, California

**Bruce T. Abbott,** Librarian, University of California, Davis, Sacramento, California

**Wynn Tranfield,** Physical and Basic Sciences Librarian, Louise M. Darling Biomedical Library, UCLA, Los Angeles, California

**Session Format:** Panel presentation including Q&A with the audience and breakout working groups facilitated by panelists and/or experts in the field to develop practical steps to work towards transformative publishing models applicable to your institution.

**Objective:** The first half of the session, a panel discussion with stakeholders, provides a multi-campus system perspective, a dean’s perspective, and a line librarian’s perspective on what it takes to do consortia agreements for transformative open access change (agreements that shift away from subscription based licensed content toward open access publishing models). The panel will lay the foundation for librarians at a variety of institutions to share how their individual and collective work can plant the seeds for change in publishing. The session’s second half will include breakout discussions of practical steps that MLA members want to or can do at their institutions to help bring change at their institution. The foci of the practical approaches discussion will be around collection building in the health sciences, advocating for OA policies at an institutional level, and creating a culture of scholarly communication issue awareness at your institution.

**Instructional Methods:** Pre-session handout (also available at the meeting): definition of terms related to collections, scholarly communication, and publishing

**During Session:** Panelists will present a brief history of one consortium’s model for transformative publishing agreements from multiple perspectives including: 1) a line-librarian assisting patrons, 2) a campus who is balancing collecting and service priorities, 3) a multi-campus perspective of creating sustainable longer-term change in the economy of scholarship. The panelists will be led through a moderated, multi-layered discussion that will shift into an open Q&A time for the audience. Recognizing that not everyone has the same level of interest in advocacy, the second half of the session will be spent in small break out groups led by panelists and experts discussing specific aspects of the transformation, e.g., the consortia perspective and how to develop institutional buy-in for change; communication and meeting patron expectations during seasons of uncertainty and change; setting collection priorities and negotiating. The small group discussion goal is for attendees to create action plans for championing open access that adjusts to their own institutional parameters.

**Participant Engagement:** With the help of Q&A moderators, we will gather questions via the MLA app and write-in cards during the session. We will use the direction of the Q&A and audience polling to adjust breakout group topics.

**Sponsors:** University of California Libraries, Collection Development Caucus

Friday, August 14, 11: 30 a.m.–3: 00 p.m.

# Roles to Play: Open Science & Health Sciences Librarians

Moderator: Erin Diane Foster, Robin Champieux

## Roles to Play: Open Science & Health Sciences Librarians

**Erin Diane Foster,** Research Data Management Program Service Lead, University of California, Berkeley, Berkeley, California

**Robin Champieux,** Direction of Education, Research and Clinical Outreach, Oregon Health & Science University, Portland, Oregon

**Lisa Federer, AHIP,** Data Science and Open Science Librarian, National Library of Medicine, North Bethesda, Maryland

**Antoinette Foster,** PhD student, Oregon Health & Science University, Portland, Oregon

**Jessica Polka,** Executive Director, ASAPbio

**Kari Jordan,** Executive Director, The Carpentries

**Angela Okune,** PhD student, UC Irvine

Open science is the movement to make research and its dissemination accessible to all. Its practices facilitate reproducible and reusable knowledge that is shared and developed through collaborative networks. At the heart of open science is the belief that an open exchange of ideas accelerates scientific progress and creates a more inclusive research ecosystem. In a panel discussion and breakout sessions, community leaders, researchers and librarians will share their perspectives on open science, its benefits and issues, and the role of health sciences libraries and the medical librarian in realizing its goals. The session will also focus on the role of open science in light of current events, specifically the COVID-19 pandemic, systemic racism, and social justice for BIPOC (Black, Indigenous, and People of Color).

**Panel presentation**

The panel to start this session will address what we mean by “Open Science” with panelists that reflect expertise and perspectives on different aspects of open science and the role of libraries.

**Panelists**: Lisa Federer, Antoinette Foster, Angela Okune, Jessica Polka, and Kari Jordan

**Breakout sessions**

The break-out sessions are concurrent and attendees can choose which to attend following the panel. Each breakout session will be an hour long and feature 2-3 experts who will speak to the topic as described below with brief presentations. Following that, there will be a moderated discussion with the speakers as well as an opportunity for audience Q&A.

**Open Science Breakout Session One: Field Notes: Medical Librarians’ Stories of Open Science Education and Advocacy**

This breakout will focus on efforts to incorporate open science practices into education and how that work fits in with the changing policy/publishing landscape. The session will also touch on advocacy roles for all when it comes to advancing open science.

**Moderator**: Erin Foster

**Speakers**: Peace Ossom-Williamson, Emily Ford, Ashley Farley

**Open Science Breakout Session Two: Tool Talk: Open Technologies and the Role of Librarians**

This breakout will highlight the experience of several experts in use of tools that facilitate open science. This session will touch on how these “open tools” fit into the open science landscape and how health sciences librarians can support open technologies.

**Moderator**: Lisa Federer

**Speakers**: Vicky Steeves, Ted Laderas, Kristi Holmes

**Open Science Breakout Session Three: Race, Equity and Open Science**

This breakout will be focused on examining open science and scholarship through a critical lens, specifically addressing how the open science community and its goals and practices reflect and perpetuate racism and white supremacy, and how we can change this and leverage OS practice to dismantle these issues.

**Moderator**: Robin Champieux

**Speakers**: Lucille Moore, Charlotte Roh

Friday, August 14, 1: 30 p.m.–2: 30 p.m.

---

# Open Science Breakout Session One: Field Notes: Librarians’ Stories of Open Science Education and Advocacy

Moderator: Erin Diane Foster

## Open Science Breakout Session One: Field Notes: Librarians’ Stories of Open Science Education and Advocacy

**Erin Diane Foster,** Research Data Management Program Service Lead, University of California, Berkeley, Berkeley, California

**Peace Ossom Williamson, AHIP,** Director for Research Data Services, University of Texas at Arlington, Arlington, Texas

**Emily Ford,** Portland State University

**Ashley Farley,** Gates Foundation

**Panel presentation**

The panel presentation prior to the start this of this session will address what we mean by “Open Science” with panelists that reflect expertise and perspectives on different aspects of open science and the role of libraries.

**Breakout sessions**

The break-out sessions are concurrent and attendees can choose which to attend following the panel. Each breakout session will be an hour long and feature 2-3 experts who will speak to the topic as described below with brief presentations. Following that, there will be a moderated discussion with the speakers as well as an opportunity for audience Q&A.

**Field Notes: Medical Librarians’ Stories of Open Science Education and Advocacy [Session 1]**

This breakout will focus on efforts to incorporate open science practices into education and how that work fits in with the changing policy/publishing landscape. The session will also touch on advocacy roles for all when it comes to advancing open science.

Friday, August 14, 1: 30 p.m.–2: 30 p.m.

# Open Science Breakout Session Three: Race, Equity and Open Science

Moderator: Robin Champieux

## Open Science Breakout Session Three: Race, Equity and Open Science

**Robin Champieux,** Direction of Education, Research and Clinical Outreach, Oregon Health & Science University, Portland, Oregon

**Lucille Moore,** Postdoctoral Research Fellow, Oregon Health & Science University, Portland, Oregon

**Charlotte Roh,** Scholarly Communications Librarian, University of San Francisco

**Panel presentation**

The panel presentation prior to the start this of this session will address what we mean by “Open Science” with panelists that reflect expertise and perspectives on different aspects of open science and the role of libraries.

**Breakout sessions**

The break-out sessions are concurrent and attendees can choose which to attend following the panel. Each breakout session will be an hour long and feature 2-3 experts who will speak to the topic as described below with brief presentations. Following that, there will be a moderated discussion with the speakers as well as an opportunity for audience Q&A.

**Race, Equity, and Open Science [Session 3]**

This breakout will be focused on examining open science and scholarship through a critical lens, specifically addressing how the open science community and its goals and practices reflect and perpetuate racism and white supremacy, and how we can change this and leverage OS practice to dismantle these issues.

Friday, August 14, 1: 30 p.m.–2: 30 p.m.

# Open Science Breakout Session Two: Tool Talk: Open Technologies and the Role of Librarians

Moderator: Lisa Federer, AHIP

## Open Science Breakout Session Two: Tool Talk: Open Technologies and the Role of Librarians

**Lisa Federer, AHIP,** Data Science and Open Science Librarian, National Library of Medicine, North Bethesda, Maryland

**Vicky Steeves,** New York University

**Kristi L. Holmes,** Library Director, and Associate Professor of Preventive Medicine, Health and Biomedical Informatics Division, Galter Health Sciences Library & Learning Center, Chicago, Illinois

**Ted Laderas,** Post-doctoral Research Fellow, OHSU/Department of Medical Informatics and Clinical Epidemiology, Portland, Oregon

**Panel presentation**

The panel presentation prior to the start this of this session will address what we mean by "Open Science" with panelists that reflect expertise and perspectives on different aspects of open science and the role of libraries.

**Breakout sessions**

The break-out sessions are concurrent and attendees can choose which to attend following the panel. Each breakout session will be an hour long and feature 2-3 experts who will speak to the topic as described below with brief presentations. Following that, there will be a moderated discussion with the speakers as well as an opportunity for audience Q&A.

**Tool Talk: Open Technologies and the Role of Librarians [Session 2]**

This breakout will highlight the experience of several experts in use of tools that facilitate open science. This session will touch on how these "open tools" fit into the open science landscape and how health sciences librarians can support open technologies.
