# Supplementary material for: 120th Virtual Annual Meeting and Conference: Medical Library Association, Inc. May 19, 2020, and July 27–August 28, 2020
Source: J Med Libr Assoc. 2021 Jan 1;109(1):E1–E40. doi: 10.5195/jmla.2021.1169 (PMC7781040; doi:10.5195/jmla.2021.1169)
Supplement: Supplementary file 2 — Appendix: MLA '20 Poster Session Abstracts [file jmla-109-1-E1-s02.pdf]

Medical Library Association MLA ’20 vConference Poster Abstracts

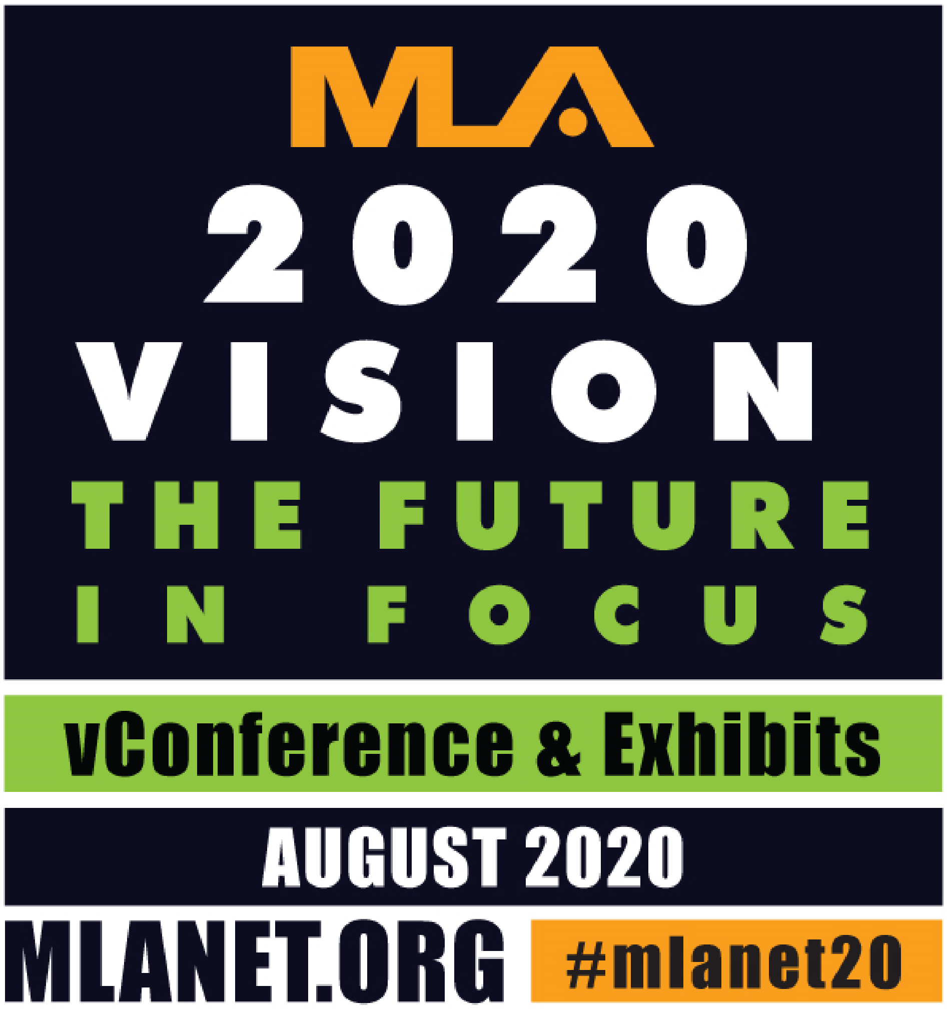

Abstracts for the poster sessions are reviewed by members of the Medical Library Association National Program Committee (NPC), and designated NPC members make the final selection of posters to be presented at the annual meeting.

Poster Number: 1

Time: Friday, July 24, 10:30 a.m.–4:30 p.m.

Developing an Evidence-Based Practice Tool in Accord with the Next-Generation Search Interfaces

Vivian Chiang, Student, EMBA of National Chengchi University

Background: In response to the demand of 2-year Post Graduate Year Training, which was established for the first new graduate of medical schools in XXX, for the implementation of EBP and Shared Decision Making, as well as the trend of cultivating nurse practitioners to carry out EBP, the medical libraries in XXX provide evidence-based literature retrieval skill training. However, most of the interface provided by the database is Google-like, which is different from the PICO framework. Therefore, this project hopes to create a tool to use the PICO framework to retrieve the most precise literature without having the advance research skill.

Description: The concept of interface design is based on PICO framework. With the function of mapping with medical subject heading, it helps users to increase the correctness of search strategy and also revised search strategy simultaneously. In addition, in order to meet the clinical needs, the clinical cases related elements such as age, gender, pregnancy, etc. are also provided in the literature filters; the conditions for selecting evidence-based literature are also provided, such as clinical queries, publication types, language, etc. All the search results will display after data deduping which also help users to do precious search strategy and retrieve data which are all evidence-based supported. According to the feedback from users, the integration workflow of EBP is easy to retrieve more clinical r literature and the usage of the database is increased by 48% in 2 years.

Conclusion: A medical center promoted this tool to medical staff through education and training in 2019. It was found that this tool helps to increase the efficiency of clinical related data retrieval with less time spending under the structure search interface design for PICO learning purposes. This tool also enhances the ability to understand and analyze clinical problems by simulating PICO framework and term mapping to MeSH. The implementation of this tool allows medical librarians to help hospital implement EBP in a more critical role in clinical practice.

Poster Number: 2

Time: Friday, July 24, 10:30 a.m.–4:30 p.m.

Family Medicine Clinic and Librarian Collaboration to Improve Patient Access to MyChart and Reduce Inappropriate Polypharmacy

William Olmstadt, AHIP, Executive Director, Health Sciences Library, Health Sciences Library, Shreveport, LA; **Kabiul Haque**, Resident physician, Family Medicine, Ochsner LSU Health Shreveport, LA, Shreveport, LA; **Marc Chaudoir**

Objectives: National surveys report the prevalence of polypharmacy in the U.S. is 15%. Inappropriate polypharmacy is preventable. Left unchecked, it increases risks for falls and fatal complications. This study explored if increased patient use of an online portal reduced unnecessary polypharmacy.

Methods: Authors conducted the study in fall 2019 in a family medicine clinic at a public medical school in the southern United States. Of the 12,000 clinic visits per year, 60% of patients use Medicaid and 70% are African American. The institutional review board declared the study exempt. During clinic visits, 65 patients not already using MyChart expressed interest in learning more about it. Of those 65, 40 randomly-chosen patients, ages 30-65, received 30 minute MyChart overviews with clinic staff, including written instructions about the sign-up process. Follow-up interviews, conducted a week later after phone and letter reminders, asked if patients had reviewed their medications in MyChart, and probed for barriers to using it. Data coding and analysis were completed by 2 physicians, one who was the patient’s provider and one who was not. Both met to resolve coding differences.

Results: All 40 patients completed the follow-up interview. 33 patients completed MyChart sign up and reviewed their medications with the physician. Of those 33, 5 had unnecessary prescriptions, and for 2 of those 5, the potential drug interactions were serious. The top three barriers to MyChart use were lack of knowledge about MyChart and its features, lack of computer or smartphone access, and lack of Internet access.

Conclusions: Although limited to one clinic, the study demonstrated a brief, in-person explanation of patient portal use could reduce polypharmacy.

Poster Number: 3

Time: Friday, July 24, 10:30 a.m.–4:30 p.m.

Focus on Nursing Point-of-Care Tools: Developing Criteria for an Evaluation Rubric

Emily M. Johnson-Barlow, AHIP, Associate Professor & Regional Health Sciences Librarian, Library of the Health Sciences- Peoria, University of Illinois at Chicago, Peoria, IL; **Annie Nickum, AHIP**, Information Services and Liaison Librarian, University of Illinois-Chicago, Chicago, IL; **Rebecca Raszewski, AHIP**, Associate Professor & Information Services & Liaison Librarian, University of Illinois at Chicago, Chicago, IL; **Ryan Rafferty**, Visiting Instructor & Regional Health Sciences Librarian, University of Illinois at Chicago, Mahomet, IL

Objectives: Registered nurses have unique practice needs and many resources are marketed to support their practice. Point-of-care tools provide evidence-based information on patient care and procedures at the time of need. This study aims to review five point-of-care tools based on their coverage, content, and transparency to support selection of a point-of-care tool for the registered nurse.

Methods: Investigators selected five point-of-care tools cited in the literature: ClinicalKey for Nursing, DynaMed, Lippincott’s Advisor and Procedures, Nursing Reference Center Plus, and UpToDate. The investigators developed a rubric containing evaluation criteria based on these point-of-care tools’ content, coverage of nursing topics, transparency of the evidence, user perception, and customization of the tools for supporting nursing practice. Thirty-five identified classified nursing terminologies, NANDA (13), NIC (11), and NOC (11), were used to examine the breadth of coverage within each point-of-care tool. Four investigators independently extracted criteria using the rubric and reported descriptive statistics of the results. Results will inform the decision-making process of recommending a point-of-care tool for nurses at our academic medical center.

Results: Lippincott had the highest coverage of diagnoses (NANDA) while ClinicalKey for Nursing had strong content focused on intervention (NIC) and outcomes (NOC). Nursing Reference Center Plus provided the most well-rounded coverage of terminology. DynaMed and UpToDate were more transparent with indicating conflict of interest but included little content on Core Measures (JCAHO) or cultural competencies compared to the nursing-specific point-of-care tools. Both UpToDate and DynaMed had lower coverage of nursing terminology and care processes. User perception was evaluated, however, the criteria was deemed to be influenced by our librarian expertise.

Conclusions: None of the five tools successfully met all of the evaluated criteria. The rubric developed for this study highlights each tools’ strengths and weaknesses that can then be used to inform the decision-making process to select a point-of-care tool based on priorities and budget. Of the tools reviewed for this study, the investigators recommend utilizing two or more to provide comprehensive, evidence-based, patient care coverage and meet the diverse information needs of nurses.

Poster Number: 4

Time: Friday, July 24, 10:30 a.m.–4:30 p.m.

Focused Training for the Future of Embedded Librarianship: How to’s

Kristy E. Steigerwalt, Head of Clinical Support, University of Missouri-Kansas City, Kansas City, MO; **Marie A. Thompson**, Dental Librarian, University of Missouri-Kansas City Dental Library, Kansas City, MO; **Cynthia Flanagan, AHIP**, Clinical Medical Librarian, University of Missouri -Kansas City, Kansas City, MO

Background: In times of limited budgets many libraries are experiencing turnover and limited staffing. These circumstances necessitate established training protocols, which result in a targeted vision for and seamless transition into medical librarianship. Even the most established programs, like our Clinical Medical Librarian program at UMKC, can easily create or improve future training using a standardized template, like the one we have created.

Description: Picture this: A seasoned librarian working in the field when…BAM-attrition. Suddenly there are new hires and job reallocations. How does training begin? How can we focus it more effectively? Have you been the seasoned librarian? The librarian in training? This poster will detail one library’s journey towards developing the necessary framework for designing medical librarian training materials at their institution.

Aim: This presentation will chronicle one library’s journey from the absence of training materials to the utilization of a set protocol and template for training embedded medical librarians.

Findings: Each library’s needs are nuanced, however, we will provide the story of what worked well and what did not for three librarians at varying levels of responsibility and experience using a newly devised training protocol.

Conclusion: Interpretation: Assessment and revision of these materials using the perspective of both seasoned and newly appointed Librarians has resulted in our training template, a copy of which will be provided for modification and immediate use in your own library.

Poster Number: 5

Time: Friday, July 24, 10:30 a.m.–4:30 p.m.

Getting a Clinical Services Program Off the Ground: Successes and Challenges

Elizabeth Frakes, AHIP, Assistant Librarian for Clinical Services, University of Utah–Salt Lake City; **Shawn Steidinger, AHIP**, Assistant Librarian for Clinical Services, Eccles Health Sciences Library / University of Utah, Salt Lake City, UT; **Christy Jarvis, AHIP**, Associate Director, University of Utah–Salt Lake City

Background: Clinician-centered support has been an under-developed and under-utilized aspect of library services at the Eccles Health Sciences Library. Two newly-hired clinical services librarians have embarked on a strategy to support providers' information needs across the health system enterprise as well as throughout the geographic region via clinical decision support tools and services. Strategies include: an EHR embedded librarian service, evidence support for order sets, Evidence Based Practice committee membership, and an increase in the marketing efforts for the literature review service as well as EBM training service

Description: Newly-hired clinical services librarians reinvigorated a stalled project to launch an EHR-embedded librarian service that would integrate into the clinicians' existing workflow, thus eliminating some of the access barriers to connecting with an information expert. Upon identifying a set of 1,000+ outdated order sets, clinical services librarians met with hospital administrators to propose a collaboration in which librarians work with individual clinicians to help them evidentiate order sets when they are due for review. Sample evidence packets were provided to demonstrate the value of librarian participation in this endeavor. Hospital administrators recognized the physician time-saving benefit of librarian-provided evidence support and agreed to integrate library information experts into the review process going forward. Additionally, the librarians will provide clinical decision support expertise to the Evidence Based Practice committee. Marketing efforts will increase for the literature review service and EBM training.

Conclusion: Due to the Covid-19 pandemic, the launch of the EHR embedded service and evidence support for order sets was delayed. As of July 2020, the EHR evidence request service is being built with a planned go live of August 2020. Statistics will be collected for the EHR embedded service to evaluate whether the new service is being utilized and by whom. Surveys will be sent to evaluate satisfaction and timeliness of the evidence provision as well as elicit feedback for improvement. Clinical Services will strive to provide the highest quality evidence to the clinicians throughout the pandemic and beyond.

Poster Number: 7

Time: Friday, July 24, 10:30 a.m.–4:30 p.m.

Keep It Movin’: Facilitating Knowledge Translation amongst Nursing Staff to Reduce Practice Variation

Philip D. Walker, Director, Eskind Biomedical Library & Knowledge Management, Eskind Biomedical Library / Vanderbilt University, Nashville, TN; **Johnston Morrison, Jr.**, Senior Associate of Nursing, Vanderbilt University Hospital, Nashville, TN; **Teresa Hobt-Bingham**; **Nancy Wells**; **Rachel Lane Walden**, Reference and Instruction Librarian, Eskind Biomedical Library, Vanderbilt University, Nashville, TN; **Heather Laferriere**, Health Sciences Informationist, Eskind Biomedical Library, Vanderbilt University, Nashville, TN; **Camille L. Ivey**, Health Sciences Informationist, Eskind Biomedical Library, Vanderbilt University, Hermitage, TN

Objectives: Application of Graham et al’s Knowledge to Action Process Framework (KTA) to assess and validate the role of information services on improving nursing policy development, standard operating procedures, and reducing practice variation.

Methods: The biomedical library was approached by the medical center’s adult clinical practice committee to assist with finding evidence for developing or updating policies and standard operating procedures. The primary goals are to improve access to educational materials and reduce practice variation among nursing staff. Health Sciences Informationists are assigned topics and search the literature for high level evidence. In addition to the project’s goals, the main objective of the project was to develop a workflow and deliverable grounded in the principles of knowledge translation. To achieve this objective, a literature search was performed in PubMed and CINAHL. The overall literature base is very limited regarding the reduction of practice variation. However, we were fortunate to find valuable information by expanding the search to include evidence-based practice, evidence-based nursing, and knowledge translation.

Results: We identified Graham et al’s Knowledge to Action Process Framework as a potential model to evaluate the project. The Framework consists of two concepts (knowledge creation and action) with each comprised of several phases. A pilot search was performed with the following course of action: search process documentation, results appraisal, create a brief summary of the search results, produce a bibliography of no more than 15 results, provide all committee members with centralized access. We have completed fifteen summaries thus far and are able to map the project’s steps with all but two of the phases.

Conclusions: Identifying and utilizing the KTA Framework proved to be invaluable for our purposes because of its ability to help us apply the appropriate knowledge translation principles and achieve the overall goals and objective. Health care practitioners are obligated to provide the best possible care yet are overwhelmed by the overabundance of patient data and biomedical research. This information overload is one of several barriers to evidence-based practice. Application of Knowledge Translation models and collaborations between clinical teams and informationists have the potential to positively impact health care at all levels by transforming the evidence from barrier to enabler.

Poster Number: 9

Time: Friday, July 24, 10:30 a.m.–4:30 p.m.

Promoting Evidence-Based Medicine and Research Development through a Library-Partner Hospital Collaboration: The Reno Experience

Alexander Lyubechansky, Clinical Librarian, Savitt Medical Library, University of Nevada, Reno School of Medicine, Reno, NV; **Susan George**, Medical Education Advisor, Renown Health; **Erin Van Kirk**, Director of Nursing Education, Renown Health, Reno, NV

Background: This project describes a collaborative instructional program between the School of Medicine Library and a partner hospital’s offices of Continuing Medical Education (CME) and Nursing Education. The program aims to expand the knowledge and practice of evidence-based medicine (EBM) and research among hospital staff through a variety of innovative instructional methods, such as the creation of a “Train the Trainers” module, focusing on the Fundamentals of EBM and Research, and a series of mini-workshops to support the development of the learners' ability to formulate an answerable question, perform search of the literature, and practice the basics of critical appraisal.

Description: Since 2017, when the School of Medicine Library opened a branch location in an 800-bed regional hospital, to serve the needs of students, residents, and hospital staff, the Clinical Librarian has been involved in promoting EBM and research skills training to hospital staff by developing and teaching continuing education sessions on topics of interest to nurses, physicians, and allied health professionals. The positive feedback and immediate success of these sessions inspired us to take a more systematic approach to the promotion of EBM and research. To ensure that medical and nursing staff attain the competencies in EBM fundamentals, we initiated a four-step planning and development process: 1) Assess Training Needs; 2) Set Training Objectives; 3) Develop Comprehensive EBM and Research Development Training Program; and 4) Implement EBM and Research Development Training Program.

Conclusion: Library – Partner Hospital collaboration has had a positive effect and demonstrated the important role of the Clinical Librarian, as well as my librarian colleagues, in the EBM and Research Development training process. We would like to implement pre- and Post-test evaluation surveys as a next step, so that we can gain a better understanding of the participants’ baseline knowledge and the degree to which the librarian-led training enhanced their knowledge and skills. In addition, Savitt Medical Library is in the process of opening another branch library; once this is completed, we hope to expand the EBM and Research Development training at our new clinical site. Finally, an EBM and Research Development library guide will include links to the training modules and other resources.

Poster Number: 10

Time: Friday, July 24, 10:30 a.m.–4:30 p.m.

The Impact of Clinical Evidence Sources on Patient Care: A Literature Review of Research Based on Independent--Not Clinician-Reported--Data

Marianne D. Burke, AHIP, Associate Professor of Libraries, Emerita, University of Vermont, Dana Medical Library/ University of Vermont, Burlington, VT; **Benjamin Littenberg**, Henry and Carleen Tufo Professor of Medicine and Professor of Nursing, University of Vermont, General Internal Medicne Research, Burlington, VT

Survey studies in which health care providers are the data source, have shown that use of clinical evidence sources (CES) by clinicians has a positive impact on clinician time, diagnoses, and treatment decisions.

Objectives: 1) Identify research studies on the impact of CES use by clinicians on clinical outcomes based on independent non-provider reported data; and, 2) Assess the strength of evidence of the research identified.

Methods: Iterative searches in MEDLINE, Google Scholar, and reference lists were conducted between 2012 and 2019. Studies measuring an impact of clinical evidence sources (CES) on clinician practice and patient outcomes based on independent non-provider reported data were eligible for inclusion in this review.

Results: 12 studies met inclusion criteria. Independent data sources included patient records, patient report, and insurance data. Positive results were found for prescribing, evidence-based treatments, better diagnoses and reduced length of stay. Negative or no difference results were found in quasi-experimental and randomized trials on referral reduction, evidence-based tests ordered, readmission to hospitals, and symptom resolution. Several study design limitations were noted.

Conclusions: Despite provider-reported impact of CES on clinical outcomes, there is only mixed evidence that CES use improves provider-level or patient-level outcomes in studies with independent patient-derived data sources.

Poster Number: 12

Time: Friday, July 24, 10:30 a.m.–4:30 p.m.

The White Coat Librarian

Elizabeth Laera, AHIP, Medical Librarian, Brookwood Baptist Health, Birmingham, AL

Objectives: To determine how many librarians wear white coats when joining a patient care team on rounds and what attitudes physicians and other librarians have towards librarians in white coats.

Methods: A short survey via SurveyMonkey was distributed via various listservs and targeted at librarians practicing clinical librarianship and/or serving as part of a clinical care team. The survey gathered data on personal experiences and feelings towards librarians in white coats, and information on their clinical librarianship practices and settings. An optional section asked participants to seek input on attitudes towards non-physicians in white coats from a physician at their institution. The author also sought informal feedback from their hospital’s physicians.

Results: No results have been reported, but the author expects the results to yield quantifiable data on how many librarians are wearing white coats and the setting they practice in, plus quantitative responses that demonstrate the attitudes towards white coats from both librarians and physicians.

Conclusions: Clinical librarians have been searching for ways to be seen as part of the “team” since the 1970s. White coats have been suggested as a way to impart competence and identify librarians as part of the care team. But the white coat is a traditional symbol of a physician and there are policies and long-held opinions on who can and cannot wear a white coat. This project seeks to understand what librarians are and are not wearing white coats while rounding and if wearing one could help validate the clinical librarian as an integral part of the clinical care team.

Poster Number: 16

Time: Friday, July 24, 10:30 a.m.–4:30 p.m.

A Study of User Experiences of Our New Website: Using the Technology Acceptance Model

Shu-Yuan Siao, Head, Division of Knowledge Services, Taipei Medical University Library, Taipei, Taipei, Taiwan (Republic of China); **Tzu-heng Chiu**, Director, Taipei Medical University Library, Taipei, N/A; **Hsiao-Fen Yu**, Librarian, Main Library, Division of Knowledge Services, Taipei Medical University Library, Taipei, Taipei, Taiwan (Republic of China)

Objectives: The old TMUL website has been used for 10 years, and it is no longer sufficient for use due to the development of multimedia contents and changing user behavior. Therefore, the TMUL created a new website and launched its beta version in April 2019. This study presents results of the use’s experience survey based on Technology Acceptance Model.

Methods: A questionnaire based on TAM was designed and then sent to all faculty, student, clinical staffs of the TMU and its affiliate hospital via email. There are four dimensions and three external variables in the questionnaire. Dimensions are (1). perceived of usefulness (interface, feature, operating, context, helpful); (2). perceived ease of use (easy to use, assistance from others, easy to find information); (3). attitude toward using (like the new website, positively helpful, satisfaction with use experience); and (4). behavioral intention to use (continue to use and recommend). And external variables include (1). roles; (2). department belong to; and (3). experience of using old TMUL website. In addition, qualitative feedback were also collected. We hope to explore the use experiences / intentions and its influencing factors about the beta version, and base on the results to improve the incoming new website.

Results: The online survey was delivered to 15,496 faculties, students, medical staff of the TMU and its affiliated hospitals on May 2019. In total, 204 valid questionnaires were received with the return ratio of 1.32%. Major findings are as follows:

(1)92.6 % of respondents have ever used the new library website.

(2) “Perceived ease of use” affects “perceived usefulness”.

(3) “Perceived usefulness” and “attitude toward using” significantly effect “behavioral intention to use”.

(4) “Perceived ease of use” and “perceived usefulness” significantly effect “attitude toward using”.

(5) The difference “identity” affected “perceived ease of use”.

(6) There were no statistically significant differences in terms of “department” and “experience” in dimensions of “perceived usefulness”, “perceived ease of use”, “attitude toward using”, and “behavioral intention to use”.

Conclusions: In conclusion, the research findings fit the TAM. We also received some qualitative feedback: “The new website is simple and easy to find information”, “It is easy to operate and find papers”, “The website menu is clearer than the old one”. The respondents generally agreed that the new library website is easy to use and it’s information is useful to them. And these will result an increasing opportunity for users to use the new website continuously.

Poster Number: 17

Time: Friday, July 24, 10:30 a.m.–4:30 p.m.

Applying the Problem, Intervention, Comparison, Outcome (PICO) Format to Research Queries of Home Birth Midwifery Students

Nora Billie Barnett, AHIP, Outreach Specialist, NNLM Greater Midwest Region, Portland, OR; **Daina Dickman, AHIP**, Scholarly Communication Librarian, Sacramento State University, Sacramento, CA

Background: Home birth midwifery is a field that relies heavily on intuition and authoritative knowledge. At a college for direct-entry home birth midwifery, research queries and theses often included alternative and complementary medicine topics. Our objective was to introduce evidence based medicine principles to information literacy instruction for direct entry midwifery students. The ability to formulate answerable research questions in the PICO (Population, Intervention, Comparison, Outcome) format increases the relevancy of search results, which ultimately has the potential to improve professional practice and benefit patient care.

Description: Within a credit-bearing information literacy course, as well in individual research consultations, students were asked to define the different parts of the PICO format as it pertained to their research queries. The PICO template was then used collaboratively with students to assist them in formulating their research interests into database-searchable research questions in terms of therapy, diagnosis, etiology, prevention, and prognosis. Feedback from students was overwhelmingly positive. Students demonstrated ability to apply the PICO format to research queries and an increased ability to evaluate search results based on relevancy. They reported increased confidence in conducting searches and usefulness in returning relevant results. Faculty responded positively to the results after seeing the literature searches and research assignments students turned in after instruction sessions and consultations.

Conclusion: Student evaluations showed that midwifery students found the PICO format helpful in navigating literature searches and evaluating responses. How PICO instruction translates into long term use of evidence based medicine (EBM) practices by midwifery students is an area for further research. The PICO model can be applied to nontraditional populations of healthcare providers and to alternative and complementary medicine research queries by all healthcare providers. There is a need for EBM instruction and outreach to other nontraditional health care populations.

Poster Number: 18

Time: Friday, July 24, 10:30 a.m.–4:30 p.m.

Bioinformatics and Biology Essentials for Librarians: Past, Present, and Future

Karen L. Coghlan, Research and Instruction Librarian, Worcester Polytechnic Institute, Worcester, MA; **Molly Knapp, AHIP**, Training Development Specialist, University of Utah, NNLM Training Office, Houston, TX; **Katherine Majewski**, Librarian, U.S. National Library of Medicine / Office of Engagement and Training, Bethesda, MD; **Dana Abbey, AHIP**, Community Engagement Coordinator, National Network of Libraries of Medicine, MidContinental Region, NNLM MCR, Aurora, CO; **Bonnie L. Maidak**, Staff Scientist, U.S. National Library of Medicine / National Center for Biotechnology Information, Germantown; **Aimee R. Gogan**, Technical Information Specialist, U.S. National Library of Medicine / Office of Engagement and Training, Catonsville, MD; **Peter Cooper**, Staff Scientist, U.S. National Library of Medicine / National Center for Biotechnology Information, Bethesda, MD

Background: In the ever fluctuating skill set of health sciences librarianship, bioinformatics has emerged as a new specialization, blossoming from the rapid growth of NCBI molecular biology databases and the explosion of genomic information. As scientists and librarians, NLM must educate its users (librarians, researchers, general public) on how to use these databases to conduct research. It's a skillset that combines biology, computation, and database science. Since the early 2000s, NLM has offered some form of bioinformatics training, particularly to health science librarians. This poster describes the evolution of bioinformatics training at NLM and future directions.

Description: “Bioinformatics and Biology Essentials for Librarians” evolved from a three day in person class originating in 2006, to a hybrid in-person/ online course (2013-2016) to the current iteration: an online asynchronous course utilizing modular learning topics. In the current online version, educational technology (Guide on the Side) is utilized for learner engagement around topics that include genetics basics, current molecular lab practices, future applications, and NCBI resources such as BLAST, Nucleotide, Gene, Protein and clinical NCBI databases. Evaluation data from the last four class cohorts was collected concurrently and at a six month follow up to measure and evaluate student application and learning outcomes. Cohort graduates are added to a bioinformatics community of practice which meets bimonthly online for discussion and updates on new bioinformatic developments. To date, over 100 students have successfully completed our program.

Conclusion: The Kirkpatrick Model was used to evaluate the training. A phone interview and survey were completed 6 months after the class to assess whether the class goals were met. Students frequently identified course materials, tools and opportunity to use as enablers and lack of opportunity and/or lack of supervisor or institutional support as barriers. Common actions by students after class completion included: research support, sharing skills and/or resources with colleagues, creating bioinformatics related information resources, contacting faculty or researchers, collaboration, teaching a workshop, a publication, and launching a new bioinformatics related service.

Poster Number: 19

Time: Friday, July 24, 10:30 a.m.–4:30 p.m.

Catalysts for Community Health: Reviving the Library and Information Science (LIS) Health Sciences Curriculum

Christina Nichole Pryor, Interim Assistant Director of Library Operations & NNLM MCR Missouri Coordinator, University of Washington, University of Missouri J. Otto Lottes Health Sciences Library, Columbia, MO; **Jenny S. Bossaller**, Associate Professor, , Columbia, MO; **Denice Adkins**, Associate Professor, School of Information Science & Learning Technologies, University of Missouri, Columbia, MO; **Deborah Ward**

Background: Poverty and low health literacy affect the most vulnerable people. Both inner cities and rural areas have high rates of poverty. Poverty can cause poor health, and poverty also limits access to resources, including quality information, about health. The (INSTITUTION) received a grant from the Institute of Museum and Library Services to improve health literacy. The program is designed to 1) create networks to support information needs in health and wellbeing environments 2) train and embed health literacy librarians, and 3) to create a sustainable curriculum to promote the development of community health information specialists.

Description: C4CH offers a model of action research and education, embedding LIS students in in health and wellness settings, and training the students across several domains (LIS, public health, and health informatics) in order to distinguish their career trajectory as public health information experts. Following the belief that community transformation is best accomplished through cross-disciplinary teams, we developed a program that bridges professional boundaries and settings in order to create more holistic and inclusive services for healthy communities. Students will be embedded health librarians through fieldwork. Their culminating practicum experience requires work in both health and wellness spaces (e.g., Boys and Girls Club; rural clinics) along with their local public library, which will prepare students to assess, develop, and provide public programs using open-access health information resources, including traditional and alternative resources that meet the needs of their community.

Conclusion: No conclusions at the time since the program started in January 2020.

Poster Number: 20

Time: Friday, July 24, 10:30 a.m.–4:30 p.m.

Creating and Teaching an Annotated Bibliography Class to Medical Students

Kevin R. Block, Information Management Librarian, Health Sciences Library, Rowan University School of Osteopathic Medicine, Stratford, NJ

Background: A new curriculum implemented at the medical school for the 2019/2020 academic year includes a medical scholarship requirement. The scholarship course designed to support this requirement runs throughout the 4 years of medical school and culminates in a Capstone project. Goals for the course include exposing students to scientific inquiry, developing scientific literacy, and cultivating basic competency in written and oral scientific communication. In support of these goals Librarians were tasked during the first semester with teaching students sessions on formulating research questions, selecting and searching appropriate databases, using EndNote, and creating an annotated bibliography on a preselected topic.

Description: Students were divided into teams and charged with finding 20 relevant, reputable sources of information to help answer a research question derived from preselected topics and to create an annotated bibliography that would be graded. A librarian developed a 45 minute lecture and PowerPoint presentation instructing students on how to format and write an annotated bibliography. The librarian prepared for the class by doing a search of the library literature and the Internet for information related to teaching and writing annotated bibliographies. Students learned what comprises a good annotated bibliography, its purpose in scholarly literature, utilizing a publication style and following good citing practices. Several published annotated bibliographies on healthcare topics were identified and select annotation were also used as examples of good annotations and to illustrate the difference between an abstract and an annotation.

Conclusion: Formal evaluations from the medical students and assessment on the class are not yet available, but feedback from faculty who oversee the medical scholarship course has been positive. Based on student questions after the annotated bibliography class it became apparent that students needed more assistance in utilizing publication styles and using good citing practices than anticipated. Plans for the next academic year are to either strengthen this section of the annotated bibliography course or to create a separate session taught by a librarian on publications styles and citing practices.

Poster Number: 21

Time: Friday, July 24, 10:30 a.m.–4:30 p.m.

Critical Appraisal of Primary Literature Citations in a Drug Monograph Project for Third-Year Pharmacy Students

Hilary M. Jasmin, Research and Learning Services Librarian, University of Tennessee Health Science Center, Memphis, TN; **Skye Bickett, AHIP**, Librarian Consultant, Northside Hospital, Suwanee, GA; **Nancy Borja-Hart**, Associate Professor, The University of Tennessee Health Science Center College of Pharmacy, Nashville, TN

Objectives: To appraise the validity of randomized controlled trials (RCT) cited in drug monograph projects within a third-year pharmacy (P3) course and compare quality across two years of assignments.

Methods: In this P3 course, student groups were assigned a monograph project where they presented their findings to a mock Pharmacy & Therapeutics Committee for a formulary decision. All drug monograph assignments from 2018 and 2019 were collected and blinded by research assistants. Data extraction included each assigned drug, the decision of whether to add the drug to the mock formulary, and the accompanying references. An analysis was conducted using a 10-point critical appraisal checklist that was adapted from Malone’s Drug Information: A Guide for Pharmacists. RCTs referenced by the P3’s in their monographs were appraised by two librarians, with a Pharm.D. co-author to arbitrate. Once appraisal was complete and checklists were graded, the records were unblinded by year and compared to determine any differences in quality between 2018 and 2019 cohorts.

Results: The 2018 cohort used nearly double the RCTs that the 2019 cohort referenced. There are a number of observations that may have affected this, including curriculum adjustments, grading scales, and librarian instruction methods. Despite a lack of 2019 RCTs compared to 2018, average grades by year were similar.

Conclusions: Appropriately appraising and applying the literature to drug information questions is a key skill that pharmacy librarians are tasked with disseminating in their instruction. Given the results of this project, the pharmacy librarian will be focusing more attention on the concept of primary literature and why it matters for evidence-based pharmacy. Additionally, the grading rubric will be modified for the 2020 cohort.

Poster Number: 22

Time: Friday, July 24, 10:30 a.m.–4:30 p.m.

Developing a Sustainable Online Video Instructional Program through Lean Production Values and Assessment

Jeffery L. Loo, Clinical Librarian, UC San Diego Library, La Jolla, CA; **Korey G. Brunetti**, Asst. Director, Academic Liaison Program & Health Sciences Librarian, University of California San Diego, La Jolla, CA; **Jeffery L. Loo**, Clinical Librarian, UC San Diego Library, La Jolla, CA

Background: Online videos can support large-scale library instruction and reference consultations while reducing time and access barriers for users. However, the development of online videos entails a time investment, a learning curve, and an ongoing maintenance effort that can be more significant compared to other instructional modes. This project aimed to streamline video production and maintenance in order to establish a sustainable online instructional program. In this poster, we describe the lean production values and assessment techniques that facilitated efficient online video development.

Description: Our video production is grounded in three principles. First, create short videos; each covering a single learning objective. Second, use modular building blocks of video lectures, demos, and transcripts, which will facilitate future video revisions. Finally, rely on low-cost, self-sufficient production. This involves a production workflow that embraces low-cost technology to lower collaboration barriers. For video maintenance, we used assessment-driven methods. We rely on a periodic review cycle and survey feedback to identify necessary changes. Examining audience retention statistics help us identify high-impact areas for focused effort. Additionally, we conducted a qualitative 2x2 matrix analysis (for skill level and subject scope) to identify gaps and trends in our instructional videos. This identified strategies for future video development.

Conclusion: Through reflective practice, we observed several positive outcomes. First, short modular videos are easier to tailor instruction to different clinical audiences and to introduce self-instruction in the reference workflow. Second, our lean production flow enabled staff to work independently and efficiently such that an instructional program was developed in a relatively short amount of time. Finally, our assessment identified precise strategies for future development. In particular, to focus on high-impact learning, to customize to learner preferences, to collaborate with other disciplinary librarians for advanced information literacy skills training, and to re-use existing video content.

Poster Number: 23

Time: Friday, July 24, 10:30 a.m.–4:30 p.m.

Developing New Faculty Services through Collaboration between Librarians and Health Sciences Faculty: Accessibility Compliance

Diana Meiser, Engineering, Applied Science & Technology Librarian; **Jason Francis**, Health Science Librarian; **Shaun R. Adamson**, Education Librarian, Head of Teaching & Information Services; Weber State University, Ogden, UT

Background: Relevant literature highlights a need for accessibility accommodations in the student population. The library's mission statement reflects a commitment to providing equitable access to materials. The purpose of this project is to proactively refine digital content to meet Web Content Accessibility Guidelines. The three objectives of the program are to review the library digital course content for compliance with accessibility standards, address accessibility issues through the use of Ally accessibility verification software, and offer training for members of the campus community, in particular those in the College of Health Professions. Training materials include online LibGuides and video instruction.

Description: The University purchased verification software for all online/hybrid courses. The library took a leadership role in partnering with the disability services office to train library faculty in addressing accessibility concerns. Common issues include a lack of captioning in recorded video content, and lack of alternative text for images in files read by screen readers. The library has implemented requirements that all instructional content be accessible. Alternative text for images, reading order issues, and tagging of uploaded content were verified through Adobe accessibility software. Video lectures and instructional videos have captions added through YouTube, Camtasia, or other audio/visual software. The library's department chair evaluates compliance with an accessibility report and mandates necessary changes. The library model is expanding university-wide. Partnering with Health Sciences faculty, captioning services, and a repository of shared content is becoming available.

Conclusion: Accessibility compliance is not something that faculty are accustomed to addressing or trained in identifying until a disability accommodation is requested. The library is assuming a campus-wide leadership role based on a partnership with the disability services office. Librarians are uniquely positioned to assist with campus-wide initiatives in academic settings through their work in advocating for access to resources. This represents a new service for Health Science faculty. Based on the library's success, an open access repository of teaching materials is being constituted and new resources are being provided to meet the demand.

Poster Number: 24

Time: Friday, July 24, 10:30 a.m.–4:30 p.m.

Developing Skills for Academic Success: A Health Sciences Library and Student Services Collaboration

Julie Breanna Horwath, Electronic Resources & Instruction Librarian, Touro Colleges, Vallejo, CA; **Amy Mikala Castro**, Access Services & Instruction Librarian, Touro University California, Martinez, CA

Background: In the fall of 2018, two health sciences librarians at Touro University California began a collaboration with Academic Support Services to teach a library session in their 1-unit Pathways course offered each semester. This course is designed to help students develop skills for academic success, including time and stress management, professional communication, and study strategies. All students are welcome to enroll, but most Pathways students are identified by their program as needing additional support and referred to the course. Based on this, Academic Support determined a library session would help students improve their study and research skills, and the librarians agreed.

Description: Each semester the librarians teach a 2-hour interactive class of varying size, depending upon student interest and instructor referrals. As most students need to improve their academic performance, areas typically covered are as follows: Library website orientation; study tools for exams and self-assessment; PubMed searching; Boolean logic and filters for advanced searching; and how to read a research paper. The specific resources, tools, search examples, and other content covered in the sessions depends on the academic programs represented and the specific needs for that group of students. Time for activities and questions is prioritized, and a feedback form distributed at the end of class. To date, the students and Pathways instructors agree, the library class is well-received and provides the help and support students need to academically succeed.

Conclusion: For the past two years, two health sciences librarians have partnered with Academic Support to teach a session focused on how library resources and services support the academic success of students. This unique opportunity has helped the librarians identify the specific needs of struggling students and how the library can support them. This is significant since there is no official library orientation for most students, leaving many unaware of how the library can serve them in their academic, and eventually professional, careers. Additionally, this collaboration allows the librarians to demonstrate the value of the library and its resources—including the librarians.

Poster Number: 25

Time: Friday, July 24, 10:30 a.m.–4:30 p.m.

Engaging Community through Graphic Medicine: The Creative Experience of Hosting a National Library of Medicine Traveling Exhibit

Erin W. Palazzolo, Library Director and Professor of Biomedical Sciences; **Norice Lee**, Associate Library Director; Burrell College of Osteopathic Medicine, Las Cruces, NM

Background: Our institution hosted the National Library of Medicine’s traveling exhibit: Graphic Medicine: Ill-Conceived and Well-Drawn. Graphic Medicine is increasingly being used to help engage, encourage, and build empathy among populations of all ages. It uses text and images communicating unique perspectives on personal experiences of illness and health. Our institution is located in an area that is considered medically underserved, where educational levels are low and poverty levels are high. Our goal in hosting the exhibit was to bring awareness to another form of communication about difficult topics to both our medical students and to the greater community.

Description: This poster communicates the logistics involved, challenges encountered, and successes achieved by hosting the NLM traveling exhibit Graphic Medicine: Ill-Conceived and Well-Drawn. In conjunction with the exhibit, nine theme-based programs occurred at multiple sites for participants of all ages. Program details, photographs, and marketing and public relations strategies are included in the poster.

Conclusion: Hosting the NLM traveling exhibit provided many positive library outreach opportunities. Collectively, the initiative engaged 8,195 participants, with several activities targeting children and families. Attendee counts and program surveys provided quantitative and qualitative data used to measure impact and satisfaction.

Poster Number: 27

Time: Friday, July 24, 10:30 a.m.–4:30 p.m.

Focusing on Information Literacy Skills in Translational Health Sciences Doctoral Students

Anne M. Linton, AHIP, Director, Himmelfarb Health Sciences Library, The George Washington University, Washington, DC; **Alexandra Gomes, AHIP**, Deputy Director, Himmelfarb Health Sciences Library, Vienna, VA; **Tom Harrod, AHIP**, Research Support Librarian, Himmelfarb Health Sciences Library, Washington, DC

Background: Over the past four years, librarians enlarged their role supporting doctoral students in the new Translational Health Sciences program-- a distance education program for working professionals, with in-person residency weekends. Librarians began by consulting with faculty on resources for the program and teaching two introductory hands-on workshops. In a program focused on knowledge integration and interdisciplinary collaboration, students need strong search skills to identify research gaps and opportunities for collaboration and implementation of research. Consequently, librarian roles have evolved in this information heavy program and librarians are now fully embedded, teaching one elective and co-teaching one required course.

Description: Initially librarians developed two workshops: searching the literature at each translational stage (T1-T4) and advanced PubMed search techniques. A later session on scoping reviews was developed and a research guide on navigating the translational health sciences field developed.

Workshops were well received and the program coordinator made two requests: 1) would librarians develop a course with more intensive instruction on literature searching and topic exploration for the doctoral process and 2) would one librarian serve as co-instructor for the Foundations of Translational Health Sciences course? Three students enrolled in the literature course which targeted skills to complete an executive summary of the dissertation topic. The Foundations of Translational Health Sciences course is required for every cohort and focuses on “wicked problems.” Students explore problems from various viewpoints and critically appraise articles as they brainstorm possible solutions to the problems posed.

Conclusion: Students enter doctoral programs with varying levels of search, analysis, and integration skills. Librarians working in the THS program have extended their instruction from a focus on searching skills to a focus on thinking critically about a broad spectrum of research disciplines. Students are taught to approach the dissertation process from a global perspective and small class size has allowed instructors to individualize instruction.

Developing and teaching these courses has allowed the librarians to create new relationships with faculty and students and given them a new understanding of the doctoral process as they reach out confidently to support other programs.

Poster Number: 28

Time: Friday, July 24, 10:30 a.m.–4:30 p.m.

From Lecture to Learning Module: A Comparison of Information Literacy Curriculum Delivery

Sara K. Motsinger, Medical Librarian, Kansas City University of Medicine and Biosciences, Kansas City, MO; **Lori Fitterling**, University Library Director, D'Angelo Library, Kansas City University of Medicine & Biosciences, Kansas City, MO; **Bonnie Turner**, Medical Librarian/Instructor of Medical Informatics, Kansas City University of Medicine & Biosciences, Kansas City University of Medicine & Biosciences/Farber-McIntire Campus, Joplin, MO

Background: The Medical Informatics and Information Literacy course is a two semester, competency-based, first year student course that serves as an introduction to medical informatics, information literacy, and evidence-based practice. Skills in searching, locating, and critically appraising information, are stressed with a final lecture and project of patient case application of these skills. The course has previously been delivered with class lectures and exams, but with a university wide initiative to use active learning and integrated learning modules, as well as student feedback that indicated a preference for fewer lectures, an adapted curriculum model was begun in the fall of 2019.

Description: This adapted model reduced course lectures to three for the year and launched content using learning modules through the Canvas LMS. Course objectives and quiz questions were mapped to core competencies. This poster will compare student comments and post-course survey results as well as faculty observations about the changes in delivering course material.

Conclusion: Student evaluations and comments overwhelmingly supported the change in delivery of the course. Students reported an increase in their information literacy skills, liked the ability to return and review skills as needed as well as the freedom to work on the modules at their own pace. The various types of learning tools used to create the modules addressed the learning needs of a wider range of learners and the requirement to complete a module before moving to the next module improved engagement for students. Moving from lecture-based to active learning modules improved the delivery and overall success of the course.

Poster Number: 29

Time: Friday, July 24, 10:30 a.m.–4:30 p.m.

Gear Up for Citizen Science: Developing a Health-Focused Online Course

Alexa Mayo, AHIP, Assoc. Director for Services, Univ. of Maryland, Baltimore, Baltimore, MD; **Katherine Downton, AHIP**, Head of Research, Education and Outreach Services, University of Maryland, Baltimore, Baltimore, MD

Background: A citizen scientist is a member of the public who either works collaboratively with a scientist to gather data to solve a problem, or investigates a research question on their own. A team at the Library is developing an online course, Gearing Up for Discovery, to build research skills in citizen scientists. The Gearing Up edX course is unique in that it focuses on health-related projects, exploring citizen science through the lens of public health and environmental health. The project is funded with an award from the National Network of Libraries of Medicine/Southeastern Atlantic Region.

Description: A team-based approach is being used to build an engaging asynchronous course for citizen scientists (also known as community scientists) who aspire to develop their own projects and investigate health-related issues in their communities. Course development team members bring experience in teaching, design, and citizen science and an external advisory committee contributes additional expertise. The course content breaks down citizen science research methods into understandable components that integrate project planning, ethical and legal issues, strategies for collecting and interpreting data, and communicating results. Instructional methodologies integrate short lectures, animation, interviews, and slides. Practical examples throughout the course will focus on public and environmental health topics geared toward non-researchers interested in developing their own projects. This presentation will also focus on the process, opportunities, and challenges involved in massive open online course (MOOC) development.

Conclusion: The course will come live in summer 2020 and will be promoted to a national audience. The Library team will collect data on the number of people enrolled in the course, the completion rate, and the number of views of individual classes. To assess the curriculum’s effectiveness, evaluation components such as pre- and post- tests, surveys, and quizzes will be embedded in each class. The Library team anticipates that Gearing Up for Discovery will provide participants with a better understanding of the complete research process and make clear the benefits that can be achieved through science.

Poster Number: 30

Time: Friday, July 24, 10:30 a.m.–4:30 p.m.

Getting the Word Out: Outreach in Medical Special Collections

Elisabeth Brander, Rare Book Librarian, Bernard Becker Medical Library, St. Louis, MO

Background: Many institutional medical libraries contain special collections divisions holding rare books and archival materials. While these resources have great cultural and research value, they are often underutilized – students and faculty used to frequenting their university’s main library branch don’t immediately think of medical libraries as a source of material for studies in art history, social history, or other areas of the humanities. This poster highlights ways in which medical special collections can increase awareness of their holdings among the community outside the medical schools they are embedded in.

Description: At the author’s institution, the special collections division has made outreach programming a key part of its mission. Throughout the year, the division hosts a number of activities that include book clubs, open houses, and lectures. These events are advertised on the library website, our social media accounts, and mailing lists; and they are open to the general public. While special collections reading rooms can often be intimidating spaces, our events are meant to be casual with a low barrier of entry, providing visitors with a welcoming introduction to our space.

Conclusion: Our outreach activities have steadily increased in popularity. While some of the events were initially poorly attended, holding them consistently throughout the year allowed awareness to spread through word of mouth. Visitors have included students, staff, and faculty from the medical school, members of the main campus community, and members of the general public.

Poster Number: 31

Time: Friday, July 24, 10:30 a.m.–4:30 p.m.

Graphic Medicine for Emotional Wellness: Therapeutic Library Programming in a Pediatric Behavioral Health Setting

Denise Shereff, AHIP, Instructor II and Coordinator of Fieldwork and Assessment, University of South Florida, Tampa, FL; **Janet Chan**, MLIS student, University of South Florida, Tampa, FL; **Peter Cannon**; **Natalie Taylor**, Assistant Professor MLIS Program Coordinator, School of Information University of South Florida, Tampa, FL; **Brittany M. Baum**, MLIS Student and Graduate Assistant, University of South Florida, Orlando, FL

Background: This poster showcases graphic medicine group programming implemented through a grant with the National Library of Medicine for a mental health literacy program for pediatric patients in an behavioral health center in Florida. The unit provides inpatient treatment for various mental health issues and features a small library established and staffed by faculty and students from the University of South Florida.

Objectives

Participants will:

• recognize emotions represented in graphic novel samples.

• identify graphic medicine resources in the library collection.

• articulate ways health information can be communicated through comics.

• design a graphic medicine comic.

Description: Project team members provide library services in the unit, including group programming, with the aim of improving patients’ mental health literacy skills. In developing group bibliotherapy programming, a search of current initiatives identified graphic medicine as a growing trend. Graphic medicine uses visual narratives to educate about health information, depicting experiences of having, caring for someone else with, or treating an illness. Graphic novels are of high interest throughout all age groups in the unit so this topic showed promise for group programming development. The National Library of Medicine’s Graphic Medicine lesson plan from the traveling exhibit “Graphic Medicine: Ill Conceived & Well-Drawn” proved adaptable for the target audience, cost effective, and up to date. This model and sample resources provided program structure, guidance, and procedures. Activity worksheets were customized for use in the programs.

Conclusion: Post-program discussions with participants focus on usefulness of the collection and program content and delivery for advance mental health literacy objectives. Post-program surveys allow participants to provide self-assessment of accomplishment of program objectives.

Poster Number: 33

Time: Friday, July 24, 10:30 a.m.–4:30 p.m.

Implementing Interactive Technology Using H5P in Library Medical Education

Jason Fetty, AHIP, Reference and Instruction Librarian, Assistant Professor, Touro University Nevada, Henderson, NV

Background: Engaging students is a challenge in library medical education. Working for the Touro University Nevada Library I started using H5P to create interactive content for research guides, in-class sessions, orientations, and in other areas to better connect with students and other library users. This has included quizzes, worksheets, and more and is designed to make learning library skills more hands-on, rewarding, and memorable.

Description: H5P is an online tool designed to build and share interactive content easily. The time to learn H5P and to create content is quick and relatively pain-free. I was able to acquire a license and decided to trial its use in a variety of library services, including instruction and research guide creation. After some initial success, I have expanded its use across different medical and education programs, for both on-ground (in person and asynchronously) and online instruction. It has also proven valuable for orientations and research guide creation.

Conclusion: There are some limitations with H5P, but as a supplement to instruction it has significant potential and has shown promise, with classes becoming more active and online content offering more engagement. The ease of use and ability to reuse and share content should appeal to content creators with limited access to technology and instructional design support.

Poster Number: 34

Time: Friday, July 24, 10:30 a.m.–4:30 p.m.

Information Skills for Health Professionals: Creating a For-Credit Course for Upper-Level Undergraduates and New Grad Students

Jane Kinkus Yatcilla, Associate Professor of Information Studies, Purdue University Libraries & School of Information Studies, West Lafayette, IN

Background: Purdue University Libraries and School of Information Studies (PULSIS) recently began offering for-credit courses under its own course designation "ILS." Librarians were encouraged to propose new courses that fill a gap on campus. In this case, the librarian designed a one-credit, 8-week course for upper level undergraduate students who intend to attend professional school (MD, DO, DVM, DDS, etc.) or graduate school (MPH, Ph.D.) in a health sciences related discipline. The result was a one-credit, eight-week course called "Information Skills for Health Professionals." The concept of evidence-based practice was used as a vehicle for readings, discussions, and assignments throughout the course.

Description: The librarian used principles of backward design to create the curriculum, starting with what the students should be able to do upon completion of the course, and working backward from there to create appropriate course content. The learning outcomes for the course were:

"By the end of this course, students will be able to:

Plan for comprehensive literature searches by clearly defining your information need and mapping search terms to those concepts;

Find information relevant to your information needs using appropriate subject database(s), including the use of keywords or controlled vocabulary, and available search facets;

Evaluate the level of evidence of various research methodologies;

Save, organize, and use materials in multiple formats (journal articles, books, book chapters, web sites) using a citation management system; Discuss what evidence-based practice means to you in the context of your career goals."

Conclusion: The course was taught in fall 2019 and spring 2020, with 5 and 6 students enrolled, respectively. Students' feedback indicated that the course was very relevant to their educational and career plans. Student requests from the first semester led to some adjustments in the curriculum for the second semester, including creation of a LibGuide for the course, spending more time on building a complex database search, finding health-related data sources, and publishers' requirements for submitted manuscripts. The course will continue to be marketed to both upper level undergrads as well as new graduate students in health-related disciplines.

Poster Number: 35

Time: Friday, July 24, 10:30 a.m.–4:30 p.m.

Is the Future in Focus for Evidence-Based Medicine (EBM)? An Observational Study of Entrustable Professional Activities (EPA 7) Instruction in Clinical Clerkships

Catherine Pepper, Associate Professor/Coordinator of Library Field Services, Medical Sciences Library, Texas A&M University, Austin, TX; **Kelly Thormodson**, Associate Dean and Director, Harrell Health Sciences Library Penn State University, Hershey, PA; **Laura Zeigen, AHIP**, Health Sciences Education and Research Librarian, Oregon Health & Science University, Portland, OR; **Andrew S. Hamilton**, Assistant Professor, Oregon Health & Science University, Portland, OR; **Kristine M. Alpi, AHIP**, University Librarian, Oregon Health & Science University, Portland, OR; **Esther E. Carrigan, AHIP**, Professor, Medical Sciences Library, Texas A & M University Medical Sciences Library, College Station, TX

Objectives: The seventh of 13 Entrustable Professional Activities (EPAs) from the American Association of Medical Colleges defines competencies for evidence-based medicine (EBM) (https://www.aamc.org/initiatives/coreepas/). These competencies are typically taught in pre-clerkship curricula. However, little is known about how—or whether—EBM instruction is continued through students’ practice-focused learning in the clinical years. The research question was: Is pre-clerkship EBM instruction reinforced in clinical clerkships?

Methods: The investigator shadowed clerkship faculty, residents, and students on patient rounds and in didactic trainings at a large teaching hospital in Fall 2019 to capture instances of EBM teaching and practice. Demonstrations of EBM instruction, practice, and competency assessment were recorded on a field guide, which was comprised of functions, competencies, and behaviors as described in EPA7. Informal interviews were conducted with medical librarians and clinical faculty, concerning frequency and depth of student exposure to EBM competencies as applied to patient cases, assessment methods, use of UpToDate, and extent of librarian involvement. Aggregated observations of functions, competencies, and behaviors were categorized by EPA7 competency levels, and exemplars identified to highlight how observations corresponded to or conflicted with the interview data.

Results: Observations of clinical teaching were recorded in various activities in several clerkships. Data collection concluded in November 2019. Final data analysis will be completed by March 2020 and results will be reported at the meeting.

Conclusions: Medical students may not possess the expected level of competencies in EBM upon entering residency. Further conclusions and recommendations will be presented at the meeting.

Poster Number: 36

Time: Friday, July 24, 10:30 a.m.–4:30 p.m.

Is There a Relationship Between Having Residency Application Personal Statements Reviewed by the Library's Editing Service and Medical Students' MATCH into Their Preferred Residency Specialty?

Lisa M. Price, Public Services/Research Support Librarian, Health Sciences Library, Rowan University School of Osteopathic Medicine Health Sciences Library, Stratford, NJ; **Jamie L. Pelley**, , Runnemede

Objectives: This study investigates whether there is a relationship between Rowan University School of Osteopathic Medicine's fourth-year medical students' acceptance into their preferred specialty for residency and their submission of the Personal Statement portion of their residency application for review/edit by the Library's editing service. We hope that our results will aid other institutions which may be considering whether to provide a similar service.

Methods: This case-control study reviews Match records for the years 2018 and 2019 (the years for which data was available) and compares the Match rates into the preferred residency specialty of those who submitted their Personal Statements to the Library’s editing service vs those who did not. 98 of the 298 medical students who participated in the Match process for the indicated years had their Personal Statements reviewed by the Library's service. Anonymized data was analyzed using Pearson correlation analysis and a logit model including choice of preferred specialty and graduation year as well as a binary variable indicating whether or not the editing service was used. The dependent variable was a 0-1 binary variable representing whether the student matched into their preferred specialty. To illustrate the relationship, basic correlations between the variables were also reviewed.

Results: The Pearson test revealed a weak positive but statistically insignificant overall correlation (.056, p = .337) between Match into Preferred and Reviewed by Library Service. When subdivided into 15 specialty categories, only one (All Surgery) showed marginal positive correlation at the 10% level (.307, p = .073) for students seeking a surgical specialty. Logit regression results indicate a low increased odds ratio (1.737:1) that students whose Personal Statements are reviewed by the editing service will match into their preferred residency specialty, but it must be noted that the other values (Wald x^2 = 2.070; p = .150) show no statistical significance.

Conclusions: As a standalone factor, there appears to be little correlation between using the editing service and matching into the preferred specialty. According to the 2018 NRMP Program Director Survey, 78% include the Personal Statement among their top five considerations for interview decisions. However, it is only one of many elements in the Match process. In addition, numerous confounding factors beyond the editor’s control exist (including students’ prerogative to reject editorial suggestions). Therefore, we cannot discount the possibility that other factors might mitigate any positive effects of the editing service (e.g., likelihood of interview) in final Match results.

Poster Number: 37

Time: Friday, July 24, 10:30 a.m.–4:30 p.m.

LOR of the Things: Supporting the Sharing and Reuse of Learning Objects through an Open-Source Repository

Molly Knapp, AHIP, Training Development Specialist, University of Utah, NNLM Training Office, Houston, TX; **Zak Risha**, Learning Management System Admin/Developer, National Network of Libraries of Medicine, Pittsburgh, PA; **Remzi Kizilboga**, Instructional Designer, NNLM Training Office, Bloomington, IN; **Rebecca Brown, AHIP**, Training Development Specialist, Spencer S. Eccles Health Sciences Library, Spencer S. Eccles Health Sciences Library, Salt Lake City, UT; **Jessi Van Der Volgen, AHIP**, Assistant Director NNLM Training Office, University of Utah–Salt Lake City

Background: This case study discusses the development, implementation and uptake of an internal learning object repository (LOR) for a large, national organization with a distributed working environment. We will describe the organizational environment and why an internal LOR was needed, as well as technical considerations, including the various document management systems (DMS) available and why we selected the open source SeedDMS to use as an internal LOR. We will weigh the benefits and challenges of implementing a LOR across a distributed working environment, reflect on challenges associated with implementation, and describe future directions.

Description: Due to our distributed working environment, we encountered several obstacles to finding, collecting, and collaborating on learning objects. First, no unified collection of educational materials existed. Second, learners and staff found it difficult to locate the most recent version of learning objects, as most materials were tied to a class registration web page, and could disappear from public view if the web content was unpublished. Finally, no space existed where educators could easily share materials. Locating related learning objects usually involved emailing multiple people. We developed a working list of technical requirements and desired features, researched open source DMS, and customized our chosen software to meet standards for security, interoperability with our learning management system (Moodle), and metadata (Dublin Core). Finally, we produced documentation and internal training to onboard staff. The entire process, from selection to production, took 18 months.

Conclusion: An internal LOR enhances ease of work and quality of resources for users. Staff have constant access to the latest versions of course materials in one location, which supports the agile development of courses by eliminating duplication of effort (re-creating content that already exists), and offers a solution to easily share new content with learners. Key Lessons learned: 1) Organization and user needs matter; 2) Communication, training, and modeling is critical to adoption 3) Working meeting are useful. Since deploying in 2018, our LOR houses over 18 gigabytes of content and adds on average 80 files per month.

Poster Number: 38

Time: Friday, July 24, 10:30 a.m.–4:30 p.m.

Mapping a Library Evidence-Based Medicine Program to the Association of College and Research Libraries (ACRL) Information Literacy Framework

Vedana Vaidhyanathan, Health and Life Sciences Librarian, Baylor University, Waco, TX; **Amy James**

Background: Multiple departments at this institution use versions of Evidence-Based Practice programs with their students from undergraduates to doctoral students. The different departments needed help with training their students. The Health and Life Sciences Librarian has taken on the role of offering multiple workshops on different parts of Evidence-Based Practice and helping with systematic reviews. At the same time the library has taken on incorporating the ACRL Information Literacy Framework into the library's teaching. The Health and Life Sciences Librarian decided to use the framework as guidelines for teaching about Evidence-Based Practice.

Description: The Evidence-Based Practice program began with a five part Saturday morning program that included individual assessments though homework assignments tailored to their papers. The workshops were tailored to graduate students, but could be altered for undergraduates. Later an undergraduate workshop for Honors students was added onto the schedule. The workshops concentrated on three parts of the framework: Information Creation as a Process, Research as Inquiry and Scholarship as Conversation. The students used the workshops to go back and have the deeper conversations about scholarship, searching and finding evidence with their PIs and advisors. Starting in Spring 2020 the program will be supplemented with online assignments and videos to deepen their understanding of the ACRL Information Literacy Framework and Evidence-Based Practice.

Conclusion: We expect to examine how much the students have learned and whether or not they came out of the workshops seeing Evidence-Based scholarship as more than something they just have to tick the boxes for, and instead as a way of thinking and examining the world. We plan to execute the same workshops in an online environment and gather even more information beginning Fall 2020

Poster Number: 39

Time: Friday, July 24, 10:30 a.m.–4:30 p.m.

Marketing Them: A New Elective for Medical and Dental Students

Teri Shiel, Head, Information Desk; **Rich McIntyre, Jr.**, Emerging Technologies Librarian; **Marissa F. Gauthier**, Research & Instructional Services Librarian; UConn Health Sciences Library, Farmington, CT

Background: In the Fall of 2017, librarians at the UConn Health Sciences Library were exploring new instruction opportunities in order to become more involved with the Medical School’s curriculum, make more connections with students and faculty, and to represent the library and librarians in a new way. We developed an elective course on professional topics that students are often expected to know but aren't necessarily taught.

Description: Our planning and ideas became “Marketing You,” a one-week, five-day course that taught students social media management strategies, how to write cover letters and resumes, how to design presentations and posters, and public speaking. Each session was two hours long and covered one professional topic with pre-class readings and media, in-class activities, discussion, and real-life examples. Students gave 10 minute presentations on the final day about what they learned over the week and how they incorporated their newly-learned skills into existing social media accounts, resumes, posters, or presentations.

Conclusion: The course has been taught several times and has always received great reviews and feedback. All students who have taken the post-class assessment rated it as being educationally beneficial, found the course content helpful, and enjoyed working with librarians. We benefited from the experience of honing our teaching skills, developing curricular materials, and building relationships with students and faculty. This course has led to more opportunities for instruction and collaboration with the School of Medicine and increased the Library's visibility on campus. We plan to continue offering Marketing You once every academic year.

Poster Number: 40

Time: Friday, July 24, 10:30 a.m.–4:30 p.m.

Medical Librarians as Key Players on the Interprofessional Education Team

Mariana Lapidus, Reference Coordinator, MCPHS University, Boston, MA; **Samuel B. King**, Manager, Manchester Campus Library, MCPHS University, Manchester, NH; **Irena G. Dryankova-Bond**, Library manager, Massachusetts College of Pharmacy and Health Sciences, Worcester, MA

Objectives: - Using the example of one academic institution, demonstrate the benefits of the effective Interprofessional Education (IPE) collaboration between the multi-campus library, different academic departments, and higher administration.

- Identify the opportunities for better involvement of medical librarians into the variety of IPE academic initiatives and the boundless possibilities for productive partnership between librarians and other faculty in healthcare education.

Methods: This case study highlights the involvement of academic librarians into the following IPE initiatives:

- Representing the Library on the two IPE Institutional Committees.

- Promoting IPE on the institutional level: the IPE designated days on two campuses planned for 2019/20 with active librarian involvement.

- Designing the IPE Research Guide serving as the major information resource for faculty across all disciplines.

- Establishing the IPE web presence: the IPE web page was created as the result of the collaboration between the IPE Committee members, librarians and the Information Services department.

- Participating in research activities: an MCPHS faculty IPE survey was administered in November, 2019. The Boston librarian actively participated in designing the survey instrument by performing extensive literature searches on similar studies.

- Contributing to the development of a virtual IPE rounds project at Manchester.

Results: Librarians at the MCPHS are well involved into a variety of IPE activities on many levels, such as departmental, inter-departmental, multi-campus, and institutional. They provide the research support to other faculty members engaged in IPE activities and create tools and resources in support of the IPE activities on a global (the IPE Research Guide, IPE Web site and Blackboard page) and project-specific (Blackboard site) basis. Librarians are also engaged in campus-specific IPE projects: Manchester-based task force is currently in the process of developing an inter-disciplinary virtual rounds for students which eventually will be expanded to all campuses.

Conclusions: Medical librarians represent an essential workforce behind IPE and are uniquely skilled to contribute to all types of inter-professional activities at their academic institutions. They need to take advantage of multiple opportunities and be proactive in order to get better engaged into IPE. This will allow information professionals to successfully understand and meet the needs of their academic community, provide highly effective outreach services, develop responsive information literacy programs, and more effectively promote library resources. It will also enable librarians to learn from the experiences of other professions, further enriching the services they provide.

Poster Number: 44

Time: Friday, July 24, 10:30 a.m.–4:30 p.m.

Preserving and Honoring the Legacy of World War II Medical Efforts

Gabrielle Barr, Archivist, NIH, Washington, DC; **Corinne Holden Miller**, Clinical Informationist, Northwestern University, Chicago, IL; **Annie B. Wescott**, Research Librarian, Northwestern University, Feinberg School of Medicine, Chicago, IL; **Mary Anne Zmaczynski**, Public Services Librarian, Galter Health Sciences Library, Northwestern University, Chicago, IL; **Ramune K. Kubilius, AHIP**, Col Dev/Spec Proj Librarian, Galter Health Sciences Library & Learning Center, Northwestern University Feinberg School of Medicine, Evanston, IL; **Abebi Espinoza**, Special Collections Assistant, Galter Health Sciences Library and Learning Center, Chicago, IL; **Katie Lattal**, Special Collections Librarian, Galter Health Sciences Library & Learning Center, Chicago, IL; **Karen Gutzman**, Head, Research Assessment and Communications Department, Galter Health Sciences Library, Northwestern University, Chicago, IL

Background: James A. Conner and Michael L. Mason, who served in the 12th General Hospital Unit during World War II, donated their papers to the library, which were processed and digitized. The library also created an interactive digital exhibit that narrated the story of the 12th General Hospital Unit and provided the broader context through images, videos, and documents from other repositories. To draw attention to resources that promote the legacy of this prestigious medical corps, a team comprised of individuals from different departments of the library embarked upon a multi-faceted outreach mission.

Description: Over the course of three months, we publicized the exhibit through social media and digital signage, and invited a military medicine expert to speak at the inauguration event for the exhibit. We performed genealogical research to find relatives of those who served and invited them to attend and participate in the inaugural event. Members of the team called the descendants that could be located and wrote them personalized notes on stationery with photographs from the collection and a link to the digital exhibit. The team also created a five-panel traveling banner display to be unveiled at the lunchtime speaker event. Following the speaker event, the library had an open house for visitors to see the 12th General Hospital materials. The event was covered by the campus media as well as by the Chicago Tribune and NPR.

Conclusion: With more than 150 attendees from across the country at the speaker event, the display of the traveling banner exhibit at locations in the Chicago area, and the increase of remote and in-person reference requests about the 12th General Hospital, the outreach activities the library developed during the summer of 2019 proved to be successful. The library has since used some of these tactics like the traveling banner exhibit to highlight other special collections projects and has most importantly learned several lessons on how to manage a large-scale outreach campaign.

Poster Number: 46

Time: Friday, July 24, 10:30 a.m.–4:30 p.m.

Reinvent Medical Reference Librarians' Role in the Systematic Review Process of International Graduate Students

Chih-Wen Chou, Medical Librarian, National Cheng Kung University Medical Library/ Department and Graduate Institute of Library and Information Science, National Taiwan University, Tainan, Tainan, Taiwan (Republic of China); **Ching-Ju Fang**, Medical Librarian, National Cheng Kung University Medical Library / Department of Secretariat, National Cheng Kung University Hospital, College of Medicine, National Cheng Kung University; **Szu-chia Lo**, Associate Professor, National Taiwan University, Taipei, Taipei, Taiwan (Republic of China)

Background: Systematic review (SR) is the highest level of research design in Evidence-based Medicine (EBM) that attempts to synthesize all the available evidence with explicit and transparent research methods and brings the available evidence to a research question. Nowadays, Ph.D. students in medical sciences are usually required to undergo the process of developing searching and writing skills for SR studies to be included in dissertations. To meet the high demand from the medical students, the medical library developed thorough step-by-step SR courses to guide medical students through the SR process, which are very unique library programs in Taiwan.

Description: The medical library starts to provide SR courses and follow-up consulting sessions since 2016. Our medical library is the first one that initiates the English-taught SR courses for international students in Taiwan. The program is designed based on the Cochrane Handbook for Systematic Reviews of Interventions and PRISMA Statement. The two-session course is developed to first introduce SR studies and the application of all-in-one documentation form. The second session introduces the use of EndNote to manage literature by removing duplicates and creating groups for review authors to reach a consensus according to the study selection process of PRISMA Flow Diagram.

The courses have been evaluated and redesigned by reviewing students' documentation forms in class and the interaction in consulting sessions. The poster illustrates the overview of international students' research lifecycle, implementation of the program, and the impact of SR courses.

Conclusion: Eight international students were interviewed in a pilot study to reveal the research lifecycle and their recognitions toward the SR courses. According to the students' statements, the courses are instrumental for those who receive scholarships within a limited time frame. The pre and post-test surveys were administered to investigate students' understanding of SR, searching skills, and application of EndNote. The findings show that the courses help the students acquire the knowledge and skills for independent study at an early stage of the research lifecycle and establish the essential knowledge of EBM that paves the way for conducting future researches.

Poster Number: 47

Time: Friday, July 24, 10:30 a.m.–4:30 p.m.

Research Preferences from Athletic Training Students: Academic Librarians Survey from Two Universities

Lisa A. Adriani, AHIP, Research and Instruction Librarian, Edward and Barbara Netter Library, Quinnipiac University, Shelton, CT; **Daniel G. Kipnis**, Life Science Librarian, Campbell Library, Rowan University, Glassboro, NJ; **Ronda Kolbin**, Public Services Librarian, Quinnipiac University, Guinnipiac University, Hamden, CT

Background: Currently, there is very little in the peer-reviewed published literature on how athletic training students conduct research and their exposure to information literacy instruction and libraries. With approximately 393 universities offering athletic training program, understanding these students will help librarians who wish to collaborate and offer information literacy instruction. Our goal as academic librarians is to introduce literature to help address working with athletic training students.

Description: Our poster will share survey results from two east coast universities with accredited athletic training programs. The responses will include answers from 72 students from the following research questions:

1. If a class was offered on how to successfully search literature/research databases, when is your preferred time during the semester?

2. How long will you surf a website or search a database before asking for help?

3. When seeking research assistance, where do you turn first?

4. Which method of communication do you prefer when seeking assistance from the library?

Conclusion: Librarians need to: Get in the classroom in the beginning of the semester, market their availability to help with research, and promote their availability to "meet" the students with a variety of communication platforms.

Poster Number: 48

Time: Friday, July 24, 10:30 a.m.–4:30 p.m.

Restructuring a Baccalaureate Nursing Research Course: A Librarian-Nurse Quality Improvement Project

Rachel Charlotte Lerner, AHIP, Reference & Instruction Librarian, Quinnipiac University, Hamden, CT; **Susan Kosman**, Assistant Professor, Quinnipiac University, Hamden , CT

Background: Undergraduate nursing curricular requirements include the ability to retrieve, evaluate, and synthesize evidence and apply it to practice. At this institution, the research course was a didactic lecture focused on research methodologies. This quality improvement project paired a librarian and a nursing faculty member to shift the focus of the senior research course from a traditional theory and research methodology approach to an increased focus on the practical application of evidence-based practice.

Description: The primary burden of the course redesign lay on the nursing faculty member. Class activities and associated literature searching became a shared project between the librarian and the faculty member. Prior to the class, a literature search was conducted to determine how to engage students in research courses. The faculty member mapped out the course topical outline. Focus shifted from traditional research methods and theory to a practical approach on the evaluation, application, and dissemination of evidence aligned to baccalaureate essentials. Librarian and faculty created class activities to further engage the students with the new content. These included guest lectures from nursing researchers and practitioners, an in-class interactive evidence retrieval contest, critical appraisal worksheets, and two group projects: critical appraisal presentation of filtered evidence sites, e.g. ECRI Guidelines Trust, and poster presentations exploring a student-generated PICO question.

Conclusion: The QI project was evaluated through an anonymous pre/post questionnaire that included a Likert scale on attitudes towards evidence-based practice. The post questionnaire included a free-text area asking, “In what ways do you anticipate incorporating EBP principles into your future studies or career?” The questions showed universally improved scores, and the free-text field revealed an understanding of the integral connection of EBP to the delivery of safe clinical care and professional responsibility. Given that this course is offered multiple times throughout the year, the insights gained from this group will be applied and evaluated once again with the next cohort.

Poster Number: 49

Time: Friday, July 24, 10:30 a.m.–4:30 p.m.

Stop, Collaborate, and Listen: Exploring the Gameful Side of Nursing Education

Nena Schvaneveldt, AHIP, Education Librarian, University of Utah–Salt Lake City; **Brandon Patterson**, Technology Engagement Librarian, University of Utah–Salt Lake City; **Ann Butt**, Assistant Professor, Clinical, University of Utah College of Nursing, Salt Lake City; **Clare Kranz**, Assistant Professor, University of Utah College of Nursing; **Jennifer Macali**, Assistant Professor

Background: Undergraduate nursing students learn many topics they may not find immediately applicable, such as identifying databases to search, quality improvement, and citation. To increase student engagement with the aim of improving learning, librarians at the Spencer S. Eccles Health Sciences Library partnered with subject faculty in the College of Nursing to develop and implement gameful learning activities for these topics.

Description: Based on research and peer colleagues' conference presentations, librarians implemented a rapid-fire searching game to teach the differences between databases, and a gameful activity using Legos to teach about plagiarism and citation. Meanwhile, librarians also partnered with nursing faculty to assist in designing, implementing, and evaluating a timed game to simulate the quality improvement process. In this activity, students assemble Mr. Potato Head toys as a team, making iterative changes and logging data to document improvements. The Mr. Potato Head Game was studied by a pre- and post- test followed by focus groups, while librarians collected informal assessment data on the librarian-led games.

Conclusion: Using games to introduce difficult concepts in nursing education has been a success. Students have had positive reactions to learning databases through the game, and have come to a deeper understanding of the dangers of plagiarism through the Lego activity. Because of extensive data collection, the Mr. Potato Head game has proved most successful in reaching student learning outcomes, showing that students' motivation and aptitude to enact quality improvement change have increased significantly. Based on this early feedback, librarians and nursing faculty are seeking to develop more gameful learning throughout the curriculum, as well as throughout other health sciences disciplines.

Poster Number: 50

Time: Friday, July 24, 10:30 a.m.–4:30 p.m.

Supporting Publishing Literacy: A Four-Step Worksheet and Accompanying Case Study

Claire O. Sharifi, Reference Librarian, Liaison to School of Nursing & Health Professions, Gleeson Library, University of San Francisco, San Francisco, CA

Background: This poster reports on the creation of a four-step worksheet and accompanying case study that introduce graduate students, doctoral students, and other novice authors to publishing concepts and common practices. The publishing ecosystem can be overwhelming and opaque, and many struggle with understanding publishing processes, copyright transfer, and publishing models.

Description: Written for an audience of nurses, but applicable to any of the health sciences, the four-step worksheet presents a structured process for identifying an appropriate journal for publication, as this can be a particularly challenging activity for students and novice authors. The worksheet walks users through the entire submission process, from generating a list of appropriate journal titles, to reviewing and understanding a journal’s author guidelines. The case study provides a clear, plain language introduction to publishing concepts and practices, which are not always explicitly addressed in academic programs.

Conclusion: The worksheet and case study, products of an iterative design process and a collaboration between a librarian and School of Nursing faculty member, have been adopted by multiple doctoral level nursing courses to aid students in submitting manuscripts. Evaluation is in progress, and outcomes include an improved ability to identify an appropriate journal for a manuscript, and an improved ability to evaluate and understand academic health sciences publishing.

Poster Number: 51

Time: Friday, July 24, 10:30 a.m.–4:30 p.m.

The Future of Health Literacy in Focus across the State

Alexandria C. Quesenberry, Research and Learning Services Librarian, University of Tennessee–Knoxville; **Kelsey Grabeel, AHIP**, Assistant Director, University of Tennessee–Knoxville; **Leah Cordova**, Research and Learning Services Librarian, University of Tennessee Health Science Center, Memphis, TN

Background: Medical librarians can have numerous responsibilities related to health literacy, including educating future medical professionals throughout the duration of their professional training. As other large, multi-site institutions have experienced, providing consistent training across multiple campus can be challenging. In order to address this challenge, librarians at the University of Tennessee Graduate School of Medicine (UTGSM), located in East Tennessee, and the University of Tennessee Health Science Center (UTHSC), located in West Tennessee, collaborated together to create an asynchronous online module for health sciences students and residents that addresses the importance of health literacy and bearings on their future practice.

Description: Given the different geographical locations and educational programs, librarians collaborated to create an online module introducing health literacy that could meet the unique needs of each campus. Content included a definition of health literacy, discussion of the impacts of health literacy on health and healthcare at the national, regional, and local levels, and identification of patient education strategies that can be used in patient care. A student self-assessment was built into the module consisting of a multiple-choice pre-/post-test, as well as an open-ended reflection question upon completion. To ensure compatibility with LibGuides and Learning Management Systems, the workshop is hosted in Mediasite and accessible to anyone with a university login. The workshop was finalized and placed online during October’s Health Literacy Month, alongside marketing via social media and relevant campus listservs.

Conclusion: Although at two different campuses across the state, this program allowed for librarians to collaborate on something relevant to all, health literacy. Plans are in place to update the online workshop to include more detailed health literacy topics and interactive elements in conjunction with Health Literacy Month.

Poster Number: 52

Time: Friday, July 24, 10:30 a.m.–4:30 p.m.

The University Libraries Open and Affordable Textbook (OAT) Program Impact on Health Sciences Programs

Matthew C. Bridgeman, Information and Education Librarian, Robert Wood Johnson Library of Health Sciences, New Brunswick, NJ

Background: This poster will discuss the library’s central role in the conception of the XXX Open and Affordable textbook (OAT) program and its success through the lens of XXX Biomedical and Health Sciences. The rising costs of textbooks and other education materials is a major issue facing students and faculty today. The goal of the OAT program is to incentivize faculty to create courses that use open and affordable resources. Since 2015 the program has helped establish courses in various departments such as Psychiatry and Physical Therapy.

Description: The Open and Affordable textbook program is run by the XXX University Libraries with a team composed of a representative from each campus. It is an incentive program where professors, instructors, lecturers apply to receive a research fund. They have to provide an actionable plan where their course must use open materials or have substantially reduced the cost of education resources. After each course we send out a faculty survey and a student survey to receive feedback on the program’s effectiveness and implementation. There have been multiple applicants from the XXX Biomedical and Health Sciences Campus where professors have adopted open materials, used library resources, or even created their own.

Conclusion: The program is on-going and successful. So far the Psychiatry Department has used self-developed materials for their clerkship. In Physical Therapy a professor has created their own ibook textbook for development Across the Lifespan course. A First Year medical School professor has created an anatomy course using solely library resources. These are just a few examples. To date, the program has impacted 724 students and saved $112,153 in textbook costs in XXX Biomedical and Health Sciences. Across the entire University the savings amount to over $3.5 million.

Poster Number: 55

Time: Friday, July 24, 10:30 a.m.–4:30 p.m.

Using Interactive Guides in the Health Sciences: Teaching Online Was Never Easier

Yvonne Mery, Librarian and Co-founder, Sidecar Learning, Tucson, AZ

Background: In this poster session, which will also include a hands-on demonstration with a laptop, participants will learn about a new tool, Sidecar Learning, (https://www.sidecarlearning.com) that allows librarians to create interactive guides in minutes. The tool was developed by two University of Arizona librarians and has been used to reach thousands of students. Attendees will learn about how Sidecar Learning was used to create engaging microlearning interactive step-by-step guides for PubMed. Attendees will also learn about why and how two librarians made the leap from librarian to entrepreneur.

Description: Librarians need a tool that to teach students how to use complicated library databases online so that they can reach more students anywhere, anytime. Videos, although a great medium, are passive and are not easy to create nor edit. Many other solutions either require programming expertise or are pedagogically lacking. We used Sidecar Learning to create in-app guides for students in medicine, pharmacy, and nursing with great success. Attendees will lean how we created the interactive guides and what students and instructors thought of them. Attendees will also have a chance to create a guide on the spot and/or act as a student and learn with a guide.

Conclusion: We have reached thousands of students with these guides. We will present usage data, student comments (anonymized), and other successes. This poster will present both this program information and information on the process of moving from librarianship to entrepreneurship with the help of the presenters' home library, the business college, and the tech transfer office at their university.

Poster Number: 56

Time: Friday, July 24, 10:30 a.m.–4:30 p.m.

Using LibGuides to Improve Information Literacy in a Dental School Curriculum

Holly Thompson, Information Services Librarian, USC, Los Angeles, CA; **Jessica Kim**, Student Worker, Wilson Dental Library

Objectives: Librarians wanted to determine whether LibGuides developed for first-year dental students in their first trimester increased the use of authoritative resources in their assignments. These guides are intended to help students transition to the unfamiliar problem-based learning (PBL) curriculum and expectations for individual learning.

Methods: In 2019, librarians developed LibGuides for each PBL case administered to first-trimester dental students. Dental faculty and librarians identified high-quality resources that addressed learning needs for each case (e.g. information about dental anxiety). Librarians added links to books (print and online), databases, trusted websites, and institution-specific resources into each guide. Guide-specific surveys were distributed after each case closed, as well as a final survey at the end of the trimester to solicit general feedback about the LibGuides. Librarians collected usage statistics through the platform’s internal tracker and gauged faculty support through informal feedback.

Results: There are 144 first-year dental students, and the guide for the first case received over 2500 views. The number of views per guide depended on the case’s length, but there was a general decline as the trimester progressed. Students reported that the suggested keywords and textbooks have been the most useful elements of the guides. The quality of cited references in 2019 show improvement when compared to assignments from 2018, which was before LibGuides were implemented. Feedback from faculty has been positive. Reference interactions for PBL-related questions has also declined since the deployment of these guides.

Conclusions: PBL case-specific LibGuides support students in their first trimester by offering structure, guidance for appropriate resources, and anytime access. As evidenced by the number of views and quality of cited references, we believe students access these guides and appreciate the suggestions made. Further research is needed to determine any statistically significant improvements to information literacy.

Poster Number: 57

Time: Friday, July 24, 10:30 a.m.–4:30 p.m.

Using the Human Library® to Provide Diversity and Inclusion Education for Current and Future Health Care Professionals

Natalie Hutchinson, Librarian and Associate Professor; **Jill Edgerton**, Collections Librarian and Assistant Professor; Des Moines University, Des Moines, IA

Background: The ability to work empathetically with a diverse array of patients and colleagues is crucial to quality health care and the success of future health care professionals. To support diversity education, a core value at our institution, we led an interdepartmental group in putting on our institution’s first ever Human Library® event. The Human Library® is a licensable event format that facilitates one-on-one conversations between community members (“Readers”) and volunteers (“Books”). Books share experiences with challenges due to their identity or life experience, speaking openly and honestly with the Readers who have “checked them out” for a twenty-minute conversation.

Description: Representatives from the Student Counseling Center, Student Life, Marketing, Community Relations, and the Office of Multicultural Affairs joined library staff in planning and executing the event. The team met weekly, biweekly, or monthly depending on need, and communicated via email otherwise. Responsibilities were divided between team members based on area of expertise and interest. These responsibilities included recruitment, selection and training of Books, space selection and setup, hospitality for Books and Readers, precautionary security and aftercare provisions, advertising, and management of the “checkout” process. Post event, the team met to review evaluations, assess the event, and note any adjustments for future events. Books were recruited from our campus community and via community partners and titles included “Person of Size”, “Teen Mom”, “Just a Nurse”, “Bi-Muslim” and “Disability Pride.” Readers included university staff, faculty and students across multiple programs.

Conclusion: Licensed materials provided included evaluations for Books, Readers, and event planners. In these post-event evaluations and in informal conversations Readers, Books, and planners reported excellent experiences, and all responses were very positive about the event overall. After review of the event, the team has decided to host another sometime in the upcoming year. We are also considering developing an IPE event around the Human Library®, as well as considering ways in which we can involve the wider metro area community.

Poster Number: 58

Time: Friday, July 24, 10:30 a.m.–4:30 p.m.

Utilization of Brief Screencast Videos to Assess Student Knowledge and to Provide Assignment Feedback

Emily P. Jones, AHIP, Health Sciences Librarian, University of North Carolina at Chapel Hill (UNC), Chapel Hill, NC; **Christopher Wisniewski**

Background: In a flipped, required first-year drug information course, students were taught the systematic approach to answering drug information (DI) questions, commonly utilized DI resources, and literature searching. The librarian taught three weeks of the course focused on mobile applications, development of literature searching skills, and practicing in PubMed and Scopus®. Course assignments were redesigned in 2019 based on assessment best practices and replaced weekly multiple-choice quizzes used in prior iterations of the course. The purpose of this assignment was to assess student literature searching skills in a practical and innovative manner.

Description: Following two weeks of literature searching instruction, students were assigned a DI question that would serve as the impetus for the search they conducted. Students (n = 66) had one week to practice and record a screencast video of their search in PubMed utilizing an institutional subscription to Panopto™, an online video platform. Students narrated their video with an explanation of the actions being performed and were assessed using a 20-point rubric created by the course coordinator and librarian. The librarian also created general feedback videos for each question utilizing Panopto™. Videos depicted the librarian performing searches and clarifying troublesome aspects for students. Feedback videos were available to students via the learning management system after grades were released. The librarian spent about 24 hours grading and six hours writing scripts, recording, and editing feedback videos.

Conclusion: Screencast videos proved an innovative way to assess student knowledge and to provide feedback on literature searching assignments. Most students performed well on the assignment (mean score = 17.35 points) and few experienced technical difficulties. Instructors will continue to use this method for assignment and feedback in the future, with minor changes to the rubric planned before the next course offering to decrease student confusion.

Poster Number: 59

Time: Friday, July 24, 10:30 a.m.–4:30 p.m.

Valued Information Resources When Making Diagnoses: The Experiences of Medical Students in a Clinical Reasoning Course

Jonathan Eldredge, AHIP, Associate Professor, Health Sciences Library and Informatics Center/Family & Community Medicine, HSLIC, University of New Mexico, Albuquerque, NM; **Laura Hall**, Division Head, Resources, Archives and Discovery, Health Sciences Library and Informatics, The University of New Mexico Health Sciences Library and Informatics Center, Albuquerque, NM; **Sumit J. Patel**

Objectives: The clinical reasoning process aimed at making an accurate diagnosis represents a complex activity. No studies to date have reported on how medical students harness existing information resources in their clinical reasoning courses to arrive at correct diagnoses.

Methods: Retrospective cohort study. An anonymous survey asked students to recall their most useful information resources halfway through their three clinical reasoning courses. Students then were asked to evaluate the comparative usefulness of these selected resources. Finally, students reported on those resources that they no longer found useful. This survey sought to learn what information resources medical students value so that librarians can provide the best possible instructional and collection resources.

Results: 69 of the 107 students enrolled in the Clinical Reasoning 2 course completed the three-part survey (64.5% response rate). Students frequently mentioned finding point-of-care tools, general web browsers, journal articles, course materials, and textbooks to be useful. When forced to rank their preferences students added medical society websites and the diagnostic tool Diagnosaurus in AccessMedicine. Student halfway through their three courses no longer found medical licensure exam review guides, patient-oriented sources, or course lecture notes to be helpful.

Conclusions: Medical students halfway their three clinical reasoning courses provided surprising results as well as some expected results in this retrospective survey.

Poster Number: 60

Time: Friday, July 24, 10:30 a.m.–4:30 p.m.

Vision of Increased Vigilance for Predatory Publishing: Focus on Educating Future Authors and Users of the Literature

Dana Gerberi, AHIP, Librarian, Mayo Clinic, Rochester, MN; **Cynthia Beeler, AHIP**, Academic Medical Librarian IV, Mayo Clinic Libraries, Rochester, MN; **Julie M. Taylor**, Outreach Librarian, Mayo Clinic, Rochester, MN

Objectives: This investigation assessed first-year internal medicine resident familiarity with the concept of predatory publishing and associated pitfalls at an academic medical center in the Midwest. While much has been written about predatory publishing, librarians questioned the level of awareness among early career professionals who are eager to publish and who also make patient care decisions influenced by various online resources.

Methods: Health sciences librarians involved with teaching PubMed searching for PICO-structured clinical questions began incorporating instructional content on the topic of predatory publishing into monthly rotating small group sessions with first-year internal medicine residents in July 2019. It was unknown whether such education was provided elsewhere. Content covered the nature of the problem and warning signs to help detect questionable journal sources. In order to gauge the knowledge of this learner population, a brief 5-question survey was conducted during each session. Residents were asked if they were aware of predatory journals prior to the session and, if so, whether they understood the ramifications of using and publishing in predatory publications. The survey also tested for general knowledge on open access, inclusion of predatory sources in scholarly databases and the need for scrutinizing journal sources when critically appraising the literature.

Results: A total of 30 residents participated in the training sessions and completed surveys at the time of reporting. Ten residents, or a third of the sample, were previously unaware with the concept of predatory publishers and journals. Of those who indicated some level of prior awareness, more than half (60%) responded as not having realized the consequences of citing and publishing in predatory journals. As a learning outcome, all of the participants acknowledged that articles from predatory journals may be found in scholarly databases as well as recognized the necessity to examine journal sources during the critical appraisal process.

Conclusions: Early results significantly support that ongoing health professional education about predatory publishing is imperative not only before the journal selection process when seeking manuscript publication but also when turning to the literature for evidence-based guidance on patient care. Invalidated articles from predatory journals could potentially misinform patient care decisions causing more harm than good. A limitation of this investigation is the small sample size. Suggestions for further research might involve additional educational assessment during training sessions and follow up with participants to determine how effectively the information learned is applied in real-life situations.

Poster Number: 61

Time: Friday, July 24, 10:30 a.m.–4:30 p.m.

What Did the Doctor Say? A Health Literacy Workshop Helps Health Care Providers Improve Patient Communication

Lauren Amber Wheeler, Information Services Librarian, University of Maryland, Baltimore, Baltimore, MD; **Mary Ann Williams**, Research, Education & Outreach Librarian, Health Sciences & Human Services Library, University of Maryland, Health Sciences & Human Services Library, Baltimore, MD

Background: Health sciences librarians at our institution monitored driving forces in healthcare putting an emphasis on health literacy. The IOM report and Joint Commission view health literacy as a needed competency for patient care. The librarians were aware of efforts encouraging patients to advocate for their health literacy; however, education for current and future healthcare providers was lacking. The librarians saw this as an opportunity to educate and expose healthcare providers to tools and resources to help providers communicate effectively with their patients. The librarians hope to teach healthcare providers how health literacy is an important component of enhancing patient outcomes.

Description: The Enhancing Patient Outcomes through Clear Health Communication workshop is a redesign of a previous library workshop about patient communication. The current workshop hopes to assist healthcare providers in identifying factors to low health literacy, utilize best practices for clear communication, and understand the importance of health literacy in patient care outcomes, patient safety, and research participation. By scaffolding learning through active participation and activities, librarians are able to achieve the learning objectives for the workshop. In their evaluations, participants state they feel empowered in their new communication techniques. The workshop has proven to increase campus awareness of librarians as health literacy advocates, partners, and experts.

Conclusion: Positive feedback from attendees endorses one of the goals within the library’s strategic plan to “engage health care providers and researchers to improve clear communications and increase health literacy awareness”. The librarians continue to solicit feedback from workshop participants and look for professional development opportunities to stay current with participant needs as well as the future directions of health literacy.

Poster Number: 62

Time: Friday, July 24, 10:30 a.m.–4:30 p.m.

Association of Vision Science Librarians (AVSL): Fifty Years of Collaboration

C. Brooke Caldwell, Electronic Resources Librarian, Southern College of Optometry, Memphis, TN; **Karen Sue Alcorn**, Reference and Instruction Librarian, MCPHS University, Worcester, MA; **Gale A. Oren, AHIP**, Librarian, Kellogg Eye Center, Kellogg Eye Center, University of Michigan, Ann Arbor, MI

Background: To demonstrate how the Association of Vision Science Librarians (AVSL), an international group of libraries and individuals in over 20 countries, has collaborated in various projects to advance the field of vision science librarianship and promote the scholarship of patrons in the fields of ophthalmology, optometry, and vision science. AVSL also offers support to its members on best practices, general assistance, and patron inquires. This poster is a user review of four collaborative projects by the AVSL over the past 50 years.

Description: A review of previous and ongoing collaborative projects show how vision science librarians across international borders are advancing the field of vision science librarianship. The authors selected four projects they considered the most important for review by its members: A Union List, a Standards of Vision Science Libraries document, the Opening Day List, and a Whitelist of Vision Science Journals. A survey was posted on the AVSL list-serve for members to review the selected projects on a five-star rating system for: field advancement, efficiency, and usefulness. Members were also asked if they were aware of the project, had used the project, and if they had recommended the project. Basic non-identifiable demographic information was also collected.

Conclusion: This survey of on-going AVSL projects shows that its members are generally pleased with these resources. Despite the high ratings, many members are not recommending these resources to others. The Whitelist ranked the highest of all the projects while the Standards document was the lowest ranked. Members were more aware of the Whitelist and Opening Day List but have used the latter more. An open dialog is needed to better understand why these resources are not being shared with others and how these selected projects can improve.

Poster Number: 63

Time: Friday, July 24, 10:30 a.m.–4:30 p.m.

Envisioning Future Partnerships: Assessing the Health Information Needs of Public Libraries

Leah Cordova, Research and Learning Services Librarian, University of Tennessee Health Science Center, Memphis, TN; **Alexandria C. Quesenberry**, Research and Learning Services Librarian, University of Tennessee–Knoxville; **Hilary M. Jasmin**, Research and Learning Services Librarian, University of Tennessee Health Science Center, Memphis, TN

Objectives: Seen as community-centered institutions, public libraries play an important role in improving the health and well-being of their communities by providing equitable access to health-related information. This project aimed to examine the perceived role and current practices of inner-city public librarians in delivering health-related information to their communities, and opportunities for future partnerships between public and academic health sciences libraries.

Methods: An online survey was created using Qualtrics and distributed to branch libraries within a single library system. This system is comprised of eighteen branch libraries, each serving diverse socioeconomic and demographic populations. The survey consisted of eighteen multiple choice and open-ended questions centered around four domains: demographics, perception of the role of public libraries in proving health-related information, current practices in providing health-related information, and perceived need for training to better support patrons with health information access. Both quantitative and qualitative assessments were conducted from the responses.

Results: 64 completed survey responses were collected from thirteen of the eighteen branch libraries surveyed. Understanding medical jargon, communicating with patrons, and limited availability of print resources were noted as being the top challenges in providing health-related information to patrons. When asked for health-related information, the majority of respondents reported consulting books from the library catalog as their go-to resources. 96% of respondents indicated that library employees need training on health literacy and ways to effectively provide health-related information to patrons.

Conclusions: Survey results indicated that many library workers believe public libraries play a significant role in providing equitable access to health-related information to patrons. However, many respondents reported a need or want for additional training regarding available resources for finding adequate health-related information online. This study shows there is opportunity for health sciences libraries to partner with public libraries on programming and training needs in order to better support the needs of our communities.

Poster Number: 64

Time: Friday, July 24, 10:30 a.m.–4:30 p.m.

Freedom to Make Informed Decisions: Presenting Consumer Health Information to the Incarcerated

Mary-Kate Finnegan, AHIP, Physical Sciences Librarian, Sacramento State University, Sacramento, CA; **Brenda Carrillo**, Director of Collection Strategies and Discovery Services, University of the Pacific Library, Stockton, CA

Background: A local correctional facility approached our academic library to ask if we could help with their First Annual Health Fair which was organized and created by the inmates and the Peacemakers’ Alliance Program. The Health Fair was created to form a comfortable and informative environment in which anyone could ask questions. What they were seeking was print consumer health information for specific health concerns listed by the population. This specific population has limited access to any resources outside of their environment and wanted to be better informed of potential, current, and future health issues affecting them and their loved ones.

Description: Limited on time, we decided to focus on a small number of health topics to maximize reach. We asked for the top five health requests and printed high quality flyers of consumer health information in English and Spanish from MedlinePlus, the CDC, and other resources. Realizing that this meant many health topics would not be addressed we collaborated with contacts at the National Library of Medicine, American Heart Association, The American Lung Association, and the Alzheimer’s Foundation of American and created double-sided handouts in English and Spanish listing health topics. These four associations agreed to provide health information at the appropriate level free upon written request. At the fair we presented the information in four sessions by first discussing a few health scenarios and further questions they might ask the above organizations to best meet their information needs.

Conclusion: The handouts were very popular and some attendees from each information session expressed increased confidence in communicating their health information needs. The information sheets on the top five topics; heart health, men's health, diabetes, high blood pressure, and eating well in a limited environment, addressed some concerns and the inmates were enthusiastic to be given resources for further print health information. This is hoped to be the first of many health fairs organized by the population of the facility and we are invited back to participate in 2020.

Poster Number: 66

Time: Friday, July 24, 10:30 a.m.–4:30 p.m.

Open Public Health Data Discovery: Start with re3data.org

Charles J. Greenberg, AHIP, Library Director, Rowan University School of Osteopathic Medicine Library, Stratford, NJ; **Sangeeta Narang**, Librarian (Selection Grade), Institution, All India Institute of Medical Sciences, New Delhi, Delhi, India

Objectives: Open public health data consists of organized online data repositories assembled during or after public health investigations. Organized public health repository data emerges in a variety of academic, government, or non-commercial discipline or subject repositories. A new registry of research data repositories, re3data.org, has the potential to improve the identification, access, and reuse of public health data.

Methods: The authors examined epidemiology, public health, health services, and social medicine data repositories tagged for public health subjects in re3data.org (n=124) for country of origin, sponsorship, features, data access, and description. Re3data.org provides searching of keywords and browsing of subject tags, and these are tags, and most repositories have multiple subject tags. A visual or linear text browse is available for users. For unclear reasons, the top level re3data.org subject browsing categories separated epidemiology from the main public health browsing category. The observations of the identified public health repositories were recorded with a shared Google Sheet, in order to facilitate and conduct observations of patterns and comparisons.

Results: The authors have created original charts and tables as educational elements, as well as provide visual instruction for how to search re3data.org for public health data repositories. re3data.org currently indexes over 2000 repositories, representing all subjects. In the combined categories public health, health services research, social medicine and epidemiology, medical biometry, and medical informatics, at last count, there are 167 listed repositories.

Conclusions: Availability and sharing of research data can model disease transmission, track health outcomes and assist the allocation of funds for all aspects of public health. The re3data.org registry of data repositories provides visibility for existing and emerging open datasets available for reproducing existing research or provoking new hypotheses.

Poster Number: 68

Time: Friday, July 24, 10:30 a.m.–4:30 p.m.

A Vision of a Post-MARC Future: Large-Scale Experimental Mapping of MARC Data to an XML Schema

Alexander DelPriore, Metadata Transformation Librarian, Lane Medical Library, Stanford University School of Medicine, Stanford, CA; **Dick R. Miller**, Special Projects Librarian, Director for Resource Management Emeritus, Stanford University, Lane Medical Library, Stanford, Redwood City, CA; **Thea Suzanne Allen**, Director of Resource Management, Stanford University, Lane Medical Library, Stanford University School of Medicine, Stanford, CA

Background: While the eventual obsolescence of the MARC format and the value of Linked Data principles for library metadata appear increasingly clear, a lack of conformity persists among the data schemas libraries have adopted to this end, particularly within special libraries. To assess the potential of an experimental, general-purpose, entity-relationship-based bibliographic metadata model, Lane Medical Library mapped over 1 million records (all MARC catalog data) to the XOBIS XML metadata format. Pairing this with a web-facing front-end to serve, browse, and query the transformed data will make it highly amenable to search engine indexing, data visualization and exploration, and future linked-data markup and conversion.

Description: With conceptual documentation on the schema as a starting point, our team of metadata librarians developed a complete mapping of MARC fields and subfields in catalog use to schema specifications, reflecting a core set of entities and relationships between them. Progress was tracked with weekly meetings and a master project spreadsheet. This mapping was implemented as a Python package, chosen to allow for flexible scripting and testing during development. An API to the transformed data, expressed in XML, was then developed using the Flask web framework, using XSLT for conversion to static HTML and allowing for static URLs for every record and queryable data view. Finally, this software was deployed to Lane's cloud platform, exposing the API to web traffic and indexing, and running regularly scheduled transform jobs, mirroring modified MARC to the new format.

Conclusion: Libraries are uniquely long-term institutions, often holding a wealth of interrelated descriptive metadata accumulated across decades. Medical library catalogs hold particularly sophisticated specialist knowledge relevant to the history, and future, of science. We believe our project succeeds as a powerful first step in liberating such metadata from its MARC silo, without resorting to a simplistic mapping that carries over the constraints of the 50-year-old format. A highly-structured entity-relationship model for bibliographic metadata facilitates discovery and access for both human users and software applications. We aim to develop additional tools on this framework that would be impractical with a MARC-based catalog.

Poster Number: 69

Time: Friday, July 24, 10:30 a.m.–4:30 p.m.

Are Commonly Searched Databases Optimized for Systematic Review Exporting Needs?

Louisa C. Verma, AHIP, Medical Reference & Electronic Content Librarian, Huntington Hospital Health Sciences Library, Huntington Hospital, Pasadena, CA; **Andy Hickner**, Education and Outreach Librarian, Weill Cornell Medicine, Woodside, NY; **Kate M. Saylor**, Informationist, Taubman Health Sciences Library, University of Michigan–Ann Arbor

Background: The purpose of this project was to explore and compare the export functionality on databases used in systematic review (SR) searching. Recent research has shown an increase in publication trends for SRs substantiating a need for easy exporting of large sets of citations. Information professionals in the User Experience Caucus (previously called "Features, Flaws & Fixes" prior to caucus formation) discussed download limits as a pain point issue with some interfaces. Systematic reviews are exhaustive by nature and often return over 1000 results per database. Interface limitations for exporting results may present obstacles to conducting SRs or introduce errors in meta-analyses.

Description: The authors tested the degree to which platforms allow exporting of large sets and other obstacles faced in the export process. Databases frequently used in SR searches were selected, including: Ovid (MEDLINE®, EBM Reviews), PubMed, Scopus, Cochrane Library (Wiley), Web of Science, EBSCO (PsycINFO,CINAHL), Embase, ClinicalTrials.gov and Google Scholar. Data were extracted including export limits, export formats, exportable fields, download options, API availability, intuitiveness, speed, citation selection ease and deduplication. Each author conducted high recall searches and tested export and related functionality of database platforms available to them. Additionally, the authors noted any obstacles encountered when exporting, downloading and uploading citations.

Conclusion: Some platforms allow large sets to be exported and others restrict either through the export itself (WOS, Ovid) or through the inability to select citations easily (GoogleScholar). The most restrictive platforms were GoogleScholar and Web of Science. PubMed, Embase, Cochrane Library (Wiley) and ClinicalTrials.gov were the least restrictive, while Ovid and Scopus had limitations which still may cause obstacles for some SR topics.

Poster Number: 70

Time: Friday, July 24, 10:30 a.m.–4:30 p.m.

Beyond the Buy-in: One Year after Building an Institutional Repository

Amanda Schwartz, Digital Asset Librarian, Providence St. Joseph Health, Missoula, MT

Background: In 2018 Providence St. Joseph Health launched an institutional repository to showcase research in a global and consumable way. Year one comprised of establishing the repository, gathering materials, and promoting services to administrators, clinical, and research staff.

Year two built on the momentum of the initial buy-in, focusing on growing submissions, incorporating special collections, and detailed marketing outreach utilizing altmetrics.

Description: In year one, the Digital Asset Librarian conducted outreach to major research groups and departments within the organization. Coupled with the utilization of advanced search algorithms, outreach yielded monthly article and presentation submissions to the institutional repository. Submissions from outreach and search algorithms maintained consistent monthly growth of materials.

Following the success of year one outreach initiatives, year two focused on maintaining materials growth and utilized altmetrics usage and interaction statistics as a visual marketing strategy.

User interaction increased 277% from year one, growing engagement from 3,656 individual metadata page hits to 10,147. Altmetrics tools reflected a similar spike in usage, with social media engagement escalating from 12,335 interactions to 42,105 interactions. Initial outreach for year two altmetrics marketing was well received. Researchers and administrator feedback noted appreciation towards seeing the whole impact of publications.

Conclusion: Evidenced by continued usage and positive reception, the institutional repository creates an investment between user, author, and institution. The institutional repository usage remains global and dynamic, reaching 111 countries in 2019, averaging a 300% increase in engagement between usage and readership. Additionally, marketing altmetrics as a visual piece of the growing engagement has helped create a well-rounded picture of organizational scholarly materials. Future and continued outreach will be necessary to maintain buy-in between the authors and library staff managing the repository.

Poster Number: 71

Time: Friday, July 24, 10:30 a.m.–4:30 p.m.

Engaging Colleagues for Feedback on a Journal Identification Decision Support Tool

Mallory Blasingame, Information Scientist, Center for Knowledge Management, Vanderbilt University Medical Center, Nashville, TN; **Taneya Y. Koonce**, Associate Director for Research, Vanderbilt University Medical Center, Nashville, TN; **Jing Su**, Information Scientist, Vanderbilt University Medical Center, Vanderbilt University Medical Center, NASHVILLE, TN; **Annette M. Williams**, Senior Information Scientist, Center for Knowledge Management/ Vanderbilt Medical Ctr., Nashville, TN; **Jerry Zhao**, Senior Application Developer, Vanderbilt University Medical Center, Nashville, TN; **Spencer J. DesAutels**, Information Scientist, Vanderbilt University Medical Center, Nashville, TN; **Dario Giuse**, Associate Professor, Vanderbilt University Medical Center, Nashville, None; **John D. Clark**, Sr Application Developer, Center for Knowledge Management, Vanderbilt University Medical Center, Nashville, TN; **Nunzia B. Giuse, FMLA**, Vice President for Knowledge Management | Professor Biomedical Informatics, Medicine, Vanderbilt University Med Ctr, Nashville, TN

Objectives: To describe how the Center for Knowledge Management team at Vanderbilt University Medical Center effectively engaged the knowledge and expertise of external colleagues for feedback on the Scholarly Publishing Information Hub (SPI-Hub™), a decision support tool that helps prospective authors identify journals in which to publish. We used the well-proven model employed by vendors to conduct live demonstrations and formal/informal evaluations of the tool at a professional library regional chapter meeting.

Methods: Information scientists attended an MLA chapter meeting as exhibitors and provided information about and live demonstrations of our decision support tool to attendees. Developed in-house, the tool provides prospective authors, through a variety of mechanisms (e.g., author topic of publication, known journal titles, colleagues’ journal preferences as viewed through their ORCID iD), a list of scholarly journals in which to publish their work. It retrieves 25 metadata record fields indicative of quality and transparency for each journal recommendation. Following each encounter, interested participants were provided remote access for one month to further evaluate and assess the tool as they share it with their local users, and provide feedback through an IRB-approved survey. In addition to these structured survey responses, commentary was highly encouraged on site by the team through informal conversations and more formal demonstrations of the tool’s capabilities.

Results: Survey respondents (n=10) gave a median score of 4 on a 5-point Likert scale for outcomes assessed: coverage, ease of navigation, relevance of search results, clarity of information, and overall usefulness. Although we received only 10 completed surveys, the live demonstrations allowed the team to discuss and demonstrate the project to the majority of meeting exhibit attendees. In total, we engaged in informal discussion with over 80 professional colleagues, and that provided a wealth of feedback greatly surpassing our expectations. Comments fell into four main categories: content coverage, overall functionality, ease of navigation, and ideas for future development.

Conclusions: The feedback indicated buy-in from our colleagues as major user stakeholders and affirmed the tool’s value. One faculty comment enthusiastically described how the tool facilitated discovery of new journals and citations that would otherwise have been overlooked. This was exciting, as the re-introduction of serendipity as a form of journal discovery was one of the motivating factors for the development of the tool. Our experience suggests leveraging the tested exhibitor model for a non-traditional project is a valuable method for gathering external feedback and establishing user engagement from expert target stakeholders.

Poster Number: 72

Time: Friday, July 24, 10:30 a.m.–4:30 p.m.

From Acute Care to Zygomycosis: Managing a Nursing/Health Sciences E-Book Collection

Camille McCutcheon, Coordinator of Collection Management and Administrative Services, University of South Carolina Upstate, Spartanburg, SC

Background: A change in nursing librarian presented an opportunity to purchase nursing/health sciences titles primarily in e-book format at a regional comprehensive university with a bachelor of nursing (BSN) and a master of nursing (MSN) in clinical nurse leadership. The purpose of this project was to identify new titles; standard resources such as drug guides; and course-related titles requested by nursing faculty members that were available to be purchased in e-book format. Objectives included working with vendors to identify e-book titles/collections, identifying gaps in the library collection, and improving collection management by creating a management schedule for reviewing these resources.

Description: In order to stay abreast of what was currently being published, the nursing librarian worked with the representative from GOBI, a library acquisitions tool, to create notification alerts for new titles and with the EBSCO representative to create notification alerts in EBSCO Collection Manager (ECM) for nursing publishers. These alerts were useful in determining when a new edition of a standard title was published. Web sites for GOBI and ECM also contained featured title collections that were regularly updated. In addition, the librarian consulted annual best book lists, such as the American Journal of Nursing Book of the Year Award winners, Doody’s Core Titles®, and CHOICE’s Outstanding Academic Titles for the Health Sciences. The librarian contacted the Ovid, McGraw-Hill, and Elsevier representatives to inquire about relevant e-book collections. Input from nursing faculty was obtained to purchase course-related titles.

Conclusion: The alerts, the annual lists, and the featured collections were all used to identify titles. Nursing faculty feedback and usage statistics were some of the factors used in considering how often an updated edition of a title should be purchased. A management schedule was created. An Excel spreadsheet was used to track how often editions of these titles were published, how often the library would purchase updated editions, and when previous editions would be withdrawn. Moving forward, this process will improve collection management by enabling better informed purchasing decisions which are critical in a time of limited budgets.

Poster Number: 73

Time: Friday, July 24, 10:30 a.m.–4:30 p.m.

Health Sciences Library Collections Assessment for a New Medical School: Reviewing the Past, Establishing the Present, Forecasting the Future

Aidy Weeks, AHIP, GME Liaison Librarian & Collections Manager, UNLV, Las Vegas, FL

Background: The **University of Nevada, Las Vegas School of Medicine** welcomed their first class of medical students in July 2017. Prior to and during this period, the Health Sciences Library faculty worked diligently to develop a collection that would support the curriculum needs of **undergraduate medical students** and **residents/fellows** in the graduate medical education programs. While it was imperative to meet the needs of the medical school faculty, proper assessment of the collection was less urgent. Since the School’s opening, two fiscal year cycles have passed providing a rich usage dataset and therefore an opportunity to thoroughly examine the collection.

Methods: The collection assessment involved a multistep process that was outlined in a proposal and provided to the Health Sciences Library director. The process began with an **environmental scan** of the existing collection and acquisition practices; **a review of the collection management literature** to establish an evidence-based approach for assessing a health sciences library collection; a **thorough review of usage statistics** through two fiscal year cycles of databases, ebooks, and e-journals and a **benchmark comparison report** of similar collections from aspirational/peer institutions.

Results: Collections strength: The initial acquisition efforts of health sciences resources by UNLV Health Sciences Library faculty and Collections Development staff set UNLV’s collection on par with regional aspirational institutions database, journal, and ebook coverage. **Collections weakness:** In reviewing usage statistics for FY2018 & 2019 it was noted that certain materials were outside the collections scope and also carried poor usage. **Collections gaps:** In reviewing both usage statistics and benchmark data the UNLV HSL Collection lacked top-tier oncology titles. This makes sense since there is no oncology program at the UNLV School of Medicine, however this is an area to pay closer attention to for the future.

Conclusion: The assessment also provided insight into how a brand new collection was established, as well as, building a site repository to support the future development and management of health sciences library collections including storing this assessment, policies, tutorials and resources accessible to the UNLV Health Sciences Library faculty and staff. The **benchmark comparison report** highlighted in this poster, proved to be a very useful tool in determining how well the collection stands at present and also areas to focus on in the future.

Poster Number: 74

Time: Friday, July 24, 10:30 a.m.–4:30 p.m.

How Long Should We Keep Data? An Evidence-Based Recommendation for Data Retention Using Institutional Meta-Analyses

Drew Wright, Research Librarian, Weill Cornell Medical Library, Weill Cornell Medical College, New York City, NY; **Michelle Demetres**, Scholarly Communications Librarian, Weill Cornell Medical College, New York, NY; **Keith C. Mages, AHIP**, Clinical Medical Librarian, Weill Cornell Medicine, Weill Cornell Medicine, New York, NY; **Antonio P. DeRosa, AHIP**, Oncology Consumer Health Librarian, Weill Cornell Medicine, New York, NY; **Caroline Jedlicka**, Clinical Medical Librarian Intern, Weill Cornell Medicine, New York, NY; **Judy Carol Stribling, AHIP**, Assistant Director, Clinical Services, Weill Cornell Medical College, New York, NY; **Becky Baltich Nelson**, Education and Clinical Librarian, University of Massachusetts–Worcester; **Diana Delgado, AHIP**, Associate Director, Information, Education and Clinical Services, Weill Cornell Medicine, New York, NY

Objectives: The National Institutes of Health (NIH) is committed to ensuring results and data are made available to the public for research they fund, in whole or in part. Therefore, institutions and researchers must maintain accessible, sharable data for published studies. Using published meta-analyses (MAs) we sought to establish an evidence-based recommendation on how long data should be kept and shared.

Methods: Using Web of Science (Core Collection – Clarivate Analytics) we identified all MAs with an affiliation of Weill Cornell Medicine (WCM) published through November 2019. The following search was used and identified 211 WCM affiliated meta-analyses: OO=(weill) AND TI=("meta-analysis") AND DT=(Article OR Review). The full text of each MA was reviewed. We then looked at all studies that met inclusion criteria for each MA and documented the publication date of the oldest included paper. For all 211 included MAs in this study, journal category (disciple) and impact factor was identified per Thomson Reuter's Journal Citation reports via Web of Science. All data and trends were analyzed using Power BI (Microsoft).

Results: The average age of included studies is 17 years. The oldest data set utilized in a MA was 51 years old at the time of publishing, and the youngest was 2. WCM meta-analyses ranged in discipline, covering 37 out of 76 Web of Science medical-related journal categories. There appeared to be no strong association between data age and impact factor, but there is a possible correlation with data age and year of MA publication suggesting that newer publications include older studies relative to themselves.

Conclusions: Based on our findings we recommend data be stored for sharing for a minimum of 17 years. This study lays groundwork for larger future studies.

Poster Number: 75

Time: Friday, July 24, 10:30 a.m.–4:30 p.m.

Inclusivity in Research Data Management: Focus on the Future Impact of Research

Jessica A. Koos, AHIP, Health Sciences Librarian/Senior Assistant Librarian, Stony Brook University Health Sciences Library, Stony Brook, NY; **Laurel P. Scheinfeld**, Health Sciences Librarian, Stony Brook University, Stony Brook, NY

Objectives: Increasing attention towards transparency and reproducibility in research has highlighted the importance of data management strategies. However, it is essential to consider how RDM tools, systems and practices may impact diverse communities. This poster is an exploration of some of the various challenges to inclusivity in research data management, and potential solutions for librarians to recommend when assisting researchers.

Methods: The investigators conducted a literature review on the topic using multiple academic research databases including PubMed, Web of Science, Library, Information Science, & Technology Abstracts, SOCIndex and Academic Search Complete. Google and Google Scholar were also searched in order to identify additional literature. Some examples of the terms used in the search strategy were “data management,” “inclusivity,” “ethics,” and “accessibility.” Next, the authors reviewed the results and identified articles discussing inclusivity as it pertains to RDM. The authors then conducted a thematic analysis of these articles using an inductive approach.

Results: Twenty-one documents that met the above criteria were identified. The document types included commentaries, editorials, research articles, a blog post and a scoping review. Several themes were identified, including inclusivity in the research process, privacy, consent, community engaged research, access to data, interdisciplinarity, transparency and metadata.

Conclusions: Librarians should consider how each of these themes may affect the outcomes of various research projects, and educate researchers on ensuring that they are taking the necessary steps to increase inclusivity throughout the data management lifecycle. This approach can help to reduce healthcare disparities, while allowing a greater number of individuals from varied backgrounds to benefit from the results.

Poster Number: 76

Time: Friday, July 24, 10:30 a.m.–4:30 p.m.

Moving beyond Assumptions for E-Book Firm Orders: Applying User Experience Methodology to Learn What Health Sciences Students Actually Value on E-Book Platforms

Karen Gau, Health Sciences Collection Librarian, Virginia Commonwealth University, Virginia Commonwealth University, Richmond, VA; **Ashley Brewer**, Web Systems Librarian, Virginia Commonwealth University Libraries

Objectives: To determine which ebook platform features are valued by health sciences students, in order to inform the decision-making process for ebook firm orders.

Methods: A 45-minute focus group session (n=6) was jointly conducted by the Health Sciences Librarian and Web Systems Librarian with a health sciences library’s student advisory committee, comprised of students from four out of five schools on the health sciences campus of a public research university. Applying user experience methodology, students noted how often they used ebooks in a provided worksheet, and then were given independent time to explore and take notes on various platforms, using example texts chosen for their discipline. Each platform exploration was followed by a guided discussion on that platform. After reviewing and discussing three different ebook platforms, the students completed a final survey on how important various features were to them, and how they would rank each platform in order of preference.

Results: Focus group participants appeared to prefer print page formatting with the conveniences of having an ebook available online, such as having the Table of Contents on the side or being able to click and immediately access a bibliographic citation. Desktop/laptop access and highlighting were important as well.

Conclusions: The discussion and survey results provided the health sciences collection librarian with information on the value of ebook platform features to students, and has assisted her with ebook platform selections for firm orders. Because the findings of this study are specific to the VCU health sciences student community, it is strongly recommended that other collections librarians consider conducting similar focus group sessions at their own institutions to learn which platform features their user groups prefer.

Poster Number: 77

Time: Friday, July 24, 10:30 a.m.–4:30 p.m.

Neuroimaging Data Primer: A Resource for Curating Digital Imaging and Communications in Medicine (DICOM) and Neuroimaging Informatics Technology Initiative (NIfTI) Files

Sara Samuel, Informationist, University of Michigan–Ann Arbor; **Michael Moore**, Grants & Special Projects Librarian, University of Washington Health Sciences Library, Seattle, WA; **Brandon Patterson**, Technology Engagement Librarian, University of Utah–Salt Lake City; **Christopher J. Sorensen**, Senior Support Scientist, Washington University School of Medicine in St. Louis, St. Louis, MO; **Helenmary Sheridan**, Data Services Librarian, University of Pittsburgh, Pittsburgh, PA

Background: Academic libraries play a foundational role in supporting and developing open research practices by providing curatorial support and hosting for research data sets, including those from biomedical research. The Data Curation Network is a network of data curators designed to provide a wider variety of curation services than offered at a single institution. Our team formed during a November 2019 workshop offered by the Data Curation Network, and for our final project we decided to create a primer that will help data specialists curate neuroimaging files.

Description: Following internal discussions and scoping, our team conducted interviews with researchers who work directly with medical imaging to better identify workflows, common file formats, and core concepts to include in the primer. Based on those findings, we decided to focus our primer on fMRI (Functional Magnetic Resonance Imaging) data that has been saved in DICOM (Digital Imaging and Communications in Medicine) and NIfTI (Neuroimaging Informatics Technology Initiative) formats. Team members supplemented researcher feedback with literature reviews and online resources to establish the primer's informational foundation. The primer provides a succinct but thorough introduction to the two file formats, giving a data specialist enough information to successfully ready a set of files for deposit into a data archive. Topics covered include file structure and common extensions, the Brain Imaging Data Structure (BIDS), and patient privacy and confidentiality concerns.

Conclusion: The primer underwent peer review in early Spring 2020 by other cohorts from DCN workshops, and feedback was incorporated by the project team. The open access primer will be deposited into an institutional repository and made openly available for anyone to download and refer to when curating neuroimaging data. All DCN primers are designed to be living documents that can be updated and tweaked by other data curators. To support future collaborative editing and maintain versioning control, the primer is shared on DCN's GitHub alongside other data curation primers, where it can be updated as needed.

Neuroimaging DICOM & NIfTI Primer on GitHub

Poster Number: 78

Time: Friday, July 24, 10:30 a.m.–4:30 p.m.

Project META: Aligning Medical and Engineering Vocabularies for Topic Mapping and Content Extraction

Margaret (Peg) Burnette, Associate Professor and Medical & Biomedine Librarian, University of Illinois at Urbana-Champaign, Champaign, IL; **Eliot Bethke**; **Jenny Amos**, Teaching Associate Professor, University of Illinois at Urbana-Champaign, Urbana, IL

Background: The Carle Illinois College of Medicine welcomed the inaugural class of 32 students in July of 2018 and continues to build a one of a kind curriculum that infuses engineering principles and competencies throughout the medical curriculum. The development of each course is led by a triad of educators – one clinician, one basic scientist, and one engineer. Initial efforts to match engineering competencies to logical medical topic areas and course objectives were hindered by differences in the respective languages of the two disciplines. The main goal of this project is to identify a corpus of terms to represent engineering concepts that are meaningful to both engineering and medical faculty.

Description: Engineering faculty identified a list of 130 core engineering concepts for the curriculum. Authors searched each of the terms in MeSH, IEEE, and NASA thesauri, and ISO Terms & Definitions, to identify the medicine and engineering terms that reflect the best alignment. The resulting 200+ terms represent exact, close or partial matches, parasynonyms, and alternative or related terms. Elimination of duplicates left 169 terms that divided into six broad topic categories created through consensus. A goldilocks approach was then used to assign “too broad”, “too narrow”, or “just right” designations for each term, resulting in a final list of 59 “just right” terms.

Results and Conclusions

The instances of each of the 59 concepts were mapped to engineering content areas of the curriculum over the past two years. This revealed both overlap and gaps that will inform future curriculum planning and engineering labs development. With a goal to identify appropriate terminology meaningful to both medicine and engineering, manual processes proved necessary and term context is critical. The degree of success of the term extraction process is largely subjective and influenced by disciplinary affiliation. While this process allows for some automated natural language processing techniques, significant human intervention is still required. The goldilocks approach provides a “best match” that negates the need to deal with any hierarchical arrangement. The terms will be “test driven” for effective use for the Carle Illinois curriculum and logical connections to specific medical topics in the curriculum may surface. Biomedical education, research, and practice are becoming increasingly interdisciplinary and leveraging controlled vocabularies to build a common language that is meaningful across disciplines can contribute to more effective interdisciplinary collaborations.

Poster Number: 79

Time: Friday, July 24, 10:30 a.m.–4:30 p.m.

Redesigning an Enterprise-Wide Intranet Library Site Using a User Experience Design Process

Valerie J. Lawrence, Consultant, Care Management Institute; **Sara Pimental, AHIP**, Senior Consultant; Kaiser Permanente, Oakland, CA

Background: This poster examines the value of using a user experience design (UXD) methodology to inform the redesign of an enterprise-wide intranet library site. Team members, including librarians, content managers, and web developers, partnered with a locally based deep immersion research firm and an internal user experience/user interface (UX/UI) team. The library’s clinician advisor group was also solicited regularly for their input.

Description: The organization’s intranet library, which provides access to internally-created clinical guidance documents as well as to librarian services and electronic resources, had not been redesigned since 2010. The redesign process began in early 2017 using a user experience design (UXD) methodology. This process employs ethnography studies, intensive usability testing, and user surveys to inform design. The team partnered with a deep immersion research firm to perform ethnography studies to gather information about clinician information-seeking behavior. They also partnered with an internal UX/UI team to conduct usability testing and to survey users as design prototypes were available. These partnerships allowed the team to develop a comprehensive plan that incorporated the latest in usability practices into the site redesign.

Conclusion: The redesigned site was released at the end of March 2019 with a cleaner, more modern look and feel and an improved browse hierarchy. Usage statistics from the five-month period immediately following the release (April - August 2019) show a 25% increase in use as measured in unique visitors to the site compared to the same period in the previous year. User satisfaction was positive as measured by user survey data collected by the UX/UI team and user feedback collected through the site feedback form.

Poster Number: 80

Time: Friday, July 24, 10:30 a.m.–4:30 p.m.

So What Do We Have in This Library, Anyway? Launching an Interdepartmental Cataloging Project for a Health Sciences Library’s Print Collection

Karen Gau, Health Sciences Collection Librarian, Virginia Commonwealth University, Virginia Commonwealth University, Richmond, VA

Background: Over the last century, the cataloging migrations of the Tompkins-McCaw Library for the Health Science’s print collection have resulted in an incomplete inventory of holdings, currently estimated to include over 450,000 items. Facing a move in the next several years, the health sciences collection team realized that it needed to update the catalog in preparation for items being moved or withdrawn. A move preparation working group was convened with members from four different departments to develop plans for and oversee the initial implementation of this project.

Description: It was determined that a pilot project for processing the library’s dental journal titles -- which are part of a print retention program -- would be a useful starting point for establishing a workflow that could be applied to the rest of the library’s journal collection, and for determining the project's feasibility at current staff levels. A workgroup comprised of collection development, serials, cataloging, and preservation librarians and staff collaborated on producing a workflow for the dental journal titles. To maximize sharing, a Google Sheet was used to log the time spent, findings, and specific activities completed for the pilot. Following cataloging training, preservation analysis, and journal vacuuming, the linking of this collection started in mid-September 2019 and was mostly completed by December 2019. Approximately 42% of items were unlinked, and 73% of holdings needed to be revised. A manageable number of items were identified as requiring preservation attention.

Conclusion: Although some work is still in progress for the pilot project, a ballpark assessment indicates that it would be beneficial to move forward with linking the rest of the library’s journal titles for further analysis. Due to the success in planning and executing the pilot, the workflow for the rest of the journal collection is anticipated to remain largely unchanged, with the exception of transitioning a majority of the linking process to the health sciences library’s on-site staff, who will continue to work closely with serials and cataloging staff to ensure proper processing. In several months, the workgroup will conduct a follow-up evaluation of the project.

Poster Number: 82

Time: Friday, July 24, 10:30 a.m.–4:30 p.m.

What’s Up with the Supp?: An Analysis of Supplementary Materials in PubMed Central

Sharon Kim Han, 2019-2020 Associate Fellow, National Library of Medicine, Bethesda, MD; **Brenna Cox**, Associate Fellow, National Library of Medicine, Washington D,C., DC; **Eden Kinzel**, 2019-2020 Associate Fellow, National Library of Medicine, BETHESDA, MD; **Louise To**, Associate Fellow, National Library of Medicine, Gaithersburg, MD

Objectives: The National Library of Medicine (NLM) aims to improve discovery, interoperability, and preservation of datasets and other supplementary materials archived alongside articles in PubMed Central (PMC). To that end, the authors analyzed a subset of these articles to identify current practices and opportunities in supplementary information and data sharing in biomedical journals.

Methods: The authors examined successive sets of journals to develop and implement a protocol for examining supplementary materials in PMC. Twenty journals were selected from four common broad subject terms: Biology, Genetics, Medicine, and Neoplasms. From these journals, articles published with supplementary materials between July 1 and December 31, 2018 were analyzed based on presentation and content. Data collection included indicators of data sharing, such as data availability statements, file formats, and file content. The dataset was also analyzed to determine potential trends across subjects.

Results: The authors examined a total of 8,765 files associated with 1,466 articles. 42% of the articles shared only one supplementary file. PDFs were the most common of the 100+ file formats found. Breakdown of file formats by subject reflects wide variation in both types and frequencies. Files most frequently had either at least one table or one figure, and they often contained more than one content type. Data availability statements were not common overall, but some subjects implemented them more frequently than others.

Conclusions: While the sample represents a small subset of articles and files in PMC, our results do not reflect any consensus on supplementary materials sharing, both within and across subjects. Such results reinforce the necessity of establishing and enforcing supplementary materials guidelines for publishers and authors. Next steps for this research include comparing data with journal policies on supplementary materials to measure compliance, as well as continued collaboration with stakeholders to identify priorities for supporting discovery and access.

Poster Number: 83

Time: Friday, July 24, 10:30 a.m.–4:30 p.m.

Which Databases Are Used Most Often? "Real Life" Database Usage in Published Systematic Reviews

Jennifer C. Westrick, AHIP, Library Research Information Specialist; **Patricia Chavez**, Library Research Information Specialist; Library of Rush University Medical Center, Chicago, IL

Objectives: When assisting with systematic reviews, librarians are often asked questions regarding database usage, such as which databases are used the most or how many databases other researchers generally search. The objective of this research is to determine “real life” database usage statistics for systematic reviews.

Methods: Published systematic reviews should include a reproducible search strategy, including a listing of the databases used to conduct the search. To locate these search strategies, a search was run using PubMed’s filter for “systematic reviews.” The top ten databases used in these systematic reviews were entered into a spreadsheet so that total usage numbers could be tallied. This provided a clear picture of usage statistics such as which databases were searched, which MEDLINE interface was used, and the number of databases searched.

The search was run with the keyword “diabetes,” which was chosen due to its wide range of possible types of interventions (pharmacological, exercise, population studies, etc.). A two month filter was applied to limit the number of citations, resulting in almost 300 citations.

Results: The most commonly used database was PubMed, followed closely by Embase. MEDLINE was present in every search strategy. Researchers tended to use either PubMed or Ovid; searches that included both were rare. The most popular number of databases to search was three or four.

Conclusions: While researchers rely heavily upon MEDLINE, especially PubMed, there is wide variety in the databases used to search for systematic reviews on diabetes. Some of this variation can probably be attributed to researchers’ access to the various databases. This analysis reveals interesting patterns and trends of how databases are used in “real life” when conducting systematic reviews on diabetes.

Poster Number: 84

Time: Friday, July 24, 10:30 a.m.–4:30 p.m.

Stages of a Grief Collection: Creating a Focused Section in a Hospital Library

Adela V. Justice, AHIP, Senior Librarian, The Learning Center, UT MD Anderson Cancer Center, Houston, TX

Background: A cancer hospital library created a separate “Grief/End of Life” section within its larger book collection. Books with a grieving/end of life focus were gathered from their various and differently catalogued locations and relocated to a new area. This area is strategically located adjacent to the library’s pamphlets on grieving, end of life, and hospice. This created a larger, focused area on these topics so library users can have a streamlined browsing experience.

Description: The first stage of identifying which books would be appropriate for this new collection was for library staff to collectively self-nominate relevant books. Some titles were easier than others to identify, such as classic books on grief and dying. The second stage was to create a new location in the cataloging management system for these titles, so they could be located when users and staff searched the online catalog. The third stage was to better define the parameters of the collection. Scope and content decisions were made so that staff had an understanding and agreement on what titles--present and future--would be appropriate for this area.

Conclusion: The topics of grief and end of life are certainly ones that present themselves in a cancer hospital library. While our library has always supported patients and caregivers by collecting on these topics, this new and thoughtfully maintained area serves these specific information-seekers in an improved, focused manner. Circulation data is being monitored within the cataloging management system, and the most updated numbers will be shared on this poster.

Poster Number: 86

Time: Friday, July 24, 10:30 a.m.–4:30 p.m.

“We Cordially Invite You”: Characteristics of Email Solicitations from Potentially Predatory Conferences

Robin O'Hanlon, Associate Librarian, User Services; **Johanna Goldberg**, Research Informationist II; **Celine Soudant**, Research Informationist II; **Donna S. Gibson**, Director of Library Services; Memorial Sloan Kettering Cancer Center, New York, NY

Objectives: The characteristics of predatory journals have been explored extensively in the fields of medicine, basic science, and information science. However, predatory conferences, a relatively new development in the realm of fraudulent research dissemination, have yet to be explored at length. This descriptive study will attempt to identify common characteristics of email solicitations from potentially predatory conferences.

Methods: We collected data from a convenience sample of emails from potentially predatory conferences publishers sent to health sciences librarians at a comprehensive cancer center over a six-month timeframe. Data points include conference name, role offered, conference fee, location, organizer name, and length of time from solicitation to conference date. Additionally, we investigated conference peer review to determine if the conferences met the criteria for predatory conferences.

Results: Between October 2019-April 2020, we received 86 total email invitations to potentially predatory conferences (n=52 original invites and n=34 reminders), and invitations shared several traits. Most invitations did not describe the peer review process (95%), nearly one third (32%) invited the invitee to attend as a speaker, and 25% of invites were issued from the same organizer (BIT Congress Inc.). Significant variation was also found among invitations in terms of conference location (n=26), discipline (n=18), and organizers (n=25). The impact of the COVID-19 pandemic was evident on the rate of invitations, with invitations dropping 70% between March and April 2020.

Conclusions: While many solicitations for potentially predatory conferences share common characteristics, the actual conferences these solicitations represent are more diverse in nature than initially anticipated, spanning multiple locations and disciples. While about half of the invitations were sent to individuals with little or no expertise in the corresponding conference topic, 50% were potentially relevant to the invitee, creating an added layer of complexity for invitees attempting to decipher if these conferences are legitimate. The decline in conference invites in relation to the growth of the COVID-19 pandemic indicates these conferences likely take place in brick-and-mortar settings. Motivations for researchers attending these potentially predatory conferences is ripe for further study.

Poster Number: 87

Time: Friday, July 24, 10:30 a.m.–4:30 p.m.

A Box Outside the Box: Introducing and Marketing a Laptop Self-Checkout Kiosk Outside the Library

Beth Auten, AHIP, Health & Human Services Librarian; **Bob R. Price**, Associate Dean Technology & Digital Strategies; UNC Charlotte, Charlotte, NC

Background: After years of planning, J. Murrey Atkins Library implemented a laptop self-checkout kiosk in the College of Health & Human Services building in January 2020. Our laptop checkout service within the library is popular with students, and this new kiosk allows students in the health and human services disciplines, as well as other students in nearby buildings, a convenient option for borrowing a laptop. This is the first self-service laptop kiosk on our campus, and at any of the state universities in our system.

Description: The library’s Technology & Digital Strategies Department, with the support of the Senior Associate Provost, purchased and installed the laptop vending kiosk. Once the kiosk was installed, training was provided for library faculty and staff by members of the Technology & Digital Strategies team to ensure our public services team can answer questions and provide front-line support to interested students and faculty members. The Health & Human Services Librarian coordinated marketing and communication about the kiosk to the faculty, staff, and students in the College of Health & Human Services, as well as to other units on campus.

Results: The usage of the kiosk was assessed during January-March 2020. During this time period, laptops from the kiosk constituted 21.1 percent of all library laptop checkouts. The number of checkouts increased over time throughout the first part of the semester, until Spring Break (the week of March 1st.) The majority of laptops were checked out by students in the College of Health & Human Services, though students from other units, and some faculty and staff, borrowed them as well. Among student populations, undergraduate students were the most frequent borrowers.

Conclusion: The closing of the CHHS building and the campus toward the end of March due to the pandemic meant that the kiosk and laptops were no longer available for students to access and use. However, the use of the kiosk by students enrolled in programs and taking classes in the building where the kiosk is housed up until that time does seem to indicate that students often have immediate need for access to a laptop in the vicinity of their courses. While there is ultimate interest in installing self-service kiosks in other locations on campus, these .

Poster Number: 88

Time: Friday, July 24, 10:30 a.m.–4:30 p.m.

Adjusting Our 2020 Vision: The Use of Databases and Information-Seeking Behavior of the Health Sciences Faculty

Irma Singarella, Health Sciences Librarian and Associate Professor, University of Memphis, Memphis, TN

Introduction: The Health Sciences Library is housed in the Community Health Building, University of Memphis, and it is considered a new information center (2015).

Objectives: Determine: a) How often faculty search online resources to find research articles b) What are their reasons for using online resources c) Have they used the resources for clinical care d) What are their information needs that motivates them to search online e) From which location did they access online resources.

Methods and materials: Survey adapted from De Groote, Shultz and Blecic (2014) and Gahn, Watts, and Quesenberry (2018), included 15 multiple choice questions with space for comments. The IRB approved and declared the research exempt in April 2019. School representatives sent the questionnaire by e-mail to 68 faculty members, and reminders two weeks later.

Results: When asked how frequently participants used various databases most (61%), said they preferred to use Google on a daily basis, CINAHL (29%) and UpToDate (29%), weekly; Medline (46%) monthly. Information needs included: Research/Background literature search (31.4%), Keeping current in my field (24.4%), Instructional Preparation (22%), Patient Care Information (18.6%). They also added: “Answer questions from students or clients”; “Teach graduate students” and, “My own verification/Need to know”. They are frequent users of interlibrary loan services. When asked from which location they access online resources, (65%) preferred the University Libraries Website.

Discussion: Many faculty members expressed that they use familiar resources with easy access. Most respondents (63%) expressed that they have access to the full text articles of the journals they need. Some (37%) respondents mentioned that access varied sometimes they don’t get the articles they need. Most participants use Google, Medline and CINAHL for research purposes with low use of other databases. Among the problems in finding literature, they mentioned: “Library cutbacks to full texts”, “Being able to narrow down a topic” and “Articles not available in PDF”. Some respondents mentioned that they needed resources and services that were already available of which they were unaware.

Conclusions: We interpret some of the access challenges as needs for training and more publicity to resources and services. The outcomes of this research project will contribute to improve our online collection to better meet educational and research needs of the faculty and next steps to develop outreach and marketing.

Poster Number: 88

Time: Friday, July 24, 10:30 a.m.–4:30 p.m.

“Scholarly Communications Faculty Advocates”: Disciplinary Professors as Reverse Liaisons between the University Libraries and Academic Units

Christina M. Seeger, AHIP, Pharmacy & Health Science Center Librarian, Medical Sciences Library, College Station, TX; **Sarah Bankston**, User Engagement Librarian, Texas A&M University Libraries, College Station, TX; **Heather K. Moberly, AHIP**, Professor, Coordinator of Veterinary Services, Medical Sciences Library, Texas A&M University, College Station, TX; **Catherine Pepper**, Associate Professor/Coordinator of Library Field Services, Medical Sciences Library, Texas A&M University, Austin, TX; **Barbara Gastel**, Professor/Coordinator of Science Communication Graduate Program, Texas A&M University/Veterinary Integrative Biosciences, College Station, TX; **Michael J. Miller**, Research Scientist II, Mid-Atlantic Permanente Research Institute, Rockville, MD

Background: In 2017, the University Libraries (UL) invited applications for 2 Faculty to serve 2-year joint appointments as Scholarly Communications Faculty Advocates. Although UL did not prescribe a project or method, advocates were expected to work in their respective colleges on promoting online scholarly identity and collaboration and/or promoting open access. To provide an introduction to scholarly communication topics, UL sponsored attendance at the Force11 Scholarly Communications Institute and presented an overview of pertinent tools and resources. Faculty advocates were selected from the College of Veterinary Medicine & Biomedical Sciences (CVM) and the Rangel College of Pharmacy (RCOP).

Description: RCOP focused on working with faculty to promote their online scholarly identities, while CVM focused on open access publishing and scholarly writing. The faculty advocate at RCOP worked with a team from the library to provide workshops and guidance to faculty on tools and resources to increase the visibility and reach of faculty scholarly output and to create a process for including that information in the faculty evaluation process. At the CVM, the faculty advocate’s endeavors included granting local researchers scholarships to her annual intensive course in research writing, organizing open lectures, collaborating with two librarians on a project to identify open access journals publishing papers in veterinary medicine and biomedical science, and providing editorial consultation and scholarly-communication consultation.

Conclusion: The diversity of these projects showcases the wide range of topics included in the area of scholarly communications. Identifying and supporting reverse liaisons within disciplinary faculty allows libraries to expand the impact of their initiatives. Both projects succeeded in expanding scholarly communications initiatives to meet an identified need in a specific population. Working with these academic units on projects with such different focuses provides a framework for expanding the role of the libraries in supporting scholarly communications to other disciplines depending on their individual needs.

Poster Number: 89

Time: Friday, July 24, 10:30 a.m.–4:30 p.m.

Aligning a Science Technology Engineering Medicine (STEM)-Based Library Program with Standardizing LibGuides: A Multidisciplinary Venture

Vedana Vaidhyanathan, Health and Life Sciences Librarian, Baylor University, Waco, TX; **Christina Chan-Park**; **Kenneth Carriveau**, Engineering Librarian, Baylor University, Waco, TX

Background: The three STEM Librarians at our institution decided that because the research on campus is often multi-disciplinary and the students are not being siloed into their disciplines that we needed to have a multi-disciplinary STEM team at our institution. The Health and Life Sciences Librarian, Physical Sciences Librarian and Engineering Librarian have pooled their ideas and are reworking the way we approach librarianship to a team-based model in order to be a stronger team for our students.

Description: In designing our program the Physical Sciences Librarian has been given the new title of STEM Coordinator. In this position she has been holding regular STEM team meetings where we have been redesigning our LibGuides to direct students not just to our liaison responsibilities but also to other places of interest. We hope that this new initiative will cause the institution to see that the STEM students (including Health Sciences) need different attention as they do research. We also plan to hold interdisciplinary workshops to show how a student can work through different science disciplines.

Conclusion: We hope to measure how many people attend our workshops and how many hits we have on our LibGuides, compared to when they were streamlined to discipline and coursework.

Poster Number: 90

Time: Friday, July 24, 10:30 a.m.–4:30 p.m.

An Innovative Editing Service for Medical Students

Lisa M. Price, Public Services/Research Support Librarian, Health Sciences Library, Rowan University School of Osteopathic Medicine Health Sciences Library, Stratford, NJ

Background: The Library serves students, staff and faculty of a medical school and related graduate school of biomedical sciences. Students face the daunting yet crucial tasks of preparing Personal Statements for residency, scholarship and other applications. Students and residents are also encouraged to seek publication opportunities. We recognized a need for proofreading and editing assistance, and one of our librarians had the background and experience to provide such a service.

Description: We developed a flyer announcing available services and distributed it within the library, at student and residency orientations, committee meetings and other appropriate campus events. The service was also announced via the library’s website and Facebook page. Collaboration with the Center for Teaching and Learning resulted in numerous student referrals. The librarian managing the program receives documents to be reviewed via e-mail. She makes comments and suggested revisions in Microsoft Word’s “Track Changes” mode (or other appropriate software) and sends both “marked up” and “clean” versions to the author for review. Further consultation is available, but final editorial decisions are left to the writer.

Conclusion: Since introducing the service in June 2015, we have received numerous submissions of documents for review from students and staff members. Personal statements for Residency and Fellowship applications constitute the majority. Other submissions have included essays for medical school applications, conference posters, articles/submissions for publication, CV/resumes, scholarship applications, rotation audition applications and independent study final papers. Feedback has been extremely positive and we anticipate continuing to offer this service.

Poster Number: 91

Time: Friday, July 24, 10:30 a.m.–4:30 p.m.

Applying Citation and Usage Analysis to Evaluate the E-Journal Package Collection in a Medical University Library

Hua-Yu Hsu, librarian, Taipei Medical University, Taipei, Taipei, Taiwan (Republic of China); **Tzu-heng Chiu**, Director, Taipei Medical University Library, Taipei, N/A; **Chun-Huei Shen**

Objectives: E-journal packages accounts for 60% of overall e-resources budget in XXX, however, some titles included in them aren’t needed by users. In 2019, authors tried to analyze the title fill rate and cost per full-text download of all XXX e-journal packages, the research results can serve as the reference for our e-journal collection development and decision making in the future.

Methods: Methodologies of citation analysis and cost of usage were applied in this study. The authors believe that the cited journals of our faculty’s papers represent their real research demand, and it could reflect the utilization efficiency of our e-journal collection. Therefore, we exported 2016-2018 publication of XXX faculty from the WOS SCI/SSCI database, which results 4,698 journal articles with 181,422 citations. We then utilized the list-checking method to compare bibliographic data of these 181,422 citations with our e-journal collection for title fill rate to find out how our collection could support the research need of XXX faculty. We also analyzed source journals of those 181,422 citations, including its frequency of publication, times cited, publisher/ package which it belongs to. In addition, the cost per full-text download for each e-journal package were calculated based on their subscription fee and download statistics.

Results: The title fill rate of 181,422 citations is 74% (among which 134,749 are the TMUL e-journal collection). The top 3 most cited e-journal packages in 2016-2018 are "SDOL" (1,080 titles), followed by " WileyOnlineLibrary " (507 titles) and “Springer” (496 titles). The cited rate and cost per download of each e-journal package are shown in Figure 1, the highest utilization efficiency e-journal packages are "JAMA" and "Cell Press" which have lowest cost per full-text download (US$ 0.81, US$ 1.27) and highest cited rate (90.9%, 90.5%). In addition, the lowest utilization efficiency e-journal package is "WileyOnlineLibrary" (US$ 3.6, 53.8%).

Conclusions: Base on the findings, TMUL changed the subscribing mode of “WileyOnlineLibrary” e-journal package from renewing whole package to a customized one in 2019 (reducing the number of titles from 842 to 381). Due to the adjustment, the cost per full-text download of Wiley reduced from USD3.6 (2016-2018 average) to 1.3 (2019) as well. In conclusion, libraries need to consider many aspects when subscribing e-journal collections to support research needs with limited budget. Through the big data analysis of citation and cost of usage, librarians can adjust subscription plan of the low utilization efficiency e-journal packages and make appropriate decisions.

Poster Number: 92

Time: Friday, July 24, 10:30 a.m.–4:30 p.m.

Bioinformatics Services Program: A User Survey after Fifteen Years of Establishment and Service

Eddie Loh, Informatics Specialist in Bioinformatics, USC Libraries / Bioinformatics Services

Background: Bioinformatics, a cross disciplinary amalgamation of computational science, statistics, and biology, has grown to become an important field of study and expertise integral to biomedical and life-sciences research. Driven by rapid technological advancements in the development and mechanization of new molecular biology techniques, the massive amounts of data generated have been deposited into countless online databases which require relevant bioinformatics expertise to efficiently search, access and analyze. The bioinformatics service program at our health sciences library was established in 2005 to cater to the increasing need for bioinformatics support in our university community, and has steadily expanded over the years.

Description: Our program currently consists of three full-time bioinformatics specialists based out of two separate campuses, serving the Medical, Dental, Pharmacy, Biological and Earth Sciences, Bio-Engineering, and Gerontology schools of the university, as well as affiliated hospitals. Our service offerings span a wide range of bioinformatics activities, including research consultation and training, access to bioinformatics software and computational resources, collaborative data analysis, and grant application support. Having completed fifteen years of service, we conducted a university-wide survey in 2019 in order to continually evaluate the evolving demographic distribution of our bioinformatics community, their self-assessment of bioinformatics needs, their evaluation of our current range of service offerings, and their opinions on different models of providing bioinformatics services.

Conclusion: The survey yielded over 300 responses comprising a good mix of faculty, research staff, post-docs, and students. Genomic data analysis, use of open-source, and commercial software, are the top three bioinformatics needs, with a majority of respondents relying on our services. Our top three service categories are bioinformatics software access, workshops/training, and research consultation, with more than 75% of respondents rating them extremely helpful or helpful. On average, over 40% of respondents consider our current services adequate, and over 40% would like to see further expansion. These valuable survey feedback have been incorporated into our strategic plans for the future.

Poster Number: 93

Time: Friday, July 24, 10:30 a.m.–4:30 p.m.

Biomedical Library Expert Search Services (BLESS): A Systematic Review Service Update

Bethany Myers, AHIP, Research Informationist, UCLA, Los Angeles, CA; **Antonia Osuna-Garcia**, Health and Life Sciences Librarian, UCLA Biomedical Library, Los Angeles, CA; **Wynn Tranfield**, Physical and Basic Sciences Librarian, Louise M. Darling Biomedical Library, UCLA, Los Angeles, CA; **Nisha Mody**, Interim Associate Director, National Network of Libraries of Medicine, Pacific Southwest Region, Los Angeles, CA

Background: Librarians at a large research university receive requests for systematic reviews on an individual basis through informal means such as personal connections, e-mail, or referrals. In 2018, the librarians at this institution created a formalized systematic review request service through Jira Service Desk (JSD). Since it launched in early 2019, we have been monitoring the workflow and determining updates and modifications needed.

Description: In 2019, the Biomedical Library Expert Search Services (BLESS) team presented a paper about the formation of a systematic review service through JSD. Since then, the team has received more search requests and used our experiences to update the workflow and processes initially put in place. We added a new team member, which has helped manage the volume of incoming requests. During this time, the team documented and analyzed the number of requests, publications, department requestors, and type of review. To streamline our service, we added and removed databases, updated policy, and modified our workflow based upon various requests we received. Additionally, we informally tracked how we advertise our service focusing on referrals, instruction, and digital signage rather than announcing the service on department listservs.

Conclusion: As we continue to use and refine the systematic review service, we will add a new Institutional Animal Care and Use Committee (IACUC) search request workflow due to demand. We are also considering adding team members from outside the health sciences and including Ovid MEDLINE as a database option. With current utilization, we plan to review and update various aspects of BLESS on a quarterly basis.

Poster Number: 95

Time: Friday, July 24, 10:30 a.m.–4:30 p.m.

Comparing Three Models for Librarian Office Hours in a School of Pharmacy

Emily Gorman, AHIP, Research, Education & Outreach Librarian, University of Maryland, Baltimore, Baltimore, MD

Objective: To determine which of three office hour models was most effective in increasing librarian visibility in and engagement with a school of pharmacy.

Setting/Population: One librarian is the liaison to a school of pharmacy (SOP) that includes approximately 90 full-time faculty, 300 staff, 800 affiliate and preceptor faculty, and 900 students. The library is across campus from the buildings where most SOP personnel are located.

Methods: The librarian held weekly office hours in the main SOP building. The “lobby” model involved two hours per week at a table in the lobby, the “hybrid” model increased the lobby time to four hours and added additional hours in an office, and the “office” model removed lobby hours and increased the office time to a full day (7.5 hours). The librarian tracked all interactions and classified them in the following categories: brief chat, brief hello, brief question, and in-depth question. Descriptive statistics were calculated to compare the number of interactions by category and population.

Results: Results indicate that the lobby model had the most interactions (71), followed by the office model (39), with the hybrid model having the least (29). The office (9) and hybrid (8) models had more in-depth questions than the lobby model (3), but the lobby model had a higher number of brief questions (25) than the office (13) or hybrid (9) models. The number of student interactions differed most drastically, with the lobby model having far more (28) than the hybrid (6) or office (3) models.

Conclusions: Based on these results, holding office hours in the building lobby is most effective for increasing librarian visibility in the SOP. However, people appear to be more comfortable asking in-depth questions in the office setting. There are advantages and disadvantages to each model depending on the goal of the office hours – visibility versus in-depth engagement and support.

Poster Number: 96

Time: Friday, July 24, 10:30 a.m.–4:30 p.m.

Compassion, Storytelling, and Processing: Using LibGuides to Support Schwartz Center Rounds

Ana M. Macias, AHIP, Manager Library Services Sacramento, Kaiser Permanente, Kaiser Permanente NCAL, Sacramento, CA; **Marina Aiello**, Lead Librarian, Instructional Design and Technology, NCAL Libraries, Kaiser Permanente, Stockton, CA; **Joy Rodriguez**, Medical Librarian, Kaiser Permanente, Kaiser Permanente Fresno Medical Center, Fresno, CA

Background: Schwartz Center Rounds is an interdisciplinary forum held at hospitals throughout the United States. Forums offer health care providers a venue where they can safely and candidly discuss difficult emotional issues inherent in providing patient care. An inter-regional hospital library system provides support to local Schwartz Center Rounds programs by providing relevant information resources at the monthly rounds. To increase the library’s visibility in the local medical center and to provide additional support to the local Schwartz Center Rounds Committee, a small team of hospital librarians created a LibGuide to showcase important library resources relevant to the Schwartz Center Rounds.

Description: During Summer 2018, a member of the library team contacted the larger inter-regional librarian group to inquire about their current participation on Schwartz Center Rounds (SCR) at their local medical centers as well as their potential interest in using a LibGuide to host and share SCR-related content with their library patrons. After gathering feedback, a small team of librarians redesigned the existing Schwartz Center Rounds LibGuide to reorganize and update the content as well as add the option for librarians to create and link to their own local SCR webpages. The team also created a template for librarians to use for their local SCR pages that links to the main guide. This resulted in a streamlined, uniform presentation of resources which replaced a previously disjointed and cluttered look.

Conclusion: The project resulted in the Schwartz Center Rounds LibGuide, which includes curated topic lists, library resources, access to related print and e-books, and links to local medical center Schwartz Center Rounds webpages. The project also provided a LibGuides template for each librarian to use to develop their individual SCR webpages.

Since its creation, the Schwartz Center Rounds LibGuide has received almost 400 visits (Nov. 2018 – Nov. 2019), and 8 librarians have developed their own Schwartz Center Round pages for their local SCR committee. The LibGuide and local pages have supported the librarians in performing effective outreach to library patrons.

Poster Number: 97

Time: Friday, July 24, 10:30 a.m.–4:30 p.m.

Covidence versus EndNote: Determining Which Deduplication Method Is More Accurate and Efficient for Systematic Reviews

Christi Piper, AHIP, Reference Librarian, University of Colorado Anschutz Medical Campus, Aurora, CO; **Lilian Hoffecker**, Research Librarian, Univ. of Colorado Anschutz Medical Campus, Aurora, CO; **Ben Harnke**, LIbrarian, University of Colorado–Aurora; **Kristen DeSanto, AHIP**, Clinical Librarian, University of Colorado Anschutz Medical Campus, Aurora, CO

Objective: The purpose of this project is to understand how to accuracy of the automated deduplication done by the Covidence software compares to a manual process of deduplicating results in EndNote. This research will determine the accuracy of Covidence's duplication recognition and identify what elements of a citation prevent a duplicate from being identified.

Methods: A systematic review level search for a clinical topic was run in EMBASE via EMBASE.com, Cochrane CENTRAL, and Ovid MEDLINE. The search results obtained provided the test set for the project. A gold standard set was created by 4 librarians manually deduplicating the results set and agreeing on what constitutes a duplicate record. The result set was then be uploaded into Covidence to formal automatic duplicate identification. The duplicates the Covidence identifies were loaded into an EndNote library and compared against the gold standard set.

Results: Our comparison found that Covidence has a 94% specificity rate and a 100% sensitivity rate, meaning that no false positives were identified by Covidence. These results support that Covidence is a viable automatic deduplication option when using the databases Ovid MEDLINE, Cochrane Library, and EMBASE. Rates of specificity and sensitivity may change depending on the databases used for the search.

Conclusions: Deduplicating results from systematic review searches can be a time-consuming process. With this study we can make initial determinations about whether Covidence's automated duplication identification is a viable alternative to the time intensive task of deduplicating results in EndNote. Additional questions that may develop are how the Covidence system compares to more established deduplication methods and how well Covidence does identifying duplicates based on the databases used in a search.

Poster Number: 98

Time: Friday, July 24, 10:30 a.m.–4:30 p.m.

Data and Statistics from a Restructured Literature Search Service

Lilian Hoffecker, Research Librarian, Univ. of Colorado Anschutz Medical Campus, Aurora, CO; **Christi Piper, AHIP**, Reference Librarian, University of Colorado Anschutz Medical Campus, Aurora, CO; **Kristen DeSanto, AHIP**, Clinical Librarian, University of Colorado Anschutz Medical Campus, Aurora, CO; **Wladimir Labeikovsky**, Bioinformationist, University of Colorado Anschutz Medical Campus, Aurora, CO

Background: In July 2018, an electronic database was implemented to help collect data on a restructured service focused on conducting literature searches. Restructuring was prompted in part by the growing demand for literature searches from our campus faculty and students, resulting in hard-to-meet time commitments by librarian searchers. The demand also meant more searchers were providing the service necessitating standardization across librarians.

Description: The literature search service involves four librarians who are experienced in searching for systematic review projects and an additional two who are capable of conducting simpler searches. The electronic database was developed to not only allow patrons to enter their key information when they make their requests, but also to allow the searchers to collect data on each of their projects, including time commitment, librarian co-authorship for publications, databases utilized, limits applied, software used, formats of delivered results, and more. Data will inform librarians on their individual search activities, but also the library on how much human resources (time worked) and which material resources (databases) were needed for these activities.

Results: In this poster, we present data for a period of 18 months (January 2019-June 2020), for 155 searches of which 31% have been completed, 60% are still in progress and 9% have been suspended. Searchers have devoted about 1818 hours to their searches which amounts to 45 working weeks. About 21 hours were spent on average on scoping review projects while 17 hours were spent on systematic review projects. .

Poster Number: 99

Time: Friday, July 24, 10:30 a.m.–4:30 p.m.

Developing a Vision of an Improved Library Website via Usability Testing

Hannah Friggle Norton, AHIP, Chair, Health Science Center Library - Gainesville, University of Florida–Gainesville; **Matthew Daley**, Web Designer, University of Florida; **James R. Clifton**, web designer, University of Florida–Gainesville; **Gregory T. Clayton**, Circulation Manager, University of Florida–Gainesville; **Bob Lockwood**, Library Assistant, University of Florida, Univ. of Florida, Gainesville, FL

Background: As the entry point to library resources and services, ongoing evaluation and improvement of the library website is vital. At our institution, an upcoming shift to a new WordPress theme provided an opportunity to re-engage library users in assessing the website’s overall strengths and weaknesses. A team of public services staff, IT staff, and a librarian conducted three types of usability tests to solicit feedback from library users.

Description: In the first phase, individuals entering the library were invited to participate in a 5-minute assessment of our website. This assessment consisted of five questions designed to capture broad, overall impressions. Next, all potential library users (especially those who do not visit the library in person) were invited to participate in a 10-question electronic survey, created in Qualtrics. The survey was distributed via links on the library website, Facebook page, and Twitter account and via email from liaison librarians; it included questions about what users are typically looking for on the website, what they use most, what they ignore, and what they can never find. Finally, the team conducted a series of focused interviews of library users, recording their paths and challenges in completing specific tasks using the library website (e.g. finding a journal article, reserving a study room).

Conclusion: Across the three phases, 103 participants provided feedback on the library’s current website. Although responses varied, the team took away several points from participants’ responses: library users find the same information on our website in multiple ways; students frequently use search rather than browse or navigate to find desired information; the overall layout and navigation works for some and doesn’t work for others. This array of feedback proved useful in directing immediate, specific changes to the website (clarifying link text, eliminating an unused external news feed) and guiding the overall implementation of the website’s next iteration.

Poster Number: 100

Time: Friday, July 24, 10:30 a.m.–4:30 p.m.

Development of the Digital Medical Library at the Faculty of Medicine: Challenges, Experiences, and Lessons Learned toward the Students’ Benefit

Alejandra Martinez del Prado, Medical Librarian - Electronic Collections Development, School of Medicine Library, Faculty of Medicine, UNAM, Mexico, Distrito Federal, Mexico

Background: The UNAM (National Autonomous University of Mexico for its initials in Spanish) Digital Medical Library is an important element of the library system of the University and the Faculty of Medicine, since it offers a wide range of academic programs to students, not only from the country but also from the region that come to do specialty programs.

The objective is to provide an overview of the development of the Digital Medical Library (DML) focused on the collections offered to the students and the training done by professional librarians covering the use and access to information resources in order to contribute to their best medical education possible.

Description: The development of the Digital Medical Library (DML) is described, since its creation in 1998 until the most recent update of the webpage in 2018, and it can be represented in mainly four stages. Each phase of the development and evolution shows the digital collections offered to the different users or patrons at each different moment, highlighting the threats and challenges faced which did not allowed to reach the huge community spread all over the country, due to internet access in the country, technology available, enrollment deadlines and costs of resources.

All experiences gained from the four main stages of the DML, surveys conducted, as well as the feedback received from the Faculty of Medicine community (including authorities) have allowed the continuous improvement and development of the DML, always having in mind the purpose to fulfill the users information needs.

Conclusion: Digital libraries are evolving entities, therefore their continuous development is imperative in all medicine schools. Throughout the years, the DML has been improving, according to information and technology updates, particularly it has focused on the collections for the students and their programs. This evolution has allowed the DML to be considered one of the best medical libraries in the country, however, there is more work that still needs to be done. Librarians are aware of it and they work every day with new initiatives to gain a better digital library that results attractive and useful to the entire community. Finally the actual coronavirus pandemic has forced the DML to improve its services more than ever and that is what we are working on right now.

Poster Number: 101

Time: Friday, July 24, 10:30 a.m.–4:30 p.m.

Do They Make Ponchos for Book Trucks? Bringing Our Collections to Our Communities

Katherine A. Morley Eramo, Administrative Coordinator; **Amanda Nevius**, Research & Instruction Librarian - Dental Liaison; **Andrea Minjee Kang**, Research & Instruction Librarian; **Katherine A. Morley Eramo**, Administrative Coordinator; **Amanda Nevius**, Research & Instruction Librarian - Dental Liaison; Tufts University, Boston, MA

Background: There seems to be little discussion around programming in health sciences libraries that go beyond health sciences educational material. However, there are many different ways that libraries can enhance student learning and well-being beyond traditional channels. The Pop-Up Library Program (PULP) concept was adapted to the health sciences setting with themes, such as LGBTQ+ History Month, Leisure Reading, and Asian American History Month, to provide space for de-stressing, as well as important discussions that are related to the health sciences but oftentimes not given focal attention in educational curricula.

Description: PULP was first developed by the main campus library to promote materials by and for people of marginalized identities, as well as to build partnerships across campus. The first PULP at the health sciences campus was in partnership with the dental school's LGBTQA Club in honor of LGBTQ+ History Month. The second was in collaboration with the Student Wellness Advisor to promote self-care during finals. The Online Privacy PULP will be a joint effort with an outside organization to promote awareness of online health and protecting privacy. The program is continually being evaluated with the following: 1) informal feedback at the events; 2) a qualitative online survey distributed to those who have checked out material; and 3) usage statistics on research guides created for each theme.

Conclusion: Through informal communication, participants for the LGBTQ+ and Leisure Reading PULPs shared their appreciation for the event and the diversity of genres within the collections. While no one has yet filled out the online survey, the LGBTQ+ PULP tab on the research guide has 35 unique views. We will also collect this data for the Online Privacy PULP planned for late March. This is a successful launch of a growing program and we are optimistic about the opportunities PULP provides for liaising with our communities beyond academic resources.

Poster Number: 103

Time: Friday, July 24, 10:30 a.m.–4:30 p.m.

Evaluating the Quality of Information Found in the Most Common Health Websites in Google Using DISCERN and CRAAP

Ivan A. Portillo, AHIP, Health Sciences Librarian, Chapman University, Irvine, CA; **Scott Yosuke Johnson, AHIP**, Director of Library Services, Marshall B. Ketchum University, Marshall B. Ketchum University, Fullerton, CA; **Catherine V. Johnson**, Research and Instruction Librarian, University of La Verne, University of La Verne, La Verne, CA

Objectives: Google search results can have an outsized impact on the health information that consumers find and trust. The researchers analyzed the health-related websites that show up most frequently on the first page of Google search results. After determining the websites that appear most frequently, the authors would evaluate the quality of the health information on these websites using the DISCERN instrument and the CRAAP test.

Methods: To replicate a common health information search, a list of search terms was gathered from the most frequently searched health-related topics on Google Trends. Each term would then be searched on Google and the authors compiled a list of the first 10 search results for each term. The authors cleared their search history and cookies from their browsers before searching. They also set their browsers to incognito/private mode and conducted the same search in three different regions of the country using a VPN to avoid any targeted results. The websites that appeared most frequently were then independently evaluated by two of the authors using the DISCERN tool and CRAAP (Currency, Relevance, Authority, Accuracy, and Purpose) test to determine if these sites were providing quality information.

Results: A total of 62 terms were identified from Google Trends and searched on Google. 174 unique websites were found through the search results from all the terms. The top six websites that appeared most frequently for all the Google searches were WebMD(61), Mayo Clinic (57), MedlinePlus (52), Medical News Today (48), Center for Disease Control (36), and MedicineNet (28). The only website to rate an excellent score in both evaluation tools was MedlinePlus (DISCERN 67.5; CRAAP 21). The lowest rated website was Medical News Today (DISCERN 44.5; CRAAP 14.5).

Conclusions: Through our search of the most frequently searched health-related terms, it was evident that Google search results appear most often from six common websites. Evaluating the information from these six websites revealed that there was a wide range in terms of quality. Based on this assessment, MedlinePlus should be recommended by health information professionals and those in the healthcare field for general health information. The health information on the most popular websites is of at least average quality, regardless of which website is used. Health science librarians can use these scores to help educate their students and faculty about the differences between websites when searching for health information on Google.

Poster Number: 104

Time: Friday, July 24, 10:30 a.m.–4:30 p.m.

Finding the Value in Reference Services

Edward J. Poletti, AHIP, Chief, Learning Resources, Health Sciences Library, Central Arkansas Veterans Hlthcare Sys., Little Rock, AR; **Priscilla L. Stephenson, AHIP**, Chief, Library Service, Library Service, James A Haley Veterans' Hospital and Clinics, TAMPA, FL; **Marilyn G. Teolis, AHIP**, Clinical Medical Librarian, James A. Haley Veterans' Hospital and Clinics, Tampa, FL

Objectives: Health sciences library staff in federal agencies provide knowledge-based information to support their agencies’ missions. They provide research support to help improve health outcomes, garner research support, and reduce costs. This study examined whether information seekers considered the information provided by their federal library staff to have positively affected patient care, research project development, or healthcare decision making.

Methods: This research project builds on previous studies of the value of information services in federal libraries conducted in 2011, 2014, and 2017. In order to evaluate the impact of their reference services, Veterans Administration (VA) librarians sent surveys to those receiving research assistance during a 3-month study period, October - December 2019. Survey participants included clinical patient care providers, researchers, and health administrators. The survey tool was designed to capture immediate evaluations of the value of material provided in response to the requestors’ specific reference questions. Results were reported in the aggregate as well as by agency and facility.

Results: Clinical staff respondents: 88% said library research support was useful for direct patient care. Patient care respondents: 47% said the information could result in reduced costs for either the patient or the medical center. Research information respondents: 44% said the information assisted with their publications, and 34% said it aided protocol development. Administrative information responses: 53% said library research supported decision making; 45% said it aided policy analysis and program development. Overall – 98.5% responders received new or updated information.

Conclusions: VA Library Staff provided information that . . . impacted decision making; influenced health care providers; avoided adverse events; assisted with research funding; and provided cost savings.

Poster Number: 105

Time: Friday, July 24, 10:30 a.m.–4:30 p.m.

Flexing Our Muscles: Promoting an Academic Library Anatomical Model Collection

Nita K. Mailander, AHIP, Director of Library Services, Grand Canyon University Library, Phoenix, AZ; **Kelly O'Neill**, Library Services Reference Manager, Grand Canyon University, Phoenix, AZ

Background: The University Library serves nursing and health sciences students ranging from pre-licensure to the doctoral level. With a growing anatomical model collection that supports the anatomy and physiology curriculum, the Library sought to promote the use of the collection while also streamlining the circulation process. The models previously circulated through the Library's ILS circulation system with an analog reservation process that required students to call or visit in-person to book hourly reservations in advance. In August 2019, the Library acquired Springshare's equipment booking module to not only accommodate the unique circulation needs of the collection, but to also allow self-reservations.

Description: The Library launched Springshare's equipment booking module in the Fall 2019 semester to promote and manage the circulation of the Library's anatomical model collection, which consists of twenty different types of models, for a total of forty-three models. The new visual equipment catalog creates a digital storefront display and allows users to see the availability of all models. More flexible circulation parameters are possible, such as varying checkout lengths, than were allowed in the ILS. The Library set parameters for users to be able to reserve models up to two weeks in advance for a maximum of two hours daily and a total of six reservations per week. With the addition of the equipment module, users also remain in the same interface and can easily navigate from an anatomical model reservation to completing a study room reservation.

Conclusion: The new equipment booking module was advertised to students, faculty, and staff and has excellent usage. For the Fall 2019 semester, there were 953 unique users, 2,575 circulations, and 3,951 total reservations. Training Library staff to use the equipment booking module was straightforward, because of the easy-to-navigate layout and staff’s familiarity with room reservations. The use of the new equipment booking module has enhanced the visibility of the anatomical models and highlights this reserve collection visually in a way that is superior to a Research Guide or the ILS, while also easily allowing hourly self-reservations during the Library's open hours.

Poster Number: 107

Time: Friday, July 24, 10:30 a.m.–4:30 p.m.

If You Build It, Will They Come?

Elizabeth Frakes, AHIP, Assistant Librarian for Clinical Services, University of Utah–Salt Lake City; **Shawn Steidinger, AHIP**, Assistant Librarian for Clinical Services, Eccles Health Sciences Library / University of Utah, Salt Lake City, UT; **Christy Jarvis, AHIP**, Associate Director, University of Utah–Salt Lake City

Background: In November of 2019, the staff of the Eccles Health Sciences Library initiated a plan to revamp and revitalize the patient-focused library located in the lobby of the medical center. In recent years, the library has seen patron numbers dwindle and its library director relocate to a different institution. Two newly-hired Clinical Librarians, along with leadership, formulated a plan to remodel its physical space, embark on an outreach plan, reimagine who the library serves, and formalize library services.

Description: To ensure the consumer library reflected the original vision of the donor family to support patient education, the clinical staff underwent a brainstorming and outreach initiative to transform and update the space. Identified steps include a remodel of the physical space to include more noticeable signage, an area for private patient consultations, and a more open, welcoming entrance. Secondly, staff will engage nurse educators and social workers about collaborative space use and further brainstorming. The library will continue to serve as a business center with computers and printers as well as adding a new collection of popular materials. Lastly, the team will begin outreach and information sessions starting with a colon cancer awareness event in March. Marketing strategies could include additional flyers, information near high traffic areas such as the cafeteria and coffee shop, and face-time in clinician breakrooms.

Conclusion: Patron numbers will be collected and analyzed after the library space has been remodeled then reopened. Patron number for the colon cancer awareness event will be counted and compared with last year's event. Consultations with patients will be documented.

Poster Number: 108

Time: Friday, July 24, 10:30 a.m.–4:30 p.m.

JUNTOS for Your Health: Librarians and Health Professionals Empowering Latinos with Evidence-Based Information for a Healthier Future

Brenda M. Linares, AHIP, Health Sciences Librarian, School of Nursing, University of Kansas Medical Center, A.R. Dykes Health Sciences Library, Olathe, KS; **Mariana Ramirez Mantilla**; **Ileana Cepeda**; **Valeria Salinas**

Objectives: To address the increasing use of social media and digital technologies within the Latinx community, this project seeks to reach this population via recorded Spanish-language health educational programs (e.g., podcasts) complemented with evidence-based, Spanish language consumer health information from the National Library of Medicine. This project is a collaboration of community organizers, health care professionals, and librarians.

Methods: The project team will conduct focus groups and surveys with Spanish-speaking community members to learn about their health information needs and the online sources they currently use. This information will help identify the appropriate online platform(s), content, frequency and length of the educational health segments (e.g., podcasts). The segments will include interviews with Spanish-speaking health professionals, updates on relevant health research in plain language, and storytelling from community members sharing health-related experiences and resources. Participants will be surveyed again to see if the information was successful in meeting their health information needs.

Poster Number: 109

Time: Friday, July 24, 10:30 a.m.–4:30 p.m.

Librarian Experience, Roles, and Challenges to Conducting Systematic and Scoping Reviews in Dentistry

Nena Schvaneveldt, AHIP, Education Librarian, University of Utah–Salt Lake City; **Elizabeth Stellrecht**, Associate Librarian, Health Sciences Library, University at Buffalo, Buffalo, NY

Objectives: This study will determine the scope of experience, roles, and challenges librarians face in dental and oral health systematic and scoping reviews. The study aims to establish the current level of librarian involvement with reviews in this environment. The data obtained will inform outreach efforts to subject researchers as well as identify areas for librarians’ professional development.

Methods: The authors developed a 23-item survey based on the findings of two recently published articles about health science librarian roles and challenges in systematic and scoping reviews. The survey was distributed via electronic mailing list to librarians who were likely to have participated in conducting these reviews. After being open for two weeks, the survey was closed and descriptive analysis was performed on the results.

Results: While respondents had worked on a wide range of reviews, working with systematic reviews was more common than working with scoping reviews. Work with dental and oral health reviews were less common than non-dental reviews. Librarian roles in these reviews tended to follow traditional librarian roles: every respondent had worked in planning and information retrieval portions of reviews, while fewer respondents had worked in screening and assessing results. The most frequently reported challenges were with the lead researcher or the research team, rather than the librarian themselves, and challenges with time and methodology were common.

Conclusions: Systematic and scoping reviews are growing rapidly in popularity, and librarians are not often involved in them. However, further librarian involvement in dental and oral health reviews, either as methodologists or as information experts in more reviews, may improve the overall quality of these reviews.

Poster Number: 110

Time: Friday, July 24, 10:30 a.m.–4:30 p.m.

Librarians and Library Technicians Collaborating on Systematic Reviews?! A Survey of Perspectives

Alissa Epworth, Library Technician, Unity Health Toronto / Knowledge Translation Department, Toronto, ON, Canada; **Glyneva Bradley-Ridout**, Education & Liaison Librarian, University of Toronto, Toronto, ON, Canada

Objectives: Though it is well established that librarians bring value to systematic review teams, library technicians have largely been excluded from this process. This study was designed to determine the extent (if any) to which library technicians are currently playing a role during systematic reviews, and to investigate where librarians and library technicians see opportunities for future collaboration.

Methods: We constructed an electronic survey of multiple choice and short answer queries. The target audience was librarians and library technicians who worked in a library with any scale of systematic review service. We distributed a recruitment email inviting participation in the survey and distributed it to a selected list of eight email listservs. The listservs were chosen based on their relevance to the targeted response population. The survey was anonymous, provided the opportunity to withdraw at any time, and did not collect any personally identifying information. Participants were not offered any financial incentive to participate. Responses were collated, coded and organized by themes.

Results: In total, 170 responses were received and evenly represented librarians and library technicians. Respondents were working in a variety of library types, including academic, hospital, and government. We received perspectives from individuals who have collaborated in the past, as well as those who have not. In this poster, we will highlight the results of our survey, which includes the reported tasks that library technicians conduct when collaborating on systematic reviews. We will also discuss the challenges and successes identified, and the barriers that presently exist for library technicians wishing to engage in systematic reviews.

Conclusions: We concluded that library technicians are already involved in systematic reviews in several ways. Additionally, there is interest and opportunity for increased collaboration during this process. Finally, both librarians and library technicians recognize significant challenges as well as benefits to working together on systematic review projects.

Poster Number: 111

Time: Friday, July 24, 10:30 a.m.–4:30 p.m.

Librarians Collaborating with Clinical Translational Science Institute (CTSI) to Create Informatics Tools to Support Precision Health Research

Sarah Meyer, Assistant University Librarian, University of Florida, University of Florida, Gainesville, FL; **Nancy Schaefer, AHIP**, Reference and Instruction Librarian, University of Florida–Gainesville

Objectives: Delivery of health care is moving toward greater precision as access to vast amounts of data with more heterogeneous variables increases. Identifying datasets with variables of interest is time-consuming and challenging. To shift researcher time and effort from locating to integrating and analyzing useful datasets, two librarians have created two versions of a one-stop-shop of relevant data sets.

Methods: Librarians collaborated with members of their institution’s Clinical Translational Science Institute and Precision Public Health Work Group to identify possible topics of interest to researchers. Librarians identified potentially useful data sets on these topics and curated metadata for each dataset including title, identifiers, creators/curators, landing and access page URLs, all relevant dates (release, creation, modification, etc.), licensing, relevant content (specific data elements of value), and indexing terms (geographical regions, diseases, etc. that the data are about) on a (SpringShare) LibGuide. Use of the LibGuide for actual research projects led to further collaboration with CTSI to convert the browsable webpage to an ontology-based data catalog.

Results: High usage statistics indicate the browsable webpage is well utilized. The webpage supports browsing and investigation of data sets for utility. A primary limitation is the inability to provide users with specific data sets that contain variables of research interest. To address this, librarians collaborated with a CTSI ontologist and a technology team, which resulted in the indexing of 94 unique variables from 28 datasets for a pilot ontology-based data catalog. The improved search capability is expected to assist researchers in hypothesis generation and assessing research feasibility.

Conclusions: The ontology-based catalog’s soft release in May 2019 will be broadened as more data sets are indexed and case studies reveal potential target audiences. A head-to-head comparison of time required and ease of navigation in the two tools (browsable vs. ontology-based) in the next year is expected to justify expansion of the pilot ontology-based data catalog.

Poster Number: 112

Time: Friday, July 24, 10:30 a.m.–4:30 p.m.

Librarians on the Loose: Meeting Our Patrons Where They Are

Nena Schvaneveldt, AHIP, Education Librarian; **Brandon Patterson**, Technology Engagement Librarian; **Christy Jarvis, AHIP**, Associate Director; University of Utah–Salt Lake City

Background: While the Spencer S. Eccles Health Sciences Library is located centrally to the health sciences campus of an academic health center, librarians noticed that busy students, staff, and faculty rarely visited. Certain programs and schools reported dissatisfaction with a lack of connection with the library, partly due to distance, lack of knowledge of whom to contact for library-related questions, and lack of time. The librarians’ previous experiences led them to believe that increasing serendipitous encounters with patrons by holding external office hours may increase opportunities for collaboration as well as feelings of goodwill.

Description: In order to provide better service and outreach to students, staff and faculty in the health sciences, librarians at [library] began holding external office hours. A pilot program for office hours were held weekly for a college of nursing and a school of dentistry. Additionally, librarians began holding strategically planned office hours for a physician assistant program during key times while students prepared their scholarly projects. Librarians physically held office hours within the programs’ buildings and had open online office hours in a virtual conference room for students geographically located far from campus. We gathered reference statistics to assess for effectiveness of the hours.

Conclusion: Statistics recorded from these office hours indicate a large increase in reference transactions and requested instruction sessions. Most strikingly, the School of Dentistry’s engagement with the library has increased drastically due to librarian investment in time spent at their school, partially because of their long distance from the library. Students, faculty, and staff have appreciated librarians’ willingness to meet them in their space. Based on this, we plan to continue cultivating these partnerships and investigating opportunities with other programs.

Poster Number: 113

Time: Friday, July 24, 10:30 a.m.–4:30 p.m.

Library Curriculum Support for a Senior Nursing Research Class

Kristine A. Petre, AHIP, Health Sciences and Information Literacy Librarian, Lehigh Valley Health Network, Moravian College, Bethlehem, PA; **Karen Diane Groller**, Assistant Professor of Nursing and Public Health, Moravian College, Bethlehem, PA; **Pamela Adamshick**, Associate Professor of Nursing and Public Health, Moravian College, Allentown, PA; **Pamela Adamshick**

Background: In fall 2018, nurse faculty of a small liberal arts program revised the Applied Research class curriculum to effectively manage a larger class size; and, to better prepare senior nursing students with research skills to prepare them for nurse residency program or graduate school. A major component was a practicum project that was changed from a sample-based to literature-based project. The literature-based project was framed within a longitudinal scoping review to place more emphasis on evidence-based practice (EBP) methodologies. Faculty requested assistance from the health sciences librarian (HSL) to teach information literacy and other evidence-based practice literature searching skills.

Description: Faculty and HSL collaboratively planned the library research session and supportive resources. Students were divided into three nurse faculty-supervised research teams. Using the scoping review framework, a main population-concept-context (PCC) question was developed; What types of behaviors influencing health status in young adults have been reported? Faculty guided each team, to develop sub-questions focusing on the three priority concept areas of coping, substance use/abuse, and weight change using the PCC framework.

The librarian provided one interactive library session employing evidence-based practice search methods and search time on PCC topics within the library databases. The HSL mapped class objectives to the ACRL Framework and Information Literacy Competency Standards for Nursing. The HSL created a research guide with relevant project resources and follow-up sessions for students needing additional search help. At semester end students evaluated the HSL services and project contribution.

Conclusion: The HSL was a new position and students had were not previously taught EBP search skills, which resulted in the HSL devoting sessions to basic and advanced searching techniques. Some of the faculty were not well versed in advanced search skills and could not reinforce what was taught by the HSL. During the pilot semester, the research guide was accessed 750 times. Scaffolding literature searching skills is recommended throughout the nursing curriculum to overcome obstacles encountered, and this has buy-in from the faculty. Students also evaluated the HSL services. The data is being used to enhance the HSL's role.

Poster Number: 114

Time: Friday, July 24, 10:30 a.m.–4:30 p.m.

Library Liaison Pop-Ups at the School of Public Heath: Reaching Students on Their Home Turf

Jacqueline Cellini, Research and Instruction Librarian, Harvard Medical School, Research & Instruction, Boston, MA; **Carol Mita**, Research & Education Librarian, Harvard University, BOSTON

Background: Our library's research and instruction team recently instituted a liaison program to match librarians' areas of expertise with departments in our schools of medicine, public health, and dental medicine. We focus here on the School of Public Health liaison program.

While we develop this liaison model, our library building is undergoing a major renovation, making it more difficult to reach our public health students, faculty, and staff. To counter this, we have initiated regular "pop-up hours" at the School of Public Health to remind them about our services, resources, and building changes.

Description: Two research librarians lead our public health liaison team, which also includes history of medicine and digital services librarians. During the fall semester, we scheduled two hour blocks of time, Wednesday mornings and Friday afternoons, when liaison librarians staffed a table in the School's café. This is a location where many students congregate, collaborate on projects, and make their way to classes. During these hours, we offered free coffee and swag while we promoted our liaison program and research support services. Many were surprised to learn they could schedule search consultations, and that we offer custom classes targeting course assignments. Each week, we focused on different services and resources, with guest appearances by colleagues from other library departments. We also used this as an opportunity to talk about our renovation, and how future improvements will benefit students.

Conclusion: Between September and December 2019, during 23 two hour pop-ups, we spoke with 793 attendees. Public health students who met with liaison librarians for research assistance reported that they learned about us at our pop-ups. These conversations have led to contacts with faculty, who encouraged students to meet with us for help with literature searching. We will continue with this program in the spring semester, January to May 2020. Continuing to gather data on how many of our requests for consultations and classes are a result of these outreach efforts will inform us about the success of the program.

Poster Number: 115

Time: Friday, July 24, 10:30 a.m.–4:30 p.m.

National Institutes of Health (NIH)-Funded Research: Do Authors Always Publish in Reputable Journals?

Konstantina Matsoukas, Research Informationist III, Memorial Sloan Kettering Cancer Center, New York, NY; **Lindsay M. Boyce**, Research Informationist II, Memorial Sloan Kettering Cancer Center, New York, NY; **Marina Chilov**, Research Informationist II, Memorial Sloan Kettering Cancer Center, Brooklyn, NY; **Donna S. Gibson**, Director of Library Services, Memorial Sloan Kettering Cancer Center, New York, NY; **Kendra Godwin**, Research Informationist II, Memorial Sloan Kettering Cancer Center, New York, NY; **Johanna Goldberg**, Research Informationist II, Memorial Sloan Kettering Cancer Center, New York, NY; **Robin O'Hanlon**, Associate Librarian, User Services, Memorial Sloan Kettering Cancer Center, New York, NY; **Celine Soudant**, Research Informationist II, Memorial Sloan Kettering Cancer Center, New York, NY

Objectives: The National Institutes of Health (NIH) released “NOT-OD-18-011: Statement on Article Publication Resulting from NIH Funded Research” on 11/03/2017 to provide recommendations for identifying reputable journals. This study will determine if this guidance is sufficient for avoiding possibly predatory journals. NIH-funded research articles from our institution that do not adhere to the recommendations will be identified and their publishers/journals characterized.

Methods: A 11/08/2019 Legacy PubMed search identified 3,313 journal articles by authors from our institution that were published after 11/03/2017 and not indexed in MEDLINE. The citations were transferred to EndNote, where 1,572 records containing PMCIDs were isolated. These records were exported into Excel where the list was consolidated into 553 unique journal titles. The MEDLINE indexing status for each of these titles was verified in the NLM Catalog, generating a list of 42 journals that were not currently indexed in MEDLINE and not fully indexed in PubMed (i.e. citations selectively added to PubMed via deposit in PubMed Central). Additional data on these journals were gathered, including status/inclusion in such tools as Journal Citation Reports, PubsHub, Ulrichsweb, SCImago, Scopus/CiteScore, DOAJ, NLM Catalog, and attributes/characteristics specified in the NIH guidance for determining their credibility. Twelve journals were identified as being potentially predatory.

Results: All twelve journals were fully open access but without records in DOAJ. Of the tools referenced for inclusion as a sign of credibility, Ulrichsweb indexed six of the journals, and seven journals provided DOIs. Nine journals had existed five years or less, while only four had more than five article records in PubMed. All journals provided publisher contact information, however, four did not provide details about the peer review process, three were not clear about fees charged, and four offered a refund/withdrawal policy. Although none of the publishers were members of COPE or OASPA, three of the journals had editorial board members from our institution (of the four editors contacted, two attested to credibility).

Conclusions: Classifying publishers/journals as predatory is difficult despite efforts to develop a universal definition. Checklists help characterize attributes but are limited in usefulness, as predatory journal websites can appear reputable by including these attributes and just-launched journals may appear predatory due to being new. Instead of relying solely on checklists, libraries could better support journal evaluation efforts by engaging early career authors with what journal attributes benefit their research (e.g. article discoverability/impact) and expanding their awareness of open access journals that deliver better value.

Poster Number: 116

Time: Friday, July 24, 10:30 a.m.–4:30 p.m.

Overcoming the Pitfalls of LibGuides through Design and Value-Added Content

Simon Robins, Reference Librarian, Northwest Ohio Medical University (NEOMED), Akron, OH; **Carrie Price**, Informationist, Welch Medical Library, Welch Medical Library, Johns Hopkins University School of Medicine, Baltimore, MD

Background: The primary objective is to present two cases of LibGuide redesigns spearheaded by medical librarians at two different universities, and to describe how they represent a departure from traditional usage of LibGuides with regard to design, layout, and content. A secondary objective is to encourage librarians to think of their LibGuides as web content deserving of the same attention to design and appearance as other university content.

Description: The authors created custom HTML and CSS to stylize the LibGuides at both universities, applied usability heuristics, audited relevant content for inclusion within guides, and used university and library branding to inform decision-making. Usage of the LibGuides was measured with analytics data pulled from both sets of guides and compared against previous usage. We also informally surveyed faculty and students to assess overall satisfaction with changes made to design and layout. The majority of feedback gathered in response to the overall look and feel of the guides was positive, and users at both institutions expressed satisfaction.

Conclusion: Guides saw increased usage correlated with the inclusion of value-added relevant content in the form of information visualization and bibliometric data visualizations within discipline-specific guides. In addition, the creation of a best practices guide helped standardize design elements and ensure consistency. Improving established library guides using design principles and value-added relevant content can improve their usage among key stakeholders and the research community at large. These lessons should be taken into consideration by the library community, especially the medical library community, given their stakeholders and reach, when creating LibGuide content for users.

Poster Number: 117

Time: Friday, July 24, 10:30 a.m.–4:30 p.m.

Partnership for Success: Developing a Health Education Outreach Program with a Local Public Library

Stephanie M. Swanberg, AHIP, Associate Professor, Information Literacy & eLearning Librarian, Oakland University William Beaumont School of Medicine, Rochester, MI; **Nancy Bulgarelli**, Director, Medical Library, Oakland University, Rochester, MI; **Erin Look**; **Mithya Jayakumar**, MS2, Oakland University Beaumont School of Medicine, Southfield, MI; **Tyler Shubitowski**; **Emily W. Yuen**, MS2, Oakland University William Beaumont School of Medicine, Rochester, MI; **Rose Wedemeyer**, Director of Education Training, Oakland University William Beaumont School of Medicine, Rochester, MI; **Victoria Lucia**, Associate Professor, Oakland University William Beaumont School of Medicine, Rochester, MI

Background: Public libraries serve as a primary destination for accessing free, trustworthy health information. As such, they provide the ideal setting to teach the local community about health and health literacy. In a partnership between an academic medical library and local public library, a community outreach program was created to develop, offer, and evaluate health education sessions for child and adult users. In addition, the program served as a learning opportunity for our next generation of physicians to help plan and lead health education sessions in the community and hone their patient education skills.

Description: The partnership between the two libraries was established in summer 2018 with the intent of piloting several health education sessions over the year. To ensure high quality education, interprofessional stakeholders were approached to help create the sessions including medical school faculty, librarians, staff, and students, as well as public librarians. For children’s programs, one-hour interactive lesson plans were outlined with input from all stakeholders; medical students and a public and medical librarian served as the primary facilitators of each session. The programs were evaluated using an age appropriate three-question survey, and observations of medical student communication skills were evaluated with a standard checklist. For adult workshops, a medical librarian approached a health professional (ie: physician, pharmacologist, dietician) to co-develop and teach each session, which were assessed using a program evaluation survey developed by the medical and public librarians.

Conclusion: Over the past two years, three preschool storytimes, six K-5 programs, and three adult workshops have been developed and offered by the team at the public library with a total of 96 participants. Topics have included germs & handwashing, nutrition, exercise, the human body, sun safety, skeletal system, and healthy hearts for the children’s sessions with adult workshops covering flu & Hepatitis A, pain management trends, and fad diets. This program has been a great success fueled by a dedicated team and can serve as a model of interprofessional collaboration in educating our local community on healthy behavior and health information seeking practices.

Poster Number: 118

Time: Friday, July 24, 10:30 a.m.–4:30 p.m.

Planning for the Transition from a Subscription-Based Reference Management System

Loren Hackett, AHIP, Medical Librarian, Cleveland Clinic Floyd D. Loop Alumni Library, Cleveland, OH; **Theresa Marie Kline**, Medical Librarian, Cleveland Clinic, Cleveland, OH

Background: Due to low usage, budgetary constraints, and the transition of RefWorks to a new platform, the library decided to end its RefWorks subscription in 2018 and support our patrons through the transition of citation data to alternative systems. The library had provided access to RefWorks since March 2004, so providing support to our users, especially those who had been committed long-term, was a top priority. Our poster will detail the steps taken to manage this resource transition.

Description: We set a 12-month timeline, during which we evaluated alternative citation management systems and developed training and support materials. Included in that timeline was a plan for contacting RefWorks account holders via email and the RefWorks platform, as well as providing in-person and online support to help choose the right replacement. Alternative supported systems were Zotero, Mendeley, and EndNote.

Conclusion: Based on feedback from our patrons, the transition away from RefWorks was overall successful for our institution. However, we did experience some difficulties along the way, including patron anxiety and time constraints. Our recommendations for the undertaking of this type of project are to start planning early, communicate frequently with users, and provide detailed instructions for the course of action to both library staff and users.

Poster Number: 120

Time: Friday, July 24, 10:30 a.m.–4:30 p.m.

Prescription Reading Glasses: Use of a Temporary Collection Display to Bring User Engagement into Focus

Kimberly R. Powell, Research Impact Informationist, Woodruff Health Sciences Center Library, Emory University, Atlanta, GA

Background: An opportunity was created when a new desk was constructed at the library entrance; temporary collections could be now be shown in a display area to engage users as they exited. After an initial trial, a template and checklist were created for building collections for a 6-month display period. Library staff were invited to suggest special-themes drawing from the circulating print and e-book materials to raise awareness of special topics. Display collections were curated to appeal to faculty, staff, and students in areas of undergraduate and graduate sciences as well as in professional schools of public health, nursing, and medicine.

Description: To date, four temporary collection displays have highlighted special-theme materials:

- Graphic Medicine: Ill Conceived and Well-Drawn
- Writing Science: Recommended Books and eBooks
- Elixirs & Extracts: A Celebration of Emory's Health Sciences Writers
- Compassionate Perspectives: Personal Narratives of Science and Health Care.

Displays have contained an average of 32 selected items, with roughly 30% being ebooks. A physical display card was created to showcase each of the digital reading options. A special URL, blog post, and digital signage were created fore each of the more recent collection displays for marketing and reference purposes. Circulation statistics as well as blog post views and ebook link-outs were used to track the impact of the collection display and materials.

Conclusion: The opportunity to curate a temporary display has been well received by librarians. Four librarians not typically involved in collection outreach have curated interdisciplinary displays, incorporating both print and e-book materials from not only diverse call-numbers, but providing justifications for interdisciplinary purchase requests.

Patron interaction has been mixed, with some displays garnering a 50% increase in average material circulation and others showing no additional usage. Staff continue to discuss evaluation and assessment techniques for measuring the impact of the display, the identification of special themes of interest, and possible future directions for user engagement with available collections and display space.

Poster Number: 121

Time: Friday, July 24, 10:30 a.m.–4:30 p.m.

PROSPERO Protocols: Wait Time from Submission to Completed Registration

Michelle Demetres, Scholarly Communications Librarian, Weill Cornell Medical College, New York, NY; **Antonio P. DeRosa, AHIP**, Oncology Consumer Health Librarian, Weill Cornell Medicine, New York, NY; **Keith C. Mages, AHIP**, Clinical Medical Librarian, Weill Cornell Medicine, Weill Cornell Medicine, New York, NY; **Drew Wright**, Research Librarian, Weill Cornell Medical Library, Weill Cornell Medical College, New York City, NY; **Diana Delgado, AHIP**, Associate Director, Information, Education and Clinical Services, Weill Cornell Medicine, New York, NY

Objectives: The U.K.’s PROSPERO is the main international registry of systematic review (SR) protocols. Due to increased submissions, protocols from the U.K. are prioritized, as PROSPERO is funded by the National Institute for Health Research, resulting in international submissions experiencing processing delays. This study aims to determine the wait-time SR teams outside the U.K. have experienced when submitting protocols to PROSPERO.

Methods: A 7-question survey was sent to information professionals via the following group listervs: AAHSL; ACRL Systematic Reviews & Related Methods Interest Group; MEDLIB-L; MLA NY/NJ Chapter; MLA Systematic Review Caucus. Survey responses were captured electronically using Qualtrics. Participants were asked: if they were located outside the U.K.; if they have registered a protocol to PROSPERO in the last 2 years; and how much time this took, including any resubmissions. Participants were then asked if this wait time was a deterrent from registering again, and if they would submit a protocol to a repository other than PROSPERO.

Results: 54 responses were received, of which 34 were outside the U.K. and had submitted a protocol to PROSPERO in the past 2 years. Wait times were varied, with 33.3% still under review. Of those that completed registration “over 6 months” wait time was the most common response (23.3%), followed by “3-4 months” (20%). Resubmissions accounted for 9 votes – 6 resubmitting once, 3 more than once. Those resubmissions took an average 1-4 months additional time to registration. 33.3% of respondents said that the wait time they experienced would be a deterrent from submitting to PROSPERO again; 66.7% said it would not. However, 70% of respondents said they would submit a protocol to a repository other than PRSOPERO. For the free text answer of alternative repositories to submit, Open Science Framework was the most common reply.

Conclusions: Our results are limited by the small number of valid responses to the survey. However, with the majority of respondents experiencing a wait time of 3 to over 6 months to complete registration, it may be useful to consider alternate options for protocol registration, such as Open Science Framework.

Poster Number: 122

Time: Friday, July 24, 10:30 a.m.–4:30 p.m.

Providing Consumer Health Information to the Underserved Public at Remote Area Medical Events

David W. Petersen, AHIP, Research & Learning Services Librarian, University of Tennessee Graduate School of Medicine, Knoxville, TN; **Kelsey Grabeel, AHIP**, Assistant Director, University of Tennessee–Knoxville; **J. Michael Lindsay, AHIP**, Head of Collections & Access Services, University of Tennessee–Knoxville; **Melanie A. Dixson, AHIP**, Health Sciences Librarian, University of Tennessee, Knoxville Libraries, Knoxville, TN; **Niki Kirkpatrick**, Health Sciences Librarian, University of Tennessee, Knoxville, Knoxville, TN; **Martha Earl, AHIP**, Director/Associate Professor, University of Tennessee–Knoxville; **Cameron Watson**, Library Associate III, Preston Medical Library, Knoxville, TN

Objectives: The University of Tennessee’s Preston Medical Library (PML) sought to expand the reach of our consumer health information service in our largely rural, underserved service region. The library received funding from the National Network of Libraries of Medicine (NNLM) and partnered with Remote Area Medical (RAM), utilizing The Newest Vital Sign, a health literacy assessment tool, to better understand health literacy levels of RAM event attendees.

Methods: PML librarians and other local librarians attended the three day Knoxville area RAM event in February 2020. Researchers promoted the library’s free services through health information handouts and promotional materials. Researchers provided health information on diagnoses, medications, and health conditions. Additionally, researchers conducted health literacy assessments using The Newest Vital Sign tool to those willing to participate. This tool uses a nutrition label. Researchers asked up to six questions to help assess the health literacy level of participants, and recorded the participant’s county and zip code for each health literacy assessment given.

Results: Librarians recorded that 137 people visited the table, looking at health information handouts and displays. We received 13 completed health information request forms; people desired information on a variety of medical conditions. Of interest, the topic area requested most frequently was mental health. Sixteen participants completed The Newest Vital Sign health literacy assessment, representing twelve regional zip codes. Results showed that the majority of volunteers likely had adequate literacy.

Conclusion: Through participating in the Knoxville RAM event and analyzing collected data, researchers sampled health literacy of RAM attendees and planned more focused consumer health information outreach. The original plan was to attend two other RAM events in eastern Tennessee in the spring 2020; however, these events were cancelled due to the COVID-19 pandemic. Utilizing health literacy assessments in conjunction with RAM events is a novel way of gaining insights into attendee’s health literacy skills. The library will participate in the annual Knoxville RAM event on a continual basis.

Poster Number: 123

Time: Friday, July 24, 10:30 a.m.–4:30 p.m.

PUN-Believably Successful: Outreach Efforts That Have GROAN on Our Patrons!

Christy Jarvis, AHIP, Associate Director; **Nena Schvaneveldt, AHIP**, Education Librarian; **Donna Baluchi**, Library Supervisor; University of Utah–Salt Lake City

Background: With limited resources to devote to outreach and marketing campaigns aimed at raising awareness of library services and resources, an academic health sciences library decided to explore low-cost, in-house solutions that would engage our patrons and provide a memorable encounter with library personnel.

Description: Library staff focused on using humor to engage our audience. Beginning with a Dental School orientation, the Library handed out inexpensive “floss cards” with personalized labels claiming “Librarians have their own FLOSSIFY on finding accurate information! Ask us to help you find the evidence.” For the remaining orientation sessions for Medicine, Pharmacy, Nursing, and Health, the Library purchased a heavy-duty button maker and created buttons with library-specific puns, such as “Libraries are good for circulation”. These were distributed at tabling events, orientation and instruction sessions, and Open House activities. Button recipients were encouraged to share back groan-worthy puns specific to their discipline, with many of them being made into new buttons for the students to wear. This engagement strategy resulted in numerous positive interactions with patrons, many of which have been cultivated into lasting relationships.

Conclusion: A low-cost marketing campaign can yield significant results by focusing on the quality of the interpersonal exchange. Injecting humor into the transaction, encouraging contributions from patrons, and providing them with a tangible memento of their library encounter proved to be a winning combination at our institution.

Poster Number: 125

Time: Friday, July 24, 10:30 a.m.–4:30 p.m.

Reenvisioning a Reference Service: Cleaning Up and Moving Forward to Improve Services and Workflows

Christi Piper, AHIP, Reference Librarian, University of Colorado Anschutz Medical Campus, Aurora, CO; **Samantha Kennefick Wilairat**, Graduate Assistant, Education and Reference, University of Colorado Anschutz Medical Campus, Lakewood, CO; **Ellie Svoboda**, OER Graduate Assistant, University of Colorado Anschutz Medical Campus, Aurora, CO

Background: In 2017, the AskUs reference service on an academic medical campus transitioned from being a department wide endeavour to being the primary responsibility of the newly hired Reference Librarian. In 2017, AskUs included answering reference questions via telephone, chat, text, email, and for walk-in patrons, scheduling consultations in the Education and Reference Department, and receiving professional search requests for the Professional Literature Search Team. The majority of these services and their data were contained within the Springshare LibAnswers platform. The addition of this librarian role allowed for the opportunity to reevaluate our services and implement changes and improvements.

Description: This poster will present the practical changes that were made to the AskUs reference service from 2017 - 2019 and the associated data for each change. When hired in 2017, the Reference Librarian began by evaluating the data collected from the service from 2012, when LibAnswers was implemented in the library, to 2017 to understand where inefficiencies and opportunities were. Four initiatives were undertaken from Fall 2017 to Fall 2019 including: changing data collection methods and type of data collected to gather more usable information; revitalizing a previous initiative to utilize the FAQ functionality in LibAnswers; implementing the use of LibCal's Appointments scheduler for all members of the Education and Reference department who provide consultations; and implementing proactive chat on the library homepage and LibGuides.

Conclusion: The initiatives undertaken to improve AskUs have had varying levels of success. Final data is still pending, but initial results show that our most impactful changes have been the implementation of LibCal Appointments for scheduling consultations and proactive chat. Majority of the Education and Reference department adopted LibCal Appointments for scheduling consultations in May 2019 and there has been a decrease in the number of transactions needed to schedule patrons, which indicates there will also be a time savings. Activation of LibChat's proactive chat feature has increased chat activity by 614% for a sample time period in 2018 and 2019.

Poster Number: 126

Time: Friday, July 24, 10:30 a.m.–4:30 p.m.

Reframing and Promoting Library Services to On-Campus and Remote Medical Students and Residents

Lauren Elizabeth Robinson, Medicine Liaison Librarian, University of Kentucky–Lexington

Background: Over recent years, our land grant institution’s College of Medicine has expanded medical education to students at three satellite campuses, in addition to the main campus. The geographically disperse and distinct campuses have provided both challenges and opportunities for our medical library.

This poster will highlight how our medical library is serving the geographically disperse College of Medicine’s students and residents. In addition, this poster will showcase how the medical library and Medicine Librarian have addressed the challenges providing services and resource to remote campuses, as well as the collaborative and exciting opportunities that have arisen.

Description: In addition to discussing the transition to a more and more remote service model, the presenter will highlight:

• the collaborations and partnerships with other state university libraries and librarians in providing remote services to students located across the state;

• a creative use of guides to provide curriculum embedded library resources to students at remote campuses; and

• tools and activities that have been used in providing remote services.

In addition to these topics, the presenter will display a series of guides that have been created through collaboration with the College of Medicine. The importance of mobile accessibility and use of open educational resources will be discussed.

Conclusion: This project provides an example of how librarians can implement and grow information services to remote and growing medical education campuses. Furthermore, the presenter will provided some of the feedback given by College of Medicine students, staff, and faculty over the first year of implementation.

Poster Number: 127

Time: Friday, July 24, 10:30 a.m.–4:30 p.m.

Responding to Public Library Information Needs with a Consumer Health Information Website

Tara R. Malone, Assistant Professor and Head of Serials, University of Oklahoma Health Sciences Center, Oklahoma City, OK; **Shari Clifton, AHIP**, Associate Director & Head, Reference, University of Oklahoma HSC, Oklahoma City, OK; **Sheryl Lynn Hamilton**, Medical Librarian, University of Oklahoma Health Sciences Center - Robert M. Bird Library, Oklahoma City, OK; **Phill Jo, AHIP**, Head of Access Services/Assistant Professor, Robert M. Bird Health Sciences Library, University of Oklahoma Health Sciences Center, Oklahoma City, OK

Background: Since 2013, the Oklahoma Health Information Specialists Program (OKHIS) has helped public, tribal, and academic librarians across the state connect their patrons to reliable consumer health information. Through the program, 106 staff from 67 Oklahoma libraries have earned their Consumer Health Information Specialization (CHIS). OKHIS instructors conducted focus groups to discover how participants might have benefited from the program, as well as what they would like to see from the program in the future. The creation of the OKHIS website addresses one of participants’ most requested information needs: a platform for materials and community.

Description: Participants expressed a desire for the ability to access materials and resources, and to connect to one another to share experiences and ideas for programming. The OKHIS website provides adaptable course packets and materials; curated lists of consumer health information resources; ready-made, patron-friendly materials in one place; and a custom-built forum for participant interactions. Subject-specific lists provided thus far have included links to consumer resources about cancer, DNA and genetics, drug information, and health literacy. In addition, the site includes a function that allows visitors to request face-to-face CHIS classes, including both those already developed by OKHIS instructors and desired new topics.

Conclusion: Due to the COVID-19 crisis, website promotion and feedback have not proceeded at the planned pace. Development continues, however. Materials to be uploaded in the near future include CHIS classes on pet health and on becoming an informed health consumer, as well as state-specific consumer health teaching materials. Resource lists regarding mental health, the opioid crisis, pet health, and healthy living are under development. Further additions will be made based upon participants’ evaluation data, with the goal of providing a dynamic source of consumer health information for the state’s information professionals.

Poster Number: 128

Time: Friday, July 24, 10:30 a.m.–4:30 p.m.

Scholarly Communications Services: Ten-Year Journey at a Medical University Library

Tzu-heng Chiu, Director, Taipei Medical University Library, Taipei, N/A; **Shu-Yuan Siao**, Head, Division of Knowledge Services, Taipei Medical University Library, Taipei, Taipei, Taiwan (Republic of China); **Hsiao-Fen Yu**, Librarian, Main Library, Division of Knowledge Services, Taipei Medical University Library, Taipei, Taipei, Taiwan (Republic of China)

Background: Scholarly communication can be defined as “the system through which research and other scholarly writings are created, evaluated for quality, disseminated to the scholarly community, and preserved for future use.” At XXX Medical University Library, we believe our library should have a role on it. In 2009, the XXX set up the XXX Institutional repository and help faculty archiving and disseminating their research outputs. Since then, we worked hard to promote the concept of Open Access (OA) in the campus. This poster presents what we have done in the past decade and what will be the next step.

Description: In 2016, an Academic Hub (expert portal) of XXX was established by the XXX to increase the university visibility. In August 2017, a scholarly communication librarian was appointed by the library director. After that, we re-org the structure of our library, and a new “Knowledge Services Sector” was formed. This sector is in charge of promoting OA (both IR and OAJ), evaluating the research impact of the university, maintaining contents of the expert portal, and providing references services. From 2017, we held international OA week promotion activities every year, and payed the membership fee of BMC, helping our faculty to get 15% discount of their OAJ article process charge (APC). From 2018, we held international conference in the topic of scholarly communication every year. In May 2019, a new web page of scholarly communication was launched

Conclusion: In Oct of 2019, a new services named “preliminary inspection of Predatory Journals” began to provide. After ten-years promotion, the awareness of the OA in our campus is quite high. Among the top ten list of most submission SCI/SSCI journals in XX, more than half of them are OAJs. In the year of 2020, the Knowledge Services Sector of XXX plans to conduct an analysis, trying to find out factors which influence the OAJ paper submission behaviors of our faculty. Base on the research findings, we hope we can plan for better scholarly communication services in the future.

Poster Number: 129

Time: Friday, July 24, 10:30 a.m.–4:30 p.m.

Spilling the RCTea: Thematic Analysis of the Role of Non-Randomized Studies in Rapid Reviews

Kathryn Vela, AHIP, Health Science Specialist, Evidence Synthesis Program Coordinating Center / Portland VA Health Care System, Boise, ID

Objectives: Rapid reviews are often used to support healthcare decision-making and can include non-randomized studies (NRS) when randomized studies are not available. However, NRS can be more complex and resource-intensive to identify and synthesize than randomized studies. It is currently unclear if and how NRS complexity is managed over the shortened time frames of rapid reviews.

Methods: A sample of rapid reviews published in the last five years was extracted from Ovid MEDLINE by searching for “rapid review” in the title field; rapid reviews focused on a health care interventions were included in the sample. The sample of rapid reviews was assessed with descriptive statistics and thematic analysis for the following criteria: 1) inclusion of NRS versus randomized studies; 2) search strategy terms for NRS; 3) inclusion/exclusion criteria regarding NRS; 4) screening, assessment, and extraction of NRS; 5) strategies for prioritizing synthesis of NRS; and 6) conclusions drawn from NRS.

Results: The search retrieved 23 rapid reviews that met our inclusion criteria and of these reviews, 14 (61%) included NRS. Only one review used NRS-specific terms in its search strategy, but half of the reviews (n=14) used NRS-specific inclusion criteria. In terms of the approaches used for full-text screening, quality assessment and data extraction, rapid reviews with NRS display greater variability than rapid reviews with no NRS. Each of the rapid reviews with both RCTs and NRS (n=10) synthesized all study types together, rather than using a prioritization method that would exclude NRS from the final synthesis.

Conclusions: The results of this study highlight some of the areas of complexity that emerge when NRS are included in rapid reviews of health interventions. While there is greater variation in certain aspects of rapid review methods when NRS are included, the approach to synthesizing results of reviews with NRS is similar to reviews with no NRS. Given the inconsistency in whether NRS are included in rapid reviews and if so, how they are included and evaluated, there is a need for standardized methodology for including, evaluating and synthesizing NRS in rapid reviews. This would improve the impact of rapid reviews with NRS on decision-making in rapidly changing fields or situations.

Poster Number: 130

Time: Friday, July 24, 10:30 a.m.–4:30 p.m.

The Future of Reference: Focus on Function

Courtney Hoffner, Librarian, UCLA, Los Angeles, CA; **Antonia Osuna-Garcia**, Health and Life Sciences Librarian, UCLA Biomedical Library, Los Angeles, CA; **Bethany Myers, AHIP**, Research Informationist, UCLA, Los Angeles, CA; **Doug Worsham**, Librarian, UCLA Library, CA

Background: Last year, the UCLA Science Libraries restructured student reference training to align with our new functional organization. In the 2019-2020 academic year, we used our new training approach to innovate a team-based model for our research services. We now provide greater opportunity for graduate student employee skill development and have expanded our training for public services staff. This poster focuses on the implementation, challenges, and future plans of this new research services model.

Description: The UCLA Science Libraries provide research assistance services through a tiered-support model. Access services staff attend in-person train-the trainer sessions, enabling them to answer basic reference questions at library service points. For patrons who need additional help, staff can refer to email and 24/7 chat, and help patrons schedule consultations. Consultations are provided by our graduate students (for general inquiries across scientific disciplines) and library liaisons (for advanced research support). Our graduate students were hired through the UCLA MLIS program and trained using online modules and shadowing librarians. Patrons can schedule appointments online with either graduate students or librarians. Graduate students are typically available for same-day or next-day appointments.

Conclusion: As we plan for the next academic year, we will assess the success of the service from a patron and student perspective. We will also address some of our ongoing challenges: graduate student scheduling and turnover, access services coordination, and promotion and outreach. We intend to expand this service model by hiring more graduate students and implementing it in multiple library locations.

Poster Number: 131

Time: Friday, July 24, 10:30 a.m.–4:30 p.m.

The Medical Librarian Peer Reviewer Database: Connecting Editors with Experienced Librarians

Kate Nyhan, Research and education librarian, Yale University, New Haven, CT; **Dana Haugh**, Web Services Librarian, Yale University, New Haven, CT; **Holly K. Grossetta Nardini, AHIP**, Associate Director, Yale University, New Haven, CT; **Melissa L. Rethlefsen, AHIP**, Associate Dean and Fackler Director, University of Florida–Gainesville

Background: The problem of low-quality systematic reviews being published deserves action from all stakeholders: authors, editors, peer reviewers, funders, and readers. Librarians frequently find design flaws and gaps in the reporting of searches in published systematic reviews. A 2019 study showed that many medical librarians are in principle willing to peer review manuscripts of systematic reviews, but few librarians have been asked to perform that service (https://doi.org/10.1186/s41073-019-0083-5 -- unblinded citation to be provided).

Description: We developed a tool to match two communities: editors in need of peer reviewers for evidence synthesis manuscripts and medical librarians willing to share their expertise in searching and reporting. Modeled on the successful Early Career Reviewer Database, the Medical Librarian Peer Reviewer Database offers easy registration for librarians and the opportunity to highlight their publications -- evidence for their qualifications as peer reviewers. Editors can request access to the database to easily locate librarians to peer review manuscripts. The tool follows principles of usability and accessibility and leverages a free, already-familiar platform. It will meet the practical need, not yet solved by existing peer reviewing platforms, for a pool of peer reviewers with information retrieval expertise. The Medical Librarian Peer Reviewer Database will also raise awareness of the value that librarians add in the peer review process.

Conclusion: Our pilot database is live at https://sites.google.com/view/MLPRdatabase/. We intend to offer a soft launch in February 2020 and anticipate presenting information about reactions from librarians and editors, as well as preliminary usage analytics, at MLA 2020.

Poster Number: 132

Time: Friday, July 24, 10:30 a.m.–4:30 p.m.

The New Health Science Librarian’s Role in Supporting New Graduate Health Sciences Programs with a Limited Budget

Kristine A. Petre, AHIP, Health Sciences and Information Literacy Librarian, Lehigh Valley Health Network, Moravian College, Bethlehem, PA

Background: During the last few years, a small liberal arts college added master degree programs in Nursing, Speech and Language Pathology, Athletic Training, and a Doctorate of Athletic Training. Prior to hiring the health sciences librarian (HSL), reference librarians supported the new programs in addition to their workloads. Programs are being developed in Occupational Therapy, Physical Therapy, and a post-masters certificate in Family Nurse Practitioners. The business plans for the new programs included the addition of a health sciences librarian.

Description: Prior to the HSL starting, CINAHL was upgraded to Full Text, Cochrane Library, PsychARTICLES, UpToDate and SportDiscus were added, along with a few e-journals; and the ILS was migrated. Subscriptions existed to Libguides, Web of Science, and PsycINFO. The HSL has responsibility to assess the collection and present recommendations to cost effectively support these programs.

Upon hire, the HSL evaluated the interlibrary loan (ILL) requests, book collection, and database usage. From the data, a cost-effective plan was implemented, which included additional e-journal subscriptions and working with the electronic resources librarian to identify linkage errors that occurred during the ILS move. Analysis showed that the print collection was outdated, while the e-book collection was more current. The HSL has developed a structured information literacy curriculum to support the health sciences (HS) that includes library sessions, LibGuides.

Conclusion: Changes made by the HSL increased e-journal use and decreased ILL. Other increases occurred in HS information literacy sessions and website usage. The revenue stream from the new programs will not be robust until approximately 2025. Conversations have begun and will continue with faculty to determine priorities in collection development. The HSL is working closely with the library director on budgetary issues.

The HSL expanded online modules published to the course management system to support online learning. Future plans are continued support of HS programs and outreach to faculty; scaffold information literacy; and incorporate EBP skills into classes.

Poster Number: 134

Time: Friday, July 24, 10:30 a.m.–4:30 p.m.

The Service Satisfaction Survey of the Hospital Library Consortia (DiLib)

ChiJu Chiu, Librarian, Taipei Medical University, Division of Knowledge Services,Taipei Medical University, Taipei, Taipei, Taiwan (Republic of China); **Shu-Yuan Siao**, Head, Division of Knowledge Services, Taipei Medical University Library, Taipei, Taipei, Taiwan (Republic of China); **Yu-Ying Lee**, Librarian, Taipei Medical University Library, Taipei; **Li-chuan Huang**, Librarian, Main Library, Taipei Medical University Library, Taipei, Taiwan; **Hsiao-Fen Yu**, Librarian, Main Library, Division of Knowledge Services, Taipei Medical University Library, Taipei, Taipei, Taiwan (Republic of China); **Ya-Fang Hsiao**, , None; **Tzu-heng Chiu**, Director, Taipei Medical University Library, Taipei, N/A

Objectives: The Taipei Medical University Digital Library Consortium (TMU DiLib) was founded in 2003 and is aimed at hospital libraries with insufficient human resources, funding and space. This study attempts to investigate the service satisfaction of users and librarians of the 12 hospital member libraries, and to better understand the future recommendations of hospital librarians and users for the consortium.

Methods: This study was based on a questionnaire survey, which had two versions: one is for physicians and medical staff of the member hospital who have ever used the services of TMU DiLib (the total number is 421) and another is for hospital librarians (the total number is 14). Contents of the questionnaire include demographic and background information of the respondents, and their satisfaction of the following services: (1). System platform (accessibility), (2). Interlibrary Services and Document Delivery (frequency, timeliness, full-text availability, quality, etc.), (3). Literature Search services (usage rate, quality, timeliness.), (4). Book Lending services (usage rate, quality, timeliness), (5). Instruction services (use, quality, timeliness), (6). Overall satisfaction. The questionnaire also had an open-ended question to collect feedback and suggestion from respondents.

Results: The 421 user questionnaires were issued to our 12 member hospital libraries and 230 valid questionnaires were returned, resulting in a response rate of 55%. While the librarian questionnaires of 14 hospital libraries were returned with the response rate of 100%. The main findings were:(1) 41% users are nursing staff, and 31% users are physician; (2) there are 11 full-time hospital librarians (79%), but only 6 (43%) of them have library science related degree; (3) more than 80% users use interlibrary document delivery services frequently; (4) 29% users have participated the instruction classes, and 64% hospital librarians have participated and organized the instruction classes; Overall, 96.5% (222) users are satisfied with the services of the TMU DiLib.

Conclusion: In conclusion, the librarians and users of the member hospitals of the TMU DiLib consortium are considered that our services are very good and meet their information needs. As to the suggestion, in addition to the platform interface, document delivery services, book lending services and promotion and instruction activities, users in member hospitals hope the TMUL can increase consultation service for bibliographic management software or plagiarism comparison services in the future.

Poster Number: 135

Time: Friday, July 24, 10:30 a.m.–4:30 p.m.

Understanding Qualitative Researchers' Needs in the Health Sciences

Andrea Minjee Kang, Research & Instruction Librarian, Tufts University, Boston, MA; **Claire Myers**, Research Assistant, University of Michigan School of Information, Ypsilanti, MI; **Jacqueline Freeman**, Informationist, University of Michigan, University of Michigan - Taubman Health Sciences Library, Ann Arbor, MI; **Jacqueline Freeman**, Informationist, University of Michigan, University of Michigan - Taubman Health Sciences Library, Ann Arbor, MI; **Claire Myers**, Research Assistant, University of Michigan School of Information, Ypsilanti, MI

Objectives: Librarian roles have evolved over the decades to include more support for researchers throughout the research life cycle. While support for quantitative research has increased, there is far less support for qualitative or mixed-methods researchers. This study strives to better understand qualitative researchers' needs and how librarians can provide more support, focussing on interviews with health sciences researchers.

Methods: A team of librarians, faculty, and library school fellows hypothesized that at their large academic research university, support for qualitative researchers lagged behind that offered to quantitative researchers. They designed and implemented a study to test this hypothesis in which data were collected about the needs of researchers conducting qualitative research through semi-structured interviews with key stakeholder groups of librarians, faculty, and doctoral researchers. Interview protocols were developed and tailored to these three specific groups. A total of 29 interviews were conducted; of these, 10 were conducted with researchers in health sciences domains. Interviews were recorded, anonymized, and transcribed. A codeset was developed for analysis and NVivo software was used to complete inter-rater agreement and transcript coding. A finalized codebook and new analyses are now supporting the creation of a quantitative survey to allow for statistical modeling and analysis.

Results: Preliminary results found that conducting qualitative and mixed-methods research in the health sciences is highly collaborative and multidisciplinary. Within this environment, researchers often found difficulty communicating qualitative research concepts within disciplines that have been traditionally more quantitative. Additionally, researchers felt that more support existed for quantitative research and found the need to create communities of their own to fill gaps of knowledge in methodology, tools, data analysis, and in creating funder- and organizationally-compliant data management plans. Sensitivity of data and data sharing are topics that have particular importance to health sciences researchers conducting qualitative studies.

Conclusions: While many of the interviewees had already been in contact with librarians throughout their research processes, the fact that they still needed to create communities of their own to fill knowledge gaps in qualitative methods and tools demonstrates that more must be done in order to support these researchers. As qualitative and mixed-methods research expands in the health sciences, library support to accommodate this growth should be further explored and implemented for the future.

Poster Number: 137

Time: Friday, July 24, 10:30 a.m.–4:30 p.m.

Utilizing Peer-to-Peer Learning to Expand Expert Searching Competencies

Phill Jo, AHIP, Head of Access Services/Assistant Professor, Robert M. Bird Health Sciences Library, University of Oklahoma Health Sciences Center, Oklahoma City, OK; **Tara R. Malone**, Assistant Professor and Head of Serials, University of Oklahoma Health Sciences Center, Oklahoma City, OK; **Shari Clifton, AHIP**, Associate Director & Head, Reference, University of Oklahoma HSC, Oklahoma City, OK; **Sheryl Lynn Hamilton**, Medical Librarian, University of Oklahoma Health Sciences Center - Robert M. Bird Library, Oklahoma City, OK

Background: Peer-to-peer search seminars began in 2016 as part of an internal training program for health sciences librarians. The purpose of this program has been to share each searcher’s experiences and hone search skills by working collaboratively.

Description: Searchers meet an average of 5 hours per week, dependent upon the volume and complexity of search questions submitted by patrons. Before the session, individuals execute a preliminary search. These searches are then presented during the peer-to-peer search session. Participants work through search questions, identify logical steps in conducting a search, select relevant subject headings and natural language terms, apply Boolean connectors, formulate alternative search strategies, and discuss database/interface-distinctive shortcuts or techniques. Each searcher offers feedback and suggestions, sharing their own challenges. Additional discussion topics include customization of search outputs, distribution of search results, communication methods to patrons, and documentation of complicated search strategies. Searchers also explore subject-specific databases, point-of-care tools, and options for addressing limitations of resources. Participants in the program have varying levels of experience in health sciences libraries, and thus contribute a variety of searching perspectives.

Conclusion: Collaboratively exploring and searching in health science resources has required all participants’ active roles in sharing their strengths and weaknesses. The activities of the peer-to-peer search seminars reinforce the performance indicators of Competency 1 (https://www.mlanet.org/p/cm/ld/fid=1217) of the MLA Competencies for Lifelong Learning and Professional Success. Challenges include the large time commitment and the varying outcomes depending on individuals. Future goals include extended documentation and analysis in order to improve sessions and searcher skills.

Poster Number: 138

Time: Friday, July 24, 10:30 a.m.–4:30 p.m.

Virtual Reality Studios at the University Health Sciences Library

Annabelle V. Nunez, Associate Director, University of Arizona Libraries, University of Arizona–Tucson; **Viviana Fimbres**, Graduate Assistant, University of Arizona Health Sciences Library, Tucson, AZ; **Curt D. Stewart**, Administrative Support Assistant II, University of Arizona Health Sciences Library, Tucson, AZ; **Kathryn M. Lowers**, Library Information Associate, SR, University of Arizona Health Sciences Library, TUCSON, AZ

Background: The CATalyst Studios at the University of Arizona Main Library has a virtual reality (VR) service. Seeing its popularity grow, the University of Arizona Health Sciences Library opened the Virtual Reality Studios at UAHSL. A primary driver for this service is to support the mission of the University of Arizona Health Science’s Strategic Plan. The VR Studios will provide the opportunity for cooperative learning and collaborative networking aimed at “addressing the global health challenges of the 21st century.” Our vision at UAHSL is to offer faculty and students VR experiences that will support the curriculum and enrich student learning.

Description: In August 2019, the VR Studios opened with health sciences curriculum support and experiential learning in mind. After reviewing the literature and consulting with colleagues at the makerspace, we purchased an HTC Vive and an Oculus Rift. They are set up in two rooms, each approximately 10’ X 11’ in size. We developed operational policies and procedures and a service model. We hired student workers to manage the Studios.

We use LibCal from Springshare for users to reserve up to two hours of Studio time per day. We met with the College of Nursing faculty interested in technology in education, and as a result, we selected programs and games based on decision-making skills. Additionally, we purchased Embodied Labs, a health care experience program. We developed a Qualtrics survey to collect user feedback at the end of their VR experience.

Conclusion: To date, fourteen faculty members have taken a guided tour of the Studios. One faculty member expressed an interest in exploring how the anatomy program (3D Organon) can be used to support the curriculum in her course. The next steps include managing the VR service with safety in mind. Currently, work is being done to understand the hygiene practices around the equipment.

Poster Number: 139

Time: Friday, July 24, 10:30 a.m.–4:30 p.m.

What Are Your Three Wishes for the Library? Focusing on User Goals across Library Services

Hannah Friggle Norton, AHIP, Chair, Health Science Center Library - Gainesville, University of Florida–Gainesville; **Michele R. Tennant, AHIP, FMLA**, University Librarian, Health Science Center Library, Biomedical and Health Information Services, Health Science Center Library, University of Florida, Gainesville, FL; **Margaret Ansell, AHIP**, Associate Chair, Health Science Center Libraries, Gainesville, FL; **Mary Edwards, AHIP**, Liaison Librarian, University of Florida–Gainesville; **Jane Morgan-Daniel, AHIP**, Community Engagement and Health Literacy Liaison Librarian, University of Florida–Gainesville; **Ariel FitzGerald Pomputius**, Health Science Liaison Librarian, University of Florida–Gainesville

Background: While our library frequently queries users on their needs related to specific services or issues (e.g. technology support, diversity/inclusion, wellness programming), our users had not been explicitly asked for general feedback on the library. In order to solicit the broadest level of feedback possible, the library’s assessment team asked users to submit their three main wishes for our library with no limits or qualifications placed on those wishes.

Description: A three-question online survey, modeled off of the successful “wishes campaign” at the University of Colorado Health Sciences Library, asked for library users' first, second, and third wishes for the library. The survey was open for two months during the fall of 2019 and was distributed via links on the library’s website, Facebook page, and Twitter account and through emails from liaison librarians to faculty, students, and staff. Additionally, the survey was advertised via digital signage in the library and other nearby buildings. All announcements featured a magic wand image and the phrase “3 wishes,” for a unified and recognizable aesthetic. Where possible, the assessment team and the library’s administrative management team replied immediately to survey responses and shared these replies on the library's website; multiple responses required further consideration and were answered after the close of the survey.

Conclusion: Eighty individuals responded to the survey, describing a total of 199 wishes for the library. While a wide variety of suggestions arose, the requests most frequently related to improvements to the library space in general, furniture, food available within the library, air conditioning, and printing options. In response, the library’s management team made some immediate changes, (more whiteboards, better library closing process, purchasing human skeleton models) and added some ideas to longer-term space planning efforts and future areas of advocacy. Overall, this methodology was a useful way to gain input from library users on improvements that would directly impact them.

Poster Number: 140

Time: Friday, July 24, 10:30 a.m.–4:30 p.m.

Academics in Focus: Leganto Reading List Campus Engagement

Yini Zhu, Managing Librarian, Head of Access Services, Rutgers University, Rutgers, the State University of New Jersey, Newark, NJ; **Judy S. Cohn**, Asst Vice President & Director, Rutgers, The State University of NJ, Newark, NJ; **Mina Ghajar**, Information & Education Librarian, Rutgers, The State University of New Jersey, Rutgers University, Newark, NJ

Background: In accordance with the libraries mission to support the instructional, research, and public service goals of the University, XXX University Libraries identified the ExLibris Leganto Reading List Tool as an important objective to enhance access to required and recommended resources.

The XXX Biomedical and Health Sciences libraries promoted this initiative to course directors in the seven health sciences schools, which enables streamlined access to the libraries resources from within the learning management systems. The objective of this initiative is to engage faculty across XXX to adapt the Leganto Reading List Tool, streamlining student access to course materials.

Description: XXX course directors and teaching faculty independently create course reading lists. A streamlined implementation approach, developed by the libraries, aims for broad adoption of Leganto, with a focus on individual faculty.

We conducted individual pilot solicitations among select course directors, while concurrently pushing out broad campus-wide announcements of the new Leganto Reading List solution. We proactively set up individual faculty demonstrations and hands-on training, which led to invitations to provide group demos at departmental meetings, school-wide faculty meetings, and curriculum committee forums.

Utilizing a multi-pronged approach, evaluating feedback and modifying as appropriate to engage and improve roll-out sessions, response has been overwhelmingly positive. The ease of implementation, and ability to identify and pull-in resources from the catalog while in the LMS, is boosting access to the libraries resources, and serving a critical role in the broader educational dynamic.

Conclusion: As this is an ongoing project, a conclusion is not available at this moment. Feedback and outcome will be analyzed prior to MLA 2020, and a conclusion will be drawn by then.

Poster Number: 141

Time: Friday, July 24, 10:30 a.m.–4:30 p.m.

Bringing It into Focus: Finding an Organization’s Intellectual Capital Using a Research Approach

Julia Esparza, AHIP, Associate Director, Health Sciences Library, Louisiana State University Health Science Center at Shreveport, Shreveport, LA; **Kathleen A. Bloomingdale**, Coordinator of Academic Area, LSU Health Shreveport, Shreveport, LA; **Montie L. Dobbins, AHIP**, Assistant Director of Technical Services, Medical Library, LSU HSC-Shreveport, Shreveport, LA; **David Charles Duggar, AHIP**, Head, Library Liaison Program, Health Sciences Library, Louisiana State University, HSC, Bossier City, LA; **Sarah Jackson**, Education Librarian, LSU Health Shreveport, Shreveport, LA; **Theresa L B Nail**, Evening / Weekend Supervisor, Access Service, LSU Health Shreveport, Shreveport, LA; **William Olmstadt, AHIP**, Executive Director, Health Sciences Library, Health Sciences Library, Shreveport, LA

Objectives: Identifying the intellectual capital of your institution is crucial to administrators. The Vice Chancellor of Research at LSU Health Shreveport discovered in 2019 that there was no specific Organization-Enhanced Name(s) for the institution in Web of Science. This paper describes the best methods to identify specific citations in Web of Science to be compiled under one Organization-Enhanced Name(s).

Methods: A systematic process using a team approach to search Web of Science was done to collect over 144,654 citations. A procedure was established to search Web of Science to find the citations to examine. Training was conducted to ensure uniformity of the searches. To ensure complete collection of all institutional affiliated citations, the faculty and staff searched by [state]. The group then used data cleaning software to cull out citations that were not affiliated with the institution. Due to the many variations on affiliation name searching by institution was not possible. After several attempts, the best approach was identified to reduce the time spent on the project. Searching by zip code, location, and partial institution name in different record fields in batches was done. Manual review of questionable citations was needed at the end to eliminate the final non-affiliated citations.

Results: Currently, few Organization-Enhanced Name(s) are listed for Louisiana State University system schools in Web of Science Core Collection. Most citations are listed under broad organizational names like Louisiana State University or Louisiana State University System. The Library faculty and staff completed 226 searches to collect 144,654 citations from 1993-2018 in the Web of Science Core Collection. Using a OpenRefine a data mining tool, Library faculty identified 10,194 unique citations. These citations are being marked with a new Organization-Enhanced Name(s) to easily identify the wealth of the work being produced by the institution.

Conclusions: Intellectual capital of your institution can be located despite variance of institutional name used by authors. The Vice Chancellor was pleased with the focused result. The new Organization-Enhanced Name(s) is now being promoted as the correct affiliation name to be used on all publications and conference abstracts. The highly skilled library faculty demonstrated a new value to the Vice Chancellor of Research. This had led to the Office of Research requesting other projects utilizing the skills of the Library faculty.

Poster Number: 142

Time: Friday, July 24, 10:30 a.m.–4:30 p.m.

Building a Faculty Research Publications Database Using LibGuides

Kyle Downey, Health Sciences Librarian, Seton Hall University, Nutley, NJ; **Chris P. Duffy, AHIP**, Associate Dean, Health Sciences Library and Information Commons, Seton Hall University, Verona, NJ; **Allison Piazza, AHIP**, Health Sciences Librarian, Seton Hall University Libraries, New York, NY

Background: After conversations between the library and the School of Medicine’s Dean of Research, it was determined there was a need for a research database highlighting work being conducted by our institution’s faculty. Primarily, this database would serve to connect students interested in research with our 1000+ basic science and clinical faculty spread across a health network. In response to this need, a School of Medicine Faculty Publications Database was built in-house using the content management and curation platform, LibGuides by Springshare.

Description: The database’s citations are procured through a keyword search for our institution. The generated list is cross-referenced with a faculty list supplied by the Office of Faculty Affairs. The remaining citations are imported into Zotero, a free citation management application, and subsequently exported as a .CSV file that is further edited in Excel. The final spreadsheet consists of the article title, partial abstract, journal title, year, PMID and affiliated author. The final spreadsheet’s cells are then pasted into Tableizer!, a website that converts spreadsheets into HTML tables. Finally, the HTML is pasted into the LigGuides page, along with JavaScript code to make the table searchable. The PMID is hyperlinked to the corresponding PubMed abstract; the author’s email address is also hyperlinked. The database is updated quarterly with new citations, a process that takes one librarian 3-5 hours.

Conclusion: Since its launch in March 2019, the School of Medicine Faculty Publications Database has been viewed over 2600 times. SOM Administrators, faculty and students have all praised the platform for having met its original objective to connect SOM students to faculty engaged in research, but also for its recruitment and accreditation potential.

In October 2019, the College of Nursing expressed interest in a similar database for its publications. With the SOM database as a model, a College of Nursing Faculty Publications Database was built quickly. Utilizing LibGuides by Springshare is a convenient, in-house solution for building a faculty publication database.

Poster Number: 143

Time: Friday, July 24, 10:30 a.m.–4:30 p.m.

Building a Systematic Evaluation Strategy for Measuring the Impact of Health Information Outreach

Alla Keselman, Senior Social Science Analyst, US National Library of Medicine, Bethesda, MD; **Renee Bougard**, Project Scientist, National Library of Medicine, Bethesda, MD; **Laura Bartlett**, Technical Information Specialist, Outreach and Special Populations Branch, National Library of Medicine, Office of Engagement and Training, Bethesda, MD; **George W. Franklin**, NLM Exhibit Manager, NIH, Bethesda; **Amanda J. Wilson**, Chief, Engagement and Training, National Library of Medicine, Bethesda, MD

Background: Our organization is a national leader in health information development, dissemination, and outreach. It also conducts health information outreach and dissemination efforts through an affiliated professional network that reaches multiple communities. Recent organizational restructuring resulted in centralization of the organization’s outreach, training and engagement activities in a newly-created office. The office is building a comprehensive evaluation strategy for measuring the impact of health information outreach activities. The objective is to create a set of customizable tools that measure different outcome variables targeted by health information outreach activities and a strategy for matching outreach activities with the appropriate tools.

Description: Using a published, research-based model of the impact of health information outreach, developed by the first author, we created a list of outcome variables potentially affected by health information outreach. The list was validated through discussions with our network evaluators. Examples of variables include awareness of health information resources, health information evaluation skills, and health information behaviors. We then developed a pilot tool for measuring awareness of health information resources, programs, and organizations (three of the variables on the list). Next, we adapted this measure to evaluating the impact of health information exhibits and pilot-tested it at two major national conferences. Other, ongoing, program activities include comprehensive environmental scan of published reports of health information outreach, with the extraction of evaluation instruments, development of measures for other variables, and pilot-testing them with one of our national programs.

Conclusion: When completed, this program will produce standardized quantitative data on various aspects of impact of our health information outreach activities, such as exhibits, workshops, health fairs, webinars and more. This will allow us to obtain a comprehensive picture of how our efforts promote health information awareness, self-efficacy, health information seeking and evaluation skills, health information behaviors, and more in the populations with which we work. This will help our organization make strategic decisions concerning its outreach efforts. The instruments, easily customizable to targeted activities, and accompanying guidelines will be available free of charge to others wishing to use them.

Poster Number: 144

Time: Friday, July 24, 10:30 a.m.–4:30 p.m.

Class Registrations and Evaluations: Tips for Getting Actionable Feedback

Carrie L. Iwema, AHIP, Coordinator of Basic Science Services, University of Pittsburgh Health Sciences Library System, Pittsburgh, PA; **Rose L. Turner**, Coordinator of Liaison Services, University of Pittsburgh, University of Pittsburgh, Pittsburgh, PA

Background: Librarians put a lot of effort into preparing for and teaching classes on a variety of topics to a variety of patrons. Both instructors and class attendees want to make sure their time isn’t wasted on mismatched expectations and content. To address this concern, reference librarians at an academic health sciences library assessed and updated their online class registration and evaluation forms to capture actionable feedback and improve class offerings.

Description: Librarian instructors at our institution have expressed discontent with feedback received on class evaluation forms. Minimal useful information was captured, including pointless Likert scale ratings and a dearth of comments. Instructors were often reluctant to request feedback via the survey given the lack of actionable data gathered, perhaps due to survey fatigue. To address this issue, subject specialist librarians created a pilot project to test a more focused set of questions using Qualtrics survey software. After a few classes and examination of the quality of feedback received, a subgroup further assessed the questions and created an internal survey to capture the types of feedback desired by all of the librarian instructors. During the process it was decided that changes to the class registration form would also be useful for class preparation.

Conclusion: The original class registration form was limited to demographic information; the updated form added a question asking registrants what they hope to learn from class attendance. The class evaluation form was completely overhauled, and the number of questions was limited in consideration of improving the response rate. Questions now include a mix of multiple choice and open answer, with five of the nine questions requiring a response. The increase in response rate and quality has already impacted class design. This poster shows examples of the original and updated registration and evaluation forms and highlights questions that resulted in actionable feedback.

Poster Number: 146

Time: Friday, July 24, 10:30 a.m.–4:30 p.m.

Future in Focus Groups

Nancy Lombardo, Librarian, Head of Digital Publishing; **Nena Schvaneveldt, AHIP**, Education Librarian; **Jessi Van Der Volgen, AHIP**, Assistant Director NNLM Training Office; University of Utah–Salt Lake City

Background: This library participated as researchers and facilitators in a series of focus groups to evaluate an Interprofessional Education simulation. The results were published in a scholarly journal. This led to a number of inquiries from other qualitative research teams as to whether the library could also conduct focus groups for them. The library capitalized on this need and the central position of the library within the health sciences center to establish[pilot a focus group service in the library.

Description: The library holds a valuable position as a central neutral agency within the health sciences. Library services are available to all colleges and departments. Providing a focus group service through the library presents new collaborative opportunities between library personnel and campus researchers with a variety of qualitative research projects. This gives library focus group leaders a clearer understanding of the research themes, the research participants, and the research answers on campus, as well as opportunities to become part of the research teams. This poster will describe the service provided, provide examples of types of focus groups conducted, and discuss the value of providing this service.

Conclusion: The focus group services puts library faculty and staff directly into the research process. It has expanded our working relationships with researchers and presented opportunities to offer direct research collaboration by participating in research projects. The focus group team is gaining better knowledge of research in the campus health sciences community and has led to co-author opportunities on articles covering the research conducted. Offering a focus group service seemed a natural fit for the library as a central research resource, as well as a neutral party.

Poster Number: 147

Time: Friday, July 24, 10:30 a.m.–4:30 p.m.

Late Nights at the Library: Offering Extended Hours at a Small Academic Library

Madeline Gerbig, Instruction & Liaison Librarian; **Helen Yueping He**, Head of Dentistry Library; University of Toronto, Toronto, ON, Canada

Background: Extended access to study spaces is important for students in medical programs as they balance clinical schedules with the demands of coursework. The Dentistry Library recently received user requests for extended access to our newly renovated study space. In response, we piloted offering extended hours during the Fall 2019 term. In our evaluation, we aimed to answer the following questions:

How frequently and for what purposes were users in the library during extended hours?

What spaces and services are most important to users in the evenings?

What are users’ perceptions of extended hours and its impact on their academic success?

Description: Due to budgetary limitations, the pilot was offered only during the final six weeks of the term, as we hypothesized that late night study space is most important for students in the weeks before exams. We chose to move our closing time from 8:30pm to 11:00pm on Monday through Thursday, offering a significant service extension while minimizing additional staffing requirements. To promote the service, a combination of communication methods were employed including posters and announcements in multiple departmental publications. To assess user needs, behaviors and perceptions related to evening hours, we collected hourly statistics throughout the project and conducted a survey to solicit user feedback. Paper surveys were distributed each evening during the third week and sixth week of the service. The survey was also distributed in electronic format via multiple communication channels to reach a wider range of respondents.

Conclusion: The majority of survey participants appreciated the service and requested that it continue in the future. Library usage was high on all evenings of the week, and increased significantly in the weeks before exams. It was apparent that later in the evenings, access to comfortable, safe and convenient spaces for group and solitary study is very important to our users. Our findings will inform future efforts as we explore continuing to offer the service, and the project design and evaluation methods may serve as a model for other small academic libraries that are interested in extending access to their spaces.

Poster Number: 149

Time: Friday, July 24, 10:30 a.m.–4:30 p.m.

Participation and Perceptions of Open Access Publishing: Research on Journal Authors in an Academy of Medical Sciences

Yuwei Liu, Librarian, Institute of Medical Information/Medical Library,Chinese Academy of Medical Sciences Department of Resources Development, Beijing, N/A; **Qin Zhou**, deputy director, Library Of Chinese Academy of Medical Sciences,Collection Development

Objectives: 1.To explore the features of published Open Access(OA) articles and authors' attitude towards Open Access publishing in an academy of medical sciences;

2.To estimate how much do authors spend on OA papers' publishing per year in an academy of medical sciences.

Methods: 1. Bibliometric method.

Data collection:Selected all OA articles published between 2008-2018 in "Web of Science" by authors of the specific institution;

Data Analysis:Statistical analysis of the selected papers' quantity and quality distribution ,OA approach and level as well as their funding situation;

2.Semi-structured interviewing.Randomly sampled 7 authors of the selected articles above,and conducted a face to face interview with each.

3.Internet data collecting and estimating. Referred to the websites of 18 main OA publishers for their average Article Processing Charge,then estimated the cost of OA papers' publishing per year according to the article amounts.

Results: The total number of OA articles published between 2008-2018 was 9205,taking up 45% proportion of all SCI articles the institution published,and both of the absolute number and proportion have been rising year by year.The average Impact Factor of these 1165 journals which published the OA articles was 4.37. PLOS ONE was the most popular choice for authors researched.Among published OA articles,65.7% were"Gold OA",27.2% were "Bronze OA",7.1% were "Green OA".89% OA articles were funded.But author's cognition of concepts and models of OA was limited,and there was a lack of knowledge and skills to acquire OA resources and information.

Conclusions: 1.Currently,The OA articles take a great part of SCI articles published by authors in the institution.And there is a growth trend of OA articles' publishing in the institution;

2.The knowledge and skills of authors is not enough to support them in their practice in OA publishing and OA resources acquiring;The library should do something to improve the situation to help authors to take the initiative in the OA field.

3.Authors spent ￥18,220,000 in OA papers' publishing in 2018.There is a need of unified fund management and price negotiation for OA publishing in the institution.

Poster Number: 150

Time: Friday, July 24, 10:30 a.m.–4:30 p.m.

Piloting a Systematic Review Service and Instructional Workshop Series Featuring Free and Open Source Tools

Amanda Adams, Reference & Instruction Librarian, CMSRU Library, Camden, NJ; **Benjamin Saracco**, Research and Digital Services Librarian, Cooper Medical School of Rowan University, Camden, NJ

Background: Residency and fellowship programs at Cooper University Hospital (CUH) increasingly request librarian support to teach the systematic review process. Specifically, the surgery department requested workshops with the goal of forming teams to publish studies. Cooper Medical School of Rowan University (CMSRU) librarians developed four workshops on conducting systematic reviews from beginning to end. Due to a lack of institutional subscriptions, workshops highlighted free and open source tools. Subsequently, librarians joined SR teams where they trained collaborators on free and open source products. We continually adapt the workshops for other departments and dispel the myth that systematic reviews require expensive software.

Description: "Conducting Systematic Review Studies" workshops were created by librarians for CUH residents, fellows, medical students, and faculty, designed for those with minimal prior knowledge. Free software for deduplication, screening, project management, and research data management were highlighted. During training, participants signed up for accounts with many of the programs to learn their utility. For example, a test rayyan collection was used in an activity to practice screening articles.

Implementation: Following the surgery workshops, teams formed of faculty, librarians, residents, biostatisticians and medical students. They put the content directly into practice. Example tools used included Rayyan, Open Science Framework, Zotero, and SRDR+. Evaluation: Outcomes that will be evaluated at this project’s completion will be the publishing of the protocols and research studies, establishing an official systematic review service, and surveying institutional systematic review authors of their experiences using open source tools.

Conclusion: After completion and eventual publication of the resulting systematic reviews, the librarians plan to survey our collaborators. The intended outcome is the widespread use of free and open source tools. Qualitative interviews will assess experiences using the software taught. Continual evaluation of the open source software provides added value to researchers. The librarians involved in the teams are gathering data on how much staff time is required to be an effective collaborator, including training. This information will help set up additional parameters for future collaboration between librarians and research teams as part of a new systematic review service.

Poster Number: 151

Time: Friday, July 24, 10:30 a.m.–4:30 p.m.

RECOVER: Growing a Model for the Future of Evidence-Based Veterinary Medicine Guidelines

Erik Davis Fausak, Health Sciences Librarian, UC Davis, Woodland, CA; **Heather K. Moberly, AHIP**, Professor, Coordinator of Veterinary Services, Medical Sciences Library, Texas A&M University, College Station, TX; **Sarah Young**, Liaison Librarian, Carnegie Mellon University, Carnegie Mellon University, Pittsburgh, PA; **Megan Kocher**, Science Librarian, University of Minnesota Libraries, St. Paul, MN; **Christine Fournier**, Life Sciences Librarian for Research, Cornell University Library, Ithaca; **Laura Rey**, Library Associate II, William E. Brock Memorial Library, Texas A&M University Medical Sciences Library/University of North Texas, Bryan, TX; **Molly Crews**, Library Associate II, Texas A&M University, College Station, TX; **Kelly A. Johnson**, Veterinary Outreach and Information Resources Librarian, Cornell University, Ithaca, NY; **Kim Mears**, Health Sciences & Scholarly Communications Librarian, University of Prince Edward Island, Charlottetown, PI, Canada; **Jessica R. Page**, Head, Science Librarians Group and Hodesson Veterinary Medicine Library, Veterinary Medicine Library, Ohio State University, Columbus, OH; **Jamie M. Burkitt**, Assistant Professor, University of California, Davis - School of Veterinary Medicine, Davis, CA; **Suzanne Fricke, AHIP**, Animal Health Sciences Librarian, Washington State University, Pullman, WA; **Manuel Boller**, Senior Lecturer Veterinary Emergency and Critical Care, The University of Melbourne, Werribee, Victoria, Australia; **Erin RB Eldermire**, Head, Flower-Sprecher Veterinary Library, Cornell University Library, Ithaca, NY; **Megan G. Van Noord**, Health Sciences Librarian, University of California, Davis, Davis, CA

Background: RECOVER is the largest veterinary effort to create evidence-based guidelines. When implemented in 2012, RECOVER's guideline recommendations became the gold standard for CPR in veterinary practice. These guidelines were modeled on the methodology of ILCOR CPR guidelines (for humans) and closely parallel the systematic review process. The RECOVER guidelines are now being updated with librarian participation.

Description: RECOVER 2.0 is now underway with the goal of updating the guidelines through an improved methodology. The project includes eight teams of veterinary specialists with two librarians recruited to each team. In addition to creating reproducible, peer reviewed search strategies, librarians have been essential in developing project workflows, including managing the incorporation of secondary human literature into the search strategies. Librarians recommendation of using Covidence systematic review software to manage the screening process was adopted by the project team. Librarians have implemented data management practices that have aided in collaboration across multiple institutions, time zones and countries, as well as ensuring that shared files are accessible and communication is transparent.

At the time of submission, search strategies are being developed and peer reviewed.

Conclusion: Librarian involvement is expected to help the workflow and reproducibility of these guidelines. This is important because these guidelines will be updated frequently. This project can serve as a model for librarian involvement in producing guidelines in other areas of health sciences.

Poster Number: 152

Time: Friday, July 24, 10:30 a.m.–4:30 p.m.

Solo but Not Alone: Utilizing a Collaborative Network to Produce High-Quality Librarian-Led Research

Rachel Keiko Stark, AHIP, Health Sciences Librarian, Library, California State University, Sacramento, Sacramento, CA; **Jenessa Marie McElfresh, AHIP**, Health Sciences Librarian, Clemson University, Clemson, SC

Background: Solo librarians in both clinical and academic institutions face growing institutional demands to publish original research in librarianship. Both groups of health sciences librarians can meet this need by growing their research network. The objective of this project was to highlight and document the ways that collaboration, specifically across great geographic and institutional differences, can be an asset when engaging in research in health sciences librarianship for the solo librarian or practitioner.

Description: This poster describes the development and implementation of the research network of two solo librarians, with experiences in both hospital and general academics. The process for starting and maintaining research networks will be described, as well as tips and tools to increase the efficacy of these collaborations. Evaluation measures include quantity and quality of research outputs, increased professional engagement, and improved job satisfaction for participants.

Conclusion: By improving their research networks, both librarians report increased professional engagement, improved job satisfaction, and positive feedback from administrative supervisors regarding both research productivity and the number of collaborators. This project has also led to the mentoring and skill building of librarians that are new to the profession and/or research. The quality of the research produced by the librarians has improved, however with a control variable, effects on quantity are difficult to measure.

Poster Number: 153

Time: Friday, July 24, 10:30 a.m.–4:30 p.m.

Users in Focus: Challenges and Strategies for Data Collection in Website Surveys

Deborah L. Lauseng, Regional Head Librarian, University of Illinois - Chicago, Peoria, IL; **Jung Mi Scoulas**, Clinical Assistant Professor and Assessment Coordinator, Assessment and Scholarly Communications, Chicago, IL; **Allan Berry**, Web Services Librarian, University of Illinois at Chicago Library, Chicago, IL; **Carmen Howard**, Regional Health Sciences Librarian & Instructor, Library of the Health Sciences - Peoria, Library of the Health Sciences at Peoria, Peoria, IL

Background: The University of Illinois at Chicago Library of the Health Sciences has developed and maintains an Evidence-Based Medicine (EBM) subject guide using Springshare's LibGuides software [1]. In 2019, the research team received IRB approval to research who is using the EBM guide and better understand their motivation. The research employed various assessment tools, combining a website survey with data analytics. In this poster, the research team will share the challenges and issues encountered related to survey data collection and provide advice for using these assessment tools to measure the impact of online subject guides or other web systems.

Description: Three categories of survey challenges were encountered by the team: 1) issues over which we had no control, 2) issues that we could change/improve, and 3) issues that might have been controlled if we better understood the challenges going into the project. The IRB process and informed consent is very specific and beyond our control, as is the decision of online users to participate in a survey or not. However, how the link to the survey is presented and the length of data collection are under our control. The amount of time spent on survey development and balancing the conflict between gathering data and keeping the survey brief would have changed if we better understood the challenge of obtaining responses. The selection of an assessment model and our approach may have changed if we knew then what we know now.

Conclusion: Data analytics provides an overall behavior patterns of users. By employing a website survey, the team aimed to better understand users and their reasons for using the guide. The survey generated challenges which if planned for could improve data collection. Simple adjustments can affect the success of a website survey, such as using better design to combat website blindness and balancing the desire to know more with the need for a higher response rate. By recognizing and overcoming these challenges, librarians may effectively use website surveys, better understand their audiences, and use evidence-based data to tailor web content for users.

Poster Number: 155

Time: Friday, July 24, 10:30 a.m.–4:30 p.m.

All Working Together: Incorporating Library Staff in Instructional/Marketing Services

Charlotte Beyer, AHIP, Library Director, Rosalind Franklin University of Medicine and Science, North Chicago, IL; **KatieRose McEneely**, Electronic Resources Librarian, Rosalind Franklin University of Medicine and Science, North Chicago, IL; **Chelsea Eidbo**, Tier II Library Assistant, Boxer Library, Waukegan, IL; **Olivia Peterson**, Library Assistant II, Rosalind Franklin University of Medicine and Science, Ingleside, IL

Background: The purpose of this poster is to describe how one library used a variety of different roles within the library team to deliver instruction and coordinate marketing and outreach. This poster includes reasons why library paraprofessional staff were included into instructional services/marketing/outreach, lessons learned, positive outcomes, as well what to avoid. Also expectations for paraprofessionals, librarians, and library managers will be addressed.

Description: The people who are part of the library team are the most valuable resource which has the highest return on investment This library has 3 librarians to support 26 programs, and in 2013 created some new positions called Library Assistant II to take on projects which were between a library assistant and a librarian. Starting in 2017, the Instruction and Reference Librarian began bringing in the Library Assistant II positions to providing some more basic instruction/orientation to patrons as well as creating emails, posters, and table tents. This was a win/win for the library as not only was there more individuals to present and create materials, the library staff also provided a unique perspective as they often had more direct interactions with patrons at the Circulation Desk. This gave them a better feel for patron needs on a regular basis.

Conclusion: Clear expectations must be set so everyone gets the most out of the teamwork, and clear communication is key to success. In 2019, to better coordinate efforts, all library team members involved in instruction/marketing formed an Instruction and marketing Committee. For this model of instruction/outreach to work successfully, assessment of responsibilities and workload is vital. Managers need to avoid not only overloading their staff but also identifying the level of involvement of their staff. Using staff should enhance services, but not in place of a credentialed librarian with title and proper compensation.

Poster Number: 158

Time: Friday, July 24, 10:30 a.m.–4:30 p.m.

Creating a Common Sense Guide for the New Graduate Medical Education Librarian

Laura A. Murray, HCA Graduate Medical Education Librarian, University of South Florida Morsani College of Medicine, Tampa, FL; **Abby L. Adamczyk, AHIP**, Graduate Medical Education Librarian, Scott Memorial Library, Thomas Jefferson University, Philadelphia, PA; **Aidy Weeks, AHIP**, GME Liaison Librarian & Collections Manager, UNLV, Las Vegas, FL

Background: Medical librarians, whether newly hired or transitioning into medical librarianship, encounter many challenges in the diverse, demanding world of graduate medical education. Although a seasoned information professional, the experience of a newly hired GME Librarian was filled with surprises and challenges. Physician faculty, residents and fellows have different scholarly needs than undergraduate medical students or master’s students in other health sciences. Adding to these demands was the need to understand and navigate the varied and diverse hospital and community cultures. Curious about the experience of other GME librarians, the librarian set out to discover their approaches for supporting this group.

Description: Using the MEDLIB-L listserv, a new GME librarian asked other GME librarians what their jobs entailed. Sample questions included: How long have you been a medical librarian? Do you regularly serve faculty? Do you have a formal way of tracking your conversations, projects, and appointments? Are you publishing alongside your residents? The responses were wide, varied, and fruitful. Librarians’ responses came from across the country, sharing stories and offering suggestions of best practices. The responding librarians also expressed interest in learning best practices from others in the field. The GME Librarians’ work is as diverse as the doctors and communities they serve. By creating a common sense guide for new librarians, or those looking for best practices and shared experiences, this initiative will help further the success of individual GME librarians in their professional practice and their individual communities.

Conclusion: The poster presentation will be a compilation of the answers received thus far. Based on the broad response, a small group of librarians decided to survey information professionals serving residents, fellows, and faculty in graduate medical education. Partnering with these interested GME librarians, this survey will be administered utilizing the MEDLIB-L listserv, word of mouth, and a questionnaire at the MLA Conference (2020). Responses will be compiled as a manuscript on best practices and submitted for publication.

Poster Number: 159

Time: Friday, July 24, 10:30 a.m.–4:30 p.m.

Critical Librarianship and Core Values: Perspectives from Librarians Working at a Publishing Company

Dana Thimons, User Services Librarian, McGraw Hill, Aberdeen, NC; **Wesley Holloway**, User Services Librarian, McGraw Hill Education, New York, NY; **Ashley Sowa**, User Services Librarian, McGraw-Hill Education, New York

Background: Critical librarianship, the inflection of critical theory in library and information science, has recently been emerging in the health sciences library profession. Librarians are engaging with critical librarianship in many different aspects of their roles from instruction to cataloging to acquisitions, by addressing issues of diversity, information ethics, access to information, commodification of information, labor, academic freedom, human rights, engaged citizenry, and neoliberalism. As three librarians uniquely positioned in a customer support role at an academic publishing company, we are creating a framework to incorporate critical librarianship principles into our work and raise awareness of these issues within our company.

Description: While we do not have editorial influence over which books are being published or platform development control of site features, the stated core values of our company already include many aspects encompassed by the ideas of critical librarianship. With this as a starting point, there are many smaller actions that we can take to ensure that diversity, inclusion, and accessibility are integrated into our work of providing training, interacting with customers, and sharing feedback with other areas of the company. With this as a starting point, there are many smaller actions that we can take to ensure that diversity, inclusion, and accessibility are integrated into our work of providing training, interacting with customers, and sharing feedback with other areas of the company.

Conclusion: We will explore the literature surrounding critical librarianship, particularly in medical and science & technology libraries, and report on our efforts to create and implement a framework in our own roles. We will also provide perspectives from other professions in the industry such as sales, marketing, platform development, and editorial on how these efforts and principles relate to and intersect with their work and with our company’s core values. Overall, we hope to provide a unique perspective on how libraries and publishers can collaborate to influence positive change in the information landscape.

Poster Number: 160

Time: Friday, July 24, 10:30 a.m.–4:30 p.m.

Exploring Mental Health First Aid Training as a Library Staff Burnout Prevention Tool

Judith Ann Wiener, Associate Professor, Associate Director for Collections and Outreach; **Lynda J. Hartel, AHIP**, Director; The Ohio State University, Columbus, OH

Background: The challenges of addressing mental health and addiction issues in public libraries has been well-documented in recent years. Public libraries are dealing with related stresses by partnering with community health agencies and hiring social workers. Academic library staff work to create comfortable and safe environments for their students who are often under stress. This poster highlights evaluation results related to training programs offered to both academic and public library employees to improve recognition of mental disorders, increase their confidence in providing services to patrons exhibiting mental health needs and with this, potentially reducing their related stress and burnout.

Description: The authors led efforts to bring the Mental Health First Aid (MHFA) training program to employees in their academic health sciences library. Participant evaluations indicated that new knowledge was gained for recognizing and dealing with mental health issues and they felt more prepared to handle stressful situations. The authors, now principle investigators on an All of Us research award, were informed by their public library grant partners that their employees are stressed by the mental health issues they face in their libraries. Mental health and addiction are among the key health priorities for the All of Us program and the authors and public library leadership determined that offering the MHFA training program to public services staff in their library system could build their knowledge and confidence in dealing with stressful situations and inform them of area mental health resources.

Conclusion: Evaluations following MHFA training for both the health sciences library and public library employees indicated a perceived value in program participation, confidence in recognizing signs that someone may be dealing with a mental health problem, and feeling better prepared for the work they do professionally. If widely offered to library staff, Mental Health First Aid training can serve to make staff more confident and prepared to deal with library visitors and coworkers who are experiencing mental health problems and potentially lessen experiences of burnout related to these workplace stressors..

Poster Number: 161

Time: Friday, July 24, 10:30 a.m.–4:30 p.m.

From Idea to Published Book: The Editor’s Role

Margaret Vugrin, AHIP, Reference Librarian, Preston Smith Library of the Health Sciences, Texas Tech Univ. HSC, Lubbock, TX

Background: OBJECTIVE: Having effectively edited Life, Purpose, and Vision: A Fiftieth Anniversary History of the (INSTITUTION) University Health Sciences Center, this poster will present those activities and actions that are necessary to create a coffee-table book for a special institutional occasion.

Description: Librarians organize materials, books, citations, and provide in-depth research. This skill set is used to organize all parts needed to cover the institution's growth and development. The librarian/editor should treat the institution as a database. A table of contents is the backbone of any book. It gives direction for locating information which is then requested, gathered, coordinated and updated for retrieval purposes. An art-log is created for images.

These steps, skills and tools will be presented as will discussions with the publisher and printer. Politics, crisis management and meeting deadlines will also be addressed.

Conclusion: A celebratory institutional book is truly an interprofessional collaborative project. It certainly “takes a village” to produce such an important work for one’s institution. Our two-year project ended recently with delivery of the book just in time for an institutional gala celebration. The steps presented will assist others in the successful production of a celebratory book for their institution.

Librarians clearly have the necessary skill set to guide such a project from idea to published book.

Poster Number: 163

Time: Friday, July 24, 10:30 a.m.–4:30 p.m.

Leadership Induction through Library Assessment Reports

Jeremy Gunnoe, Assistant Director of Operations; Medical Librarian, Howard University, Washington, DC; **Fatima M. Barnes**, Executive Director, Howard University, Arlington

Background: A year ago, the author was promoted from Medical Librarian to Assistant Director of Operations for a health science library at a major metropolitan university. The library supports the colleges of medicine, dentistry, pharmacy and nursing & allied health, which includes over 1,800 students and 390 faculty and instructors. In 2019 the university implemented a campus-wide interdepartmental assessment program. Major units, including the library, were required to complete detailed assessments which were reviewed by an assigned group of peers who also offered suggestions for improvement. This was the first large-scale assignment for the author.

Description: The Program Prioritization Task Force (PPTF) was a new program implemented by the university administration to streamline processes and report outcomes in a consistent manner in alignment with the university’s mission and strategic goals. All 1,100 academic and non-academic departments participated in this program. The library was required to complete an extensive self-assessment for the past three years containing detailed data to describe the library’s operations. Additionally, library programs and services were assessed to demonstrate alignment with the mission and goals of the university. The library reports, along with site visits and interviews, were then reviewed and evaluated by a separate group of university peers. The library executive director and assistant director were also assigned to teams who peer-reviewed non-academic units given specific PPTF criteria.

Conclusion: This process was invaluable for the new assistant director. Completing the extensive assessment assisted in identifying and addressing gaps in the library’s data collection processes. In addition to the statistical and operational data the library collects for annual reports, the PPTF instrument provided insight into designing needs-based services with measurable outcomes. The report also informed the implementation of programs that would demonstrate value. Additionally, being part of a peer review team allowed the author to learn how the library fits into the university system and the opportunity to work with high level employees outside the scope of the library.

Poster Number: 164

Time: Friday, July 24, 10:30 a.m.–4:30 p.m.

LGTBQ+ Medical Subject Headings: A Timeline

Ellie Svoboda, OER Graduate Assistant, University of Colorado Anschutz Medical Campus, Aurora, CO

Background: A wealth of research, analysis, and advocacy has been done in the realm of library and information science regarding Library of Congress subject headings and the treatment of marginalized populations, specifically the LGBTQ+ community (Berman, 1971; Adler, 2009; Johnson, 2010; Ferris, 2018). A similar initiative has not been undertaken for Medical Subject Headings (MeSH). Since the modern incarnation of MeSH was introduced in 1960, the terms related to the LGBTQ+ community have changed and increased in number. In many cases, these changes correspond to changing attitudes and understanding in both US society and the medical community.

Description: This poster will present changes to LGBTQ+ MeSH terms in the form of an interactive timeline. This timeline will include annual changes and additions to MeSH terms in both indexing and terminology. The changes will be contextualized by milestones in medical literature, societal and legal events, as well as pop culture. Trends in usage of terms will also be presented. For example, the term “Sexual and Gender Minorities” was introduced in 2018, and in 2019, 408 articles were indexed using that MeSH term. Alongside these events will be the 2017 election of Danica Roem, the first openly transgender person to be elected to a state legislature in the United States. Events will be presented chronologically but will be color coded to differentiate between MeSH changes and societal changes.

Conclusion: The purpose of this poster will be to illuminate the history of LGBTQ+ related MeSH terms in a contextualized timeline. It will hopefully lay the groundwork for future analysis and advocacy. Because the format will be interactive, and open-ended, participants will be able to move through the timeline independently and observe trends and themes. Medical librarians use MeSH on a daily basis and this poster will help them gain a new perspective and frequently used tool.

Poster Number: 166

Time: Friday, July 24, 10:30 a.m.–4:30 p.m.

New to Health Sciences Librarianship: Strategies, Tips, and Tricks for New or Unexpected Health Sciences Librarians

Kelsa Bartley, Education & Outreach Librarian, University of Miami Miller School of Medicine, Miami, FL; **Jahala Simuel**, Medical Librarian and Head of Access Services, Howard University, Clayton, NC; **Jamia J. Williams**, Liaison Librarian, University of Rochester Medical Center, Rochester, NY

Background: Purpose: Provide tips and tricks for new health sciences librarians for learning the job and opportunities for professional development. This poster will showcase resources that have helped us with transitioning into health sciences librarianship from various points of entry into the profession.

Description: Three new librarians will highlight their journeys into the health sciences profession from different entry points, and how they are navigating the challenges and successes. Transitioning from academic to health librarianship, starting as a brand new librarian in health sciences from library school, and transitioning from health library staff to a health science librarian position are the three experiences that will be showcased.

Conclusion: Health care, technology, and education are changing dramatically, health sciences librarians and libraries must continue to adapt to keep up with the needs of patrons. Health sciences librarians need to learn to spot trends, anticipate the needs of their patrons, and proactively find new roles that will help the mission of their organization. New health sciences librarians need to quickly learn and adapt to stay relevant by linking their career goals and activities to their institutions’ goals for research, patient care, and education.

Poster Number: 167

Time: Friday, July 24, 10:30 a.m.–4:30 p.m.

Seeing Underwear Doesn't Help: How to Overcome Presentation Terror and Inch Out from Behind the Podium

Mary-Kate Finnegan, AHIP, Physical Sciences Librarian, Sacramento State University, Sacramento, CA; **Rachel Keiko Stark, AHIP**, Health Sciences Librarian, Library, California State University, Sacramento, Sacramento, CA

Background: Introverts have an acknowledged difficultly with public speaking, and as the profession has continued to become more forward facing, introverted librarians can have difficulties overcoming their fear of speaking in front of people. With an increased demand for libraries to do instruction, and other crowd based speaking, there is a need for self-directed programs to enable and empower introverts to develop not only speaking skills, but also learning to create comfort in those kinds of spaces.

Description: This poster will describe the process and tools used by an introverted librarian to overcome stark terror at the idea of speaking in front of people, and will focus on the elements that were used to create a sense of comfort found in speaking in public spaces. Collaborations with an extroverted librarian, the importance of networking, and constant immersion are some of the tools that are combined with traditional public speaking practices to enable the introverted librarian to claim a new sense of home in modern library professional practices.

Conclusion: Without this self-directed process, professional opportunities are limited, job options are narrowed, and personal well-being is decreased for the inverted librarian. By learning how to not only overcome but to adapt to the modern needs of the profession, librarians have more professional opportunities and other benefits. This program utilizes free and open resource materials and can be done by solo librarians or any librarian willing to be open to a little terror.

Poster Number: 168

Time: Friday, July 24, 10:30 a.m.–4:30 p.m.

Sexual Harassment at the University of California Libraries: Understanding the Experiences of Library Staff Members

Courtney Hoffner, Librarian, UCLA, Los Angeles, CA; **Elizabeth McMunn-Tetangco**, , None; **Nisha Mody**, Interim Associate Director, National Network of Libraries of Medicine, Pacific Southwest Region, Los Angeles, CA; **Jill Barr-Walker**, Clinical Librarian, ZSFG Library, University of California, San Francisco, San Francisco, CA

Objectives: Sexual harassment of academic library employees has not been comprehensively studied. We conducted a research study to understand how sexual harassment is experienced by employees who work in University of California (UC) Libraries. Our objectives were to: measure sexual harassment experiences quantitatively and qualitatively; measure opinions of library staff regarding institutional support around sexual harassment; and determine policy recommendations based on this data.

Methods: Participants completed an anonymous online survey that asked for information about sexual harassment behaviors they had experienced and/or observed in UC Libraries. Sexual harassment behaviors listed in the survey were developed through an iterative process involving feedback from our advisory team, campus advocates, and use of existing scales. Demographic information, including gender and campus, was also collected. The study team developed a study protocol detailing our study design, data analysis techniques, and project plan prior to creating and disseminating the survey. The survey data was gathered in a way to ensure anonymity for the respondents.

Results: After quantitative and qualitative analysis of our survey results, we found that:

-Addressing sexual harassment in UC Libraries is very important to 66% of survey participants.

-Over half of UC Library employees who completed the survey have experienced and/or observed sexual harassment at work.

-74% of respondents who experienced and/or observed sexual harassment did not report it.

-Fewer than 20% of people surveyed were aware of their campus confidential resources for reporting sexual harassment.

Conclusions: 49% of respondents suggested training to address sexual harassment. Other recommendations included more support from leadership, workplace culture change, consequences for harassers, and clear reporting guidelines. Our study team detailed our results through a website, in-person presentations at UC Libraries, and a webinar. We will also be sharing this study through publishing and conference presentations. We encourage anyone interested in the study's results and recommendations to work with others on their campus, including library administrations, to follow up as needed.

Poster Number: 169

Time: Friday, July 24, 10:30 a.m.–4:30 p.m.

Teaching Internet Hygiene and Professionalism in the Biomedical Library

Wynn Tranfield, Physical and Basic Sciences Librarian, Louise M. Darling Biomedical Library, UCLA, Los Angeles, CA

Background: Issues of personal privacy protections are increasingly important, and typically not addressed in traditional university coursework. Students bound for public-facing positions in the health sciences need to be particularly aware of their online visibility as they move into the professional landscape. A library within a large research institution piloted workshops aimed at addressing this information need among patrons at various levels. Issues of privacy, professionalism, and internet hygiene worked well as in a one-off workshop offered to undergraduates, graduates, and faculty.

Description: A desire to increase library-sponsored programming in the Biomedical library led librarians to create an interactive poster and twitter poll to gauge interest in several subjects. A combination of “Internet Hygiene and Professionalism” and “Cookies” received the most combined votes, so we decided to discuss both subjects in one workshop. Attendance had been an issue for past workshops, so in an effort to increase attendance we scheduled a shorter workshop during lunchtime. Course content outlined why privacy was important, recent legislation, and how the cookie impacts online experiences. We also examined password protection best practices. To make the workshop more engaging, we used Sesame Street characters to outline themes. For example, Oscar the Grouch represented forgotten accounts left vulnerable to hacks. Bert & Ernie represented professional and personal internet personas. A Libguide was created to compliment the presentation.

Conclusion: So far, there has been one successful workshop reaching undergraduate, graduates, and post-grads -- the course will repeat quarterly. The themes and information were well received, and students left excited to take steps toward improved internet hygiene. In evaluations, all claimed the workshop provided them with new information. Other research institutions can take steps to adopt a similar curriculum for one-shot workshops, meeting this information need for their patrons.

Poster Number: 170

Time: Friday, July 24, 10:30 a.m.–4:30 p.m.

Words in Focus: Piloting a Librarian Writing Group

Kimberly R. Powell, Research Impact Informationist, Woodruff Health Sciences Center Library, Emory University, Atlanta, GA

Background: At many academic institutions, faculty librarians are encouraged or required to make scholarly contributions. At academic health sciences centers, the published journal article predominates as the form in which the library’s users most frequently define their own successes in this area. However, the journal article can also be one of the more daunting scholarly outputs which librarians engage in for their own work. To increase the number of librarians regularly engaging in academic writing, a librarian writing group was piloted at one academic health sciences library using Wendy Laura Belcher’s book “Writing Your Journal Article in Twelve Weeks” (ISBN 9781412957014).

Description: Fourteen librarians were invited to participate; eight librarians completed the pilot in Spring 2018. Belcher’s workbook was selected as a guide and participants rotated leading discussions. The bi-weekly meetings aimed to develop habits and approaches to regularly engage in writing and break down the common planning components of the journal manuscript. Surveys at the beginning and end of the pilot measured the impact of the writing group in helping participants meet their writing goals and the extent to which participation in the group increased participants’ confidence towards completing a manuscript for publication.

Conclusion: All participants reported increased confidence, with four participants submitting a manuscript for publication by the end of the program. Administrative support was found to be a key factor in success as well as a major influence in the positive feedback from the program participants.

Poster Number: E1

Time: Friday, July 24, 10:30 a.m.–4:30 p.m.

Content Analysis of Diversity in Association of Academic Health Sciences Libraries (AAHSL) Mission Statements

Mellanye J. Lackey, AHIP, Director, University of Nevada Las Vegas Health Sciences Library, Las Vegas, NV

Purpose: This research project analyzed Academic Health Sciences Libraries’ (AHSLs) online mission statements, diversity mission statements and other statements, for diversity, equity, inclusion, and accessibility content.

Methods: Included were all full, associate, and developing members, regardless of geographic location, in the AAHSL 2019 member directory. Excluded were non-academic HSLs and one AHSL without a website. Websites of 164 AHSLs were searched for mission statements, diversity mission statements, and other statements which included DEIA content in February 2020. Data were collected using a google survey and google sheets. The research team counted AHSLs with, without and using a parent organization’s mission statements, diversity statements and other statements. Only content from the AHSL (not parent) organization was considered. The team created an initial list of DEIA words to code DEIA content in the statements. Content was coded by a single researcher, and as new terms were identified, they were added to the list.

The research team coded data for DEIA qualitative themes for tone (uplifting/positive, discriminating/negative, a combination of positive and negative, or neither), clarity (clear DEIA, possible DEIA, or no DEIA words) and intended audience (outside, inside, both, not specified).

Results: From 164 AHSL websites, 87 had mission statements, 44 had no mission statements, and 33 linked to parent organizations’ mission statements. Six of the 164 AHSLs had diversity mission statements on their websites, and 14 AHSLs linked to parent organizations’ statements. Of the 87 mission statements, 11 had clear DEIA content, 45 had possible DEIA content, and 31 had no DEIA content. Twenty-four AHSLs posted other statements with diversity content, while 35 AHSLs linked to parent organizations’ statements. The most frequent DEIA words were variations of community, access, diverse, or inclusion. Possible DEIA meaning of these words and others was unclear, since they may have other meanings dependent on context.

Tone was overwhelmingly positive/supportive in statements, and most statements addressed audiences outside the library such as students, healthcare practitioners, or the university. Internal library audiences were addressed with regards to professional development or continuous learning.

Of note, data were collected before recent protests over the deaths of George Floyd, Breonna Taylor and police brutality, among other BLM issues. AHSLs may have added or changed DEIA content during or after the protests.

Conclusions: Research data quantified DEIA content and explored selected themes in AHSL mission statements, diversity mission statements, and other statements which included DEIA elements.

Poster Number: E2

Time: Friday, July 24, 10:30 a.m.–4:30 p.m.

Graphic Medicine Assessment

Ariel FitzGerald Pomputius, Health Science Liaison Librarian, University of Florida–Gainesville

Poster Number: E3

Time: Friday, July 24, 10:30 a.m.–4:30 p.m.

LGBTQ+ Cultural Competency Integration into a Dental School Curriculum

Holly Thompson

Background: LGBTQ Cultural Competency concepts in academic dental training programs has slowly garnered more interest, but is still far behind medical school and other training programs. After a local incident on campus where a transgender patient at the dental clinic was harassed by both a staff member and student the librarian was approached by a concerned faculty member to develop a training to improve student awareness of LGBTQ topics and issues, especially as they pertain to receiving clinical care.

Methods: The training was integrated into a Behavioral Dentistry course. In order to maximize in-class time for discussion it was decided we would create an online, asynchronous tutorial. The tutorial built on workshops coordinated by another librarian at USC and gathered additional information from other resources, including the Human Rights Campaign, the Safe Zone Project, and Lambda Legal. The tutorial had a built in pre- and post-test to gauge student improvement. The tutorial was built in Qualtrics and embedded text, video, links to resources, case scenarios, and short quizzes throu: hout. The tutorial was distributed via the Blackboard learning management system.

Results

In Fall 2019 118 out of 144 students completed the tutorial. Although there was improvement between the pre and post test scores, the results were not statistically significant. Conclusions: Without requiring responses to answers and failing to collect any kind of information to hold students accountable we found students did not engage fully in the tutorial. The pre and post test questions did not yield meaningful outcomes. Discussion: Assumptions have been made the students are already familiar with these concepts and that it is staff and faculty who would benefit most from this training. In fact it is now being adapted for that audience. The training has been modified to require responses and collect student information for accountability purposes only, responses are deidentified. Participation has already improved.

Poster Number: E4

Time: Friday, July 24, 10:30 a.m.–4:30 p.m.

Research Capacity of Academic versus Non-Academic Hospital Librarians Serving Graduate Medical Education Programs

Tanisha N. Mills, AHIP, Medical Librarian, Northeast Georgia Health System, Gainesville, GA

BACKGROUND: Community (non-academic) hospitals are joining the ranks of "teaching" status by implementing graduate medical education [GME] programs in order to offset current and projected physician shortages in the United States. Both academic and non-academic teaching hospitals are tasked with supporting Accreditation Council for Graduate Medical Education [ACGME] requirements for scholarly activity, yet professional literature does not reflect if and/or how the respective research capacity of librarians at each type of institution contributes to this accomplishment.

RESEARCH QUESTIONS: 1.) What is the difference in research capacity between librarians at non-academic and academic hospitals serving GME programs? ; 2.) How does librarian research capacity impact scholarly activity support for their respective GME programs?

METHODS: Quantitative approach to surveying librarians serving hospitals with ACGME-accredited residency programs; composite instrument e-mailed to professional association listservs for recruitment; data to be analyzed using descriptive statistics, with inferential statistics in progress.

RESULTS: There were 107 total responses. Two “filter” questions left 96 respondents: 38% worked in academic hospital libraries and 46% served non-academic hospital libraries. Initial analysis indicated that “There is no difference in research capacity or impact on GME scholarly activity between librarians at academic vs. non-academic hospitals with GME programs”. Statistical testing of sub-questions is in progress to determine any significant differences.

Poster Number: E5

Time: Friday, July 24, 10:30 a.m.–4:30 p.m.

Assessing Health Literacy in Nursing Students

Ying Zhong, NSME & Web Services Librarian, California State University, Bakersfield, Bakersfield, CA

Health information literacy is an important component of information literacy and receives growing attention as the need for healthcare workers rises and the knowledge base for all those working as health professionals continues to expand. This study investigates information seeking behaviors and confidence by surveying nursing students in hybrid and fully online classes as well as traditional face-to-face classes. Students were asked to self-report their confidence level in 7 different aspects of health information literacy. 6 questions were asked to help researchers understand nursing students’ information seeking behaviors.

88 surveys were completed with a response rate of 35%. 80% of study participants reported feeling fairly or very confident in their ability to find Evidence-based resources and peer-reviewed journal articles. 73% of students felt confident giving patients’ health information. 69% of students felt confident gathering statistics on health data. Majority nursing students used the library website to find information from home.

Many students were using the online portal to the library to access resources, therefore nursing librarians should work with nursing students through multiple platforms but with an emphasis on the digital, this is especially true when Covid-19 outbreak shifted most instruction to online learning.

Poster Number: E6

Time: Friday, July 24, 10:30 a.m.–4:30 p.m.

Do Medical Librarians Search for Clinical Trials When Working on Systematic Reviews, and If So, How?

Jennifer C. Westrick, AHIP, Library Research Information Specialist, Library of Rush University Medical Center, Chicago, IL

Introduction: Librarians who work on systematic reviews are often tasked with creating a search strategy that locates many types of clinical trials. This article reports the results of a survey that examined how often experienced medical librarians are asked to find clinical trials, their comfort level in doing so, and the methods and tools they employ.

Methods: A 21-question survey was sent to experienced medical librarians via several email listservs. Respondents were limited to those librarians who make the decisions on search terms for systematic reviews. Responses were collected for one month (April 2020). The survey was reviewed and exempted by an Institutional Review Board.

Results: 136 responses were collected. Responses indicate that librarians are often asked to search for various types of clinical trials and that there is not a consistent method for doing so. While these experienced librarians consider themselves to be proficient in locating trials, there is a demonstrated need for more resources about the types of clinical trials themselves.

Conclusion: Resources about the types of clinical trials need to be developed to better support librarians who work on literature searches for systematic reviews.

Poster Number: E8

Time: Friday, July 24, 10:30 a.m.–4:30 p.m.

Health Sciences Librarians’ Engagement in Work-Related Reflection: A Qualitative Exploration of Why They Invest in Reflective Practice

Jolene M. Miller, AHIP, Director, Mulford Health Science Library, University of Toledo, Toledo, OH

OBJECTIVE: Published literature about how librarians engage in reflective practice tend to report on the author’s personal experience with reflective practice, or the results of literature reviews or quantitative studies. This poster presents preliminary findings of a phenomenological study that explored how and why health science librarians use intentional reflection to better understand work situations with the purpose of performance improvement. The goal of the research was to capture how health science librarians use reflection for work in their own words.

METHODS: Potential interview participants were recruited by an invitation to email distribution lists focusing on medical and health science librarianship. Six librarians were interviewed before the research was interrupted by the COVID-19 pandemic. The interviews ranged from 45-60 minutes were conducted using video conferencing software. They were transcribed and analyzed manually using an open-coding scheme.

RESULTS: Two main themes have arisen so far. First, participants described a variety of benefits of using reflection at work that were enough to overcome barriers such as a lack of time. Second, participants talked about the importance of being intentional about reflective practice.

CONCLUSIONS: While themes are starting to arise from the data, the study has not yet reached the point of data saturation. It is unclear at this time whether current findings will be transferrable to the population of health sciences librarians. Interviews continue.

Poster Number: E9

Time: Friday, July 24, 10:30 a.m.–4:30 p.m.

Integrating Evidence-Based Medicine Skills into a Medical School Curriculum: A Quantitative Outcomes Assessment

Laura Menard, Assistant Director for Medical Education and Access Services, Indiana University, Indianapolis, IN

Introduction: Faculty are increasingly being called upon to teach medical students the skills necessary to have a strong foundation in evidence-based medicine (EBM) before they move on to the professional phase of their education. All too often, the expectation is that these skills can be imparted in a class or two when the curriculum allows. Prior to the inception of this study, our institution had been teaching EBM within a discrete 2-month time period during medical students’ first year. During a large-scale curricular overhaul, the approach to teaching EBM was changed to a more scaffolded, integrated approach with sessions being taught over the course of two years. In this study, we assess the differential impact of these two approaches to teaching EBM in the first two years of medical school.

Objectives: This research project aims to determine the potential differential impact of two curricular approaches to teaching EBM on student performance on an EBM assignment administered during the first year of clerkship.

Methods: In order to assess and compare student learning under the different curricula, the PI and a team of five faculty members used a modified version of the previously validated Fresno rubric to grade three years’ worth of EBM assignments given to students in clerkship rotations 1-3 (n = 481) during the Internal Medicine clerkship. Specifically, EBM performance in three separate student cohorts were examined. Assignments were anonymized before being randomly assigned to graders, and all possible student and date identifying information was removed.

Results: Four hundred and eighty-one assignments were graded. Mean scores were compared for individual questions and cumulative scores using a one-way Welch ANOVA test. Overall, students performed .99 of a point better on the assignment from Year One (Y1), prior to EBM curriculum integration to Year Three (Y3), subsequent to EBM integration ($p = <.001$). Statistically significant improvement was seen on questions measuring students’ ability to formulate a clinical question and critically appraise medical evidence.

Conclusions: Results of this study suggest that taking a scaffolded, curriculum-integrated approach to EBM instruction during the pre-clinical years increases student retention of and ability to apply EBM concepts to patient care. Although it is difficult to fully attribute students’ retention and application of EBM concepts to the adoption of a curricular model focused on scaffolding and integration, the results of this study show that there are value-added educational effects to teaching EBM in this new format.

Poster Number: E10

Time: Friday, July 24, 10:30 a.m.–4:30 p.m.

Health Sciences Librarian Competency Perceptions: A Survey of National Community College Librarians

Sandra C. McCarthy, Faculty Librarian, Washtenaw Community College, Ann Arbor, MI

Objective: The purpose of this study is to determine whether community college health sciences librarians responsible for nursing and/ or health sciences collections and services perceive that they are proficient in the essential skills, knowledge, and abilities that are necessary for the practice of a health information professional as defined in the Medical Library Association Competencies (MLA) for Lifelong Learning and Professional Success. In addition, the study identifies what barriers and how engaged community college health sciences librarians are with developing their competencies.

Methods: A survey was posted to Medical Library Association (MLA) Nursing and Allied Health Resources and Services (NAHRS-L), all regional MLA Chapter groups, select American Library Association (ALA) community discussion boards via ALA Connect, and the Tribal College librarians institute (TCLI-L). Also, volunteer follow-up interviews were conducted via GoToMeeting and email.

Results: The survey was completed by 75 community college librarians and seven follow-up interviews with librarians public and private US community colleges. Results from the study report self-perceptions of community college health sciences librarians consider themselves proficient in the six competencies with as majority score of “intermediate proficiency” or higher for each question. Community college librarians face the same barriers as other librarians for travel, publishing, and engagement and are engaged within the profession.

Conclusion: This study affirms that community college librarians who are responsible for collections and services in the health sciences self-assessment meet the MLA competencies The societal benefits are that the study will fill a gap in the literature that identifies how health sciences community college librarians develop professional proficiencies as defined by the MLA Competencies. They are very involved with professional associations other than MLA. Furthermore, these results show that community college health sciences librarians can improve their skill levels in all six MLA competencies by continuing their education and following trends in the literature.

Poster Number: E11

Time: Friday, July 24, 10:30 a.m.–4:30 p.m.

An Examination of Medical Faculty and Librarians’ Perceptions of the Function and Role of Librarians within the Academic Medical Institution

Sa'ad Laws, Education & Reference Librarian, Weill Cornell Medicine - Qatar, Doha, Ad Dawhah, Qatar

Understanding the role and value of medical librarians within the institute is an important element for medical librarians in forming a coherent social identity. For many librarians, the pursuit of faculty status is a means of institutional validation, however it is often gained with the expectation to fulfill criteria that are mostly germane to academic faculty that may not be natural or valid for librarians who fulfill a different need and function in the institute. This study serves as an exploratory work into how librarians at US-based medical academic institutions formulate their identity and role within the institute. 64 Librarians and 19 clinical faculty at academic medical institutions completed surveys. Results suggest that both groups have, generally, good perceptions of the other, however librarians may be failing to expose outside groups to their research and utility and that faculty are largely unaware of the activities of librarians outside of their immediate interactions with individual librarians. Further, there is an exploration into the nature of librarian-faculty collaborations and the implications for productive relations between both groups.

Poster Number: E13

Time: Friday, July 24, 10:30 a.m.–4:30 p.m.

Exploring Feelings of Professional Fraudulence: Patterns, Trends, and Lived Experiences of Health Sciences Librarians with Impostor Phenomenon

Hilary M. Jasmin, Research and Learning Services Librarian, University of Tennessee Health Science Center, Memphis, TN

Objective: To capture the essence of the experiences of mid-career academic health science librarians and their experiences with impostor phenomenon (IP) through phenomenological inquiry.

Methods: Through a call for participation via listserv, 24 participants were acquired for semi-structured interviews. Inclusion criteria was limited to academic health science librarians with five or more years of experience. Interviews were recorded via Zoom and transcribed through Temi, an online transcription platform. Finally, NVivo was used to thematically analyze the qualitative data.

Results: Though twelve primary themes were of initial interest, the phenomenological essence of IP was a lack of comfort and confidence in a medical, STEM-forward environment. This primary theme was supported by non-STEM undergraduate degrees, feelings of "accidentally" becoming a health science librarian, and thoughts of inferiority when interfacing with medical professionals.

Conclusions: IP may not dissipate completely over time. Acknowledging that everyone feels some level of fraudulence at some point in their professional lives may allow for more honest conversations and opportunities. Given the travel restrictions of COVID-19, online STEM trainings may be of benefit to the profession. Additionally, mentorship was a noted theme as a key solution for moving past feelings of IP.

Poster Number: E15

Time: Friday, July 24, 10:30 a.m.–4:30 p.m.

Evaluation of Library Usage and Attitudes of Residents and Fellows: Results of 2017-2019 Exit Surveys

Mary Pat Harnegie, AHIP, Library Manager/Medical Librarian II, Cleveland Clinic, Olmsted Falls, OH

Objective- In this era of justifying costs to hospital administration, the library wanted to measure what services are used by residents and fellows and attitudes regarding library services. Measuring usage and attitude by this stakeholder group adds proof of effective services administration and areas for innovation. In addition, there is a gap in the literature as there is little research exploring residents’ use and attitudes of library resources on a longitudinal basis.

Method- A survey of exiting residents from 2017-2019 was conducted to evaluate library usage and attitudes of residents/fellows. A twelve question survey explored who was using the library, what core library services were used, what instructional services were used, and where and how were the resources accessed. The survey asked suggestions for improvement, services that should be offered, and services that were most appreciated. The results were recorded and analyzed using Select Survey software. The survey was completed by 823 of 1305 possible participants- 63% completion rate. Library accountholder status was assessed at departure- did you have a library account/not. No personal identifiers were used. The library accountholder status allowed a look at the usage habits of residents who had library accounts vs. no library accounts.

Results-

Interlibrary loan of articles used by 2 of every 3 residents (60%).

Studying in the library used by 1 of every 2 residents (50%).

Help from library staff was sought by 1 in 2 residents (50%).

A librarian prepared a literature search for 1 in 3 residents (33%).

Interlibrary loan of books used by 1 in 5 residents (20%).

Where and how residents accessed library resources was measured with the highest usage within the library, elsewhere on campus, and from home. Frequent access occurred using EPIC and mobile phone and while rotating at other hospitals. Over 65% of the respondents received a library orientation/overview of library resources training while 25% did not have an orientation.

PubMed training was received by 33% and bibliographic databases training was received by 16%.

Improvement recommendations were that the library be open longer hours, that departing resident retain library access, expand the pediatric collection, and expand available study rooms. The overwhelming favorite feature of the library services was the extensive collection and the ease of access for research and study materials. Other top 5 features were “helpful staff”, “physical space of the library”, “librarian-assisted lit searches”, “interlibrary articles”, and “convenient location”.

Poster Number: E16

Time: Friday, July 24, 10:30 a.m.–4:30 p.m.

What Are the Differences between Student Assessment Approaches for Determining Library Usage and Barriers among Incoming Public Health Students?

John Bourgeois, AHIP, Reference Librarian, LSUHSC - New Orleans, New Orleans, LA

BACKGROUND: In librarianship, as in most other social sciences, surveys have become the default method of acquiring information about a target population. For libraries though, little research has been done as to how to administer questionnaires efficiently. By building on previous research, this poster looks at how response rate and how response similarity differ across two modes of survey administration to populations with the same demographics, i.e. students just beginning a graduate program at a school of public health.

METHODS: In the 2017 Fall Semester, new School of Public Health affiliates registered with the library. Afterward, those new registrants were recruited to participate in a semester long research project that sought to understand their experiences and expectations with library resources. Electronic questionnaires were sent out monthly, and respondents were given a $5 gift card per questionnaire completed. Of the 39 affiliates approached to participate, 15 completed the entire battery of questionnaires – 4 questionnaires in total. This project sough to find a more efficient way of collecting the same information. For the 2019 Fall Semester, the current research protocol was far simpler than the previous one. There were two data collection events: at new student orientation and at the end of a mandatory first semester class. Also data collection occurred in-person with paper administered questionnaires. The questionnaires were pared down to a single piece of paper in order to facilitate easier physical management of the materials.

RESULTS: In the 2017 analysis, at the beginning of the semester 27 SPH students responded to the electronically administered questionnaire. In 2019, 23 responded to the in-person administration of the survey during orientation. This represents a response rate of 92% in 2019 and 68% in 2017. Analysis from this first administration shows differences between the 2017 and 2019 groups. Mostly the 2017 group expected to use quiet spaces more, according to the initial administration. In the later administrations, reported usage of library resources showed an increase in the library’s database subscriptions in 2019; there was no corresponding increase for e-books. Other differences, such as difficulties experienced, were minor.

DISCUSSION: When possible, in-person administration is preferable. Paper yielded similar results as electronic administration with a higher response rate. The differences noted could possibly be explained by changes in library operations and in SPH curriculum. More research is needed into the effects of in-person electronic administration to maintain response rate but to lessen the post-processing burden.

Poster Number: E19

Time: Friday, July 24, 10:30 a.m.–4:30 p.m.

Prevalence and Incidence of Sexual Harassment in Medical Libraries

Stephanie M. Shippey, AHIP, Associate Managing Director, Texas Tech University Health Sciences Center Library, Lubbock, TX

Poster Number: E20

Time: Friday, July 24, 10:30 a.m.–4:30 p.m.

Assessing Student Perceptions of Curriculum Resources

Karen Heskett, Instruction Librarian, UC San Diego, La Jolla, CA

Intro: In the tightly packed space of medical education, librarians seek the most effective ways to reach their students especially in support of problem-based learning. With no time to meet with students, online guides and online instruction becomes the new goal. But, if one is created, will students use it and continue to use it?

Method: This study seeks to determine if online instruction via an online guide (Libguide) makes a difference in the types of resources students cite as well as how the students interact with the online guide. In addition to a student survey, CrazyEgg's heat map tool and Springshare statistics will be used to note usage of the online guide.

Results: Nearly unanimous agreement was found to the statement I learned about new resources. However learning and applying new skills was split between agreement and neutral/disagreement. Usage or view statistics showed good use of the guide for the year with 5,931 views. Looking at the interactions for one case late in the quarter showed 156 views with 57 returning users, 9 new users, and a quick selection and click on a resource taking less than 20 seconds.

Conclusions: The survey response, while providing equal number of users and non-users, was low with only 38% response rate. It did provide insight into the acceptability of using Libguides for small doses of online instruction. Student responses provided actionable items that could help improve the guide but their list of most helpful/least helpful sections included mostly the same items. Two items did emerge: 1) Links to favorite resources are useful, and 2) citation examples are not. Those who did not use it preferred resources that may or may not serve them well as they move into the clerkship and practice setting. The heat map showed interesting usage of the guide beyond the basic view statistics available in Springshare including more specific views of the current case, returning as well as new users, resources accessed and how quickly they were selected.
